# Supplementary material for: Antiinflammation Derived Suzuki-Coupled Fenbufens as COX-2 Inhibitors: Minilibrary Construction and Bioassay
Source: Molecules. 2022 Apr 29;27(9):2850. doi: 10.3390/molecules27092850 (PMC9105197; doi:10.3390/molecules27092850)

Antiinflammation derived Suzuki-coupled fenbufens as COX-2 inhibitors: minilibrary construction and bioassay

Shiou-Shiow Farn,[1,2,†] Yen-Buo Lai,[1,†] Kuo-Fong Hua,[3,†] Hsiang-Ping Chen,[1] Tzu-Yi Yu,[1] Sheng-Nan Lo,[2] Li-Hsin Shen,[1] Rong-Jiun Sheu,[4] and Chung-Shan Yu[1,4*]

[1]Department of Biomedical Engineering and Environmental Sciences, National Tsinghua University, Hsinchu 30013, Taiwan

[2]Isotope Application Division, Institute of Nuclear Energy Research, Taoyuan 32546, Taiwan

[3]Department of Biotechnology and Animal Science, National Ilan University, Ilan 260007, Taiwan.

[4]Institute of Nuclear Engineering and Science, National Tsinghua University, Hsinchu, 30013, Taiwan.

[†] equal contribution

* To whom correspondence should be addressed. csyu@mx.nthu.edu.tw

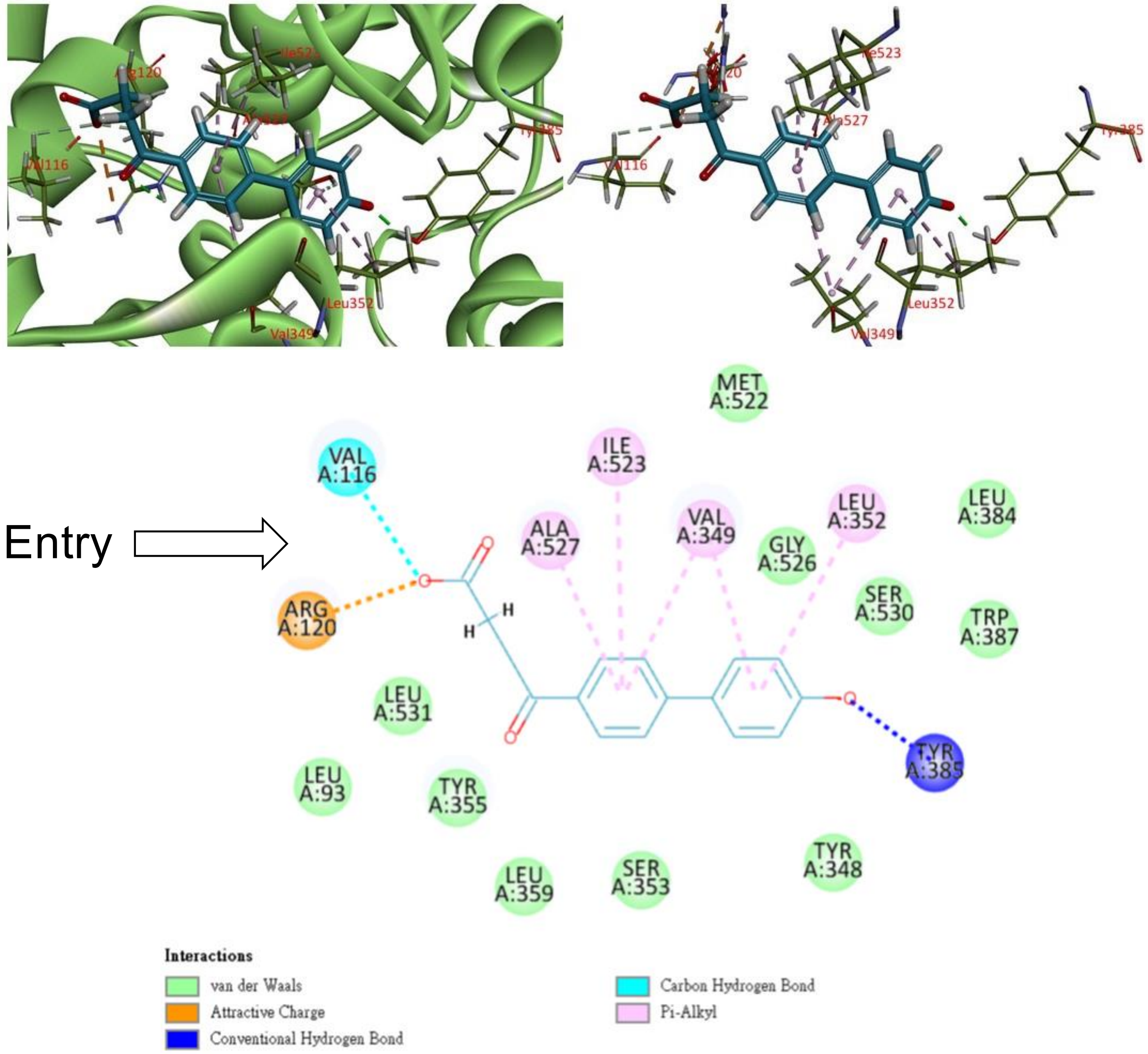

Figure S1. The 3-dimensional and 2-dimensional representations of the interaction of compound **6l** with the active site of COX-1 enzyme (1EQG). The subunits responsible for interacting with the compound **6l** are labeled in color and the interactions are denoted by the dotted lines.

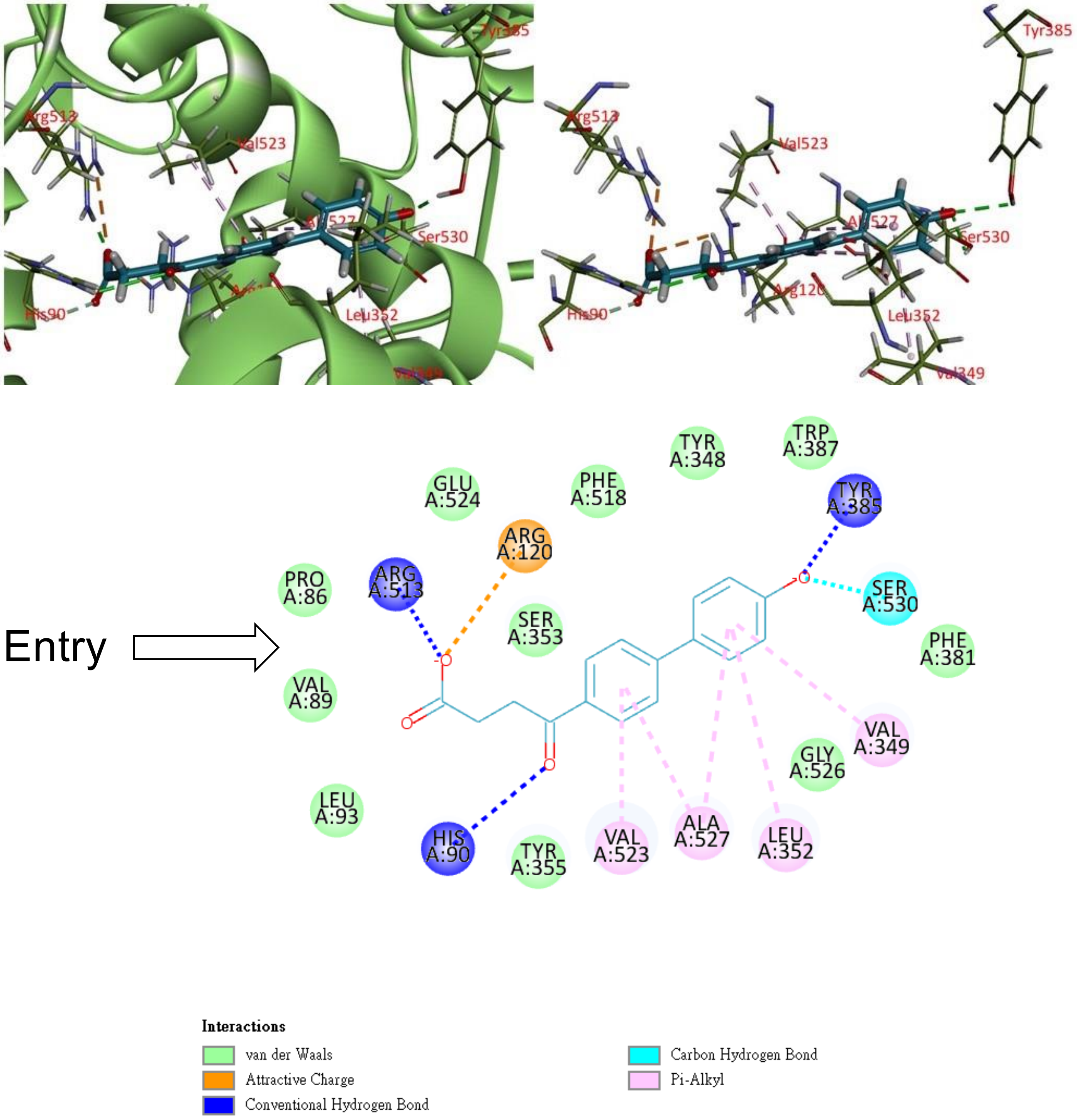

Figure S2. The 3-dimensional and 2-dimensional representations of the interaction of compound **6l** with the active site of COX-2 enzyme (1CX2). The subunits responsible for interacting with the compound **6l** are labeled in color and the interactions are denoted by the dotted lines.

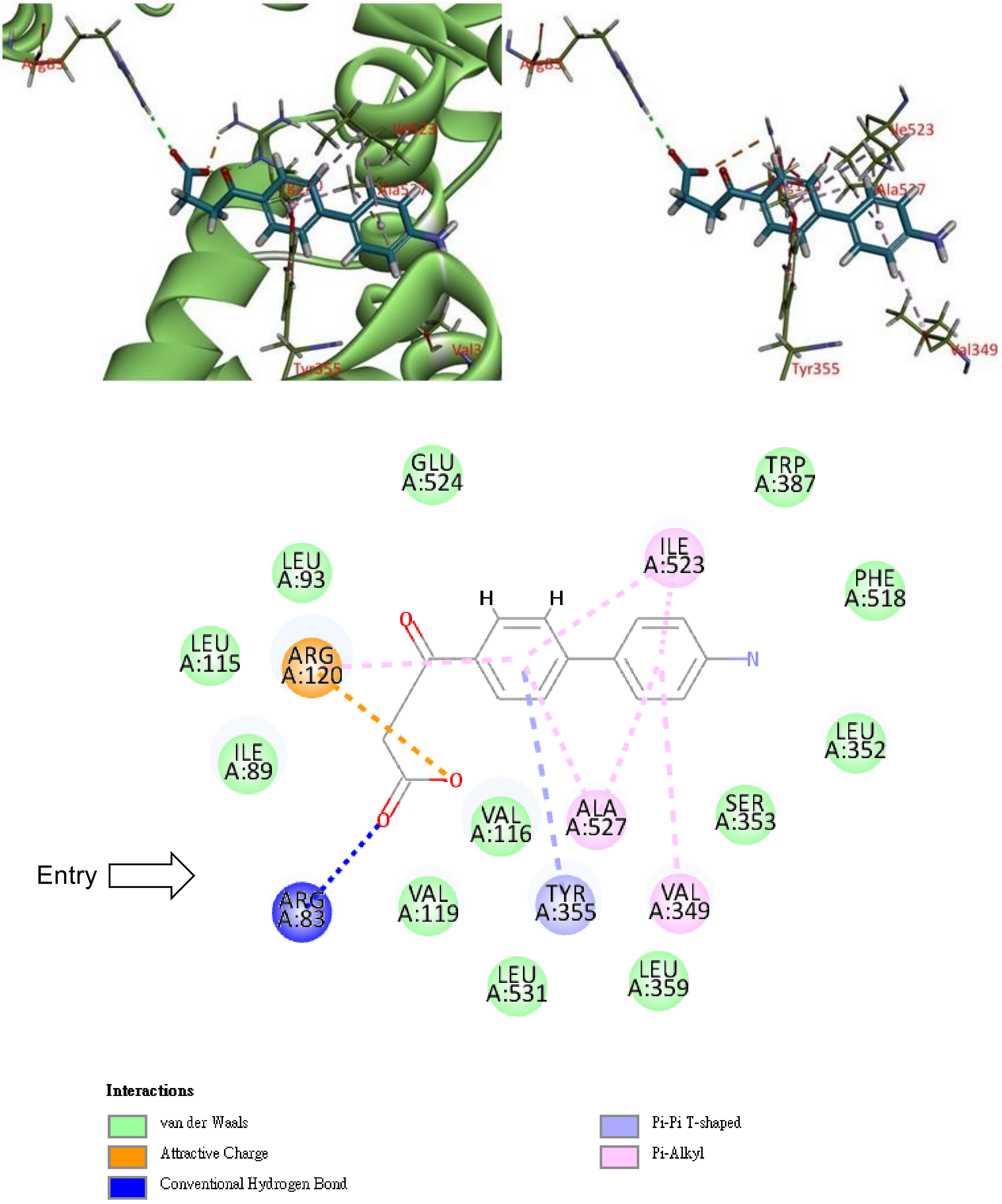

Figure S3. The 3-dimensional and 2-dimensional representations of the interaction of compound **6o** with the active site of COX-1 enzyme (1EQG). The subunits responsible for interacting with the compound **6o** are labeled in color and the interactions are denoted by the dotted lines.

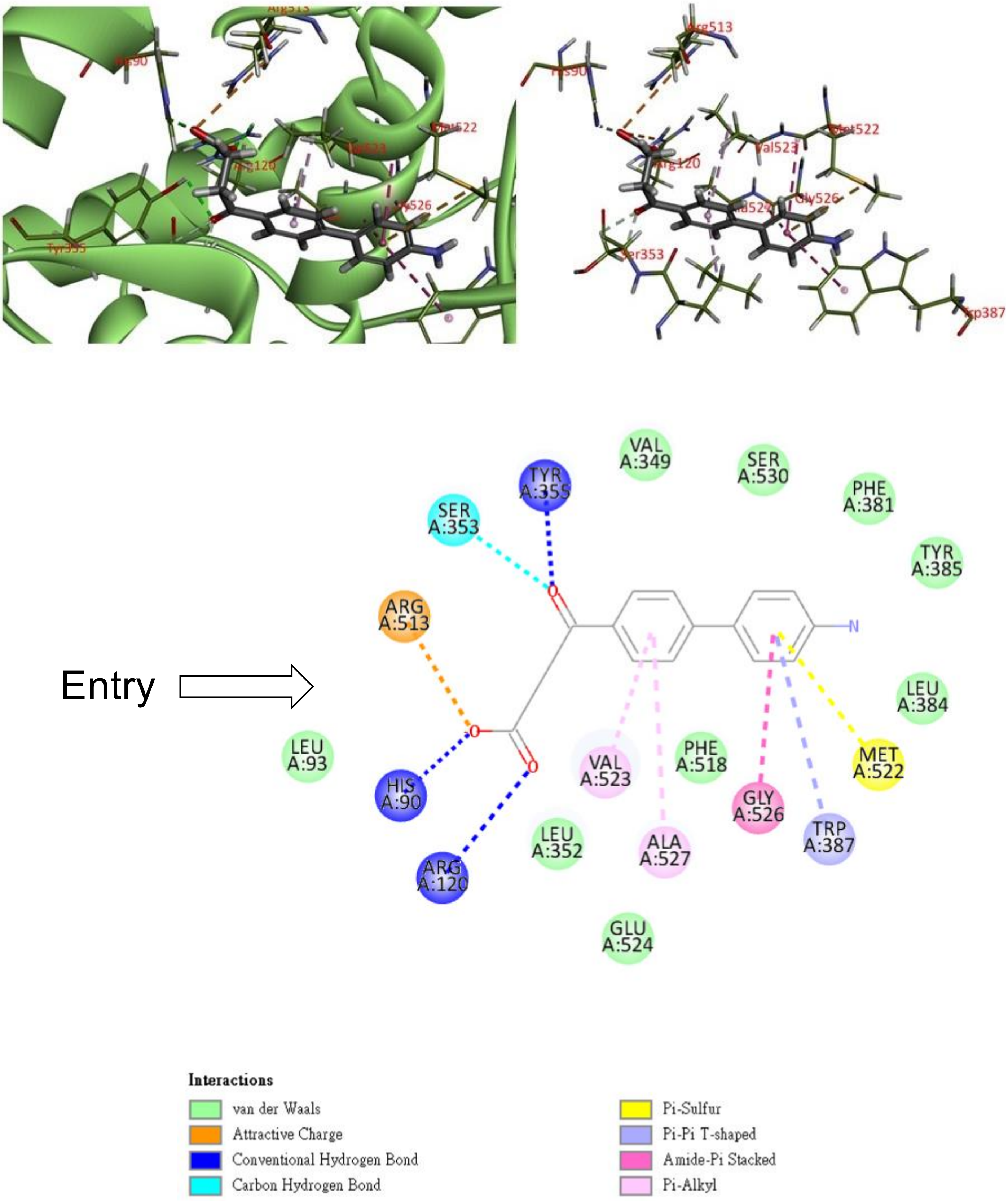

Figure S4. The 3-dimensional and 2-dimensional representations of the interaction of compound **6o** with the active site of COX-2 enzyme (1CX2). The subunits responsible for interacting with the compound **6o** are labeled in color and the interactions are denoted by the dotted lines.

1. Spectra and biological assay data

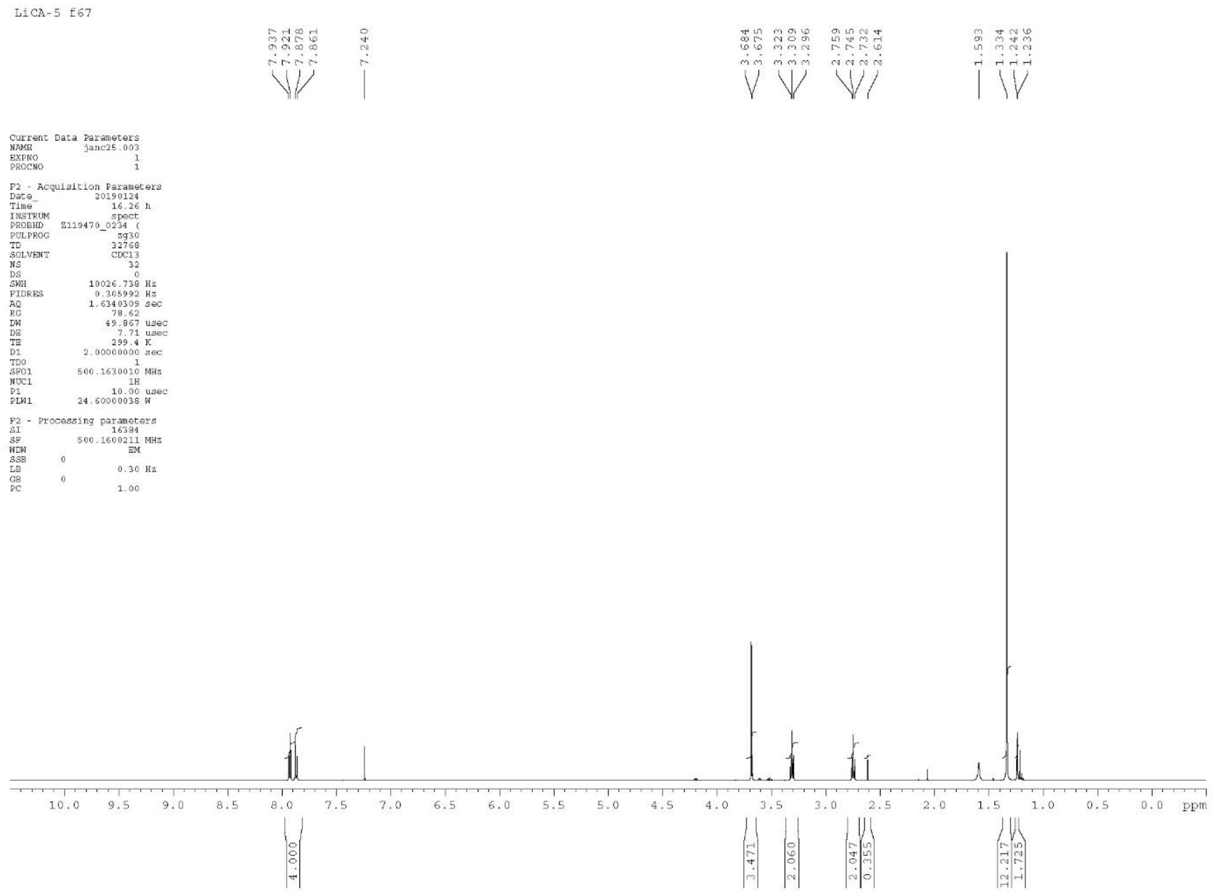

^{1}H NMR

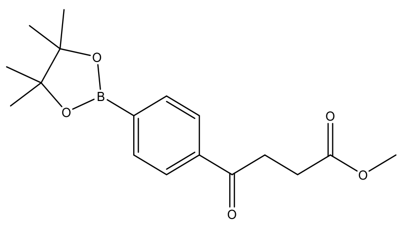

3

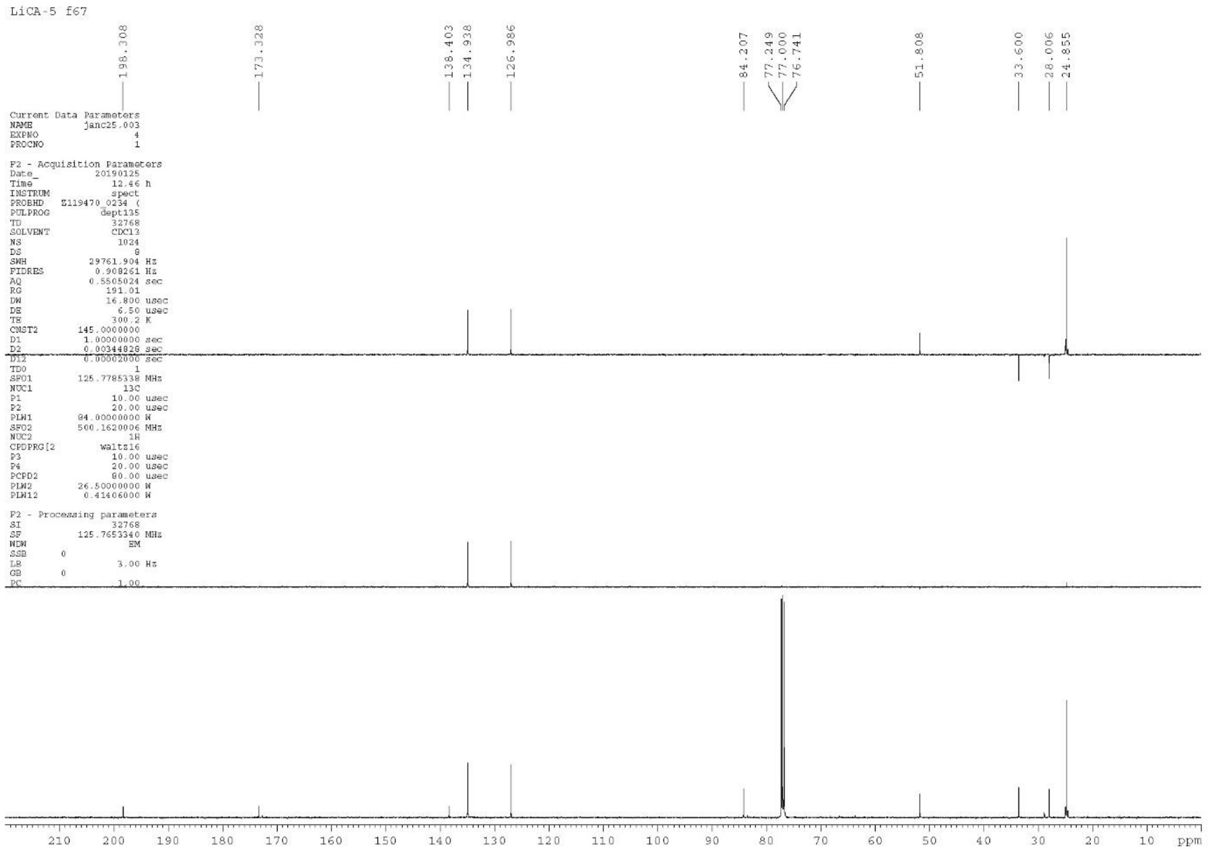

^{13}C-DEPT-135 NMR

3

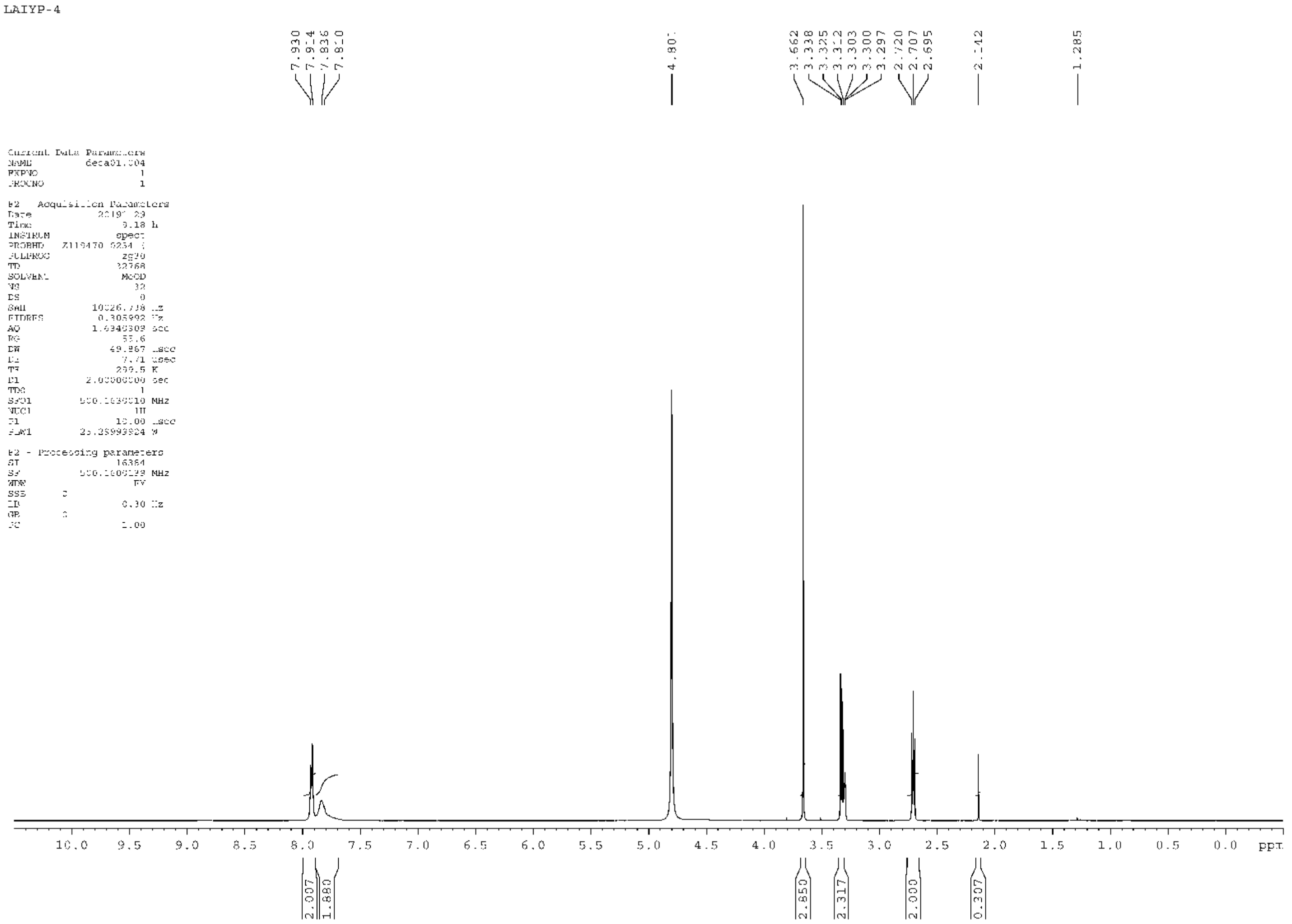

LAIYP-4
Current Data Parameters
F2 Acquisition Parameters
F2 - Processing parameters
ppm

^{1}H NMR

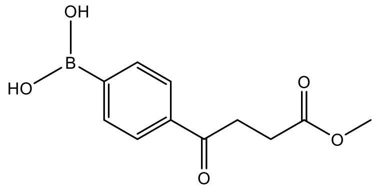

OH
B
HO
O
O
O

4

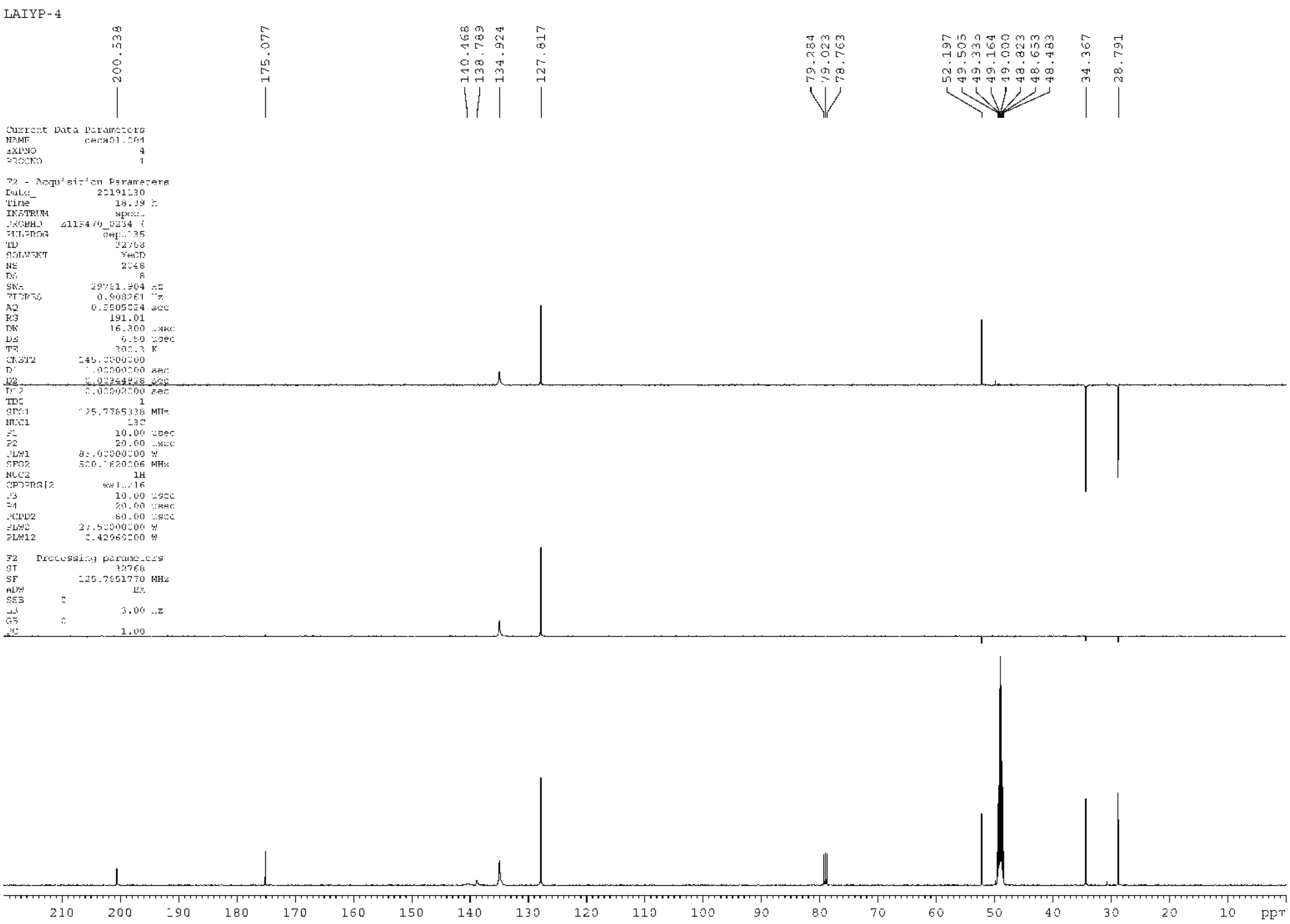

^{13}C-DEPT-135 NMR

4

Display Report

Analysis Info

Analysis Name	D:\Data\NCTU SERVICE\Data\2019\20190326\LAIYP-1_GB7_01_22902.d	Acquisition Date	3/26/2019 12:10:13 PM
Method	Small molecule.m	Operator	NCTU
Sample Name	LAIYP-1	Instrument	impact HD 1819696.00164
Comment			

Acquisition Parameter

Source Type	ESI	Ion Polarity	Positive	Set Nebulizer	1.0 Bar
Focus	Active	Set Capillary	4500 V	Set Dry Heater	200 °C
Scan Begin	50 m/z	Set End Plate Offset	-500 V	Set Dry Gas	6.0 l/min
Scan End	1500 m/z	Set Charging Voltage	2000 V	Set Divert Valve	Waste
		Set Corona	0 nA	Set APCI Heater	0 °C

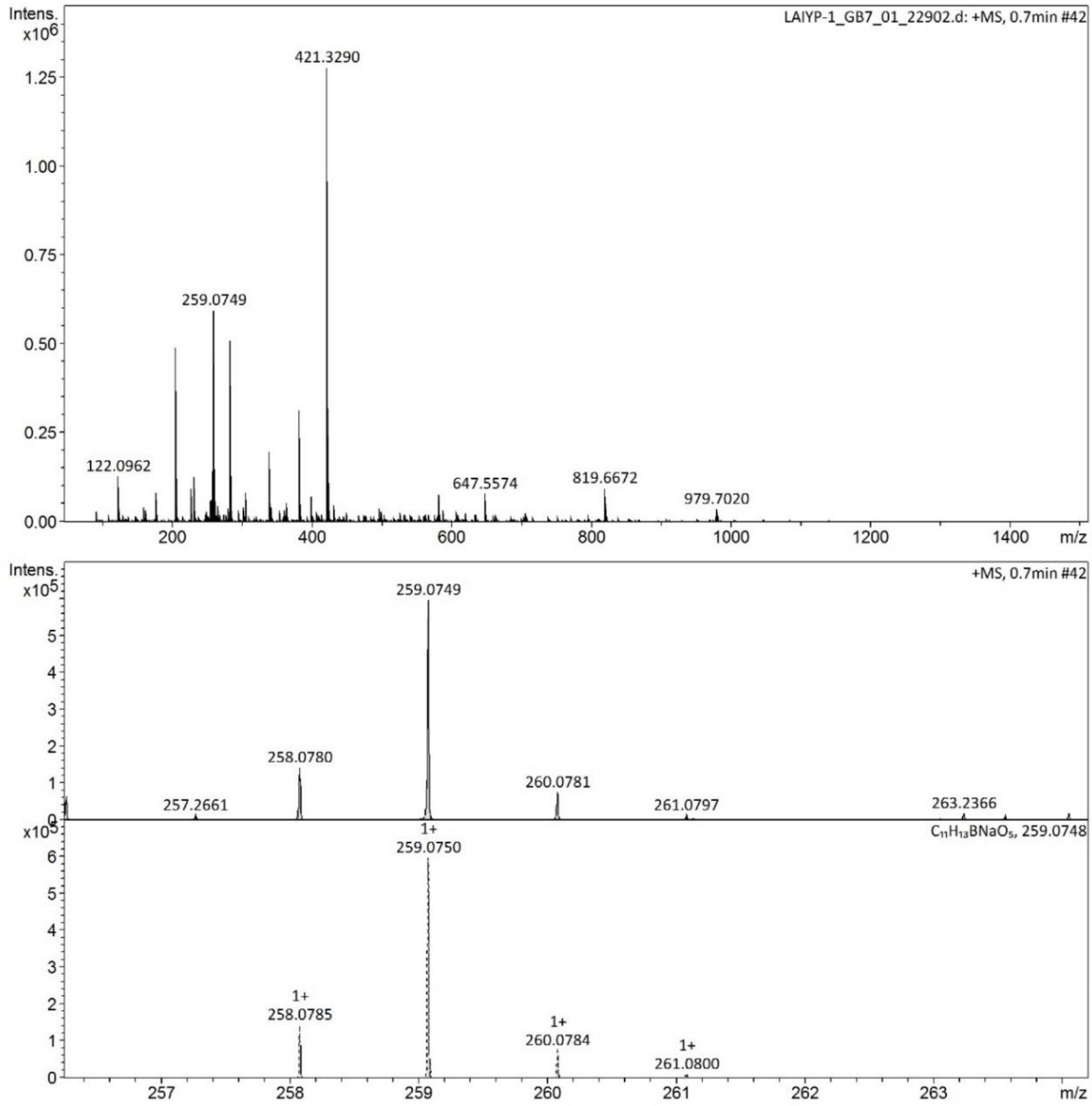

HR ESI-MS

4

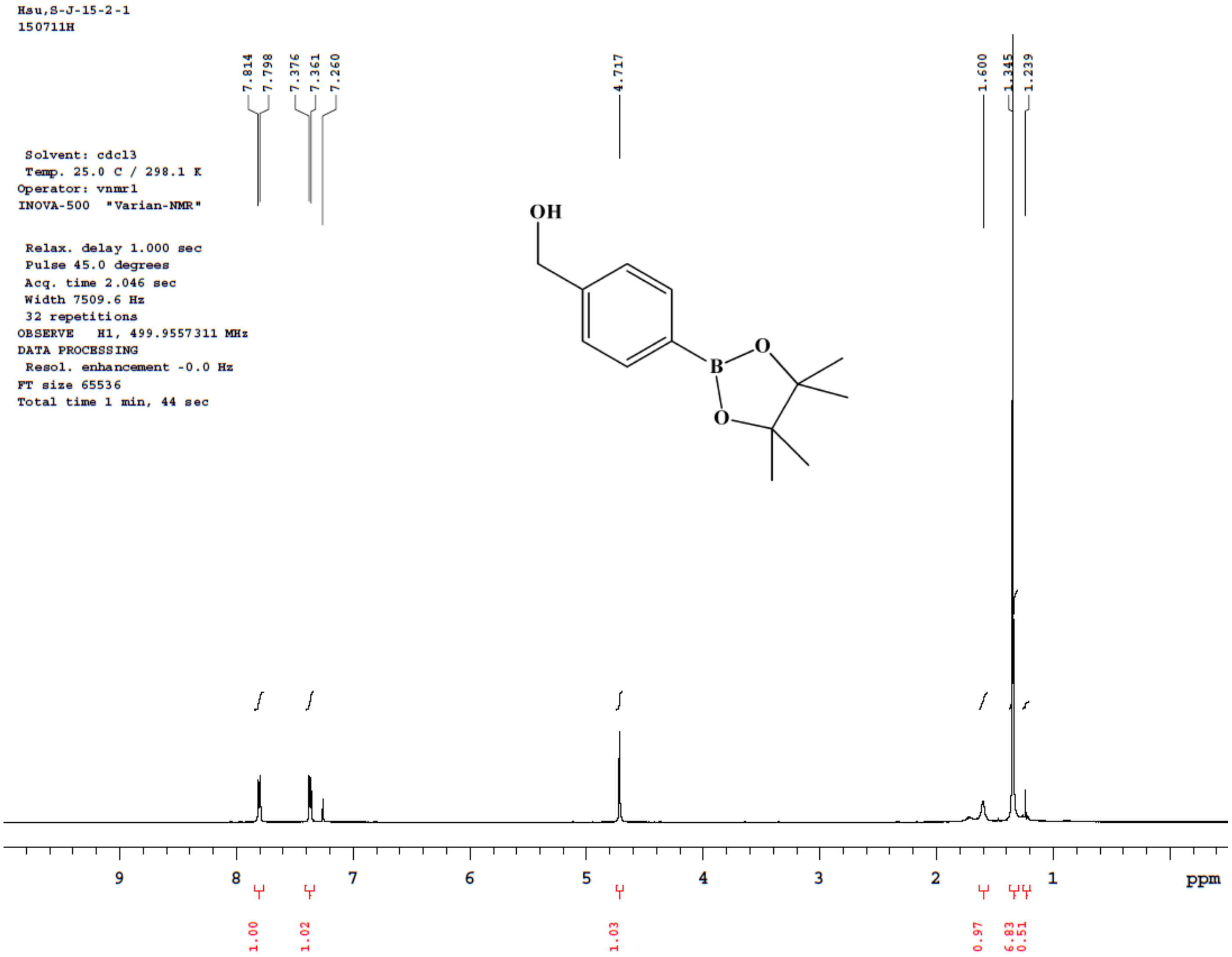

4-boronopinacol phenylmethanol

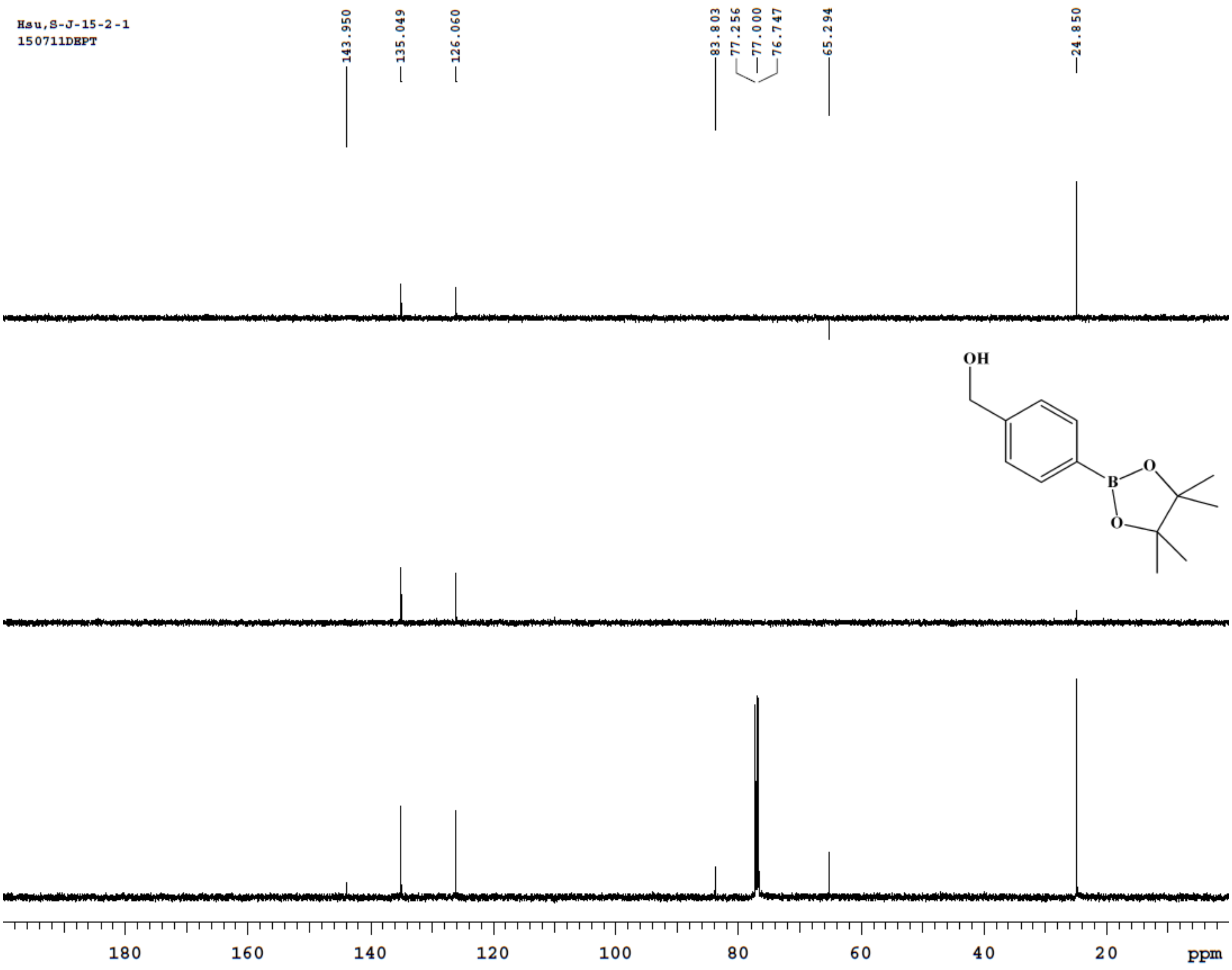

4-boronopinacol phenylmethanol

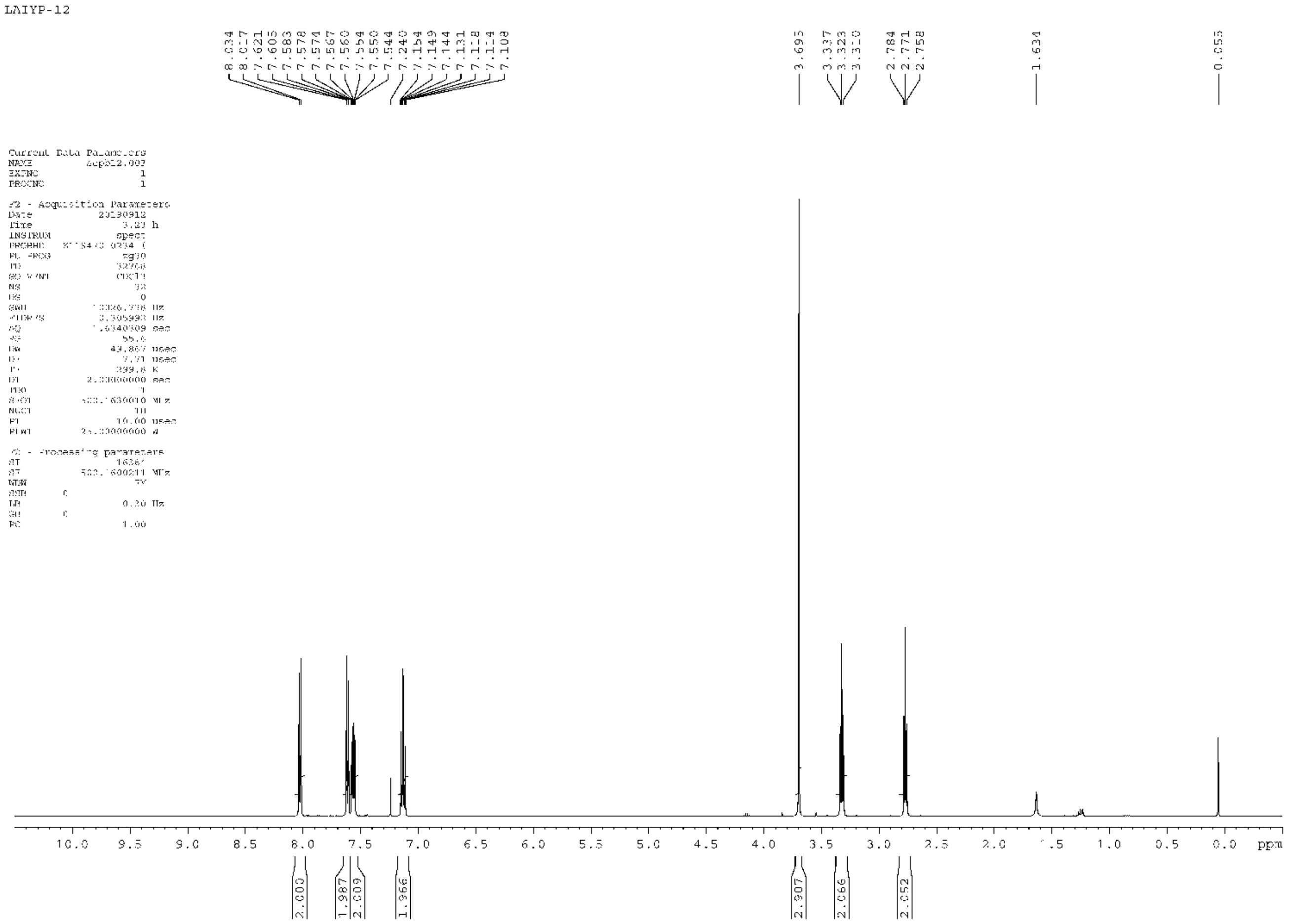
LAIYP-12
8.034
8.017
7.621
7.605
7.583
7.578
7.574
7.567
7.560
7.554
7.550
7.544
7.240
7.154
7.149
7.144
7.131
7.118
7.114
7.108
3.695
3.337
3.323
3.310
2.784
2.771
2.758
1.634
0.055
10.0 9.5 9.0 8.5 8.0 7.5 7.0 6.5 6.0 5.5 5.0 4.5 4.0 3.5 3.0 2.5 2.0 1.5 1.0 0.5 0.0 ppm
2.000
1.987
2.009
1.966
2.907
2.066
2.052

^{1}H NMR

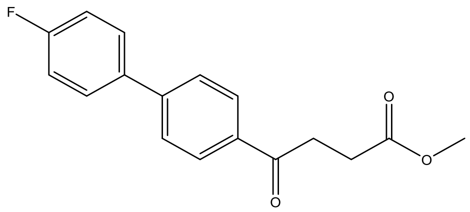
F
O
O
O

5a

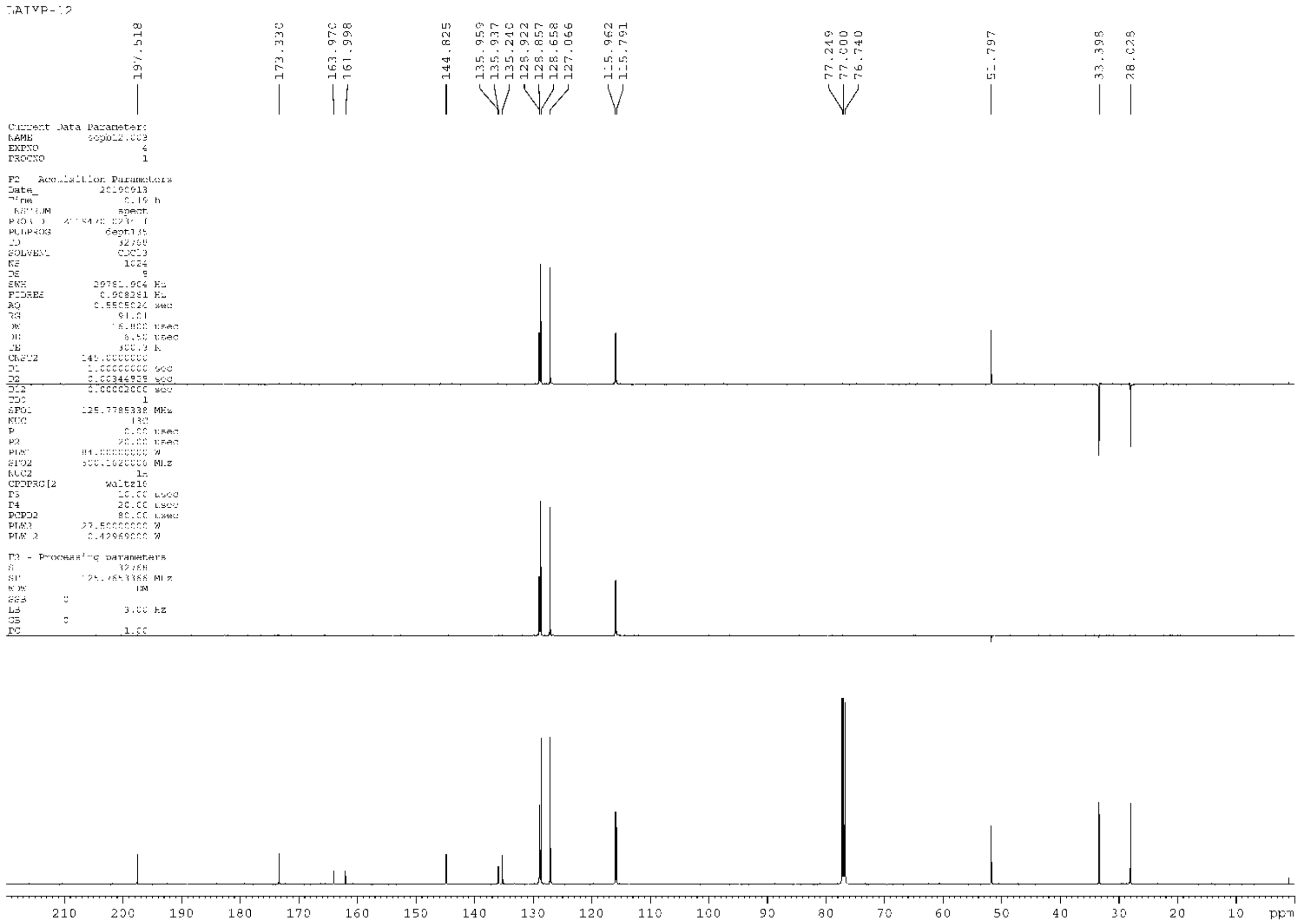

^{13}C-DEPT-135 NMR

5a

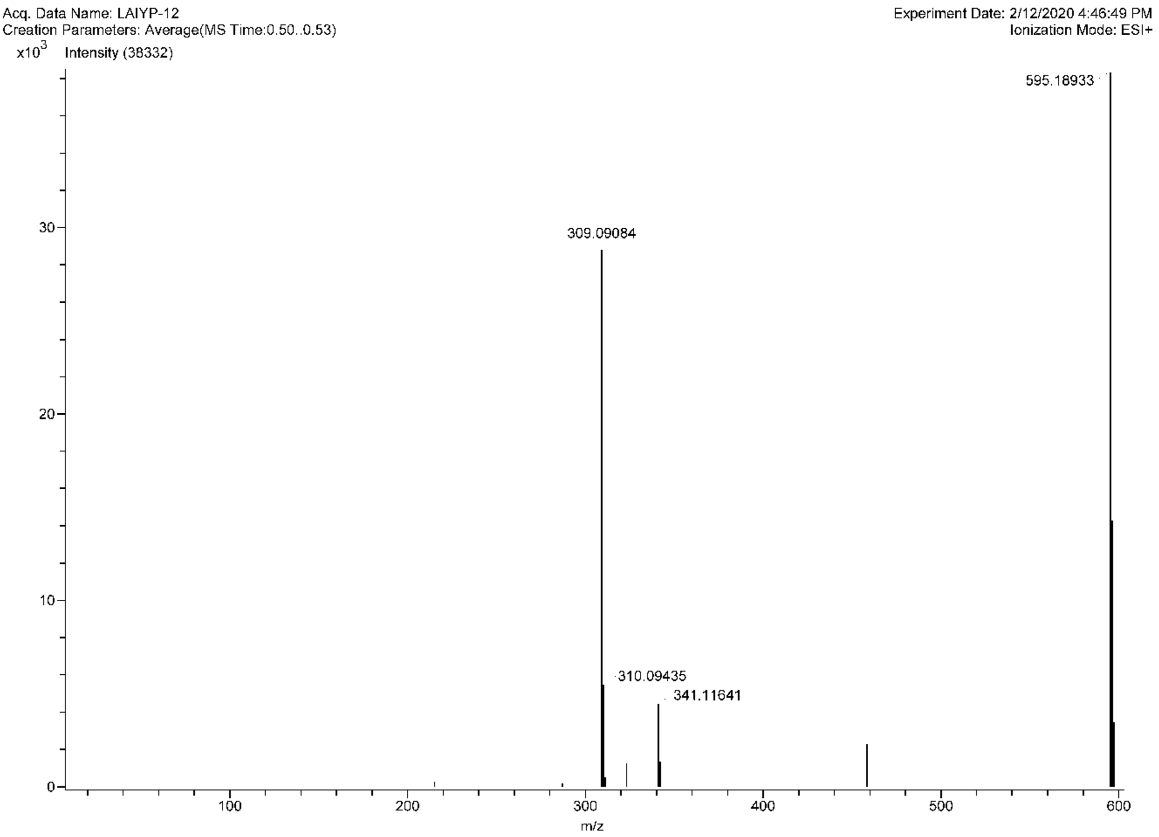

Acq. Data Name: LAIYP-12
Creation Parameters: Average(MS Time:0.50..0.53)
Experiment Date: 2/12/2020 4:46:49 PM
Ionization Mode: ESI+
x10^3 Intensity (38332)
595.18933
309.09084
310.09435
341.11641
0
10
20
30
100
200
300
400
500
600
m/z

HR ESI-MS

5a

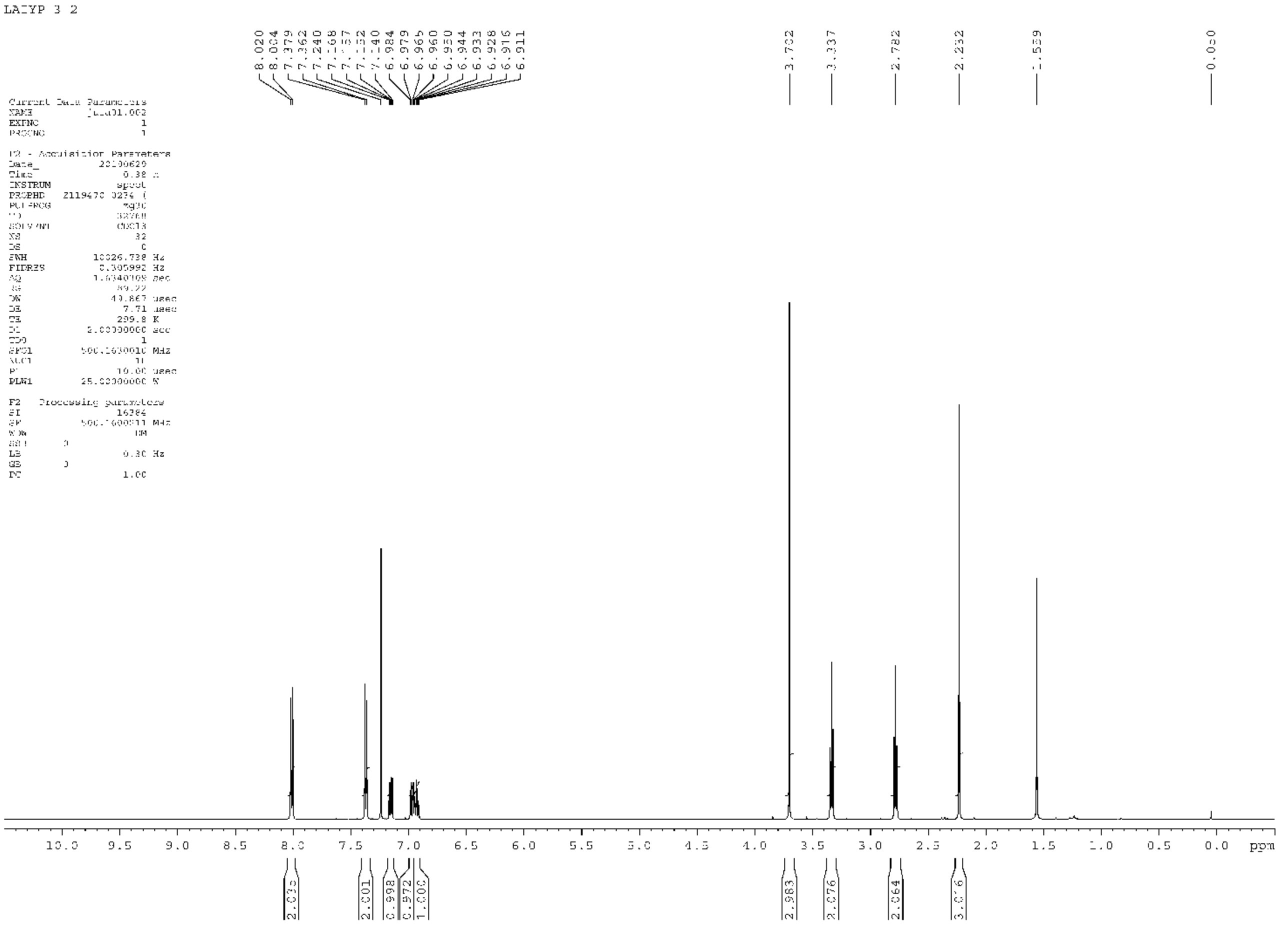

^{1}H NMR

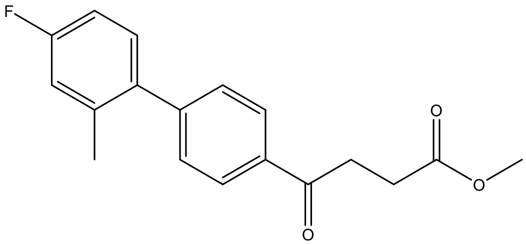

5b

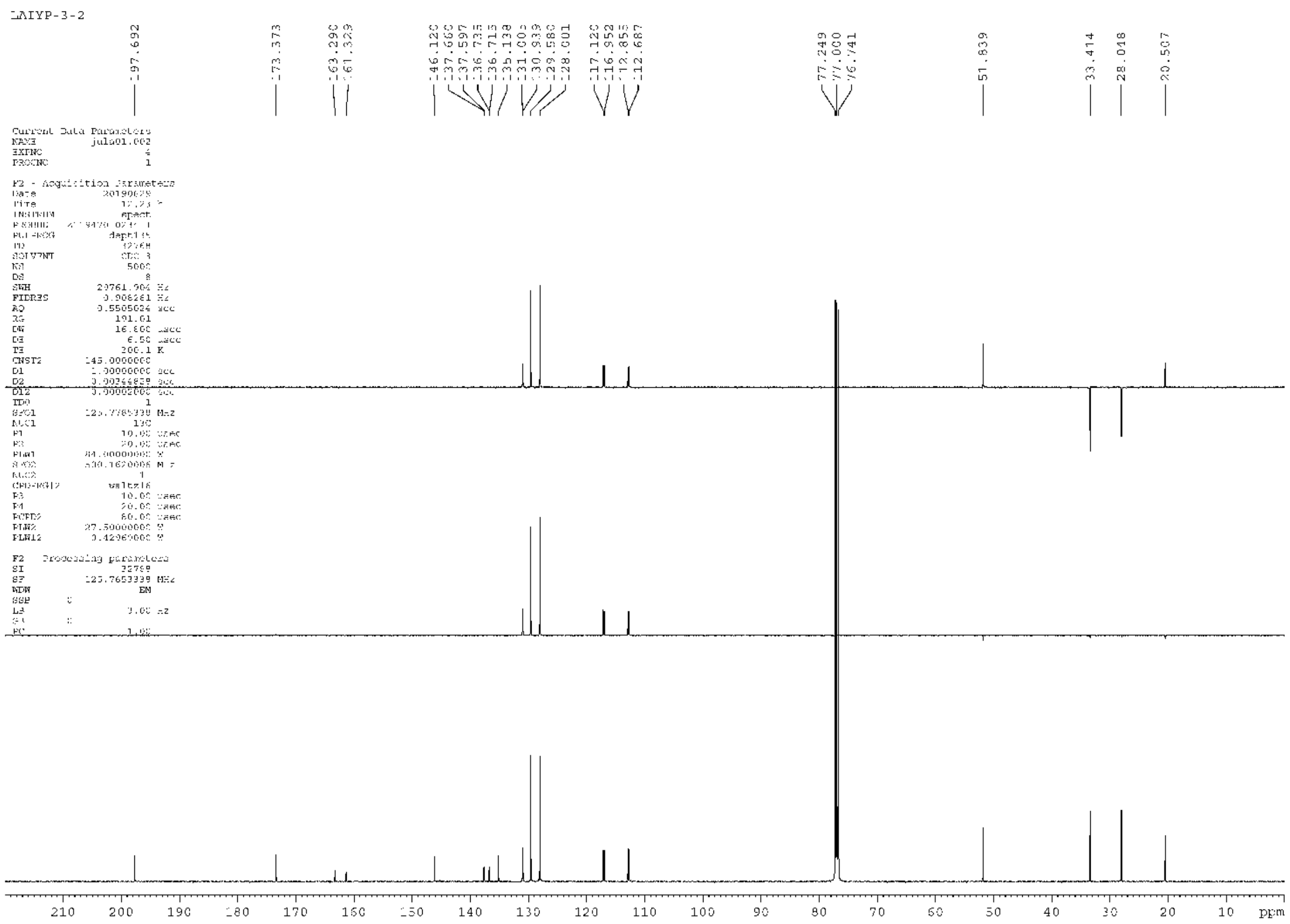

^{13}C-DEPT-135 NMR

5b

Display Report

Analysis Info

Analysis Name	D:\Data\nctu service\data\2019\20190705\LAIYP-3-2_GD5_01_17399.d	Acquisition Date	7/5/2019 4:14:59 PM
Method	Small molecule.m	Operator	NCTU
Sample Name	LAIYP-3-2	Instrument	impact HD 1819696.00164
Comment			

Acquisition Parameter

Source Type	ESI	Ion Polarity	Positive	Set Nebulizer	1.0 Bar
Focus	Active	Set Capillary	4500 V	Set Dry Heater	200 °C
Scan Begin	50 m/z	Set End Plate Offset	-500 V	Set Dry Gas	6.0 l/min
Scan End	1500 m/z	Set Charging Voltage	2000 V	Set Divert Valve	Waste
		Set Corona	0 nA	Set APCI Heater	0 °C

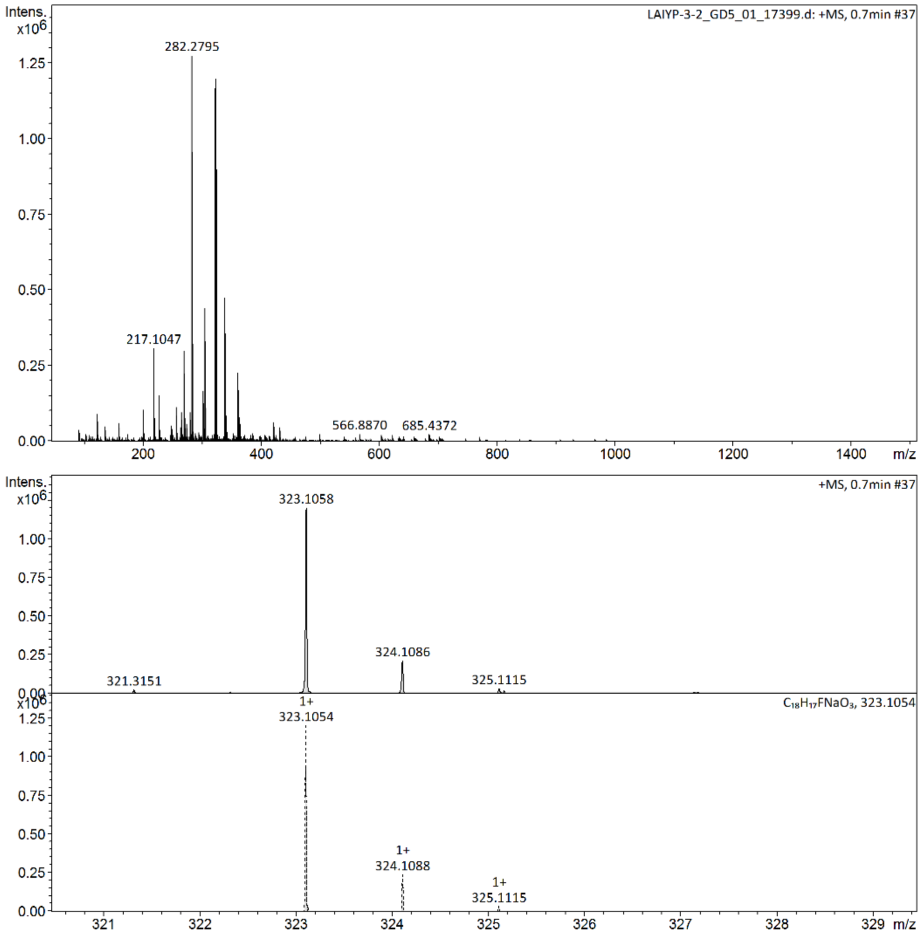

HR ESI-MS

5b

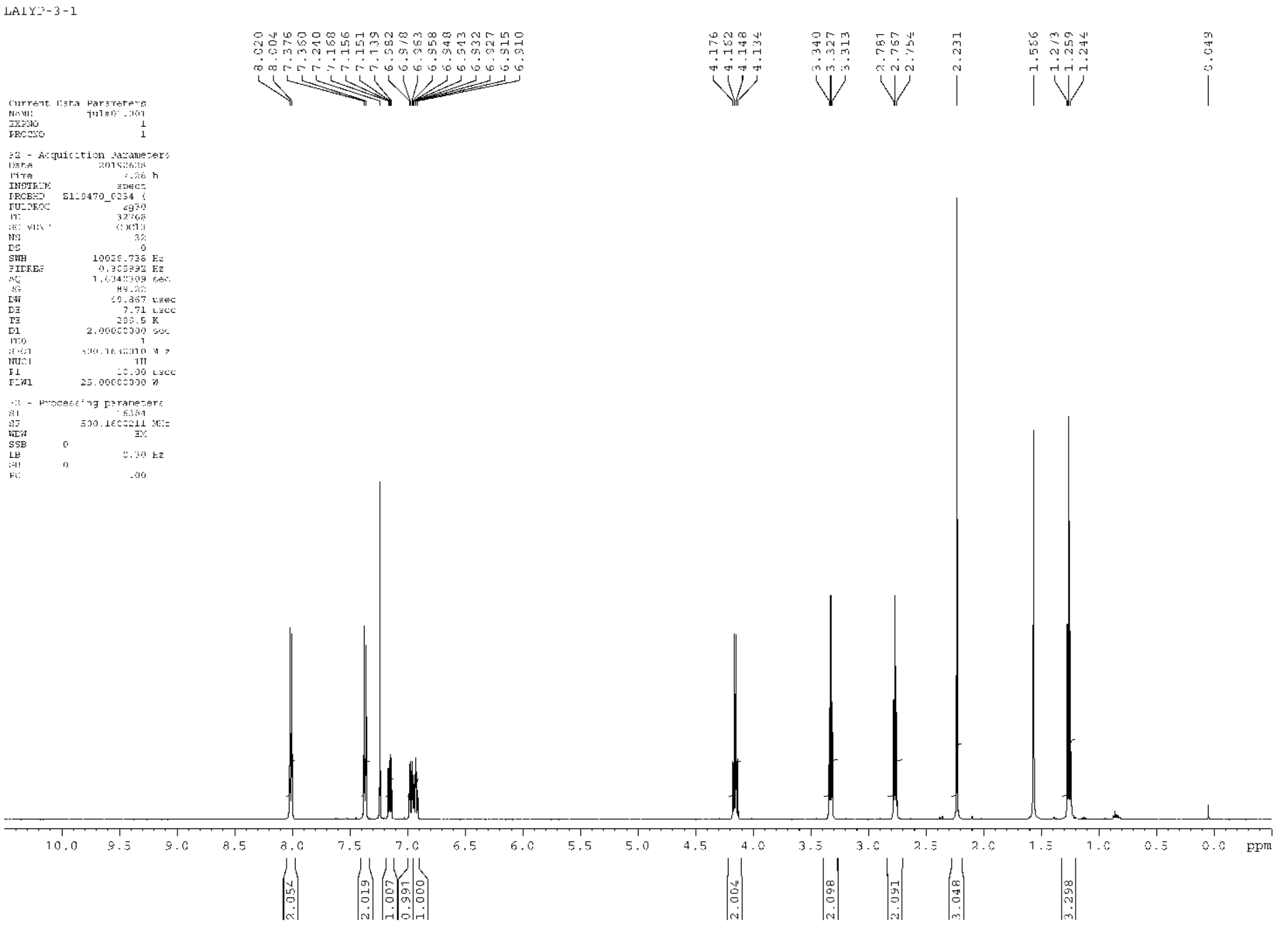
8.020
8.004
7.376
7.360
7.240
7.168
7.156
7.151
7.139
6.982
6.978
6.963
6.958
6.948
6.943
6.932
6.927
6.915
6.910
4.176
4.162
4.148
4.134
3.340
3.327
3.313
2.781
2.767
2.754
2.231
1.566
1.273
1.259
1.244
0.049
ppm
2.054
2.019
1.007
0.991
1.000
2.004
2.098
2.091
3.048
3.298

^{1}H NMR

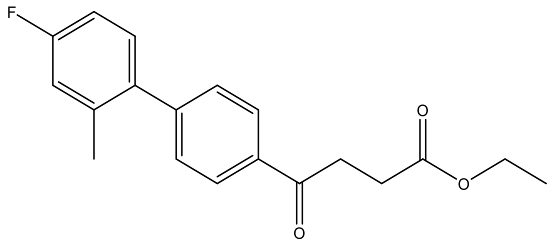
F
O
O
O

5bbyp

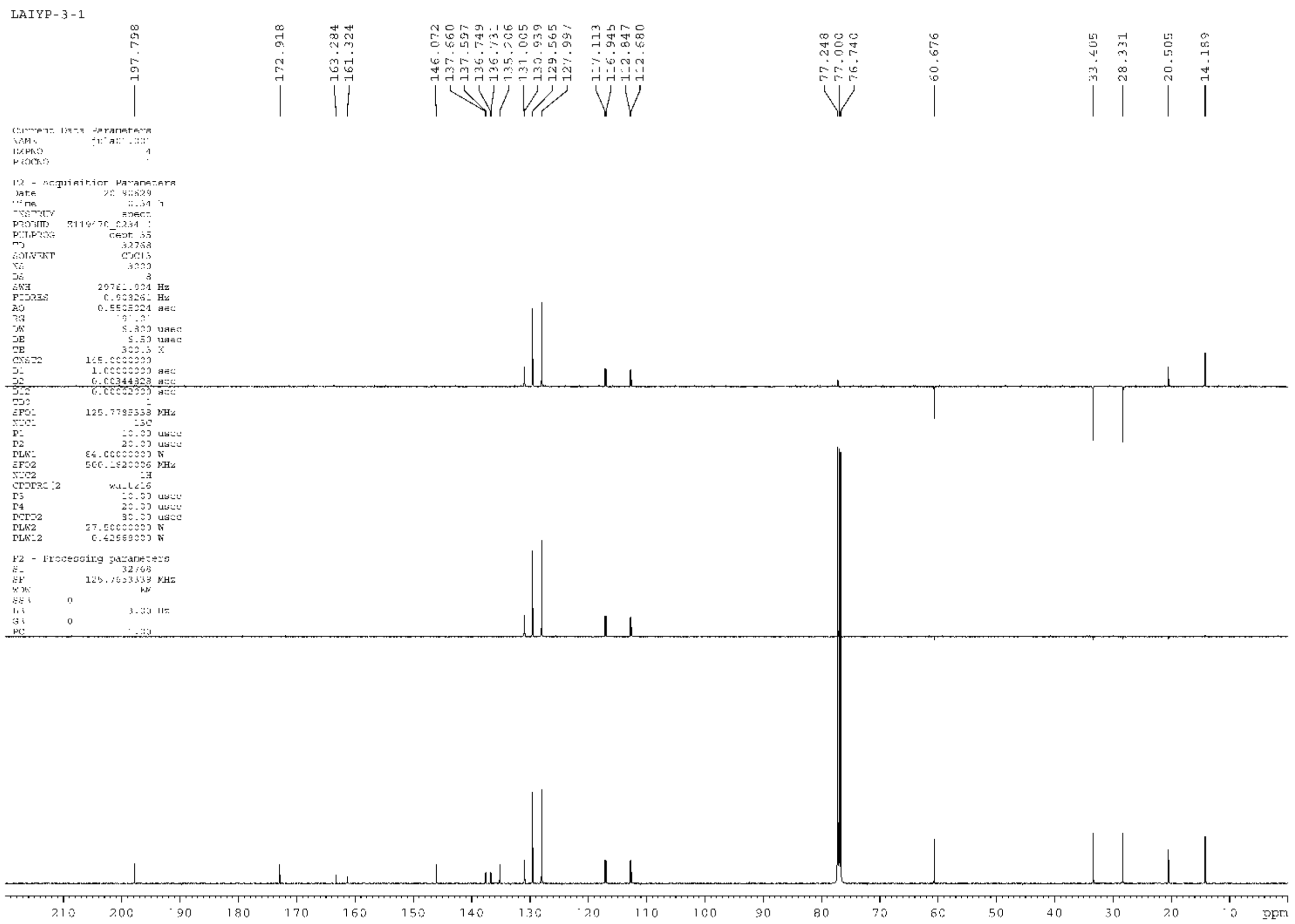

^{13}C-DEPT-135 NMR

5bbyp

Display Report

Analysis Info

Analysis Name	D:\Data\nctu service\data\2019\20190705\LAIYP-3-1_GD6_01_17400.d	Acquisition Date	7/5/2019 4:19:15 PM
Method	Small molecule.m	Operator	NCTU
Sample Name	LAIYP-3-1	Instrument	impact HD 1819696.00164
Comment			

Acquisition Parameter

Source Type	ESI	Ion Polarity	Positive	Set Nebulizer	1.0 Bar
Focus	Active	Set Capillary	4500 V	Set Dry Heater	200 °C
Scan Begin	50 m/z	Set End Plate Offset	-500 V	Set Dry Gas	6.0 l/min
Scan End	1500 m/z	Set Charging Voltage	2000 V	Set Divert Valve	Waste
		Set Corona	0 nA	Set APCI Heater	0 °C

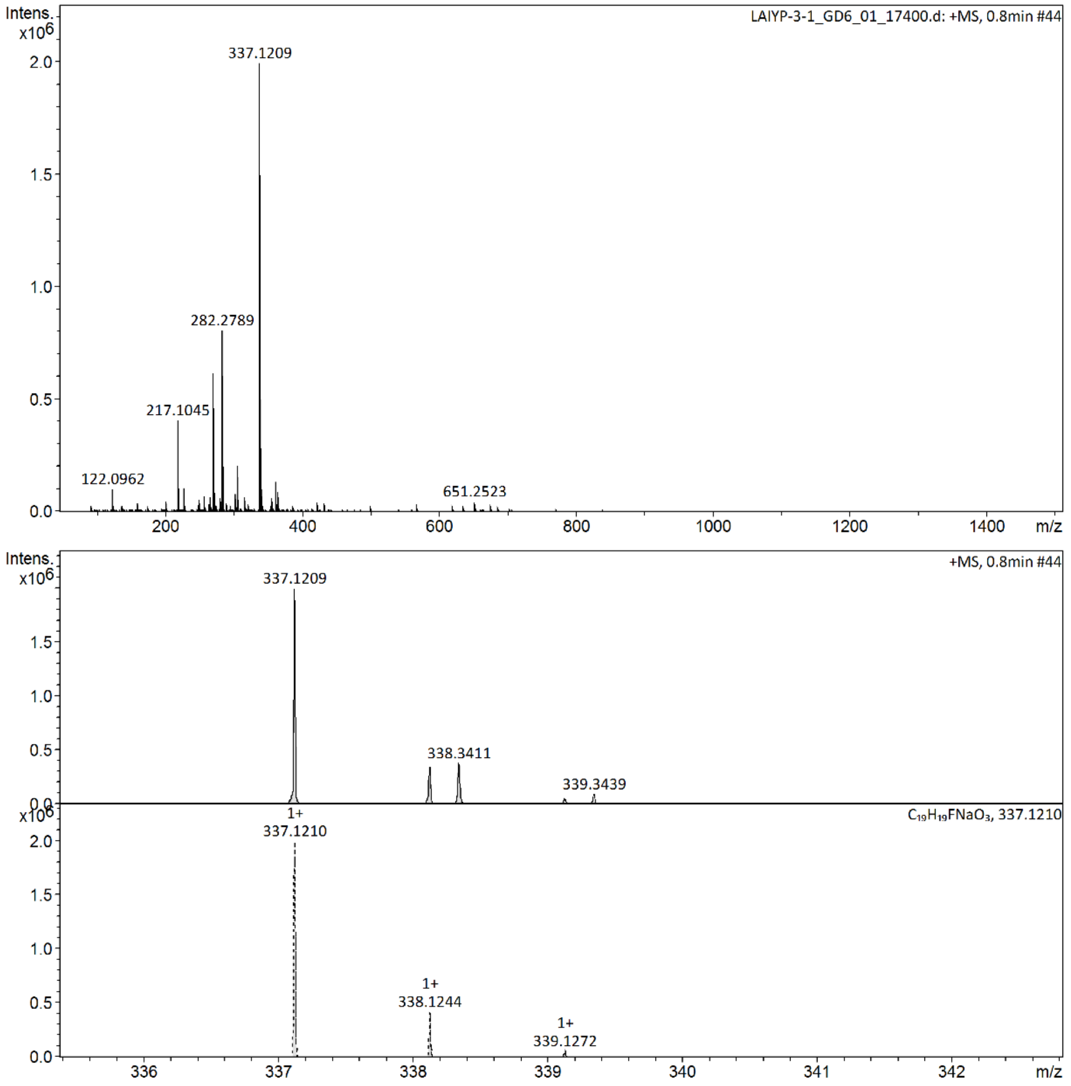

HR ESI-MS

5bbyp

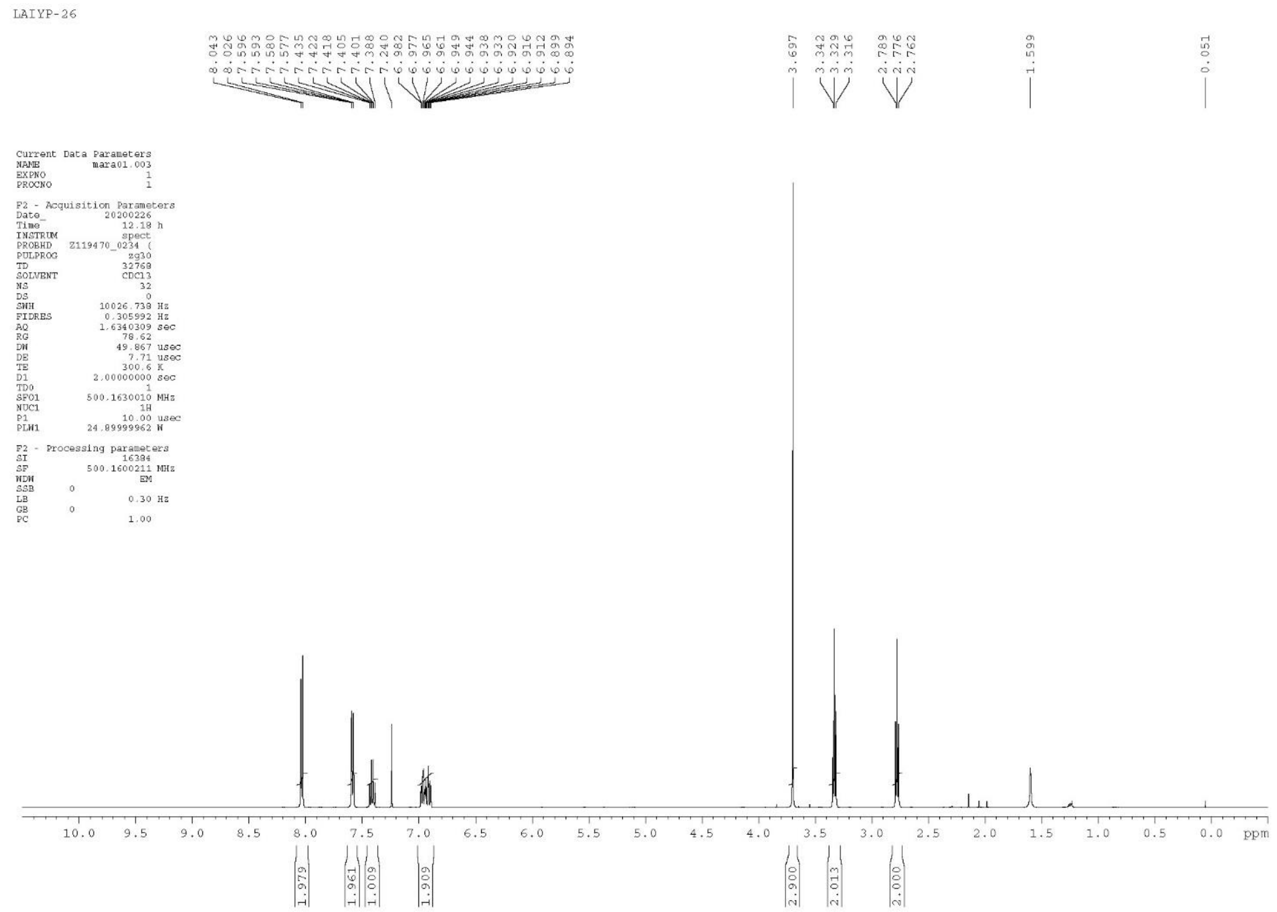
LAIYP-26
8.043
8.026
7.596
7.593
7.580
7.577
7.435
7.422
7.418
7.405
7.401
7.388
7.240
6.982
6.977
6.965
6.961
6.949
6.944
6.938
6.933
6.920
6.916
6.912
6.899
6.894
3.697
3.342
3.329
3.316
2.789
2.776
2.762
1.599
0.051
Current Data Parameters
NAME mara01.003
EXPNO 1
PROCNO 1
F2 - Acquisition Parameters
Date_ 20200226
Time 12.18 h
INSTRUM spect
PROBHD Z119470_0234 (
PULPROG zg30
TD 32768
SOLVENT CDCl3
NS 32
DS 0
SWH 10026.738 Hz
FIDRES 0.305992 Hz
AQ 1.6340309 sec
RG 78.62
DW 49.867 usec
DE 7.71 usec
TE 300.6 K
D1 2.00000000 sec
TD0 1
SFO1 500.1630010 MHz
NUC1 1H
P1 10.00 usec
PLW1 24.89999962 W
F2 - Processing parameters
SI 16384
SF 500.1600211 MHz
WDW EM
SSB 0
LB 0.30 Hz
GB 0
PC 1.00
10.0 9.5 9.0 8.5 8.0 7.5 7.0 6.5 6.0 5.5 5.0 4.5 4.0 3.5 3.0 2.5 2.0 1.5 1.0 0.5 0.0 ppm
1.979
1.961
1.009
1.909
2.900
2.013
2.000

^{1}H NMR

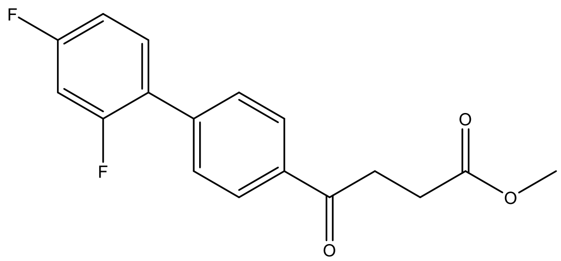
F
F
O
O
O

5c

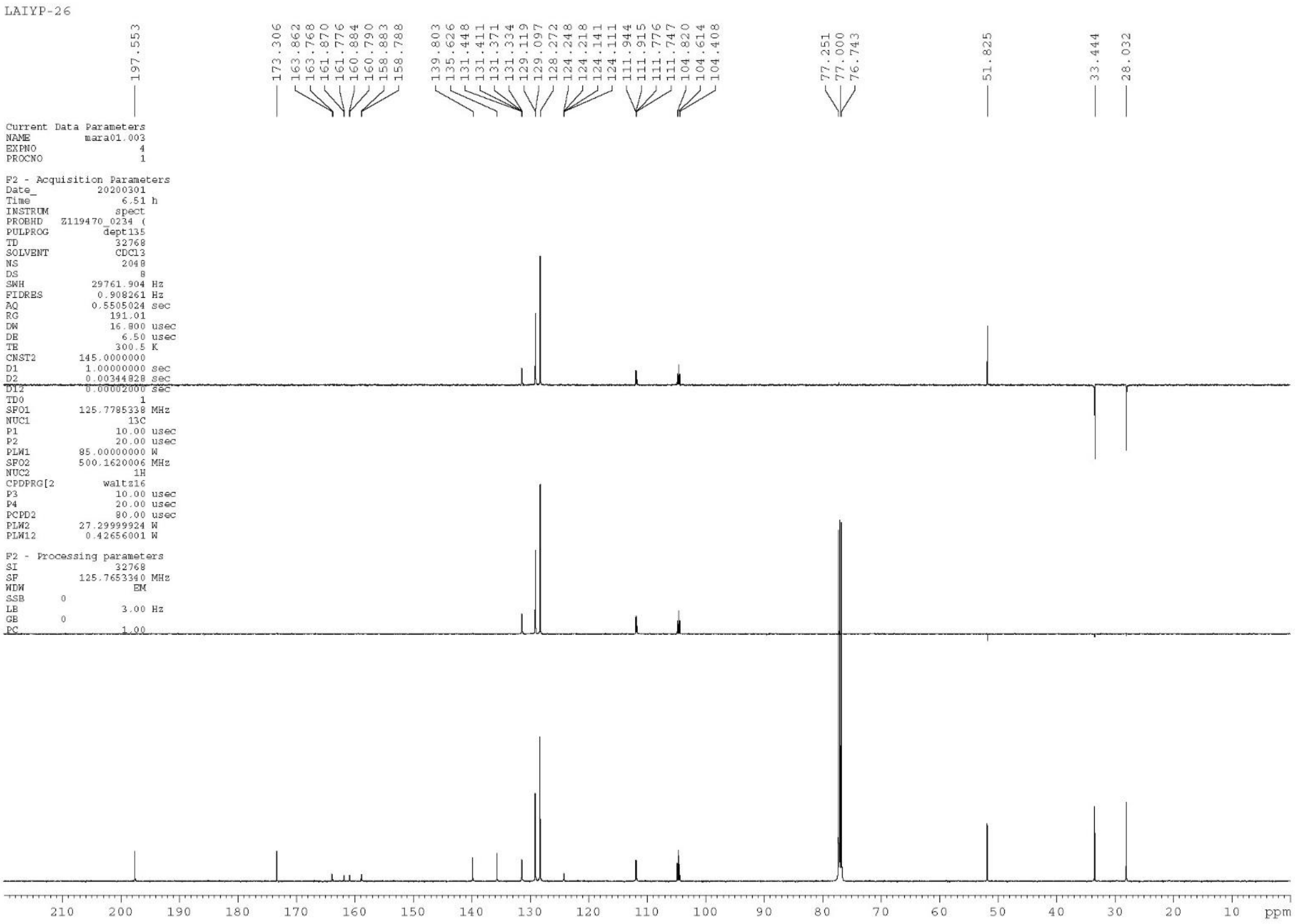

^{13}C-DEPT-135 NMR

5c

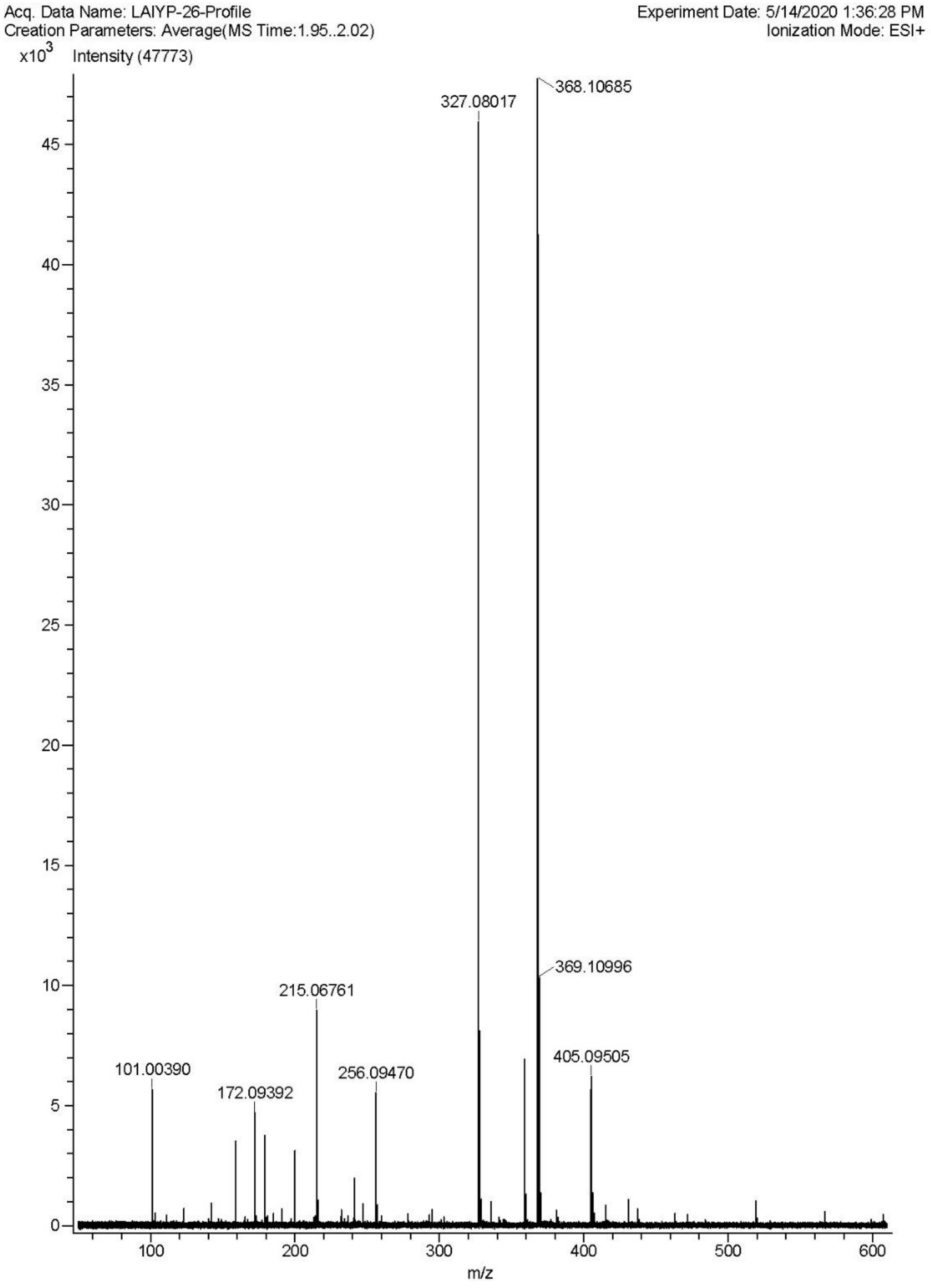

Acq. Data Name: LAIYP-26-Profile
Creation Parameters: Average(MS Time:1.95..2.02)
Experiment Date: 5/14/2020 1:36:28 PM
Ionization Mode: ESI+
x10^3 Intensity (47773)
368.10685
327.08017
369.10996
215.06761
405.09505
101.00390
256.09470
172.09392
m/z

HR ESI-MS

5c

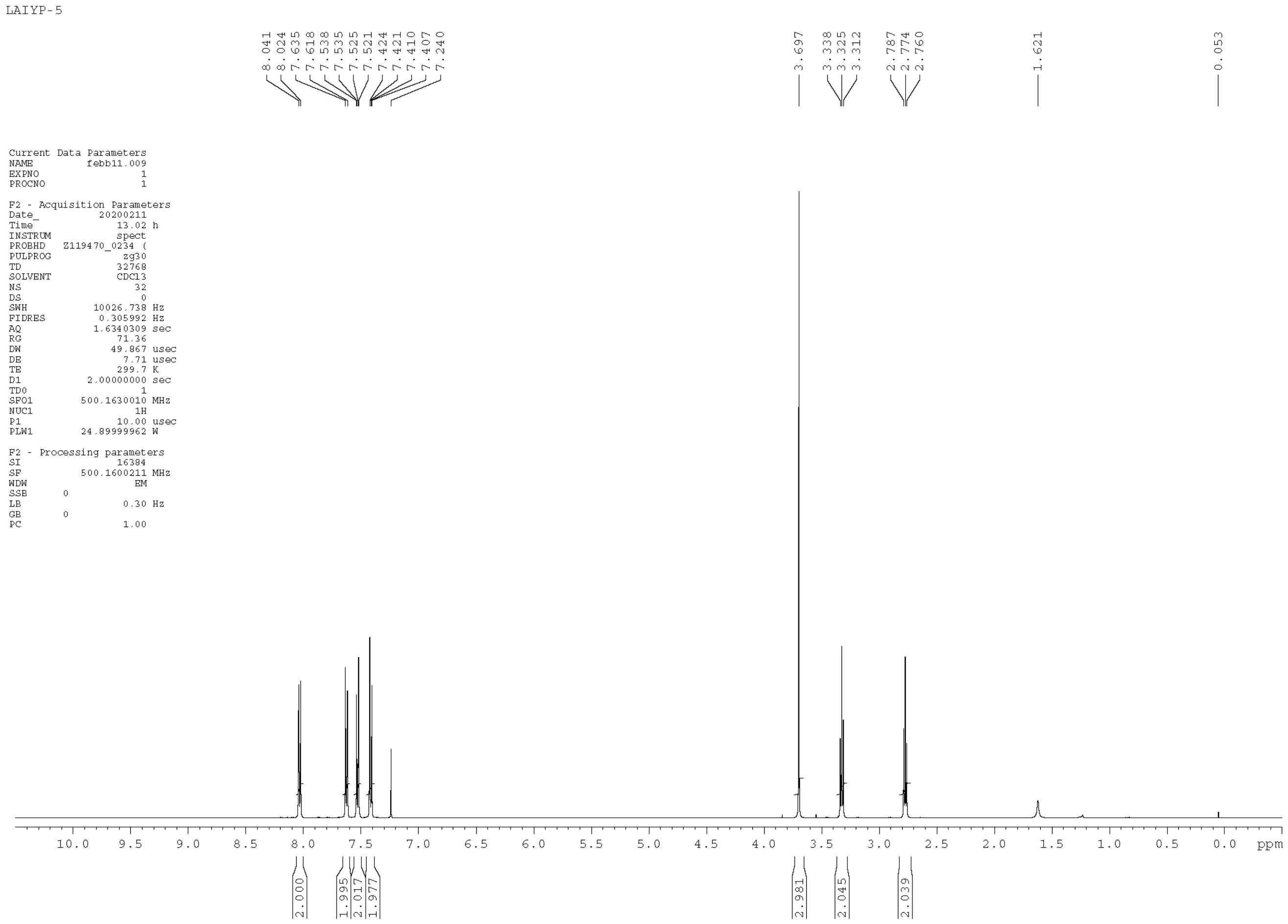
LAIYP-5
8.041
8.024
7.635
7.618
7.538
7.535
7.525
7.521
7.424
7.421
7.410
7.407
7.240
3.697
3.338
3.325
3.312
2.787
2.774
2.760
1.621
0.053
Current Data Parameters
NAME febb11.009
EXPNO 1
PROCNO 1
F2 - Acquisition Parameters
Date_ 20200211
Time 13.02 h
INSTRUM spect
PROBHD Z119470_0234 (
PULPROG zg30
TD 32768
SOLVENT CDCl3
NS 32
DS 0
SWH 10026.738 Hz
FIDRES 0.305992 Hz
AQ 1.6340309 sec
RG 71.36
DW 49.867 usec
DE 7.71 usec
TE 299.7 K
D1 2.00000000 sec
TD0 1
SFO1 500.1630010 MHz
NUC1 1H
P1 10.00 usec
PLW1 24.89999962 W
F2 - Processing parameters
SI 16384
SF 500.1600211 MHz
WDW EM
SSB 0
LB 0.30 Hz
GB 0
PC 1.00
10.0 9.5 9.0 8.5 8.0 7.5 7.0 6.5 6.0 5.5 5.0 4.5 4.0 3.5 3.0 2.5 2.0 1.5 1.0 0.5 0.0 ppm
2.000
1.995
2.017
1.977
2.981
2.045
2.039

^{1}H NMR

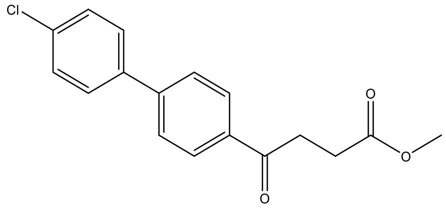
Cl
O
O
O

5d

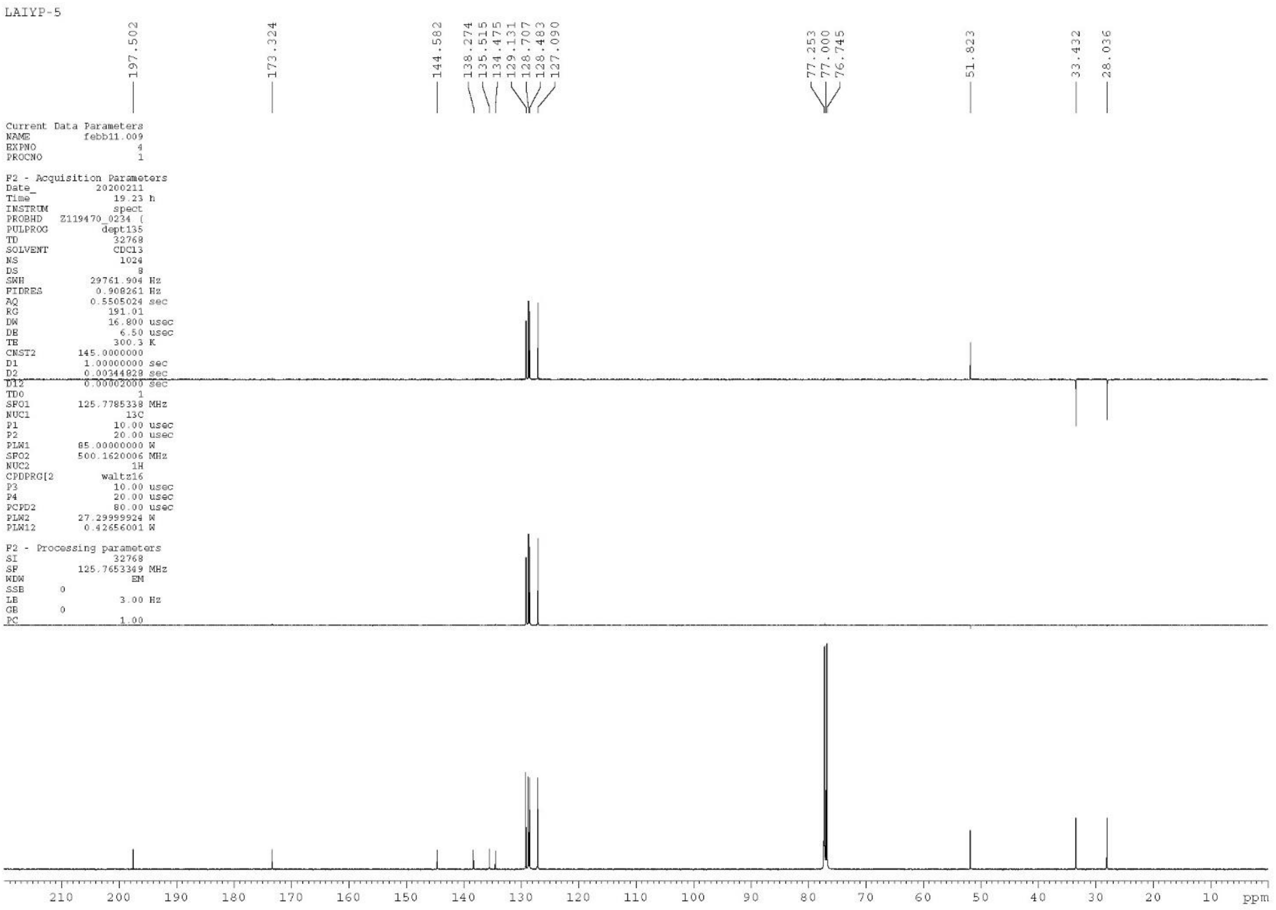

^{13}C-DEPT-135 NMR

5d

Display Report

Analysis Info

Analysis Name	D:\Data\nctu service\data\2019\20190621\LAIYP-2_GA2_01_17203.d	Acquisition Date	6/21/2019 1:19:01 PM
Method	Small molecule.m	Operator	NCTU
Sample Name	LAIYP-2	Instrument	impact HD 1819696.00164
Comment			

Acquisition Parameter

Source Type	ESI	Ion Polarity	Positive	Set Nebulizer	1.0 Bar
Focus	Active	Set Capillary	4500 V	Set Dry Heater	200 °C
Scan Begin	50 m/z	Set End Plate Offset	-500 V	Set Dry Gas	6.0 l/min
Scan End	1500 m/z	Set Charging Voltage	2000 V	Set Divert Valve	Waste
		Set Corona	0 nA	Set APCI Heater	0 °C

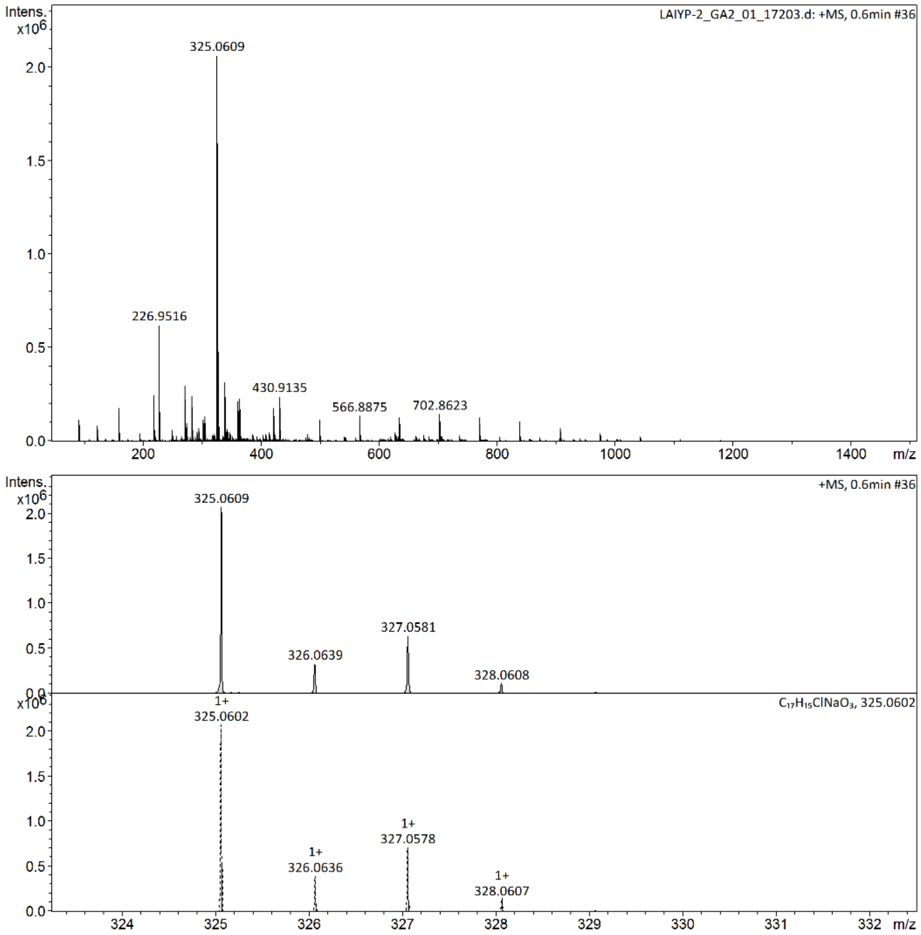

HR ESI-MS

5d

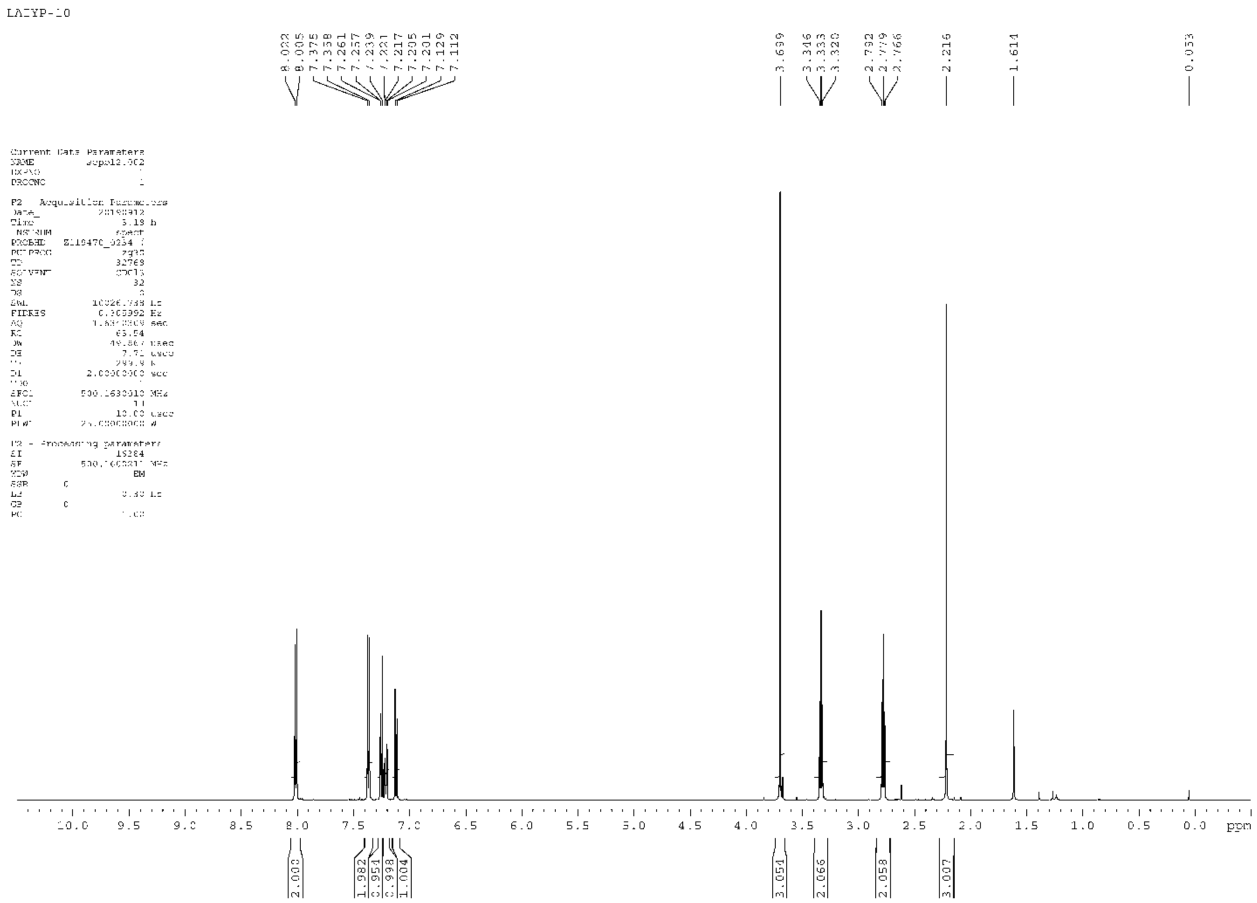

LAIYP-10
8.022
8.005
7.375
7.358
7.261
7.257
7.239
7.221
7.217
7.205
7.201
7.129
7.112
3.699
3.346
3.333
3.320
2.792
2.779
2.766
2.216
1.614
0.053
Current Data Parameters
F2 - Acquisition Parameters
F2 - Processing parameters
10.0 9.5 9.0 8.5 8.0 7.5 7.0 6.5 6.0 5.5 5.0 4.5 4.0 3.5 3.0 2.5 2.0 1.5 1.0 0.5 0.0 ppm
2.000
1.982
1.004
3.054
2.066
2.058
3.007

^{1}H NMR

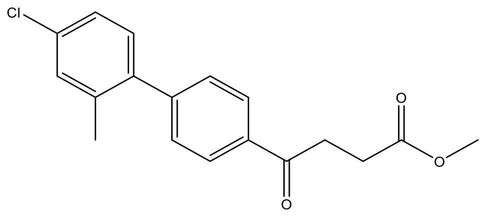

Cl
O
O
O

5e

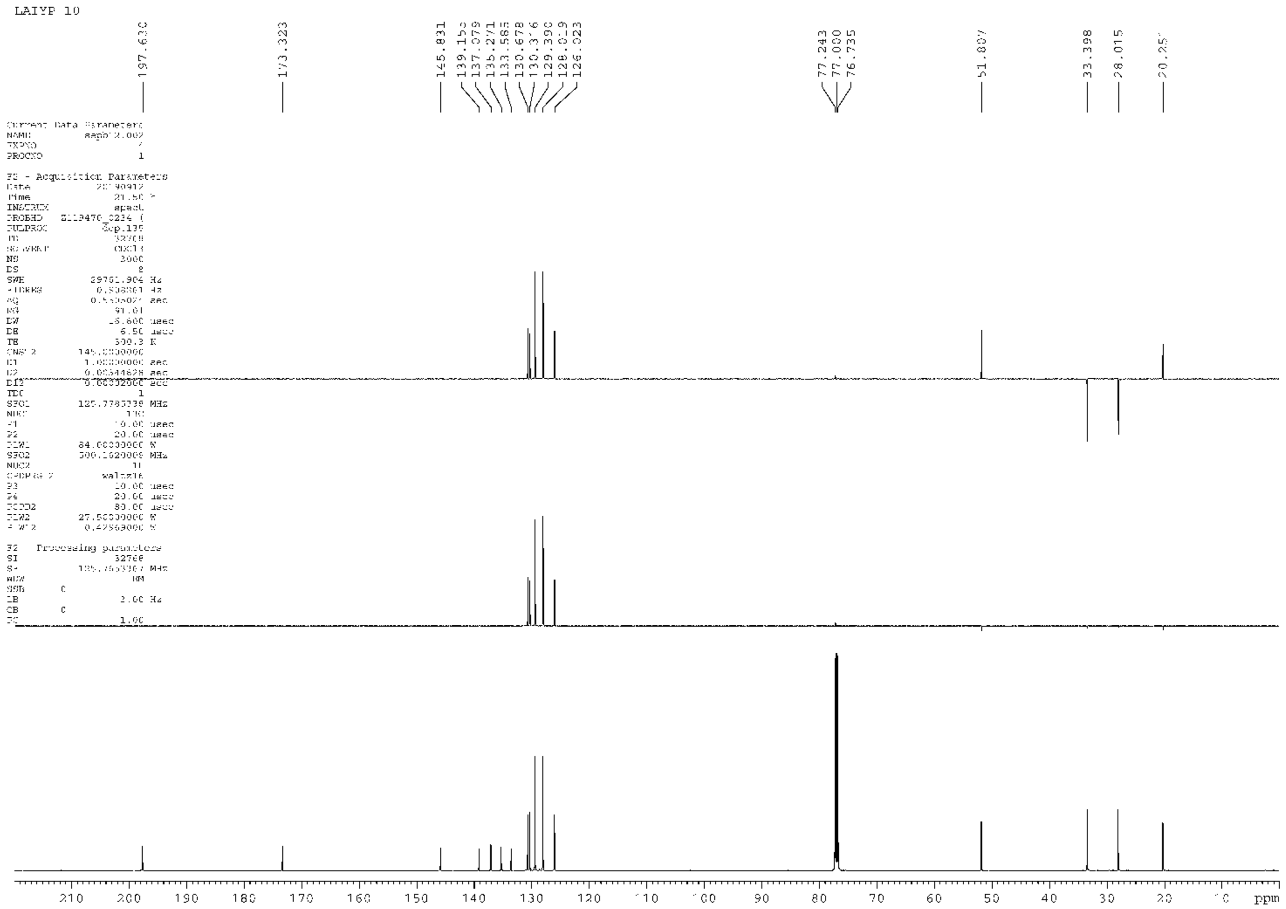

^{13}C-DEPT-135 NMR

5e

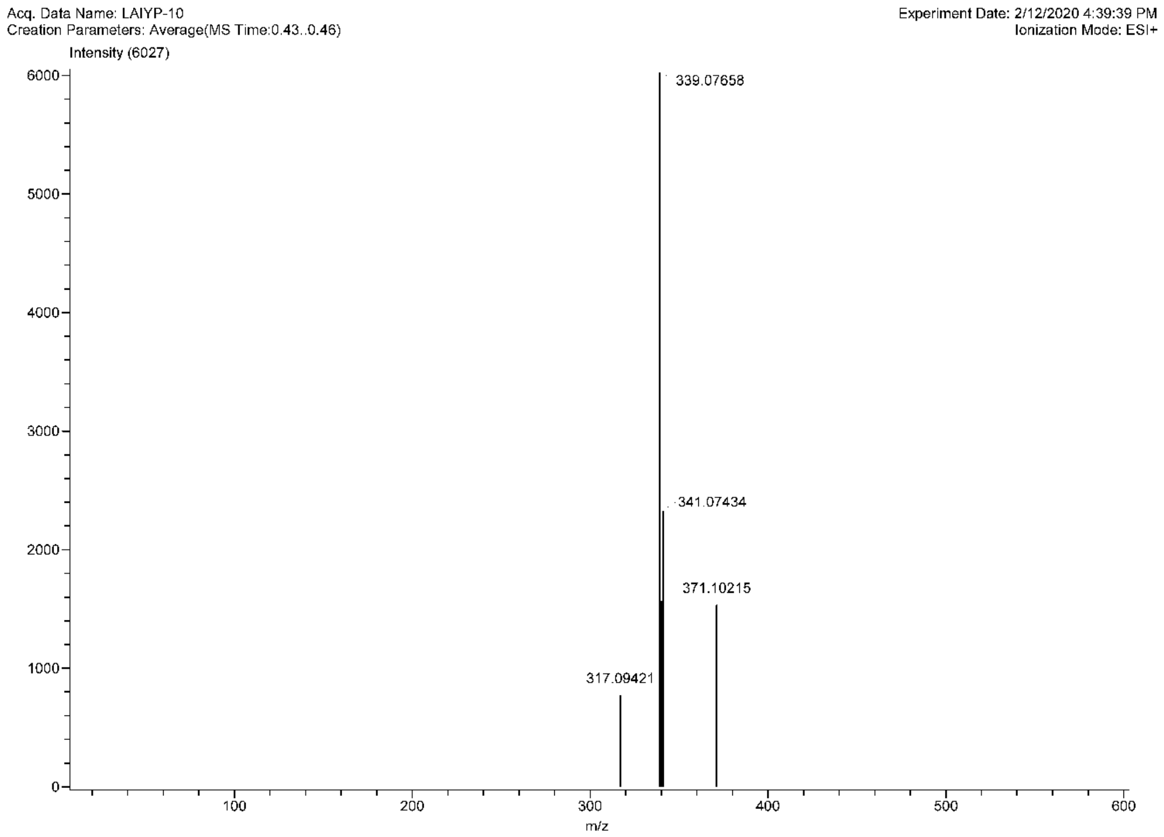

Acq. Data Name: LAIYP-10
Creation Parameters: Average(MS Time:0.43..0.46)
Experiment Date: 2/12/2020 4:39:39 PM
Ionization Mode: ESI+
Intensity (6027)
339.07658
341.07434
371.10215
317.09421
m/z

HR ESI-MS

5e

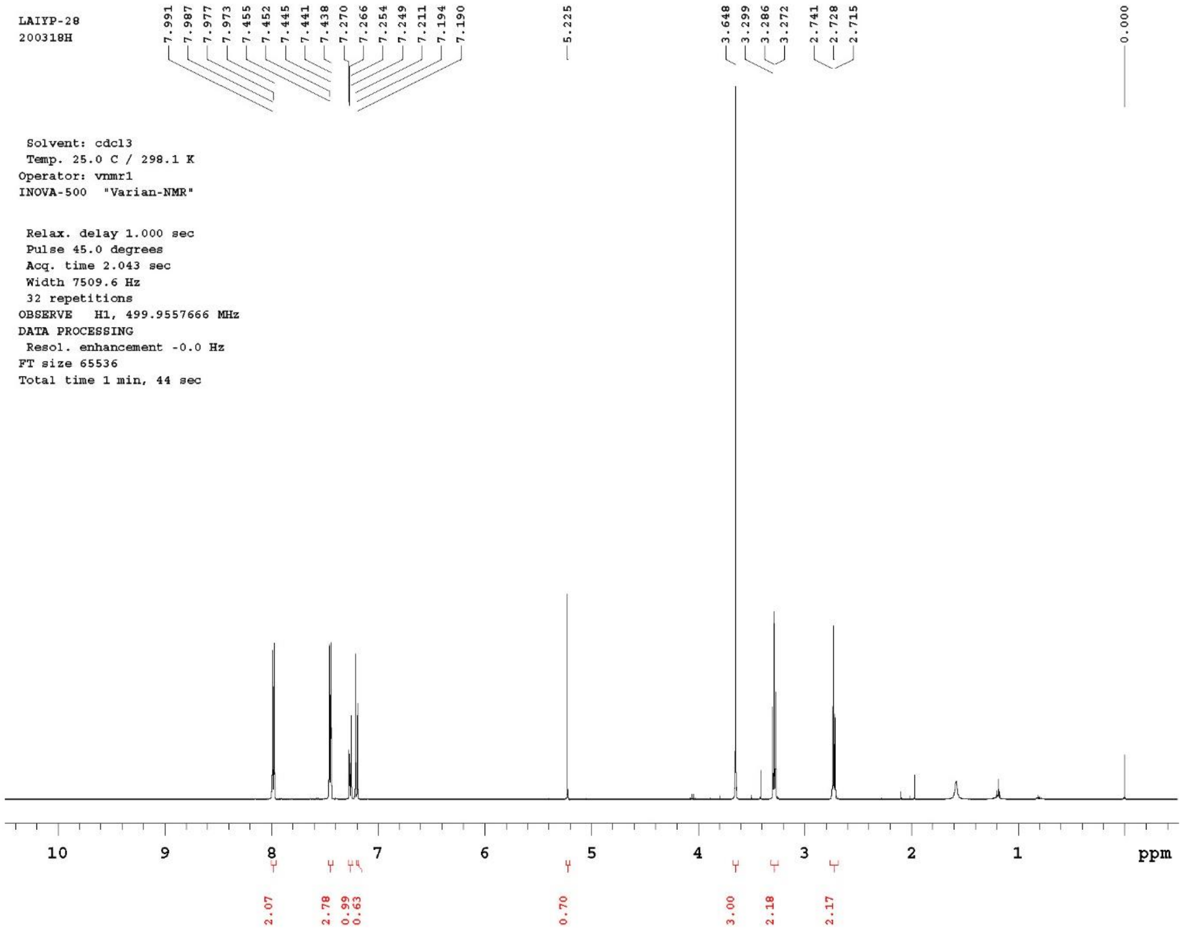

LAIYP-28
200318H
7.991
7.987
7.977
7.973
7.455
7.452
7.445
7.441
7.438
7.270
7.266
7.254
7.249
7.211
7.194
7.190
5.225
3.648
3.299
3.286
3.272
2.741
2.728
2.715
0.000
Solvent: cdcl3
Temp. 25.0 C / 298.1 K
Operator: vnmr1
INOVA-500 "Varian-NMR"
Relax. delay 1.000 sec
Pulse 45.0 degrees
Acq. time 2.043 sec
Width 7509.6 Hz
32 repetitions
OBSERVE H1, 499.9557666 MHz
DATA PROCESSING
Resol. enhancement -0.0 Hz
FT size 65536
Total time 1 min, 44 sec
10
9
8
7
6
5
4
3
2
1
ppm
2.07
2.78
0.99
0.63
0.70
3.00
2.18
2.17

^{1}H NMR

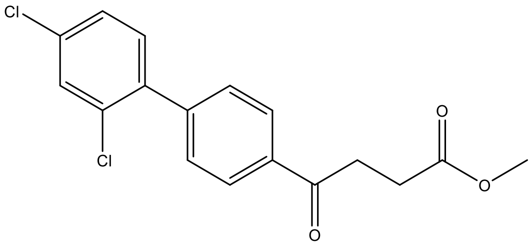

Cl
Cl
O
O
O

5f

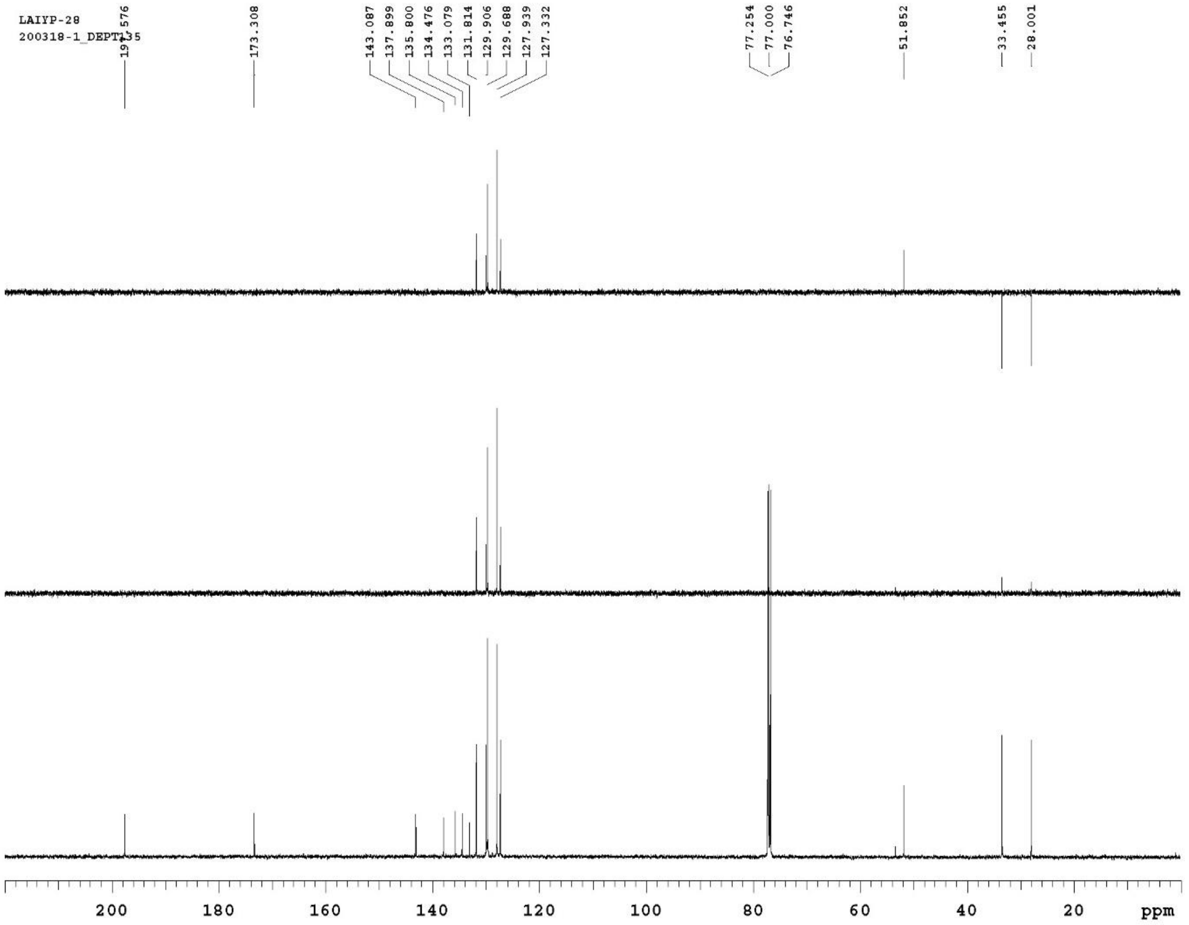

^{13}C-DEPT-135 NMR

5f

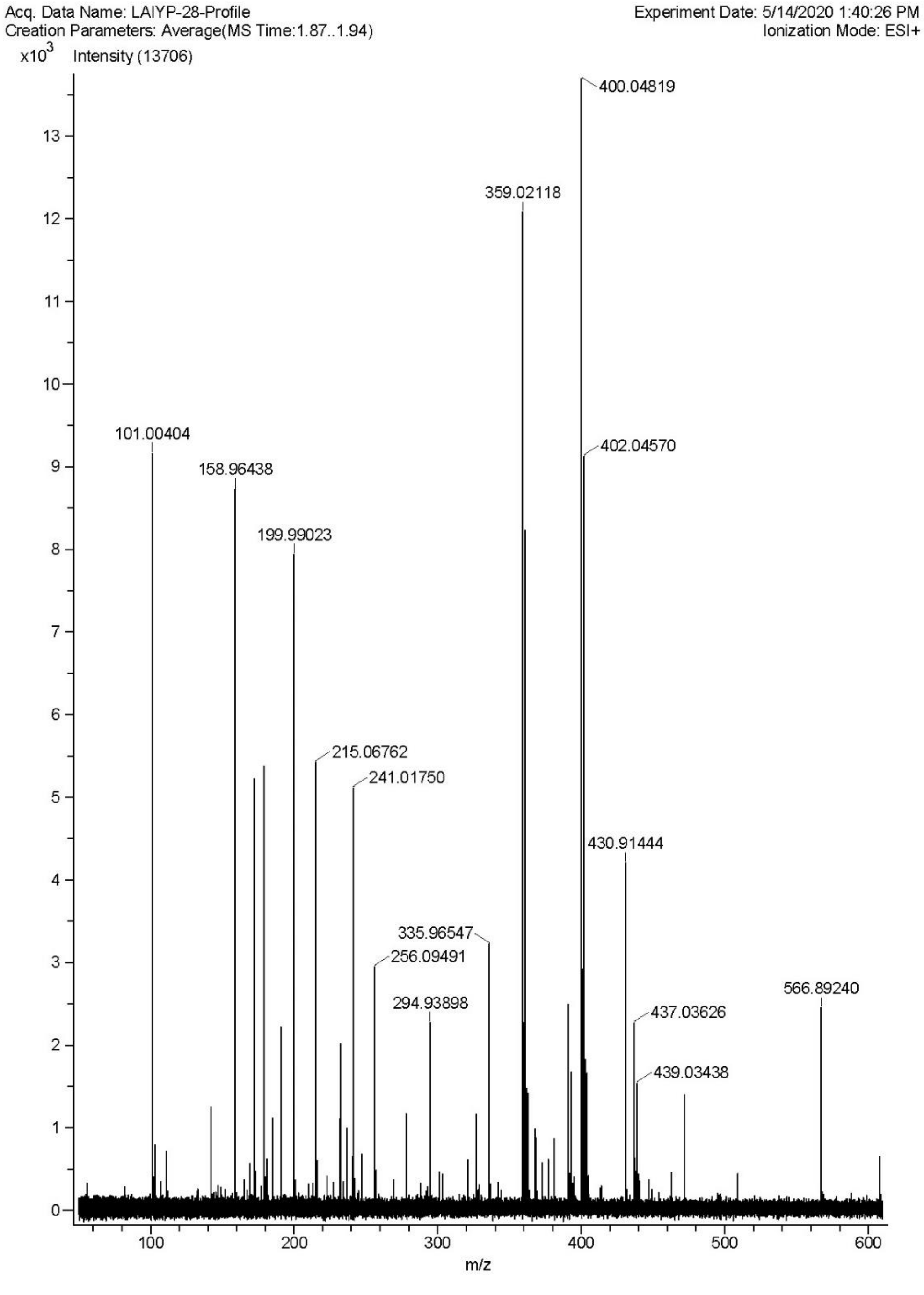
Acq. Data Name: LAIYP-28-Profile
Creation Parameters: Average(MS Time:1.87..1.94)
Experiment Date: 5/14/2020 1:40:26 PM
Ionization Mode: ESI+
x10^3 Intensity (13706)
400.04819
359.02118
101.00404
402.04570
158.96438
199.99023
215.06762
241.01750
430.91444
335.96547
256.09491
566.89240
294.93898
437.03626
439.03438
m/z

HR ESI-MS

5f

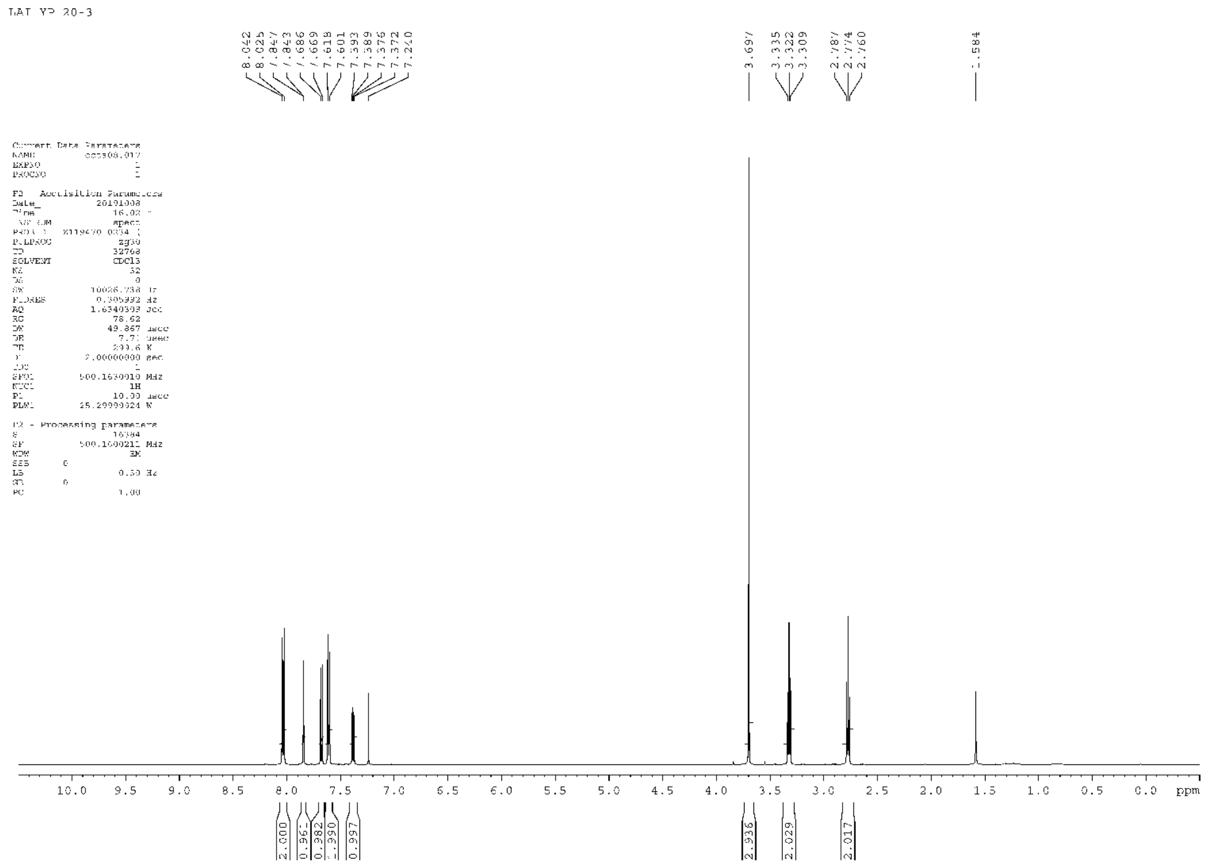
8.042
8.025
7.847
7.843
7.686
7.669
7.618
7.601
7.393
7.389
7.376
7.372
7.240
3.697
3.335
3.322
3.309
2.787
2.774
2.760
1.584
ppm
2.000
0.982
0.997
2.936
2.029
2.017

^{1}H NMR

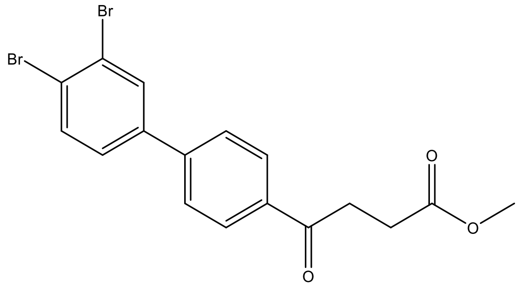
Br
Br
O
O
O

5g

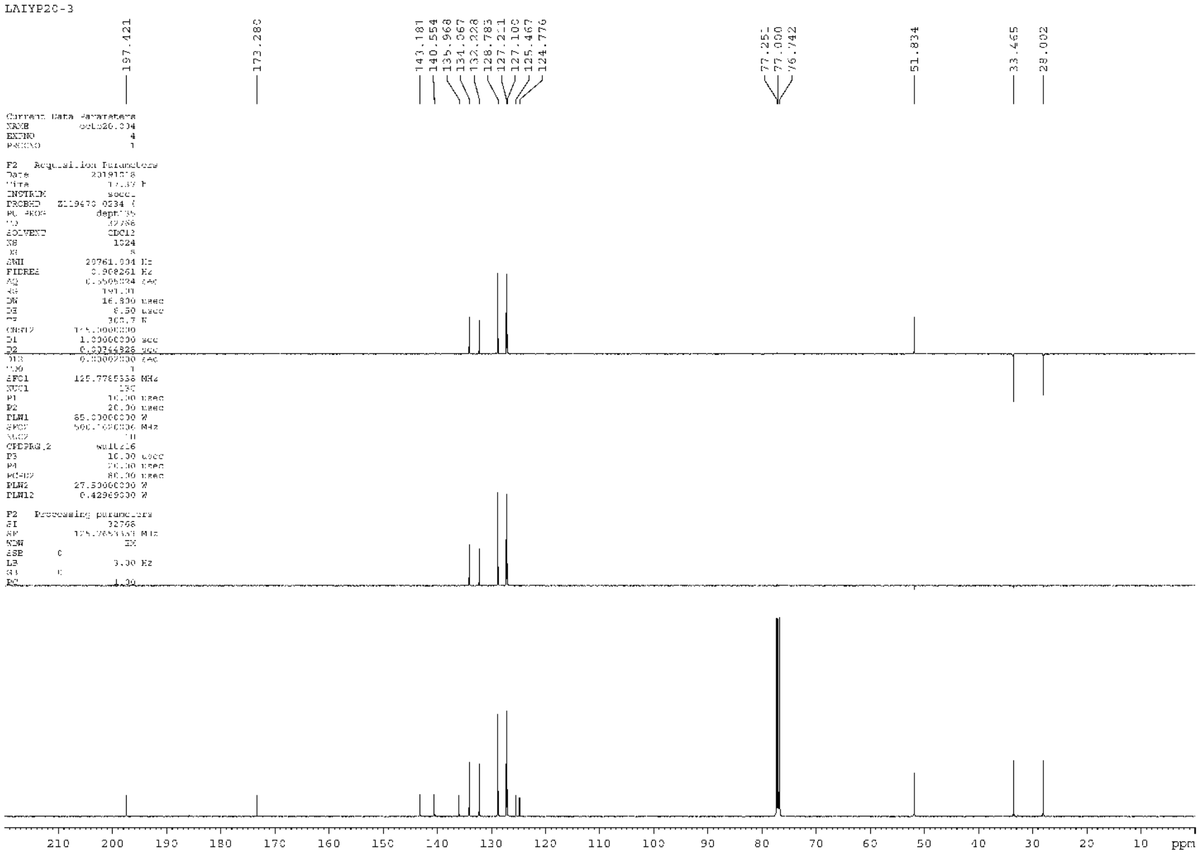

^{13}C-DEPT-135 NMR

5g

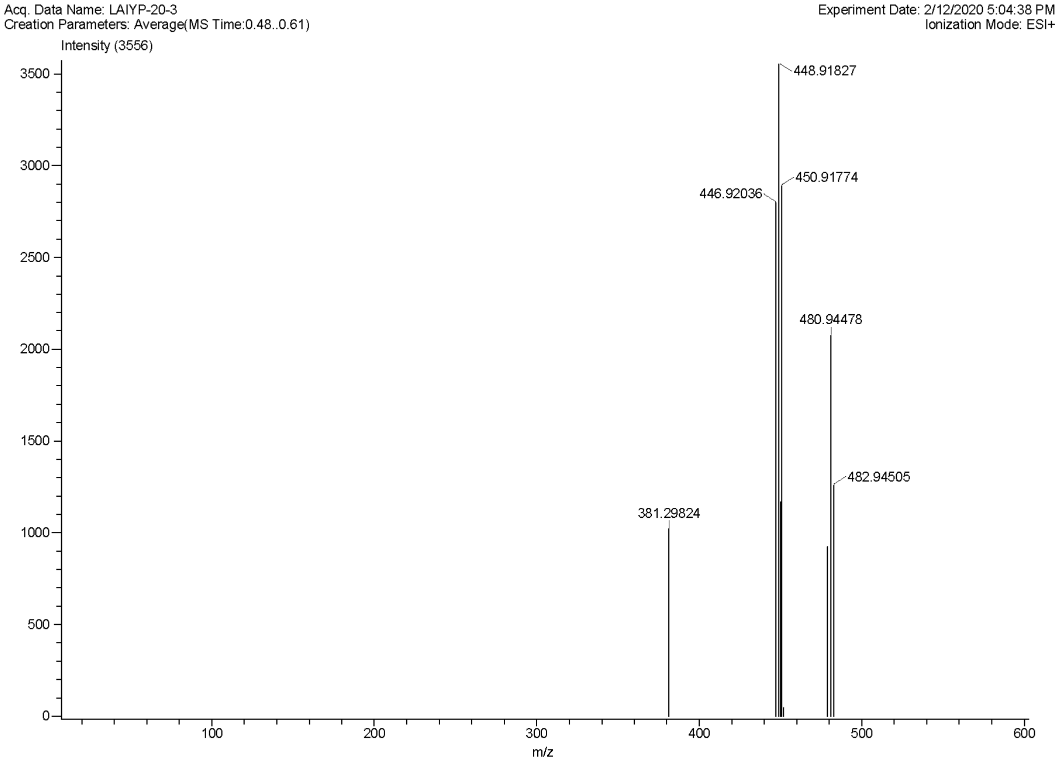

Acq. Data Name: LAIYP-20-3
Creation Parameters: Average(MS Time:0.48..0.61)
Experiment Date: 2/12/2020 5:04:38 PM
Ionization Mode: ESI+
Intensity (3556)
448.91827
450.91774
446.92036
480.94478
482.94505
381.29824
m/z

HR ESI-MS

5g

2019.10.06 LAIYP-20 fral
inject 0.4 mL 50 mg/0.1 mL +CHCl3 to 1.2 ml total, EtOA
c/n-Hexane 1:9 (500 mL), Rf1 = 0.34 ,Rf2 = 0.30,Rf3 = 0
.26,Rf1 = 0.20 (TLC), semi-preparative column, 9.4 mm
x 250 mm, Si-90

===

Injection Date : 10/5/2019 12:54:50 PM
Sample Name : LAIYP-20 fral Location : Vial 1
Acq. Operator : YP-LAI
Acq. Instrument : Instrument 1
Method : C:\HPCHEM\1\METHODS\DUKE.M
Last changed : 10/5/2019 11:40:33 AM by YP-LAI
(modified after loading)

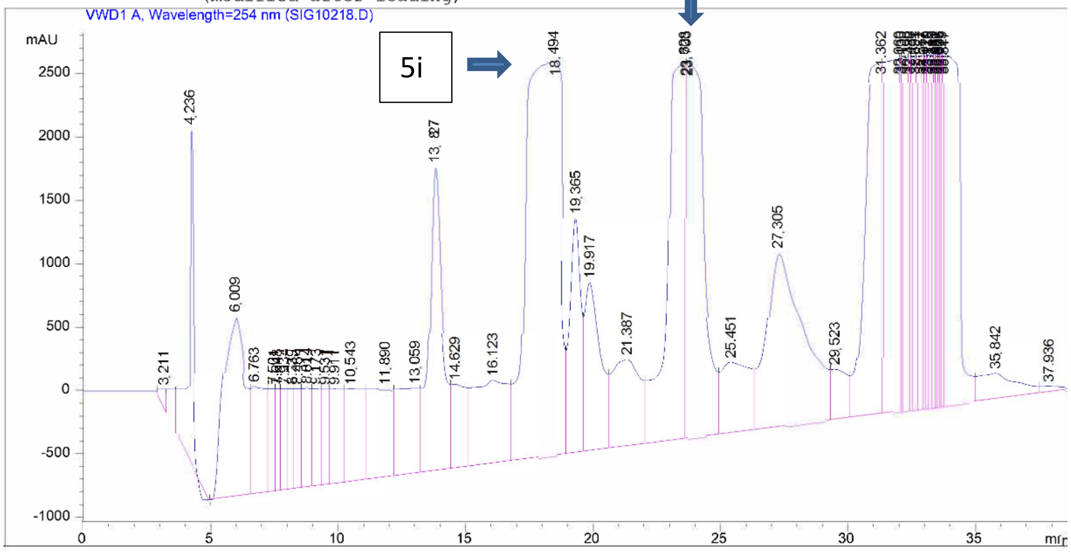

===

Area Percent Report

===

Sorted By : Signal
Multiplier : 1.0000
Dilution : 1.0000
Use Multiplier & Dilution Factor with ISTDs

Signal 1: VWD1 A, Wavelength=254 nm

Peak #	RetTime [min]	Type	Width [min]	Area mAU *s	Height [mAU]	Area %
1	3.211	BV	0.2245	2432.10547	143.09842	0.1097
2	4.236	BP	0.1840	3.40689e4	2615.73633	1.5363
3	6.009	PV	0.7165	8.34569e4	1419.56104	3.7635
4	6.763	VV	0.5110	3.44736e4	847.48108	1.5546
5	7.501	VV	0.2211	1.36426e4	810.50098	0.6152
6	7.648	VV	0.1797	1.02982e4	805.94385	0.4644
7	7.934	VV	0.2338	1.37207e4	798.94049	0.6187
8	8.239	VV	0.2152	1.17117e4	790.04535	0.5281
9	8.464	VV	0.2321	1.30842e4	786.01025	0.5900
10	8.814	VV	0.3261	1.88592e4	779.09839	0.8505
11	9.173	VV	0.3243	1.86384e4	766.81598	0.8405

HPLC chromatogram of the mixture of 5g + 5h + 5i, Eluents used were EtOAc/n-hexane 1:9 at 3 mL/min.

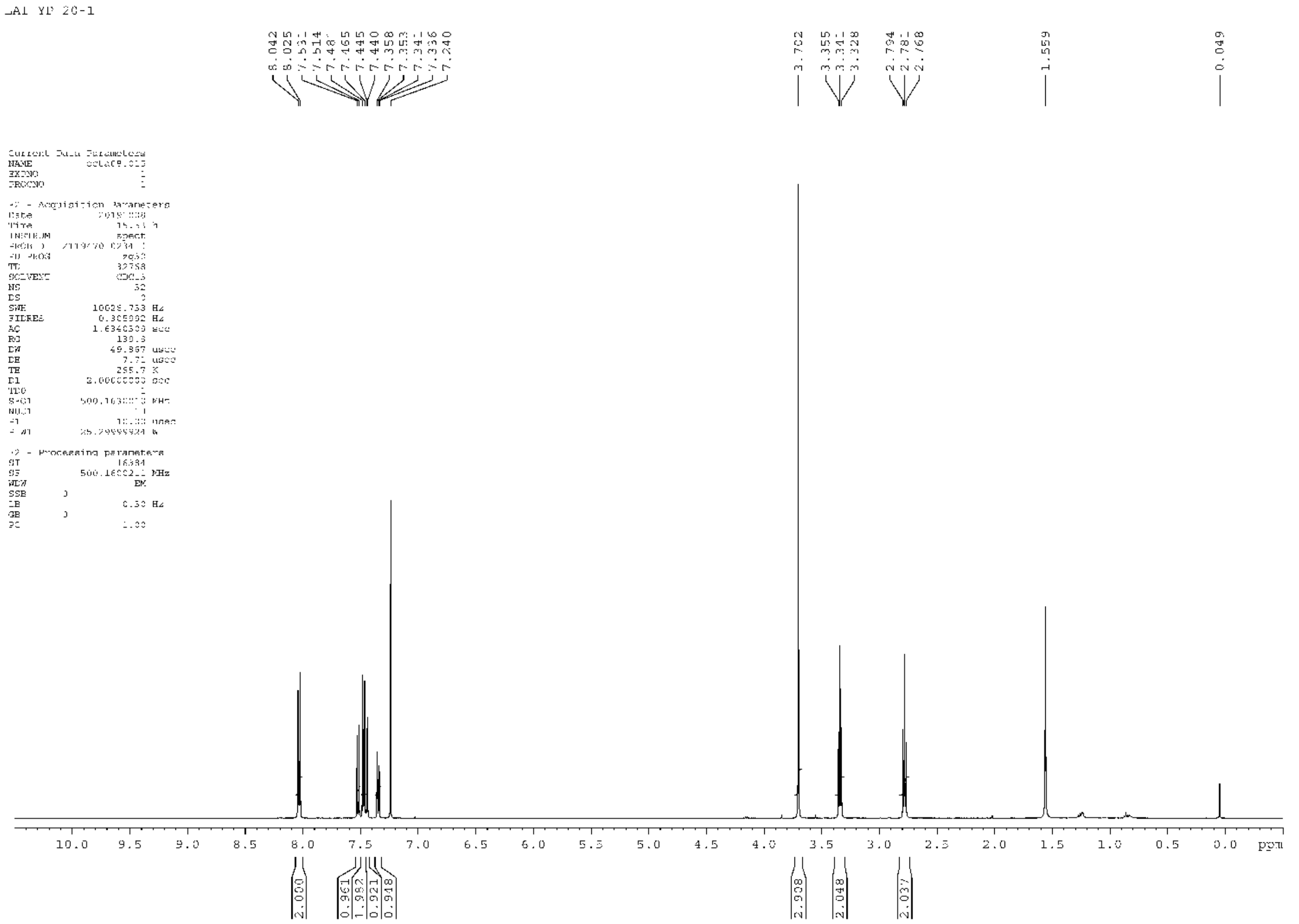

^{1}H NMR

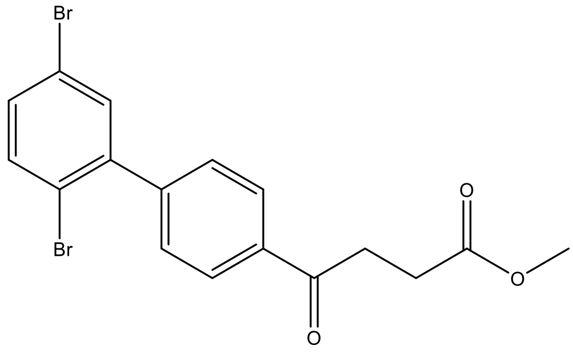

5h

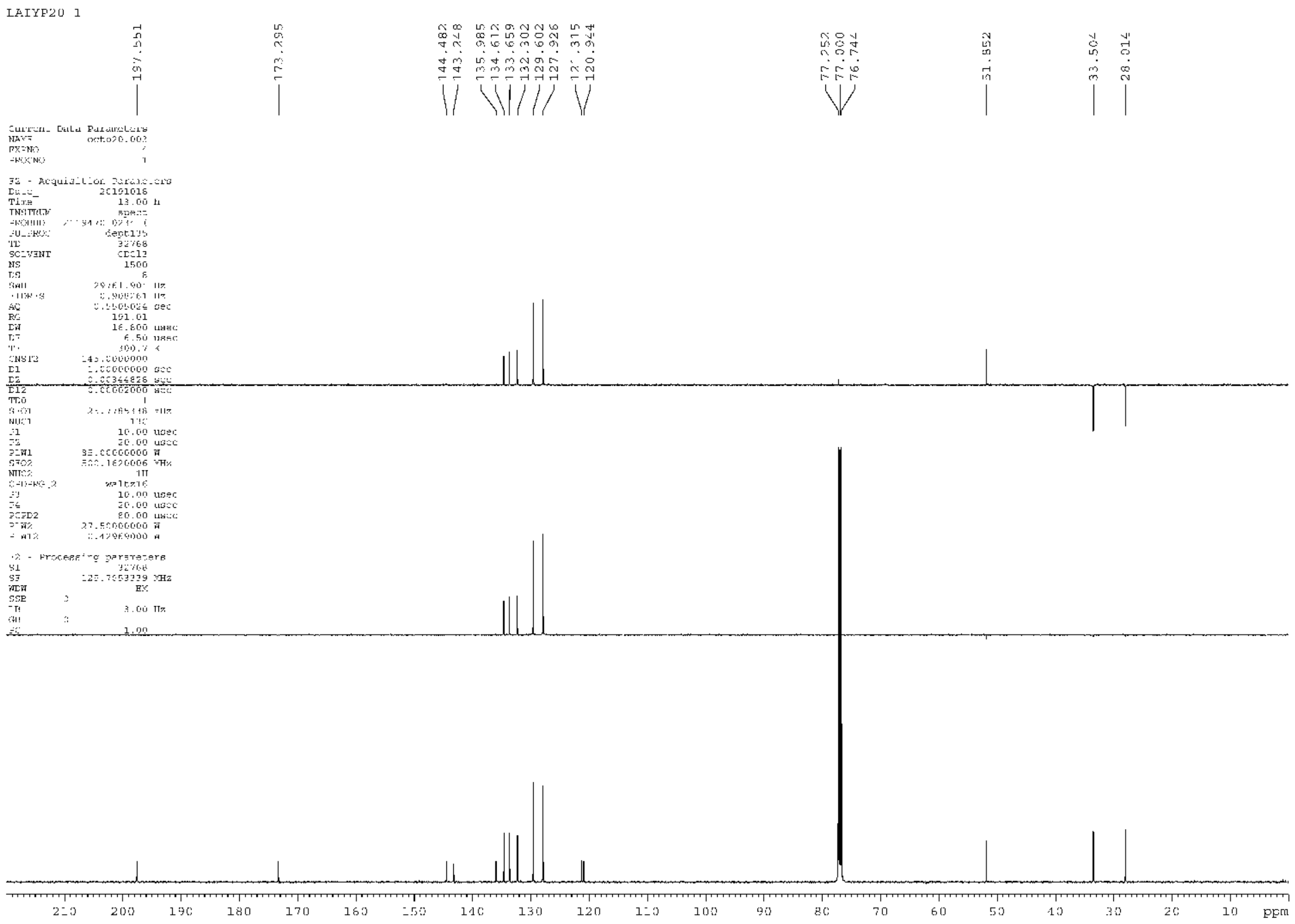

^{13}C-DEPT-135 NMR

5h

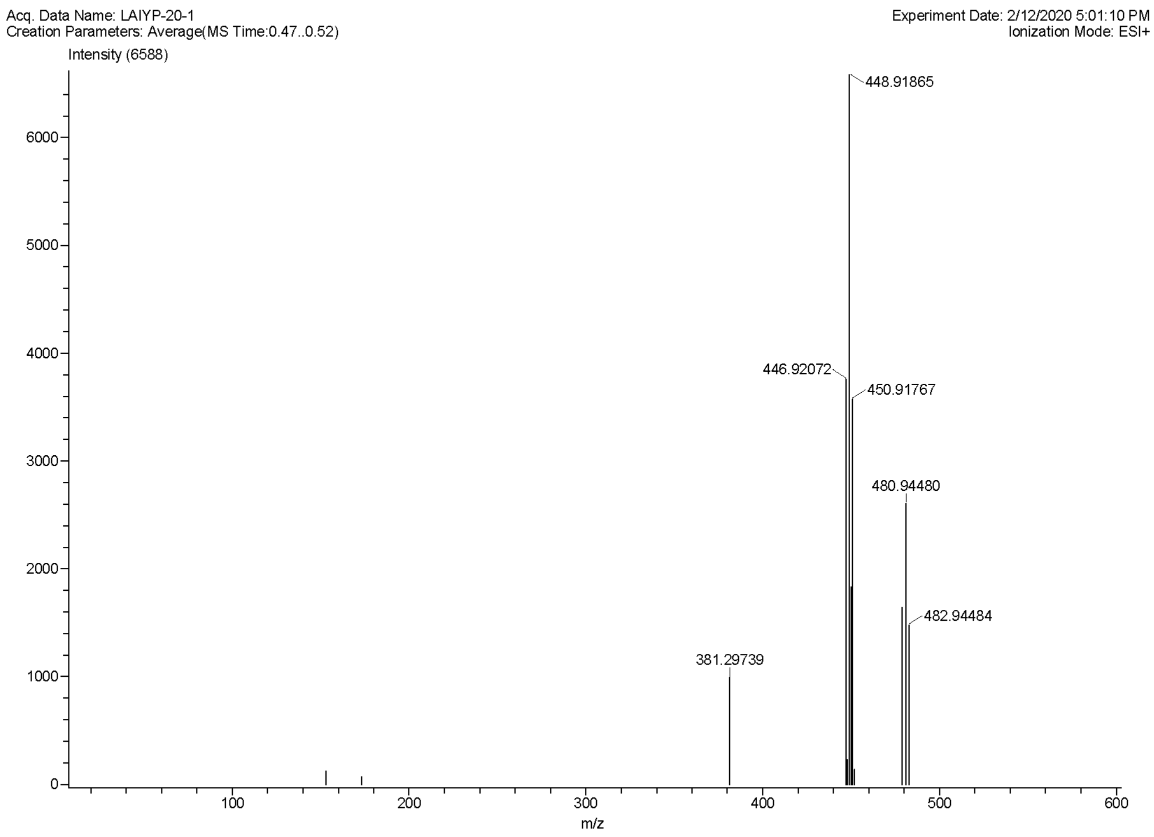

Acq. Data Name: LAIYP-20-1
Creation Parameters: Average(MS Time:0.47..0.52)
Experiment Date: 2/12/2020 5:01:10 PM
Ionization Mode: ESI+
Intensity (6588)
448.91865
446.92072
450.91767
480.94480
482.94484
381.29739
6000
5000
4000
3000
2000
1000
0
100
200
300
400
500
600
m/z

HR ESI-MS

5h

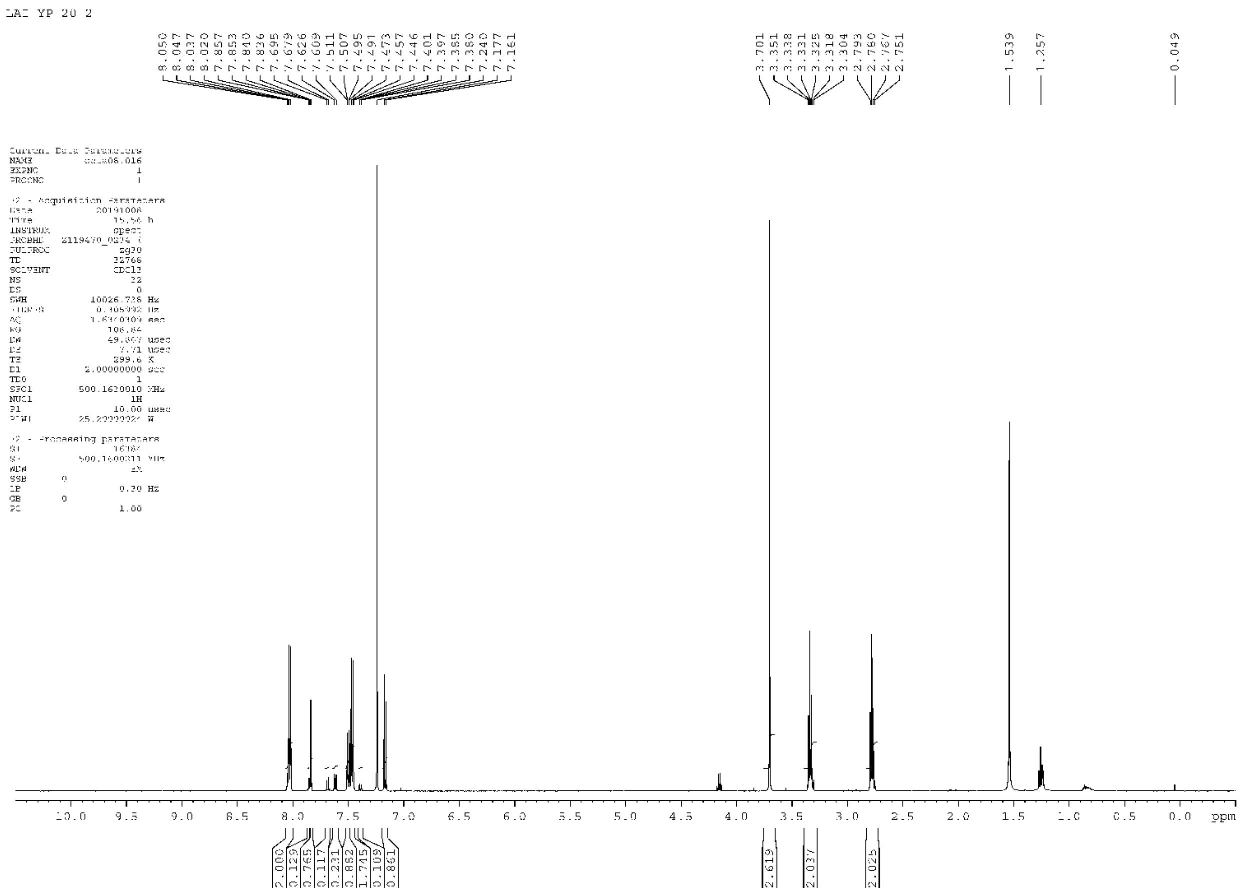

^{1}H NMR

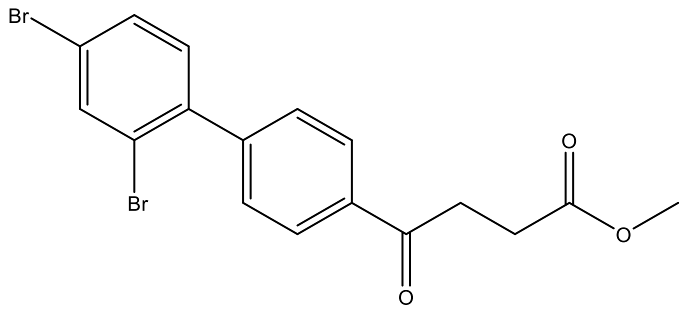

5i

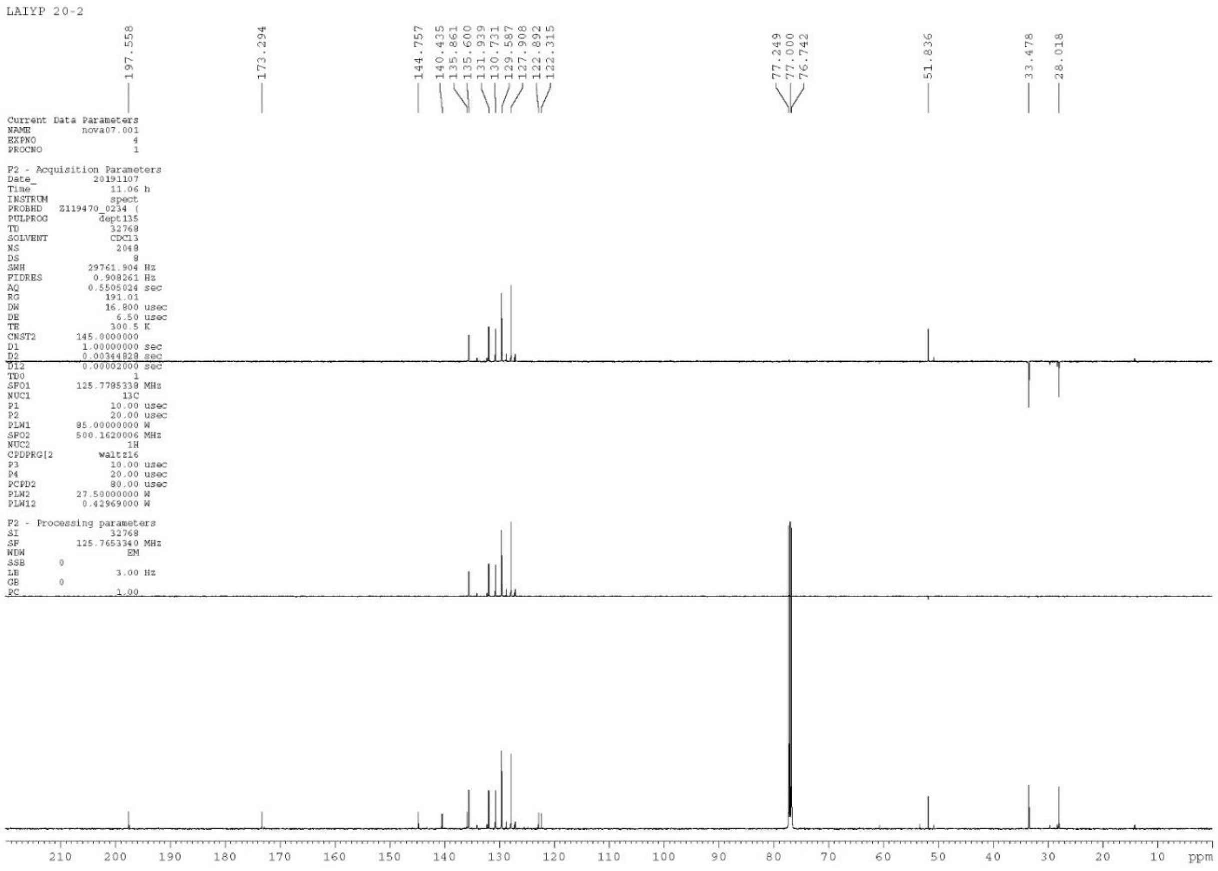

^{13}C-DEPT-135 NMR

5i

2019.10.21 LAIYP-20-2
inject 20 uL dilute with 0.3 mL EtOAc/n-Hexane 1:9 30
0 mL + EtOAc/n-Hexane 1:10 550 mL +CHCl3 to 1.5 ml tota
l, EtOAc/n-Hexane 3:7 (500 mL) Rf=0.30 ~ 0.10
semi-preparative column, 9.4 mm x 250 mm, Si-90

===

Injection Date : 10/20/2019 1:27:05 PM
Sample Name : LAIYP-20 -2 Location : Vial 1
Acq. Operator : YP-LAI
Acq. Instrument : Instrument 1
Method : C:\HPCHEM\1\METHODS\DUKE.M
Last changed : 10/20/2019 9:31:41 AM by YP-LAI
(modified after loading)

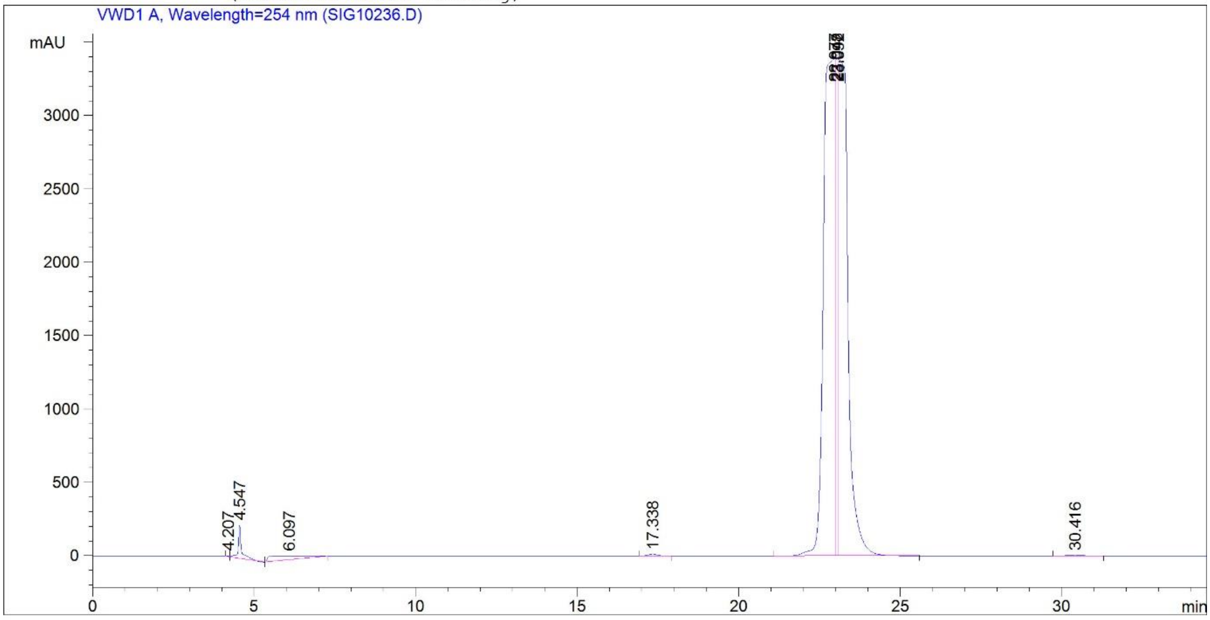

===
Area Percent Report
===

Sorted By : Signal
Multiplier : 1.0000
Dilution : 1.0000
Use Multiplier & Dilution Factor with ISTDs

Signal 1: VWD1 A, Wavelength=254 nm

Peak #	RetTime [min]	Type	Width [min]	Area mAU *s	Height [mAU]	Area %
1	4.207	BV	0.1053	47.81774	6.35252	0.0272
2	4.547	VP	0.0984	1645.23157	225.08220	0.9370
3	6.097	VB	1.0634	2396.13770	26.83969	1.3647
4	17.338	BB	0.3035	242.25626	12.25061	0.1380
5	22.977	BV	0.3143	8.79706e4	3390.79053	50.1036
6	23.048	VV	0.0476	1.12804e4	3387.78906	6.4247
7	23.092	VB	0.2576	7.18218e4	3386.75708	40.9060
8	30.416	BP	0.5196	173.31084	5.12662	0.0987
Totals :				1.75577e5	1.04410e4	

HPLC Chromatogram

5i

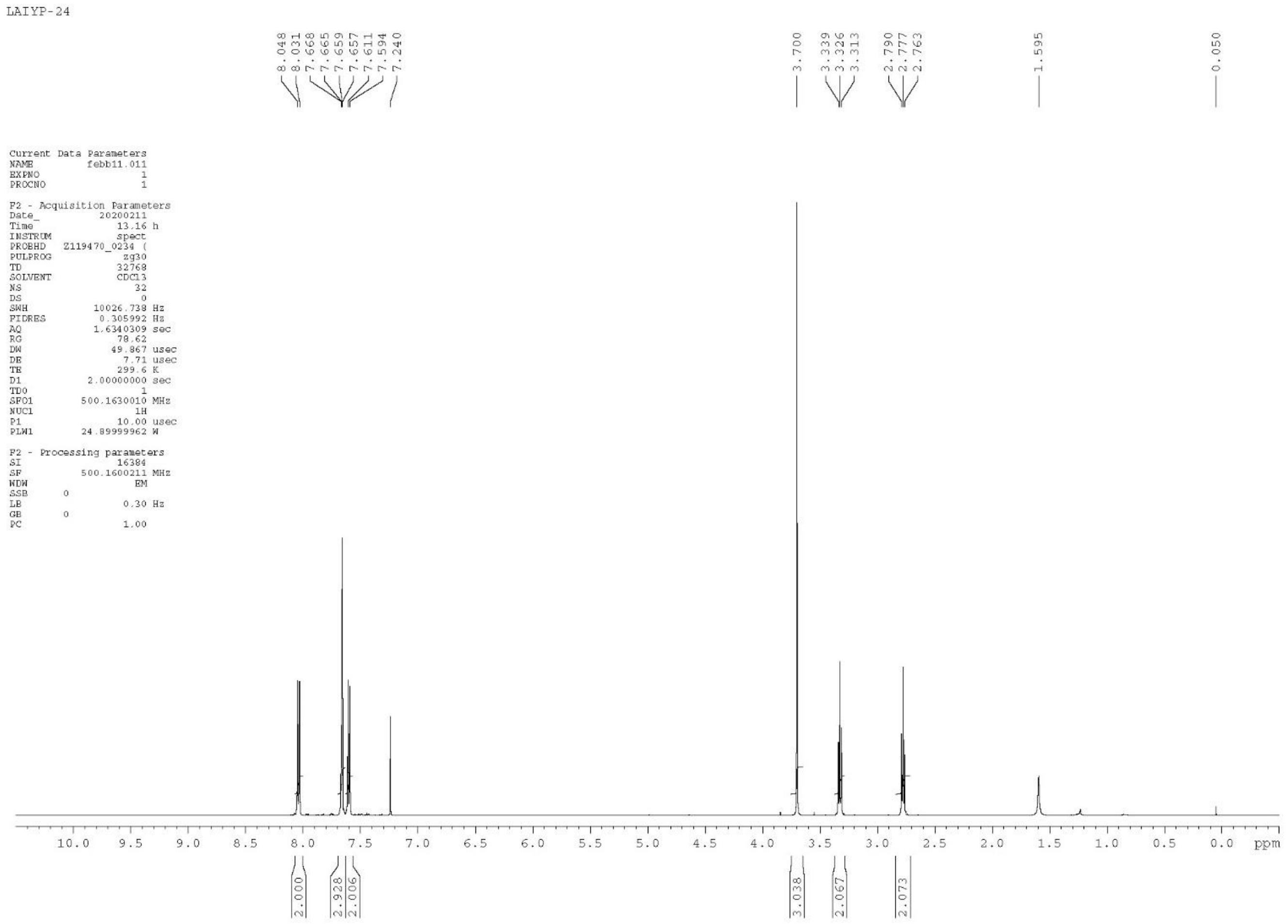
LAIYP-24
8.048
8.031
7.668
7.665
7.659
7.657
7.611
7.594
7.240
3.700
3.339
3.326
3.313
2.790
2.777
2.763
1.595
0.050
Current Data Parameters
NAME febb11.011
EXPNO 1
PROCNO 1
F2 - Acquisition Parameters
Date_ 20200211
Time 13.16 h
INSTRUM spect
PROBHD Z119470_0234 (
PULPROG zg30
TD 32768
SOLVENT CDCl3
NS 32
DS 0
SWH 10026.738 Hz
FIDRES 0.305992 Hz
AQ 1.6340309 sec
RG 78.62
DW 49.867 usec
DE 7.71 usec
TE 299.6 K
D1 2.00000000 sec
TD0 1
SFO1 500.1630010 MHz
NUC1 1H
P1 10.00 usec
PLW1 24.89999962 W
F2 - Processing parameters
SI 16384
SF 500.1600211 MHz
WDW EM
SSB 0
LB 0.30 Hz
GB 0
PC 1.00
10.0 9.5 9.0 8.5 8.0 7.5 7.0 6.5 6.0 5.5 5.0 4.5 4.0 3.5 3.0 2.5 2.0 1.5 1.0 0.5 0.0 ppm
2.000
2.928
2.006
3.038
2.067
2.073

^{1}H NMR

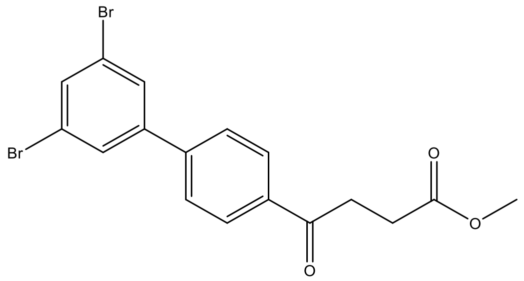
Br
Br
O
O
O

5j

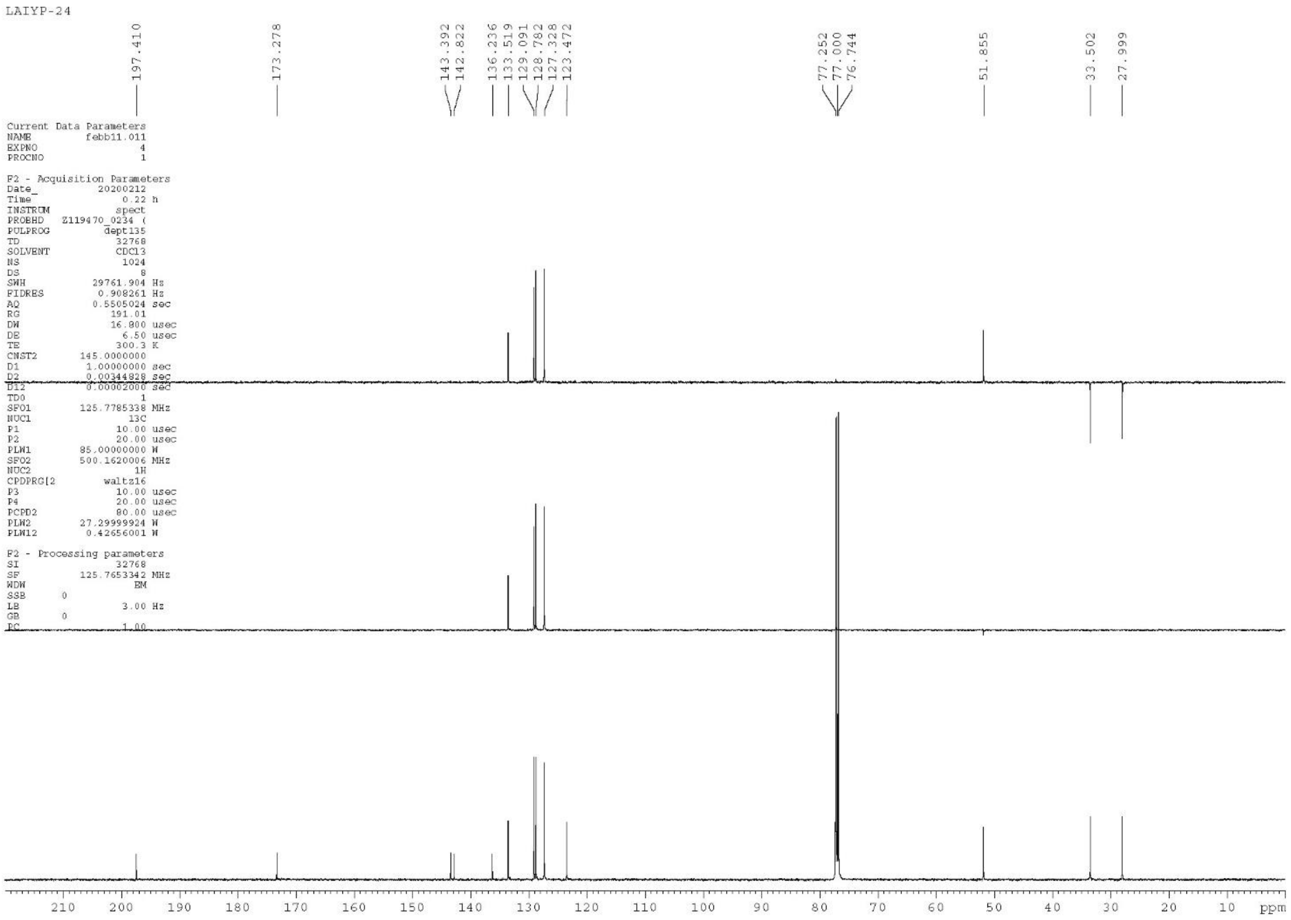

^{13}C-DEPT-135 NMR

5j

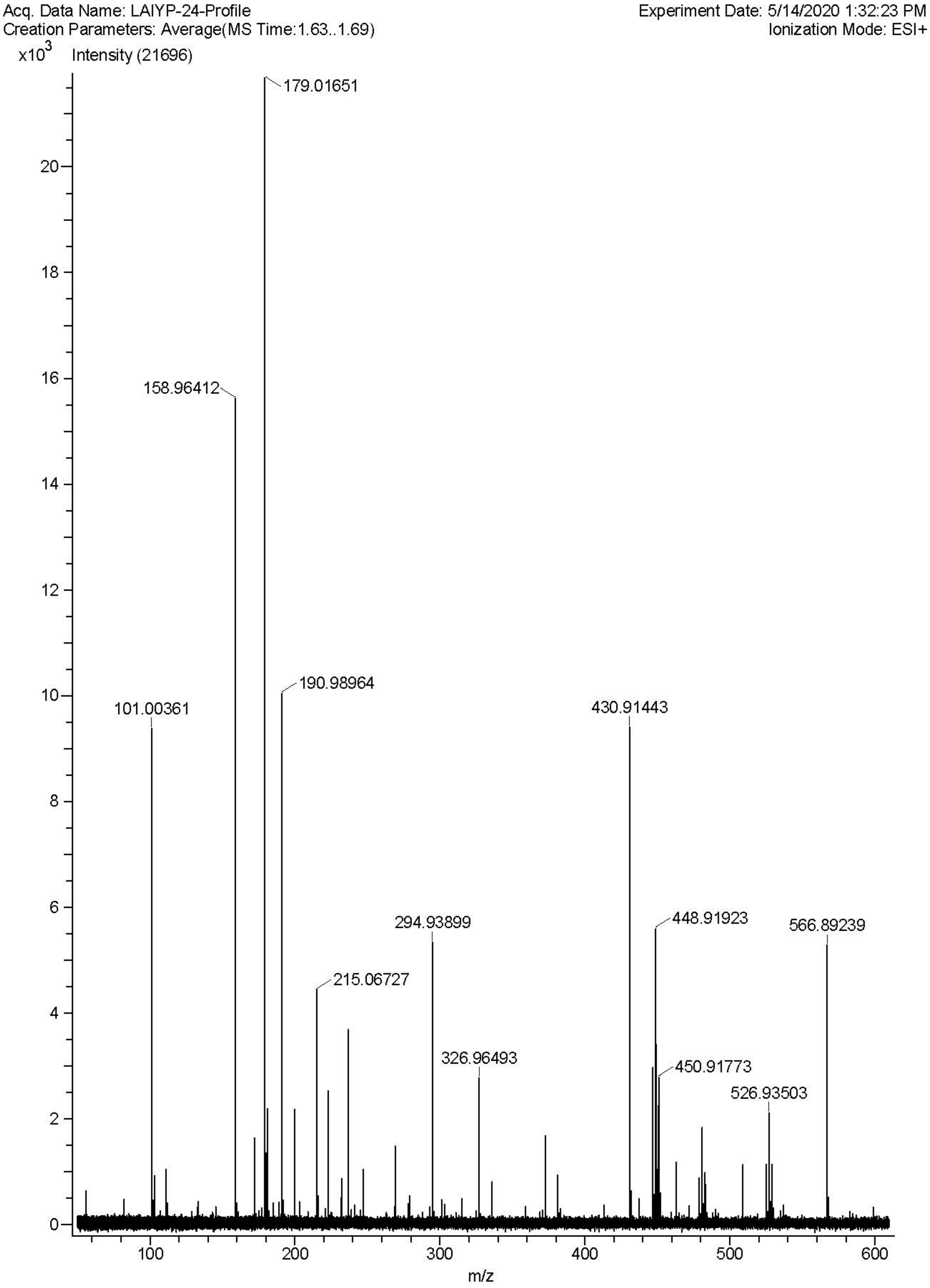

Acq. Data Name: LAIYP-24-Profile
Creation Parameters: Average(MS Time:1.63..1.69)
Experiment Date: 5/14/2020 1:32:23 PM
Ionization Mode: ESI+
x10^3 Intensity (21696)
179.01651
158.96412
190.98964
101.00361
430.91443
294.93899
448.91923
566.89239
215.06727
326.96493
450.91773
526.93503
m/z

HR ESI-MS

5j

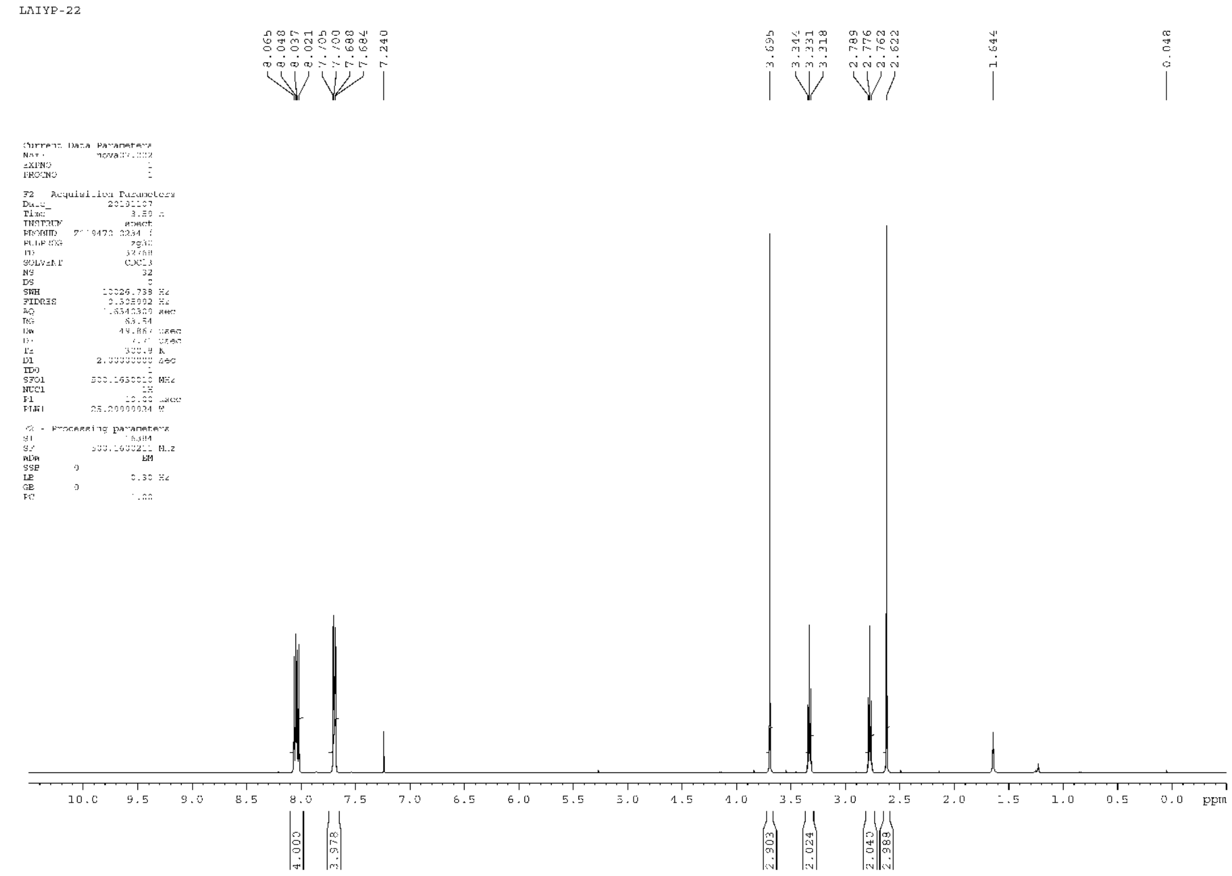
LAIYP-22
8.065
8.048
8.037
8.021
7.705
7.700
7.688
7.684
7.240
3.695
3.344
3.331
3.318
2.789
2.776
2.762
2.622
1.644
0.048
ppm
4.000
3.978
2.903
2.024
2.040
2.989

^{1}H NMR

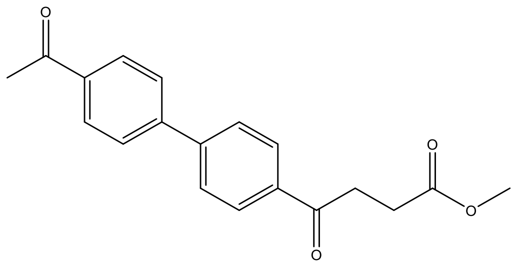

5k

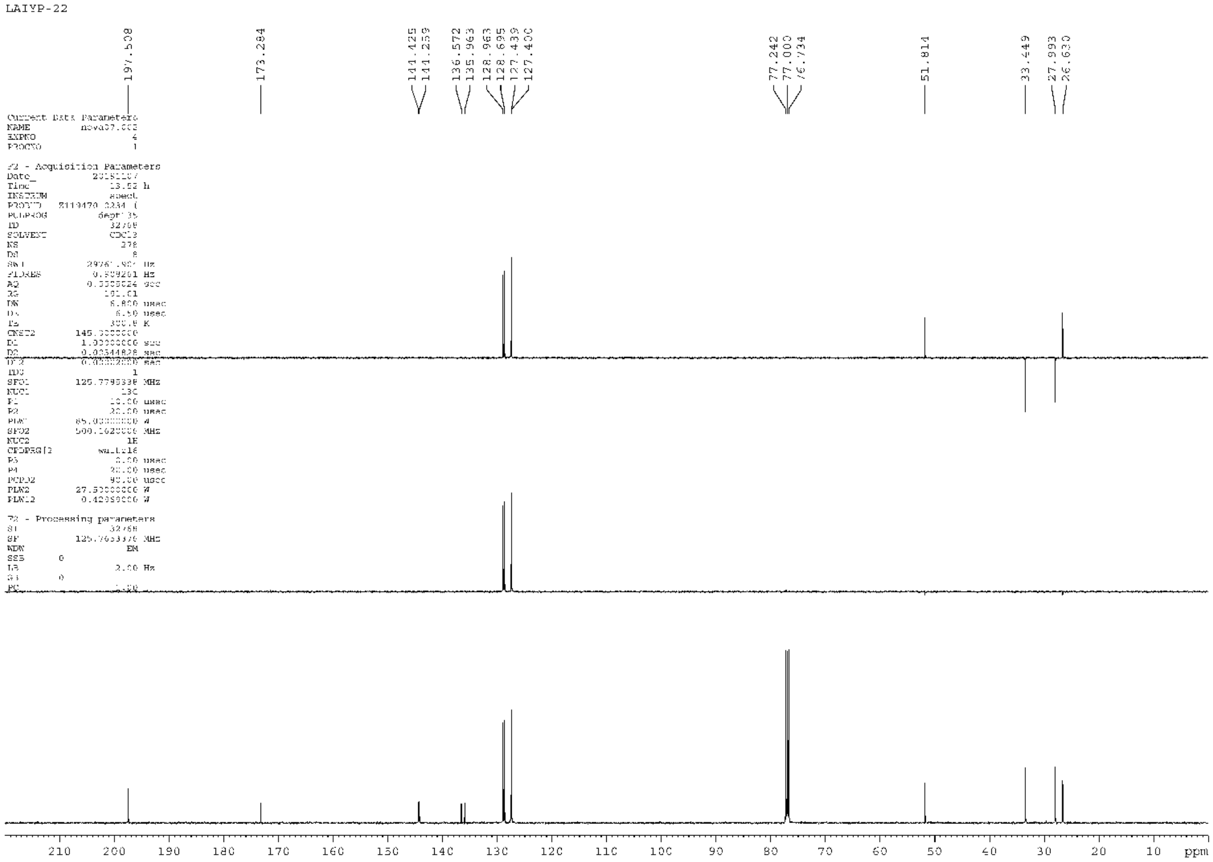

^{13}C-DEPT-135 NMR

5k

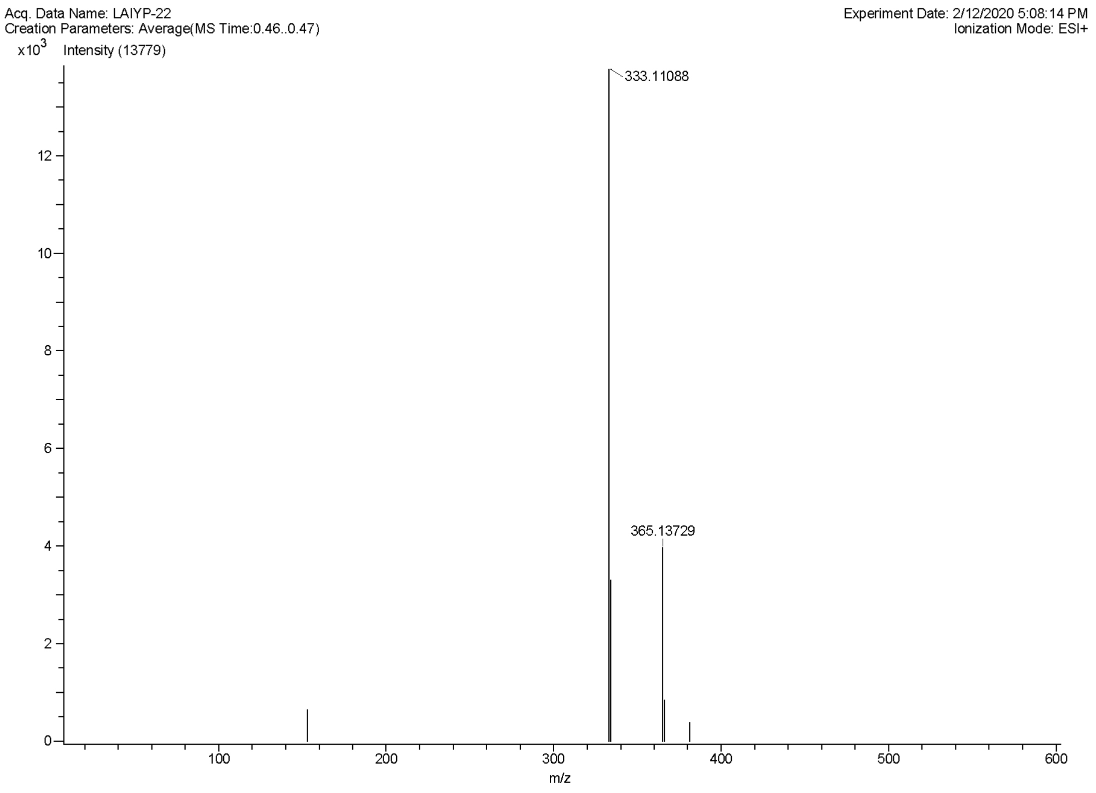
Acq. Data Name: LAIYP-22
Creation Parameters: Average(MS Time:0.46..0.47)
Experiment Date: 2/12/2020 5:08:14 PM
Ionization Mode: ESI+
x10^3 Intensity (13779)
333.11088
365.13729
m/z

HR ESI-MS

5k

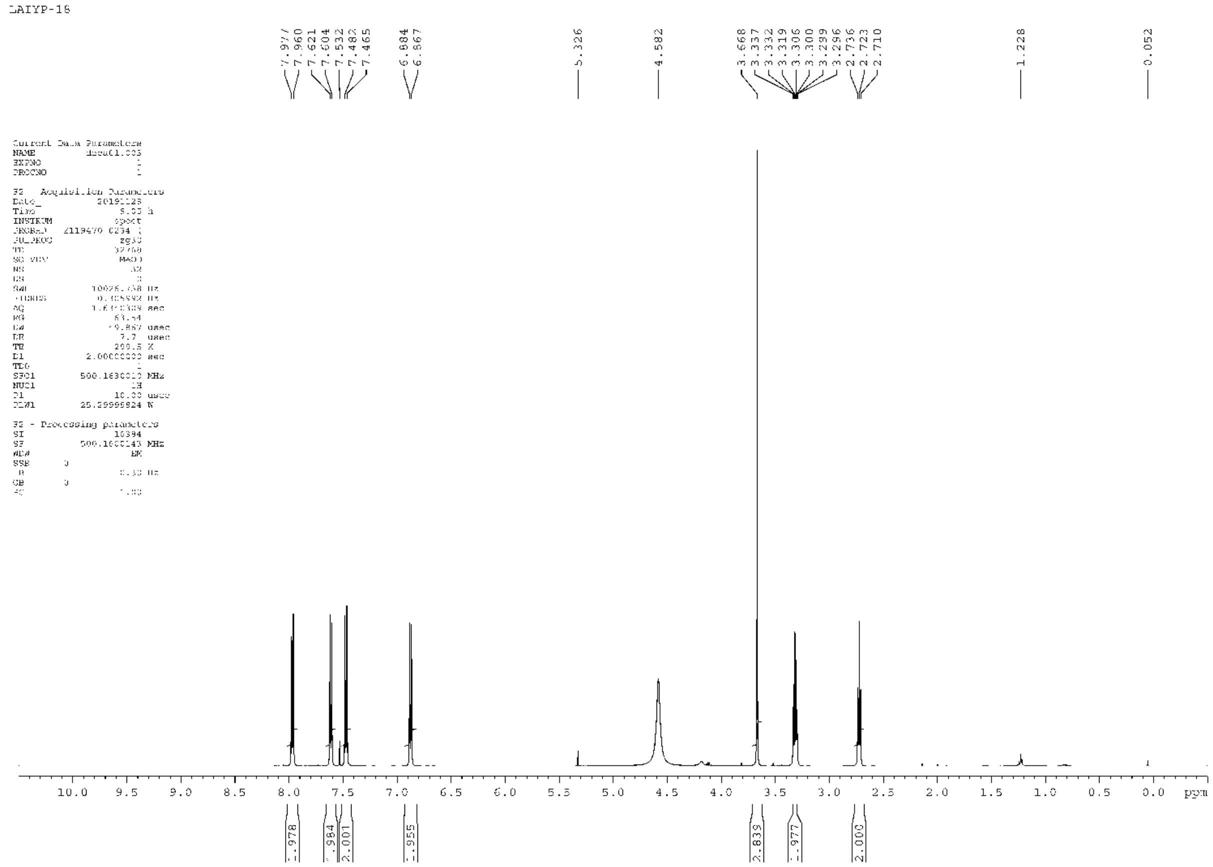
LAIYP-18
7.977
7.960
7.621
7.604
7.532
7.482
7.465
6.884
6.867
5.326
4.582
3.668
3.337
3.332
3.319
3.306
3.300
3.299
3.296
2.736
2.723
2.710
1.228
0.052
10.0 9.5 9.0 8.5 8.0 7.5 7.0 6.5 6.0 5.5 5.0 4.5 4.0 3.5 3.0 2.5 2.0 1.5 1.0 0.5 0.0 ppm

^{1}H NMR

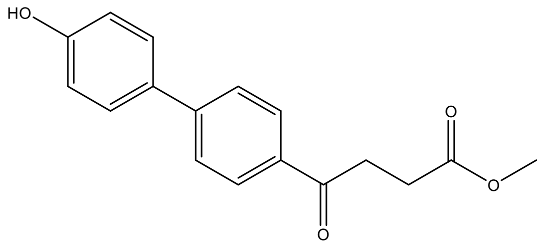
HO
O
O
O

5l

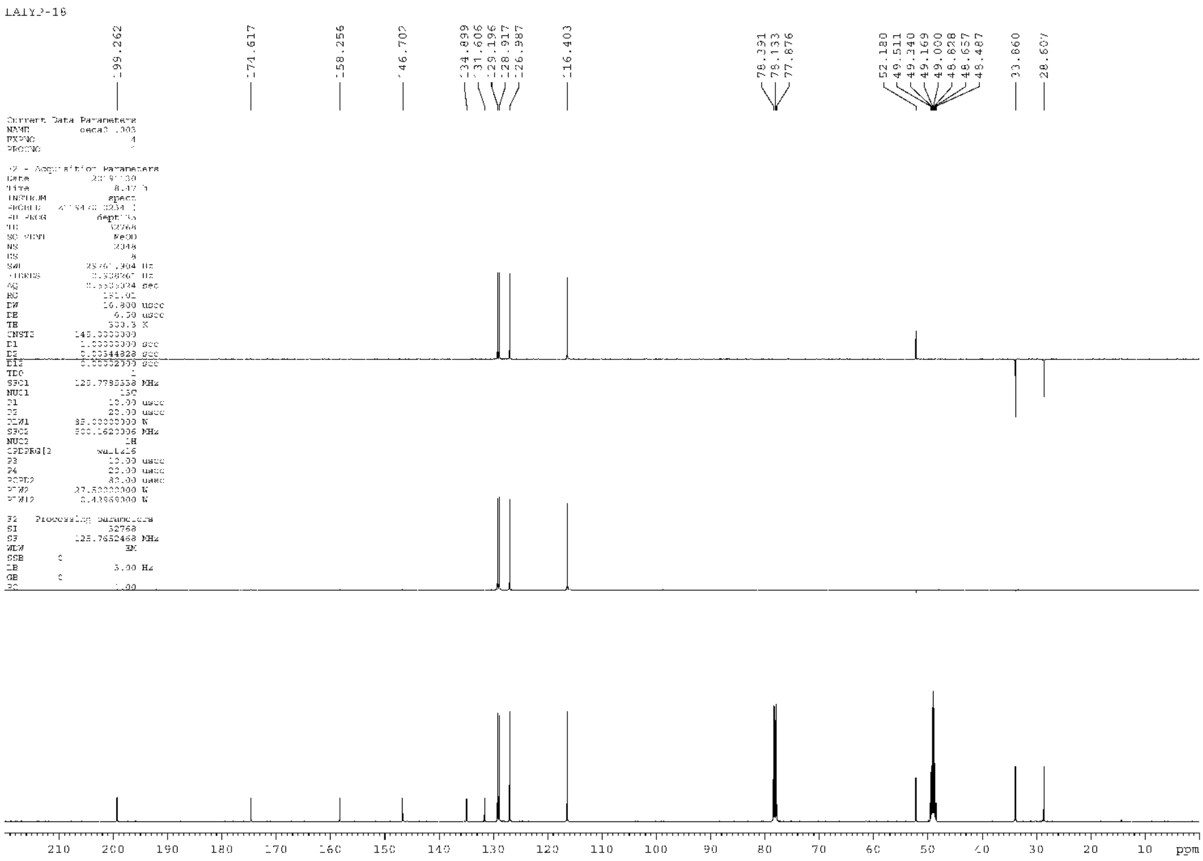

^{13}C-DEPT-135 NMR

5l

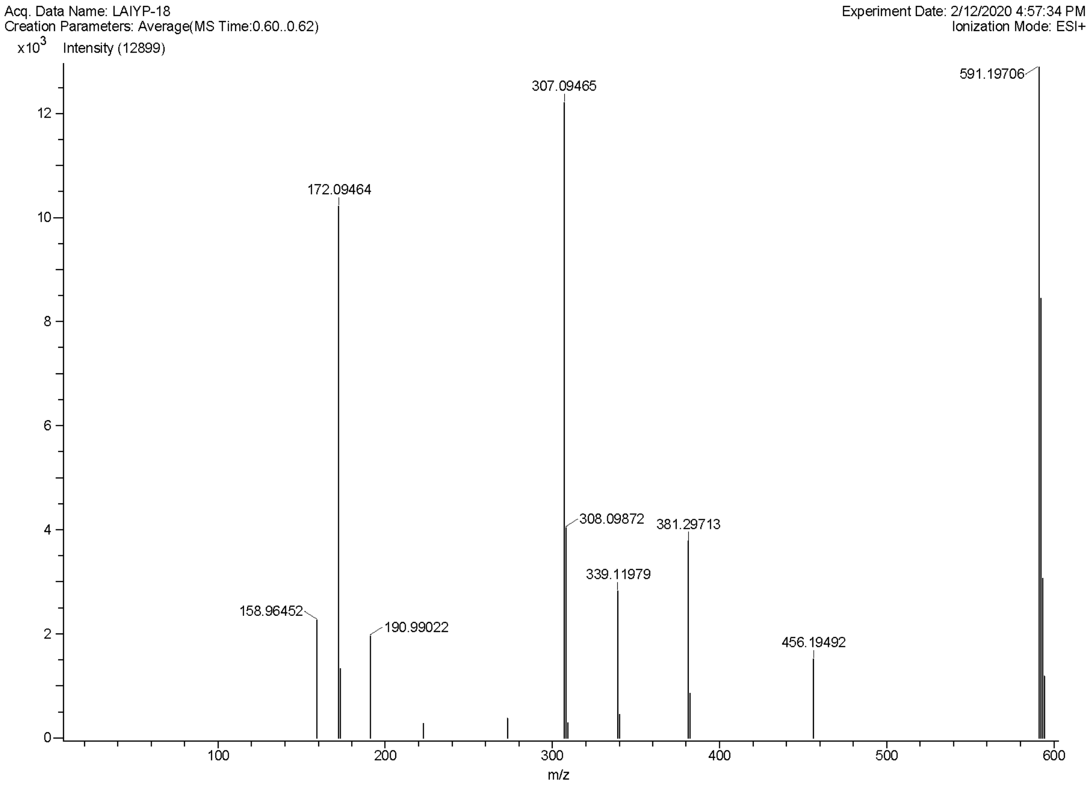

Acq. Data Name: LAIYP-18
Creation Parameters: Average(MS Time:0.60..0.62)
Experiment Date: 2/12/2020 4:57:34 PM
Ionization Mode: ESI+
x10^3 Intensity (12899)
307.09465
591.19706
172.09464
308.09872
381.29713
339.11979
158.96452
190.99022
456.19492
m/z

HR ESI-MS

5l

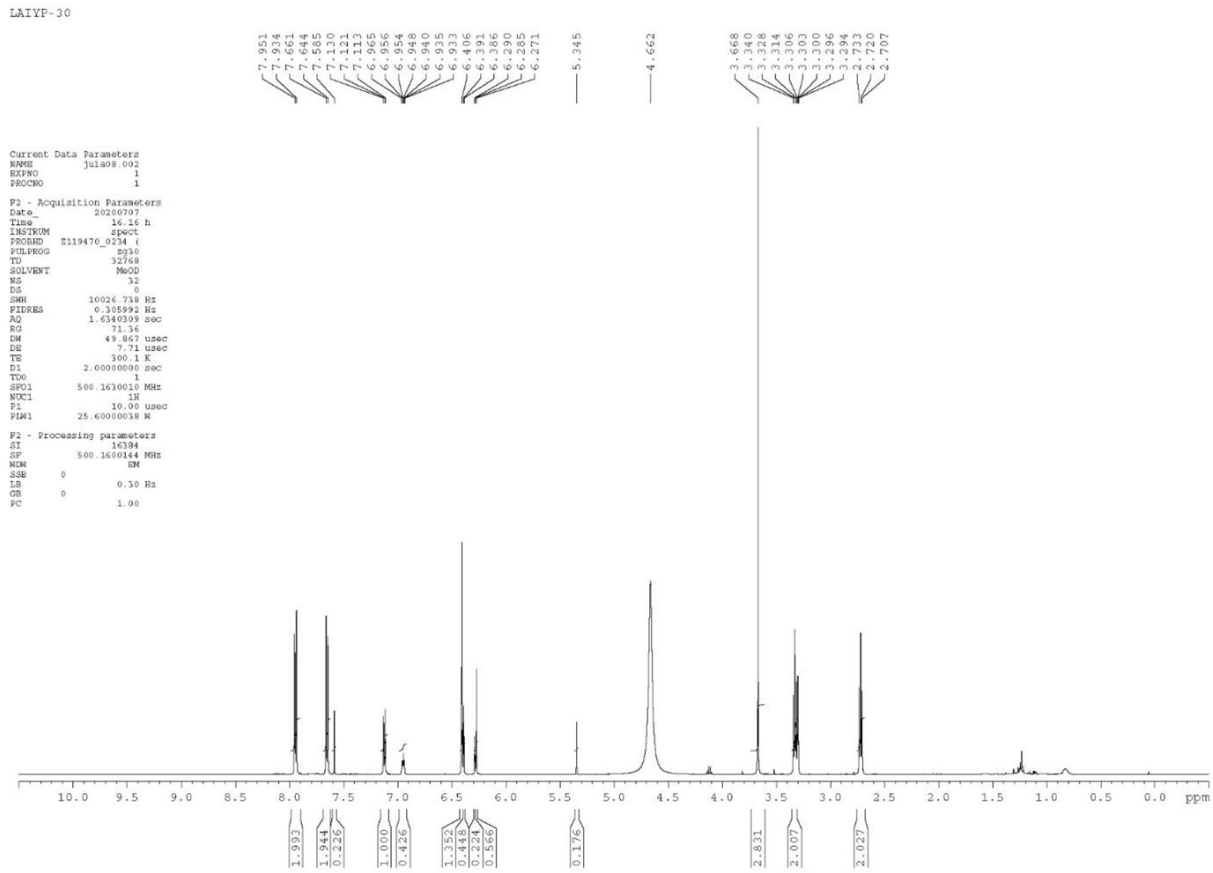

^{1}H NMR

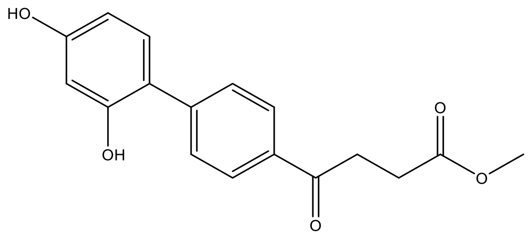

5m

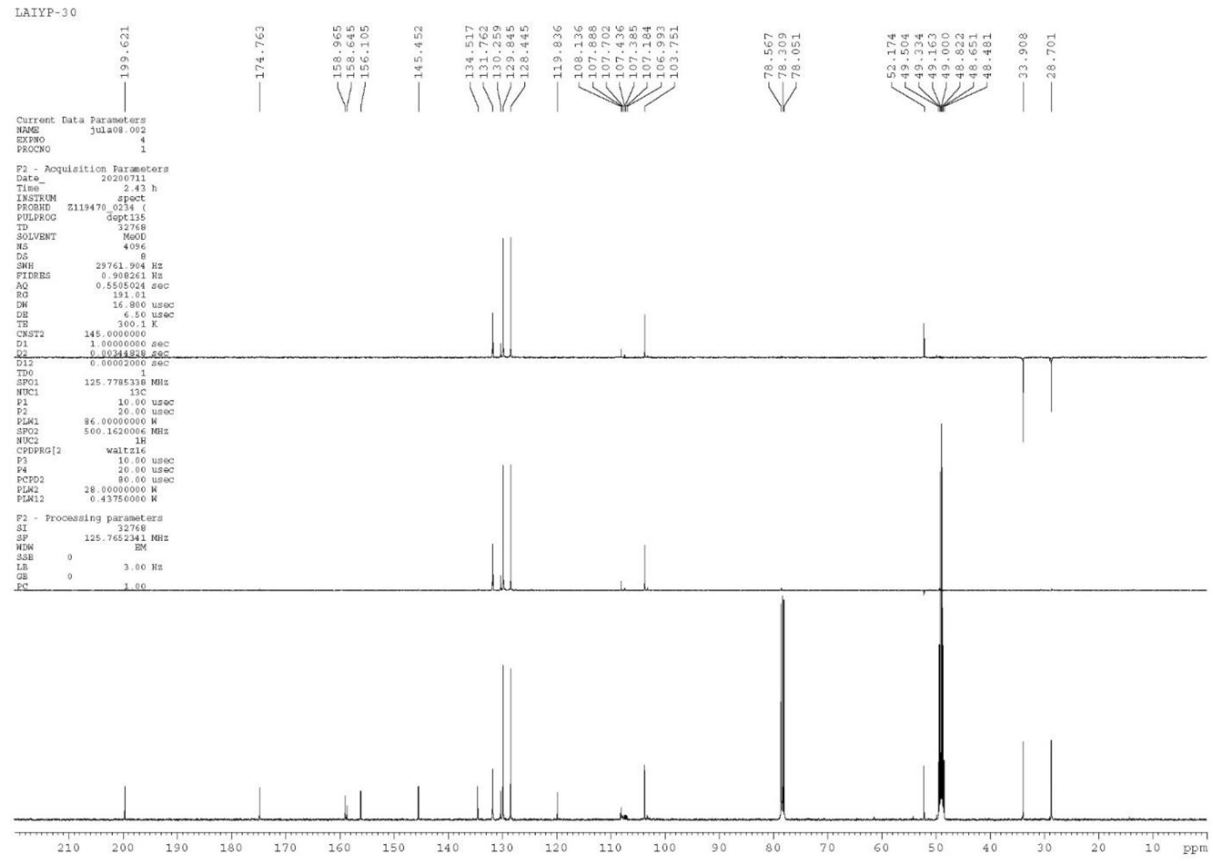

^{13}C-DEPT-135 NMR

5m

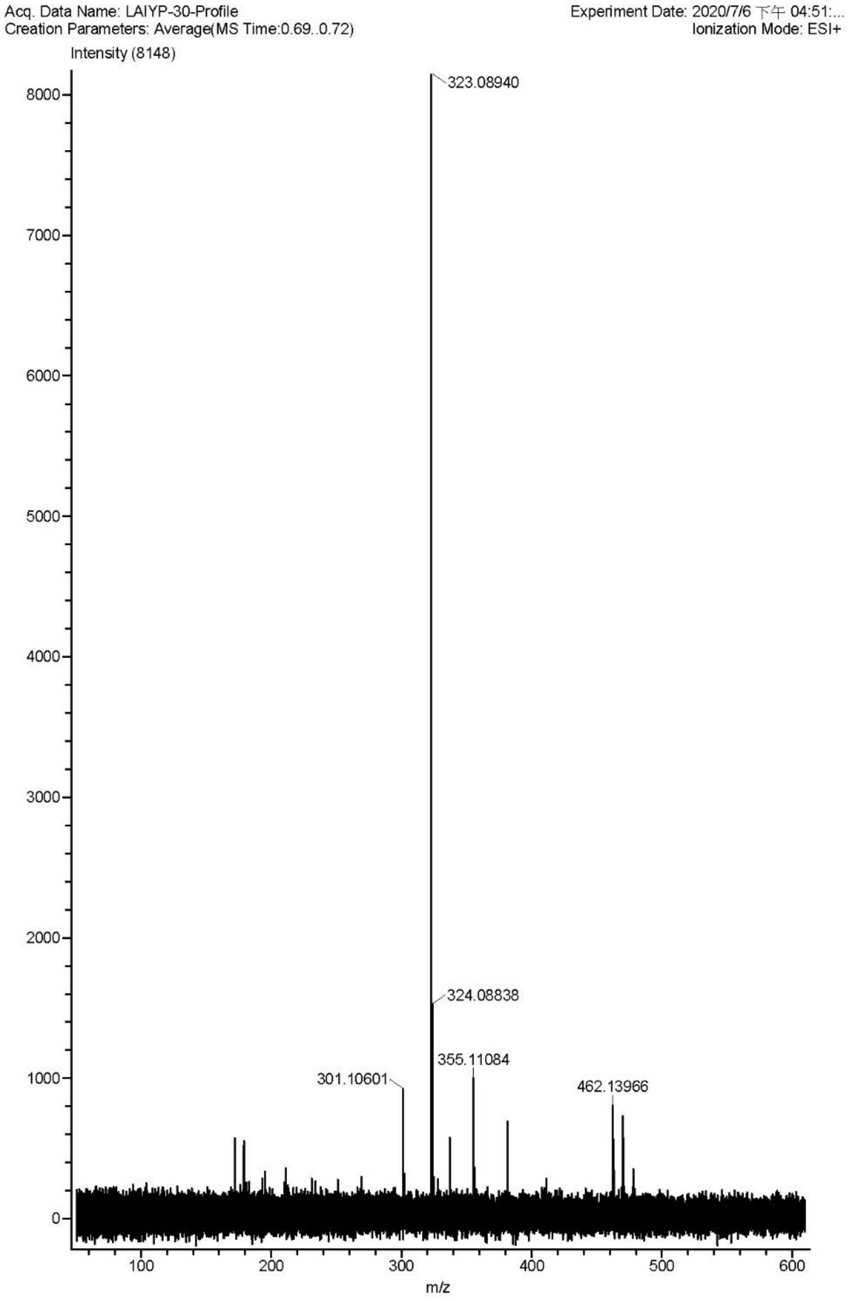

Acq. Data Name: LAIYP-30-Profile
Creation Parameters: Average(MS Time:0.69..0.72)
Experiment Date: 2020/7/6 下午 04:51:...
Ionization Mode: ESI+
Intensity (8148)
323.08940
324.08838
301.10601
355.11084
462.13966
m/z

HR ESI-MS

5m

2020.7.19 LAIYP-30
inject 0.4 mL 321 mg dilute with 15 mL MeOH/CH2Cl2=3
/97
MeOH/CH2Cl2 = 1/50 (500 mL)
semi-preparative column, 9.4 mm x 250 mm, Si-90

===

Injection Date : 6/26/2020 3:27:10 PM
Sample Name : LAIYP-30 Location : Vial 1
Acq. Operator : LAIYP
Acq. Instrument : Instrument 1
Method : C:\HPCHEM\1\METHODS\DUKE.M
Last changed : 6/12/2020 7:50:57 PM by LAIYP

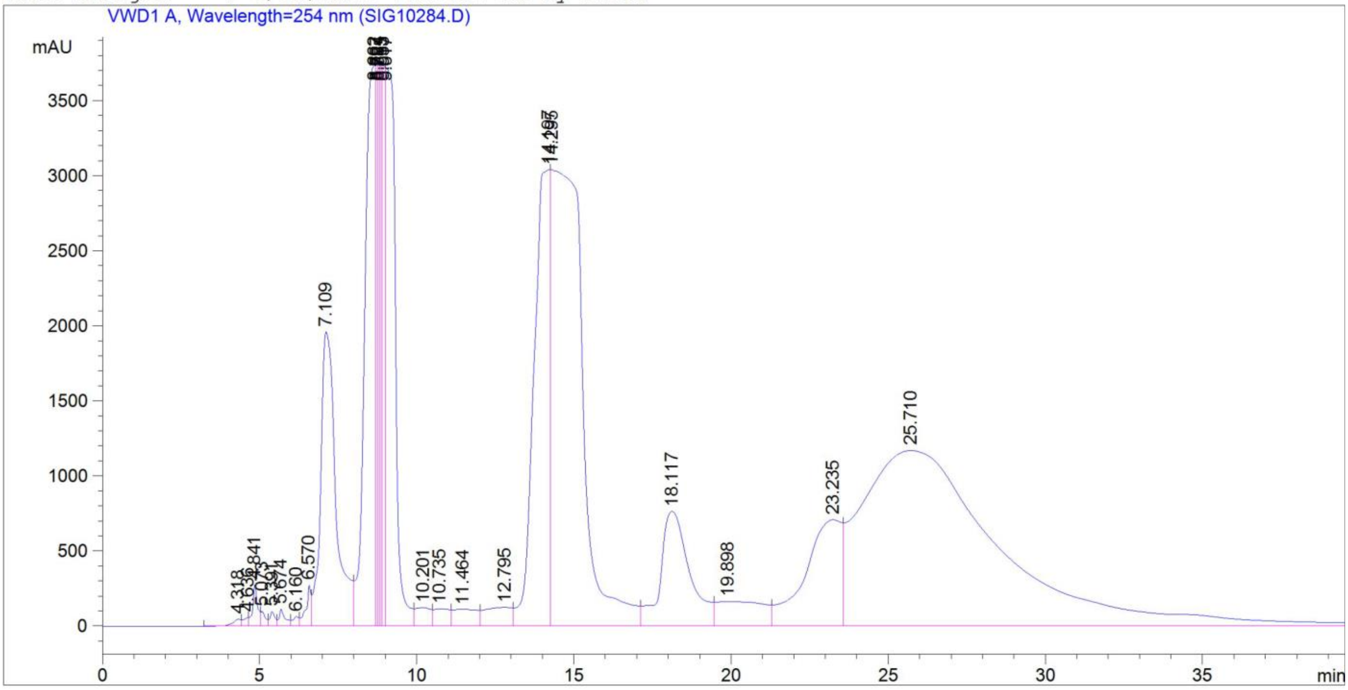

===

Area Percent Report

===

Sorted By : Signal
Multiplier : 1.0000
Dilution : 1.0000
Use Multiplier & Dilution Factor with ISTDs

Signal 1: VWD1 A, Wavelength=254 nm

Peak #	RetTime [min]	Type	Width [min]	Area mAU *s	Height [mAU]	Area %
1	4.318	BV	0.2733	874.43140	45.88009	0.0771
2	4.636	VV	0.1489	647.79034	58.76298	0.0571
3	4.841	VV	0.1589	3124.85132	274.77679	0.2754
4	5.073	VV	0.1429	1010.05475	98.52652	0.0890
5	5.391	VV	0.1583	1129.08984	97.58620	0.0995
6	5.674	VV	0.1873	1580.51111	112.40068	0.1393
7	6.160	VV	0.1843	883.45483	64.02430	0.0779
8	6.570	VV	0.1498	3132.06689	273.03619	0.2761
9	7.109	VV	0.5159	6.63627e4	1959.97607	5.8495
10	8.662	VV	0.2849	8.65105e4	3733.99536	7.6254
11	8.727	VV	0.0596	1.58523e4	3740.40820	1.3973
12	8.784	VV	0.0567	1.48832e4	3737.07349	1.3119

HPLC chromatogram of the unseparated **5m** from flash chromatography

2020.7.18 LAIYP-30
inject 20 uL 19 mg dilute with 1.5 mL MeOH/CH2Cl2=3/97
MeOH/CH2Cl2 = 1.5/98.5 (500 mL)
semi-preparative column, 9.4 mm x 250 mm, Si-90

===

Injection Date : 6/25/2020 7:11:50 PM
Sample Name : LAIYP-30 Location : Vial 1
Acq. Operator : LAIYP
Acq. Instrument : Instrument 1
Method : C:\HPCHEM\1\METHODS\DUKE.M
Last changed : 6/12/2020 7:50:57 PM by LAIYP

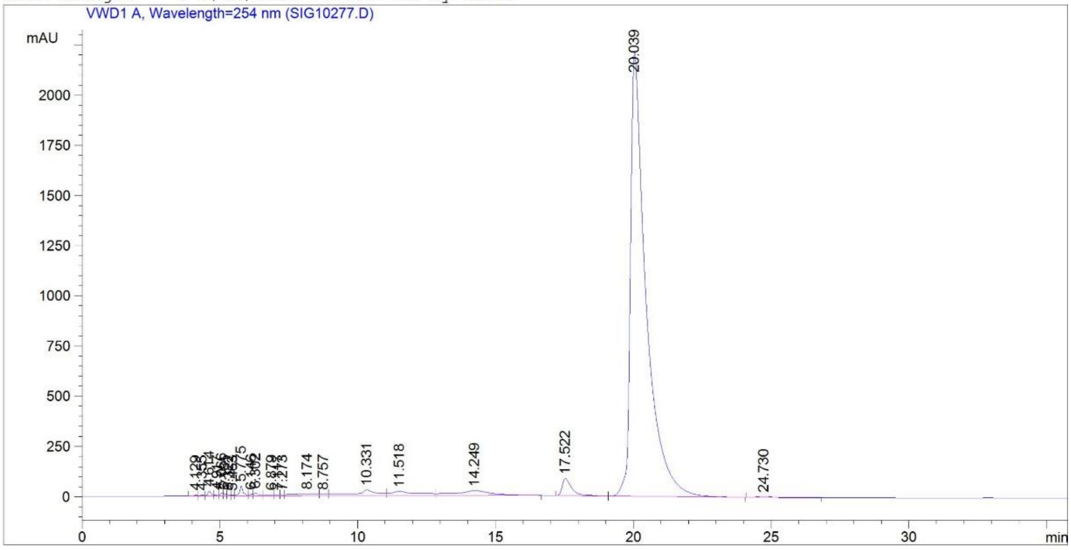

===

Area Percent Report

===

Sorted By : Signal
Multiplier : 1.0000
Dilution : 1.0000
Use Multiplier & Dilution Factor with ISTDs

Signal 1: VWD1 A, Wavelength=254 nm

Peak #	RetTime [min]	Type	Width [min]	Area mAU *s	Height [mAU]	Area %
1	4.129	VV	0.1511	63.02722	6.10127	0.0648
2	4.355	VV	0.1572	63.02929	5.87361	0.0648
3	4.614	VV	0.1302	225.26292	24.92461	0.2314
4	4.917	VV	0.1265	77.19509	8.26216	0.0793
5	5.066	VV	0.0918	106.79453	17.09851	0.1097
6	5.181	VV	0.0911	68.79041	10.89378	0.0707
7	5.322	VV	0.1030	56.74982	8.01628	0.0583
8	5.463	VV	0.0963	37.04968	5.58883	0.0381
9	5.775	VV	0.1404	508.55206	50.64421	0.5225
10	6.146	VV	0.1193	78.52660	9.40108	0.0807
11	6.302	VB	0.1670	199.29010	16.51520	0.2048
12	6.879	BV	0.2873	160.12352	7.15572	0.1645

Reinjection of the isolated fraction into HPLC for confirmation of the purity

5m

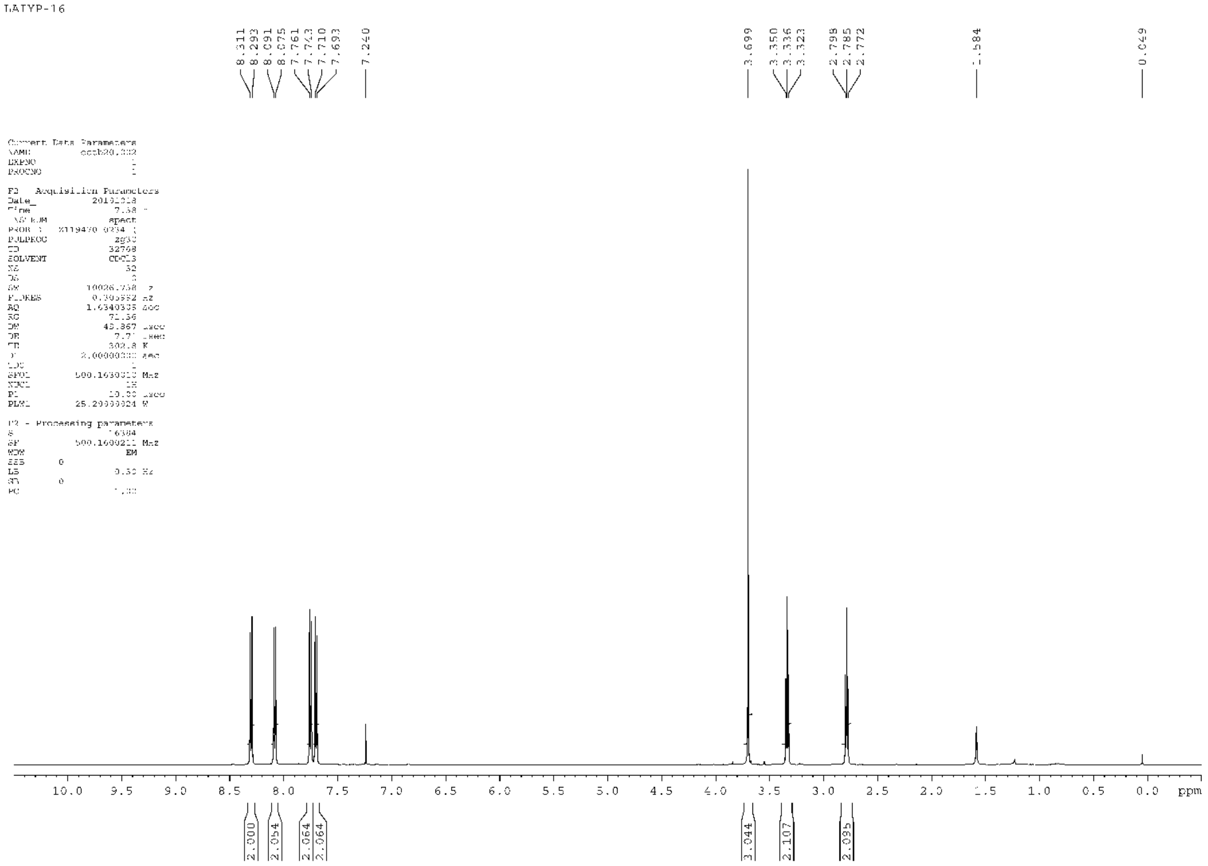
8.311
8.293
8.091
8.075
7.761
7.743
7.710
7.693
7.240
3.699
3.350
3.336
3.323
2.798
2.785
2.772
1.584
0.049
10.0 9.5 9.0 8.5 8.0 7.5 7.0 6.5 6.0 5.5 5.0 4.5 4.0 3.5 3.0 2.5 2.0 1.5 1.0 0.5 0.0 ppm
2.000
2.054
2.064
2.064
3.044
2.107
2.095

^{1}H NMR

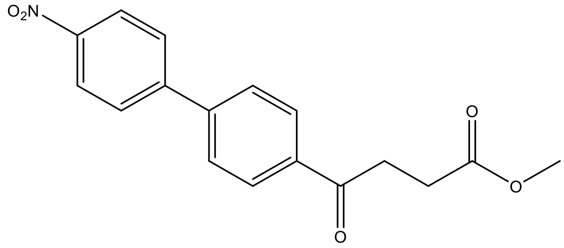
O2N
O
O
O

5n

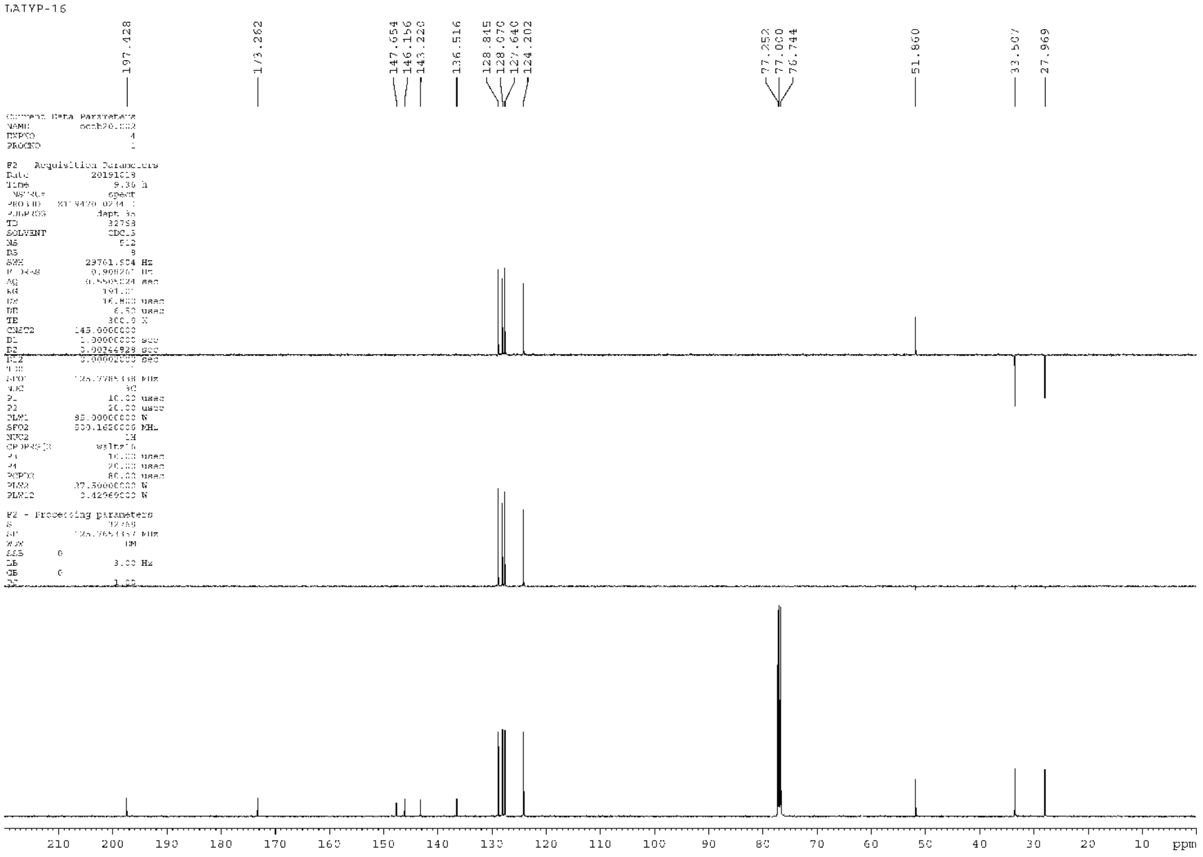

^{13}C-DEPT-135 NMR

5n

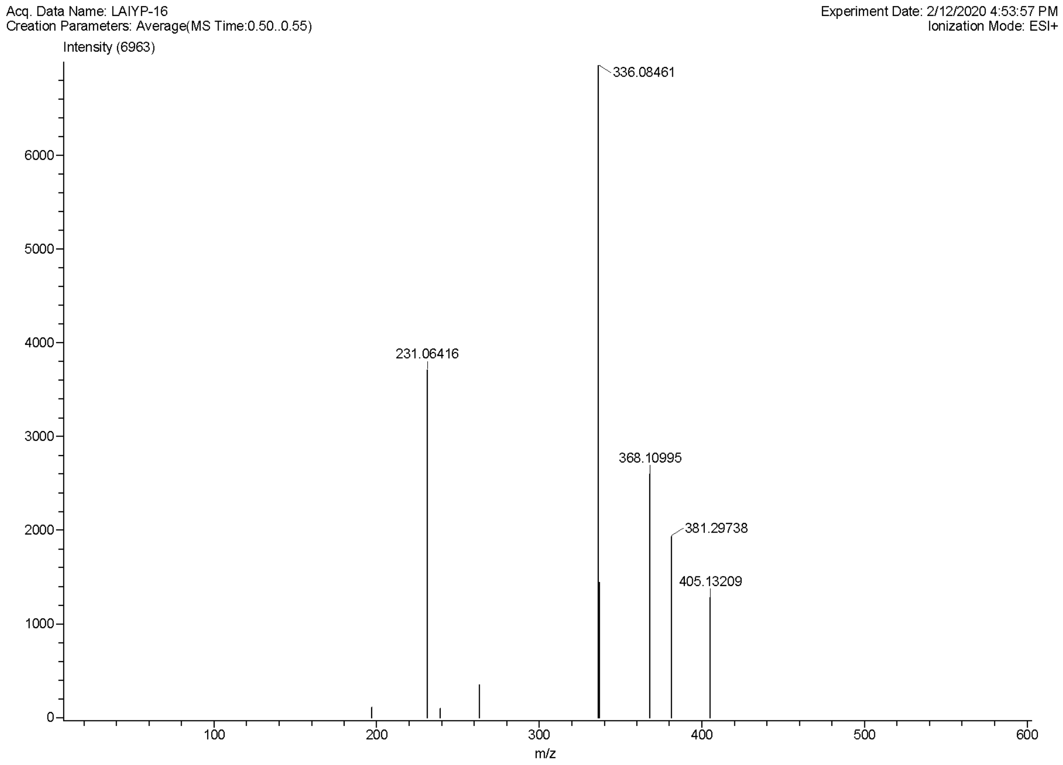
Acq. Data Name: LAIYP-16
Creation Parameters: Average(MS Time:0.50..0.55)
Experiment Date: 2/12/2020 4:53:57 PM
Ionization Mode: ESI+
Intensity (6963)
336.08461
231.06416
368.10995
381.29738
405.13209
6000
5000
4000
3000
2000
1000
0
100
200
300
400
500
600
m/z

HR ESI-MS

5n

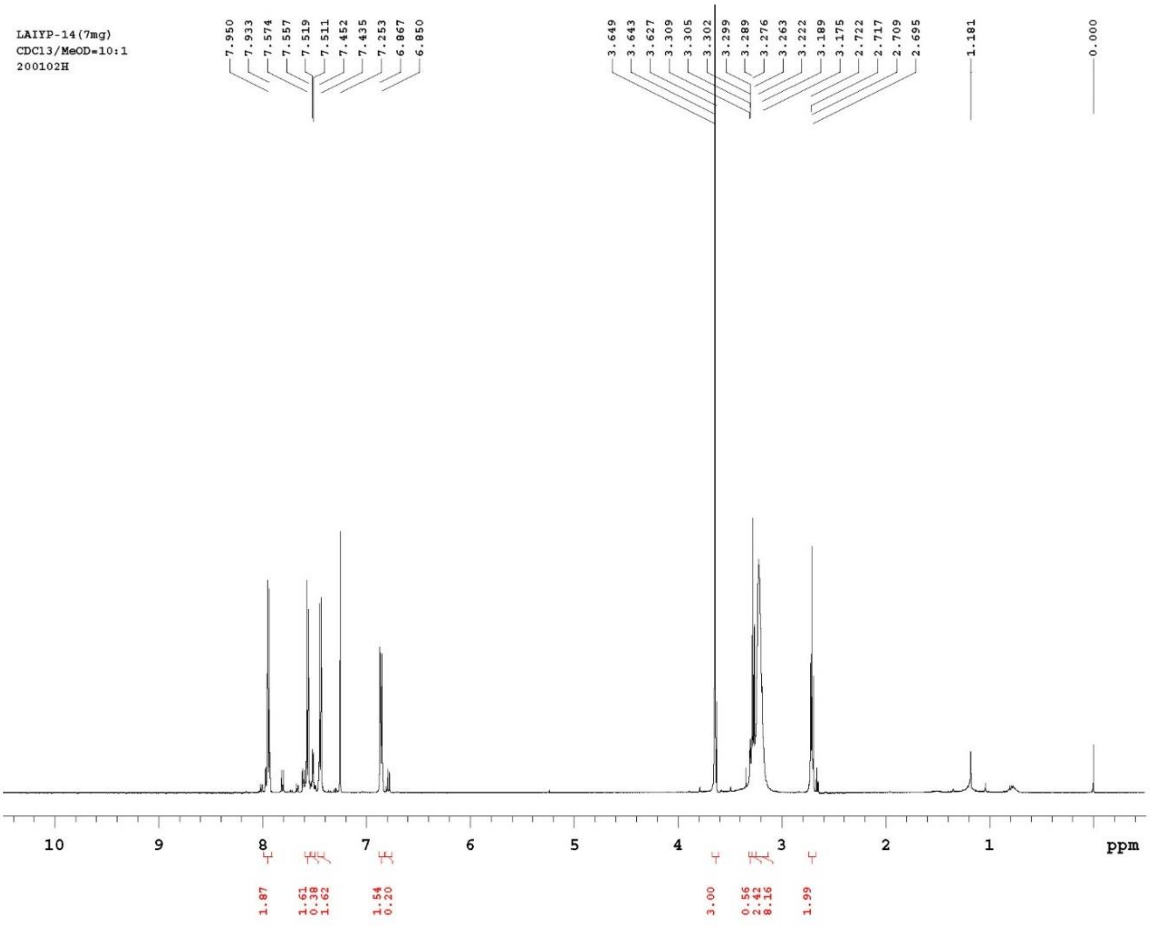
LAIYP-14(7mg)
CDCl3/MeOD=10:1
200102H
7.950
7.933
7.574
7.557
7.519
7.511
7.452
7.435
7.253
6.867
6.850
3.649
3.643
3.627
3.309
3.305
3.302
3.299
3.289
3.276
3.263
3.222
3.189
3.175
2.722
2.717
2.709
2.695
1.181
0.000
10
9
8
7
6
5
4
3
2
1
ppm
1.87
1.61
0.38
1.62
1.54
0.20
3.00
0.56
2.42
8.16
1.99

^{1}H NMR

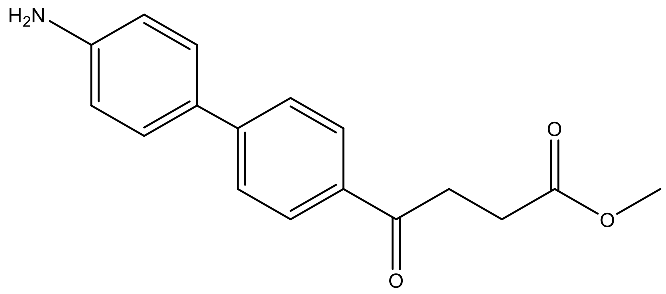

5o

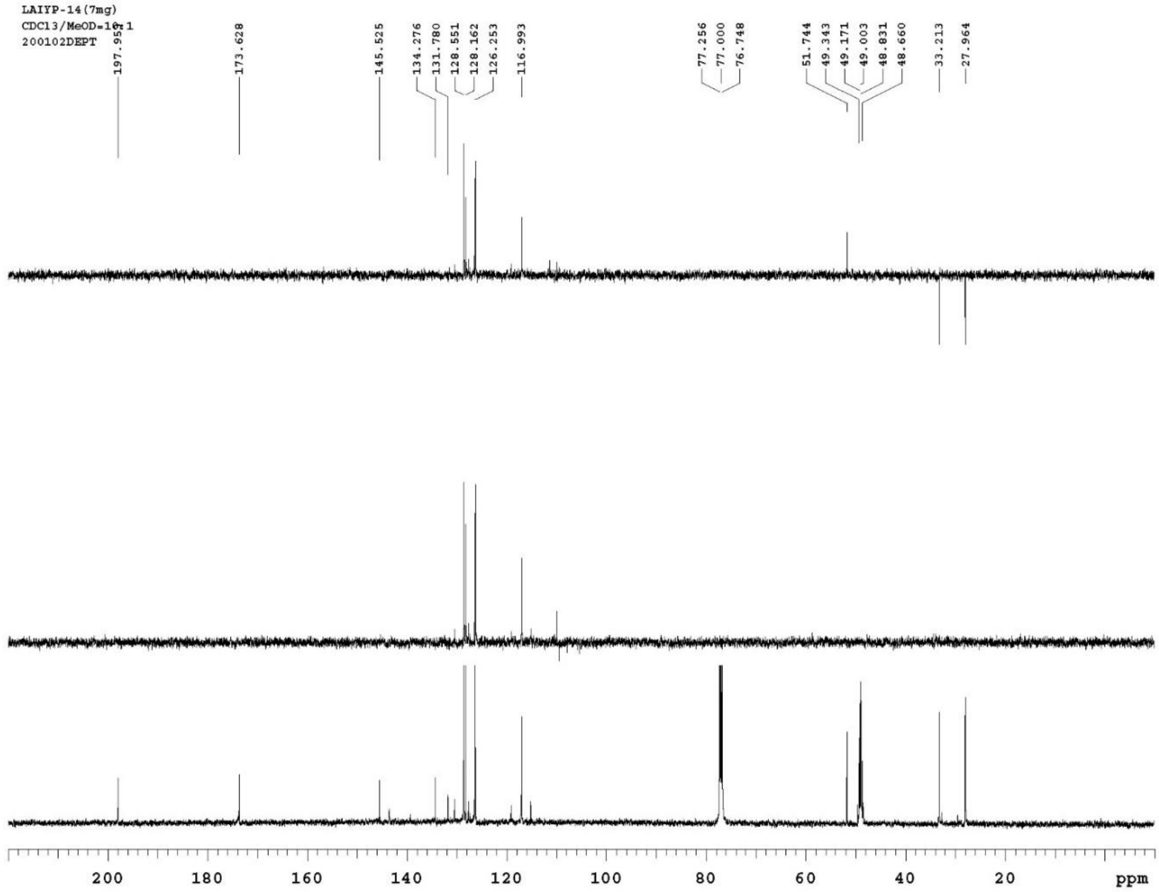

^{13}C-DEPT-135 NMR

5o

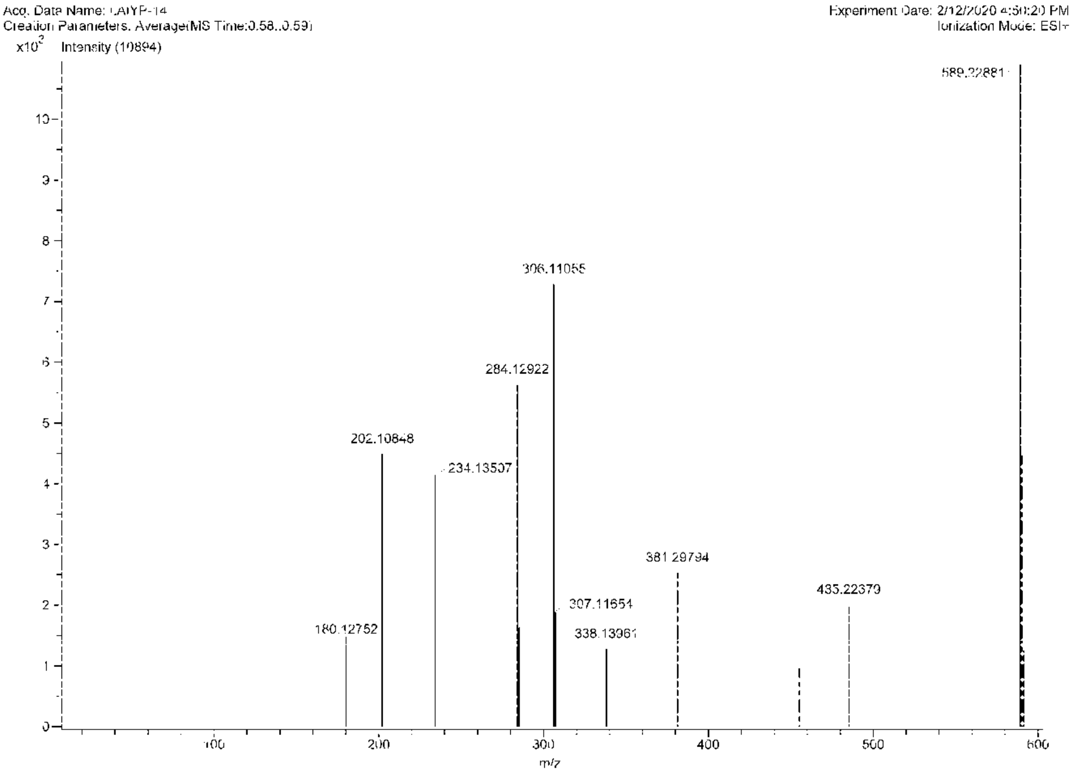

Experiment Date: 2/12/2020 4:50:20 PM
Ionization Mode: ESI+
Intensity (10894)
589.22881
306.11055
284.12922
202.10848
234.13507
381.29794
435.22379
307.11654
338.13961
180.12752
m/z

HR ESI-MS

5o

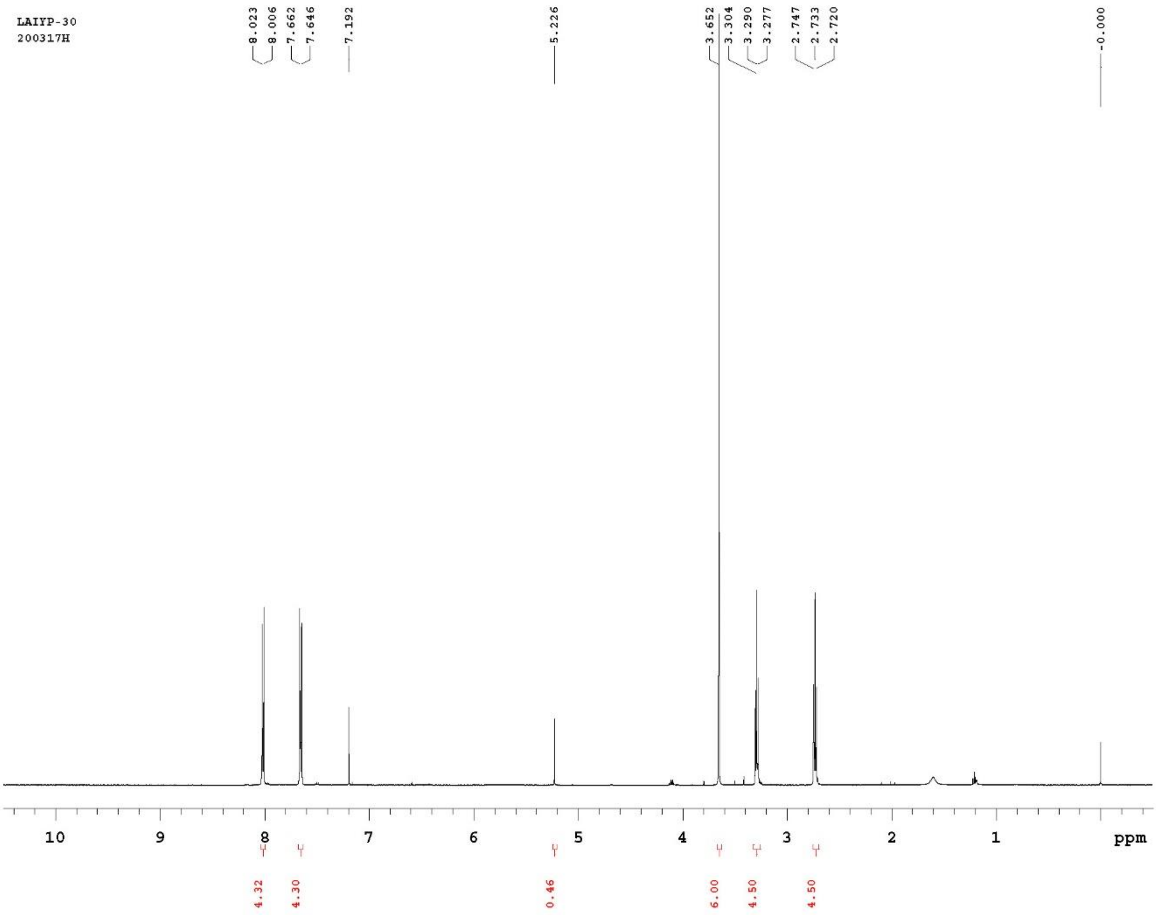

^{1}H NMR

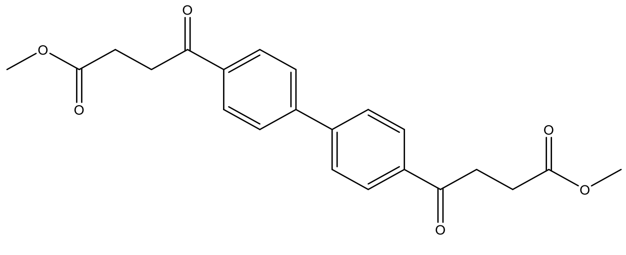

Homocoupling by-product

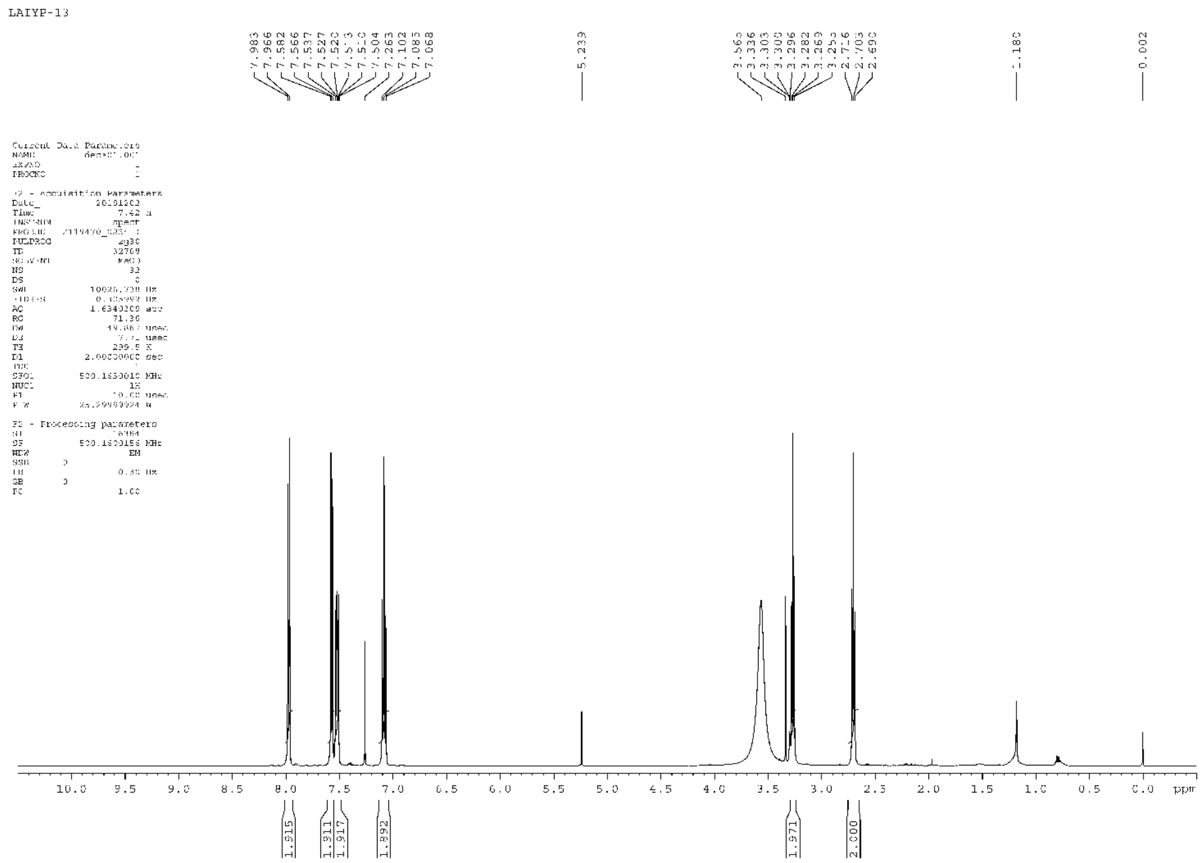

^{1}H NMR

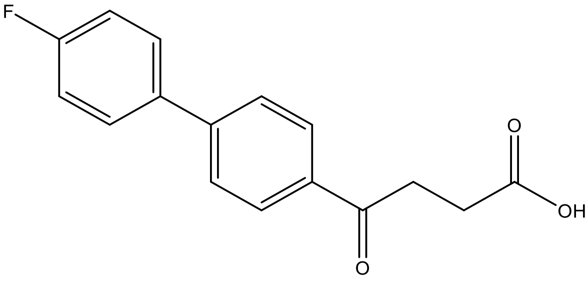

6a

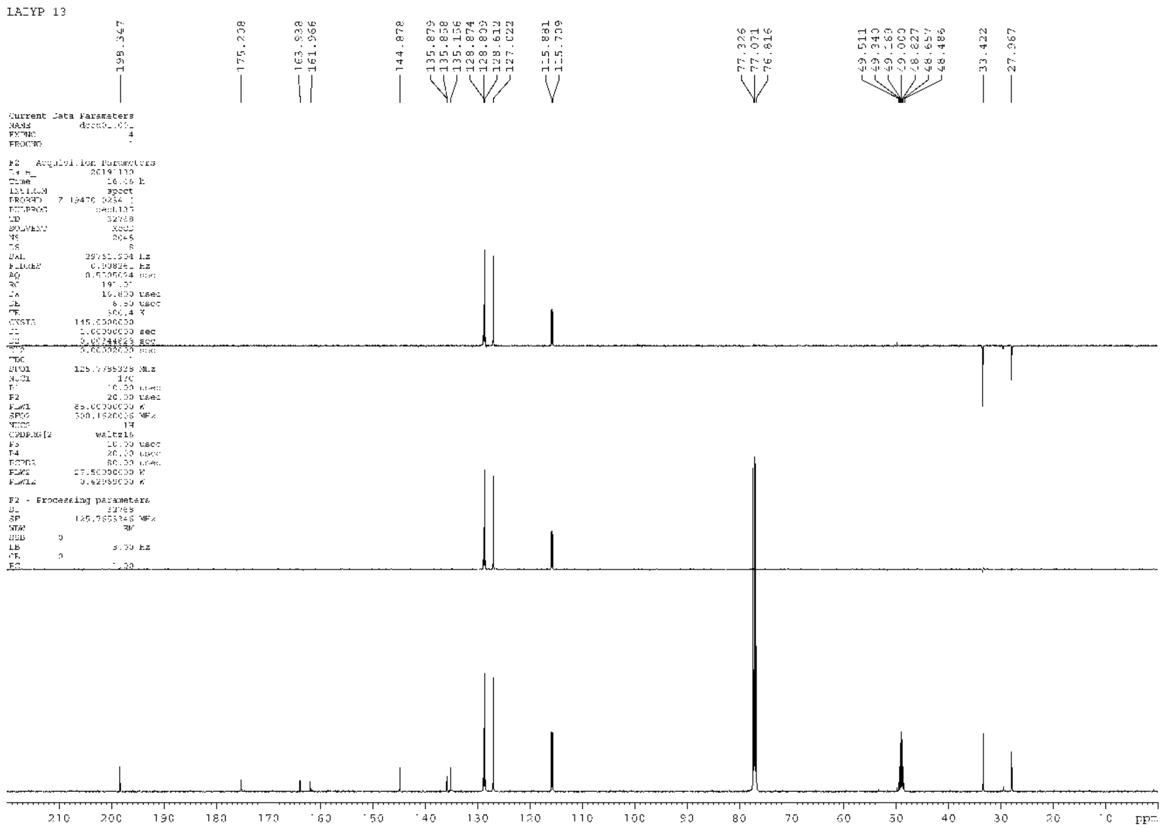

^{13}C-DEPT-135 NMR

6a

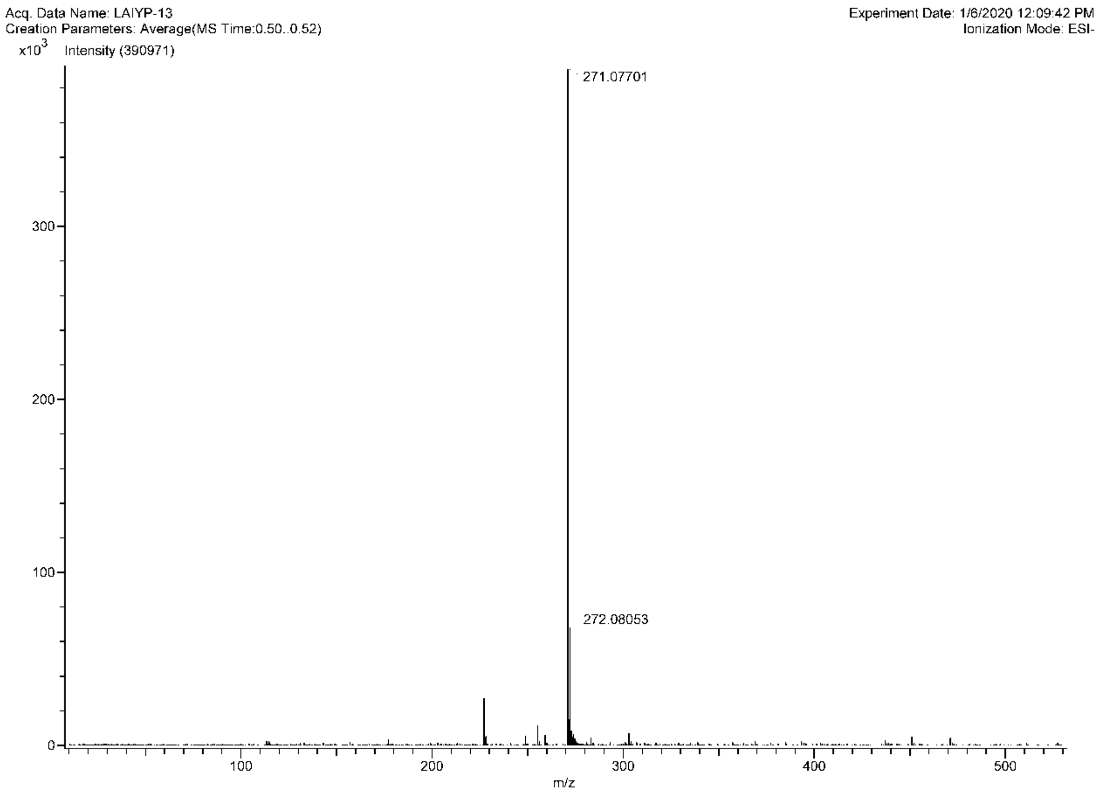

Acq. Data Name: LAIYP-13
Creation Parameters: Average(MS Time:0.50..0.52)
Experiment Date: 1/6/2020 12:09:42 PM
Ionization Mode: ESI-
x10^3 Intensity (390971)
271.07701
272.08053
300
200
100
0
100
200
300
400
500
m/z

HR ESI-MS

6a

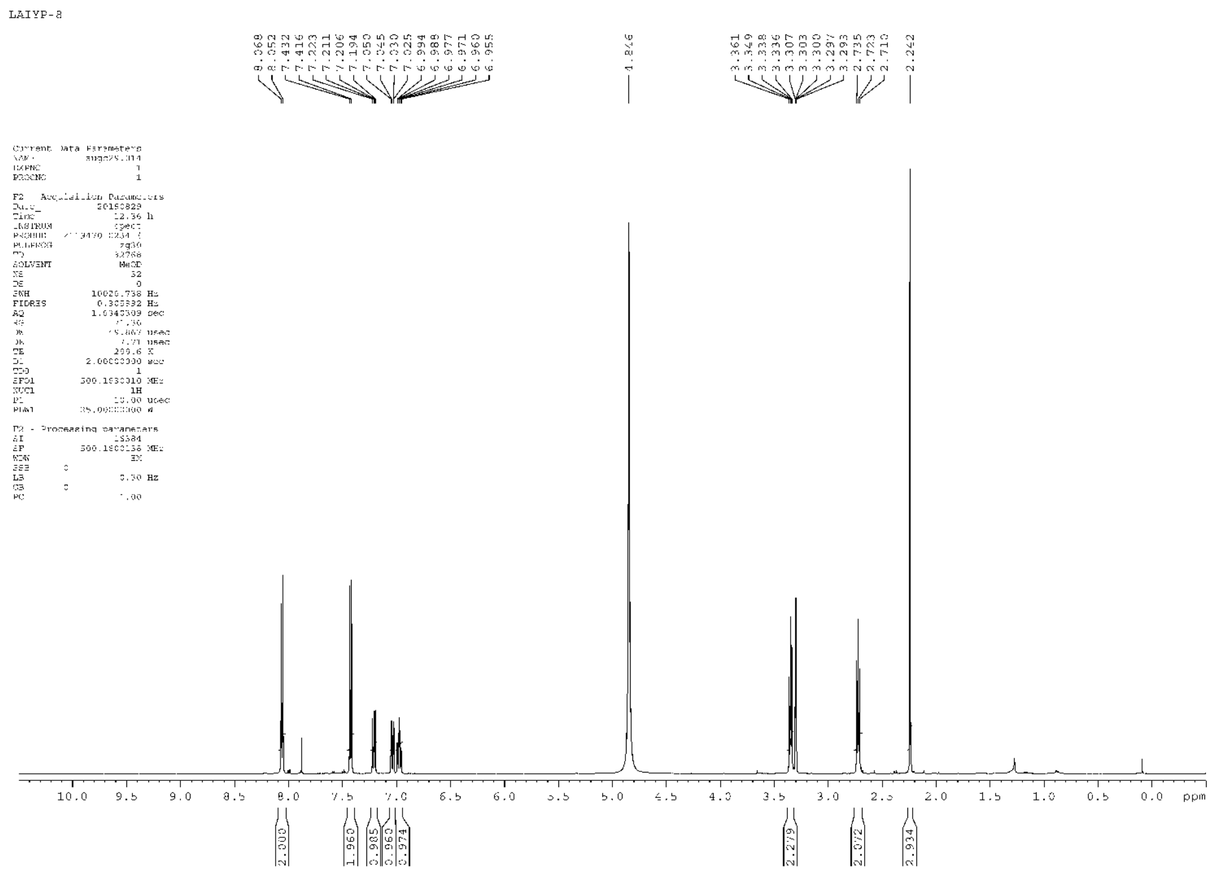

^{1}H NMR

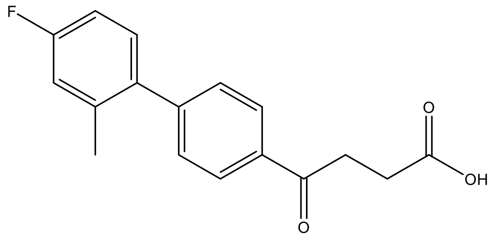

6b

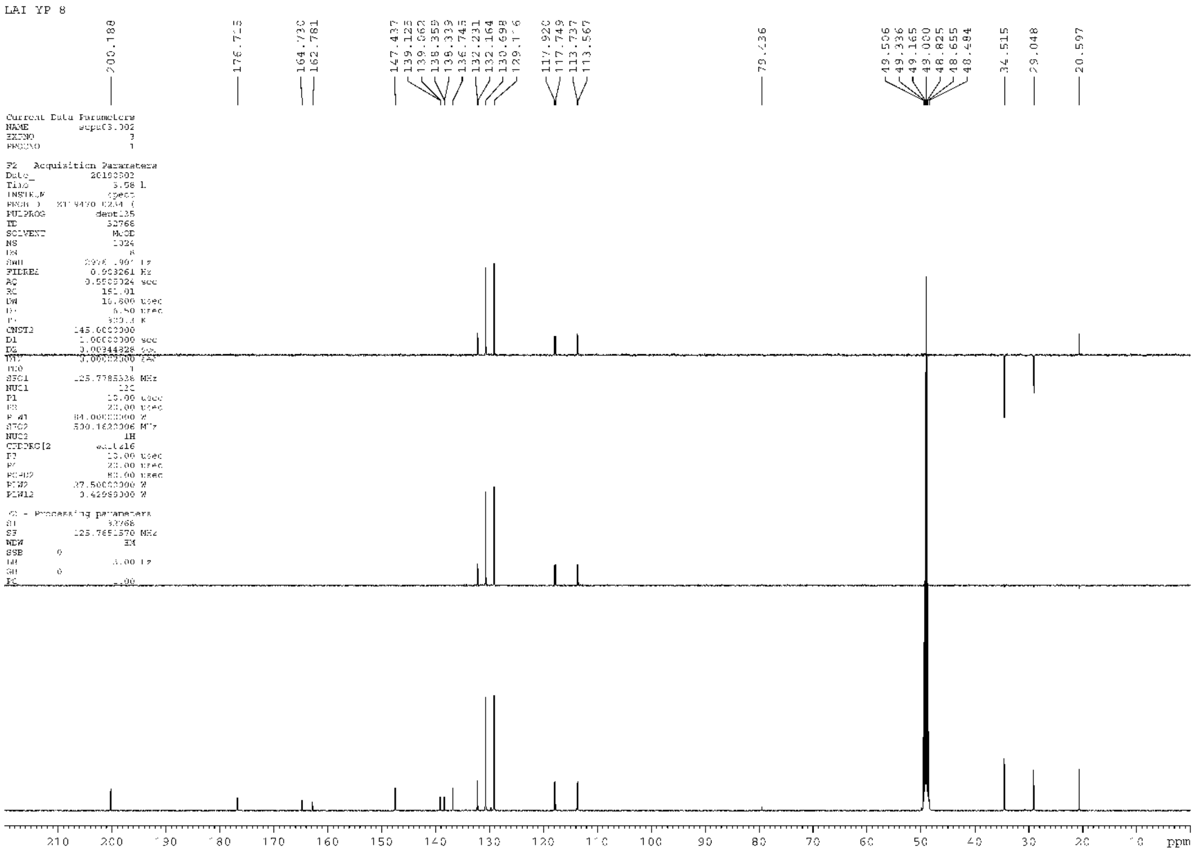

^{13}C-DEPT-135 NMR

6b

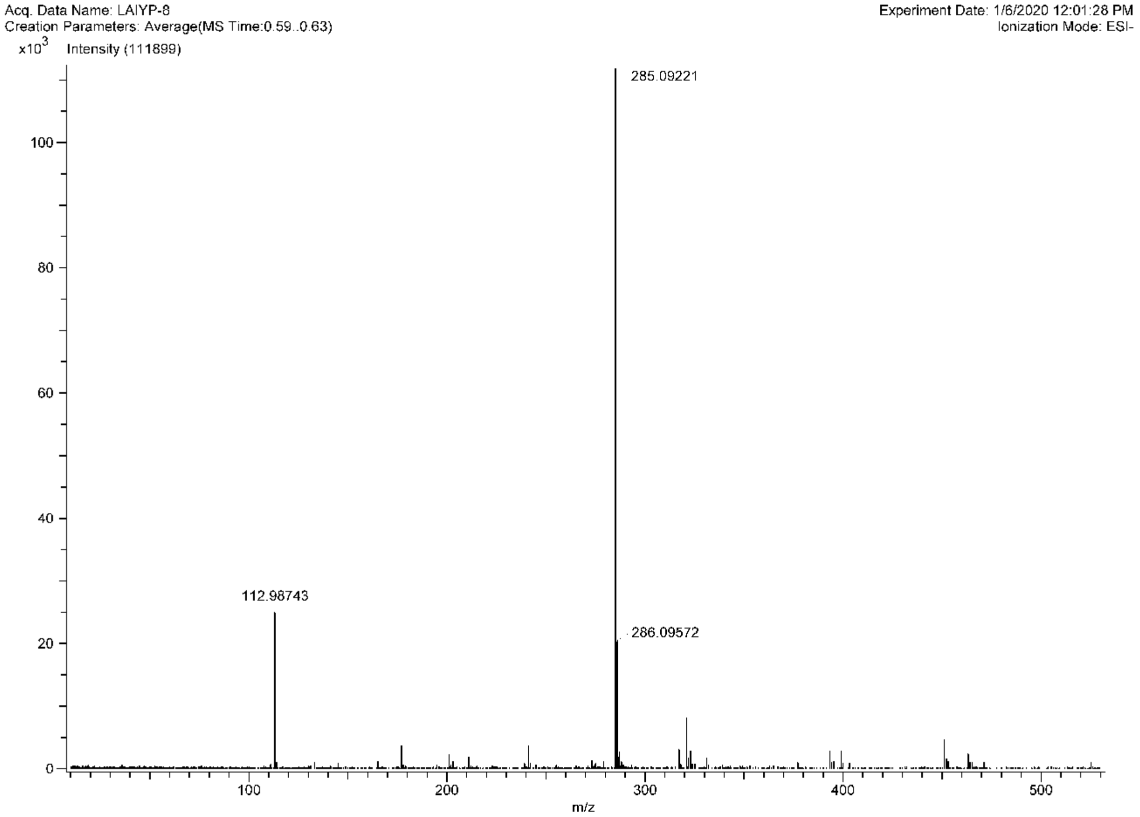

Acq. Data Name: LAIYP-8
Creation Parameters: Average(MS Time:0.59..0.63)
Experiment Date: 1/6/2020 12:01:28 PM
Ionization Mode: ESI-
x10^3 Intensity (111899)
285.09221
112.98743
286.09572
m/z

HR ESI-MS

6b

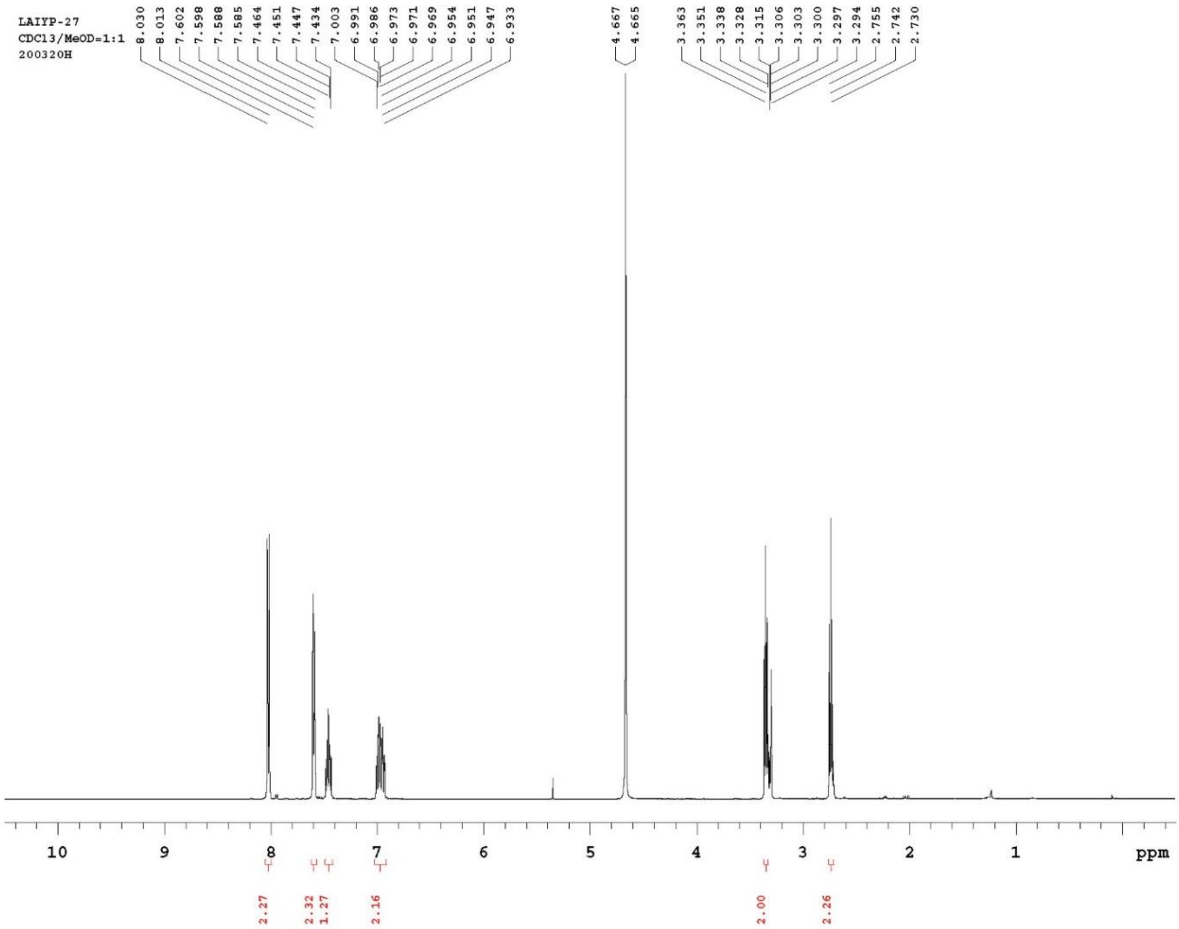

LAIYP-27
CDCl3/MeOD=1:1
200320H
8.030
8.013
7.602
7.598
7.588
7.585
7.464
7.451
7.447
7.434
7.003
6.991
6.986
6.973
6.971
6.969
6.954
6.951
6.947
6.933
4.667
4.665
3.363
3.351
3.338
3.328
3.315
3.306
3.303
3.300
3.297
3.294
2.755
2.742
2.730
10
9
8
7
6
5
4
3
2
1
ppm
2.27
2.32
1.27
2.16
2.00
2.26

^{1}H NMR

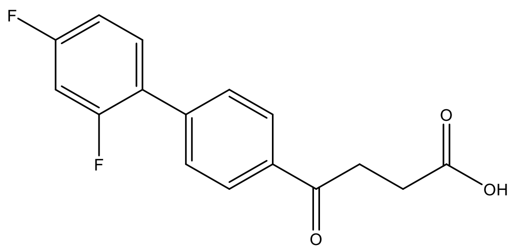

F
F
O
OH
O

6c

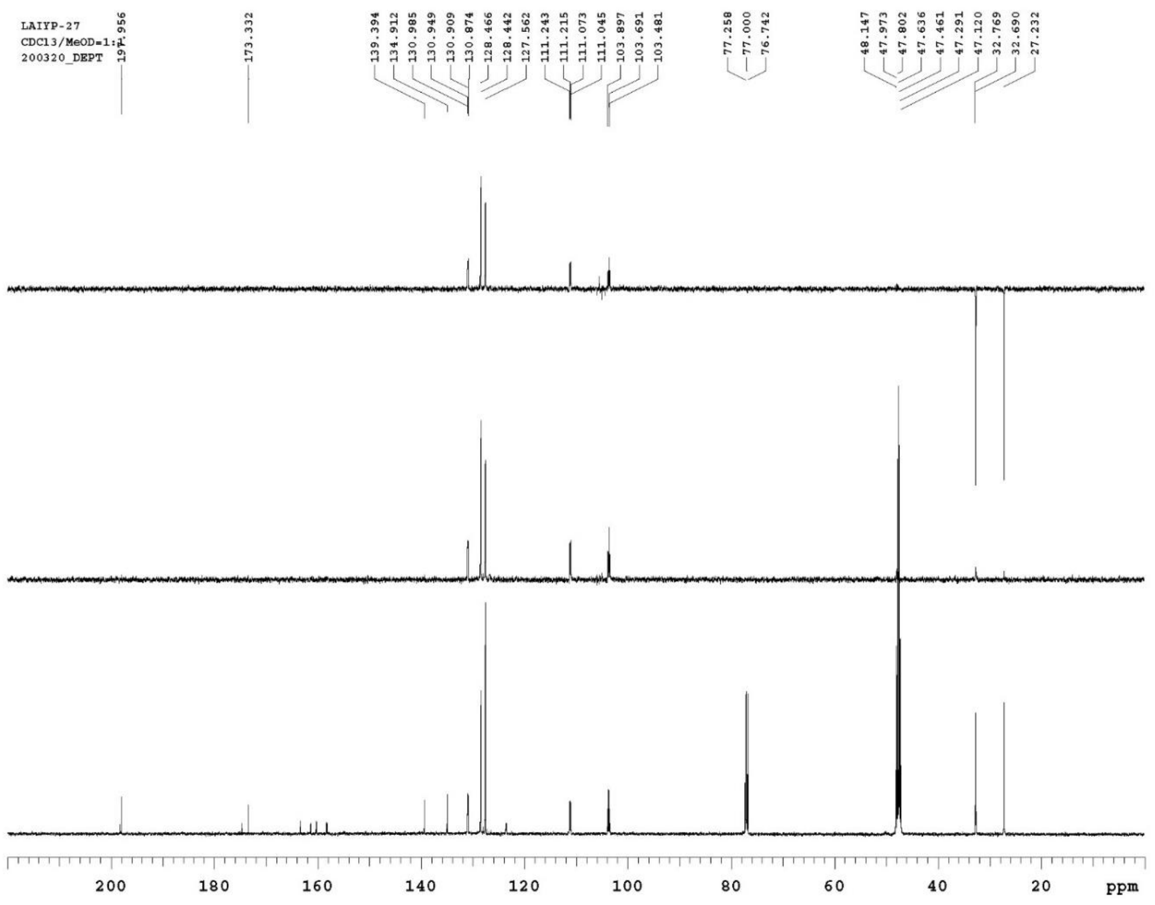

^{13}C-DEPT-135 NMR

6c

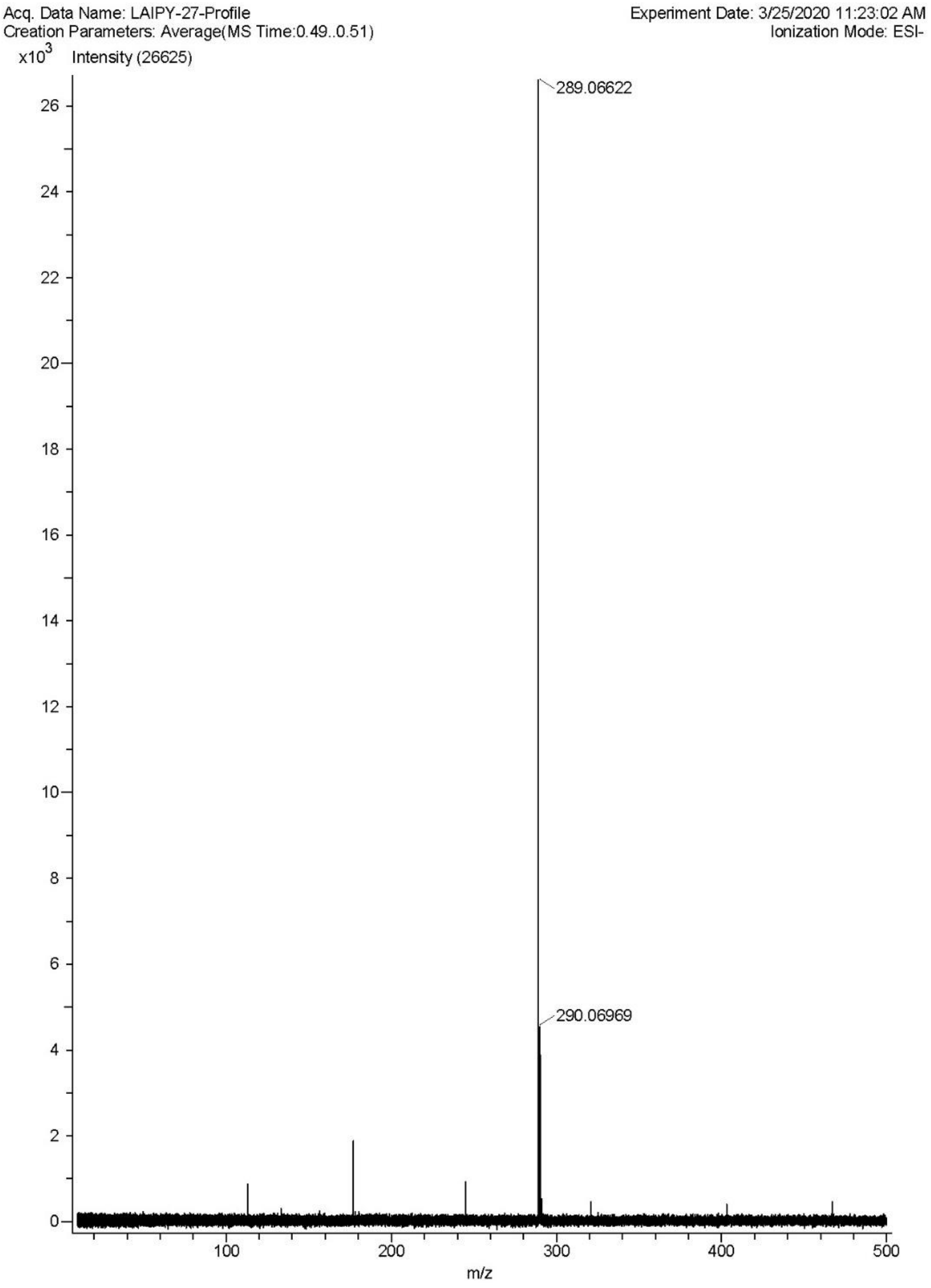

Acq. Data Name: LAIPY-27-Profile
Creation Parameters: Average(MS Time:0.49..0.51)
Experiment Date: 3/25/2020 11:23:02 AM
Ionization Mode: ESI-
x10^3 Intensity (26625)
289.06622
290.06969
m/z

HR ESI-MS

6c

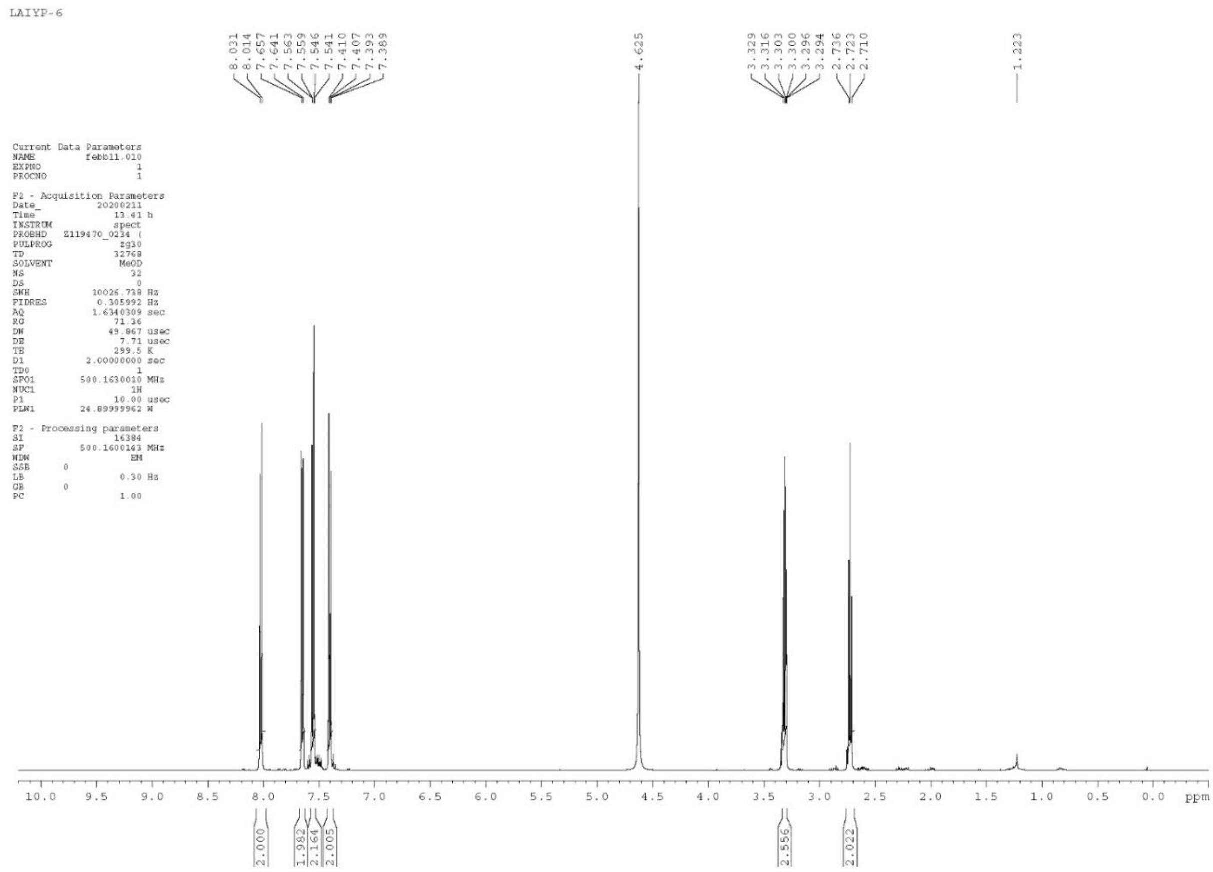

^{1}H NMR

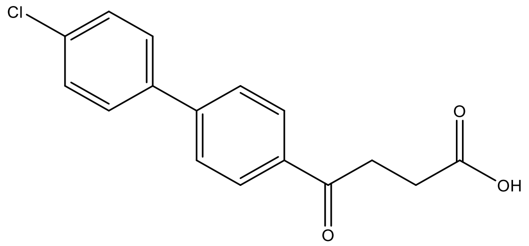

6d

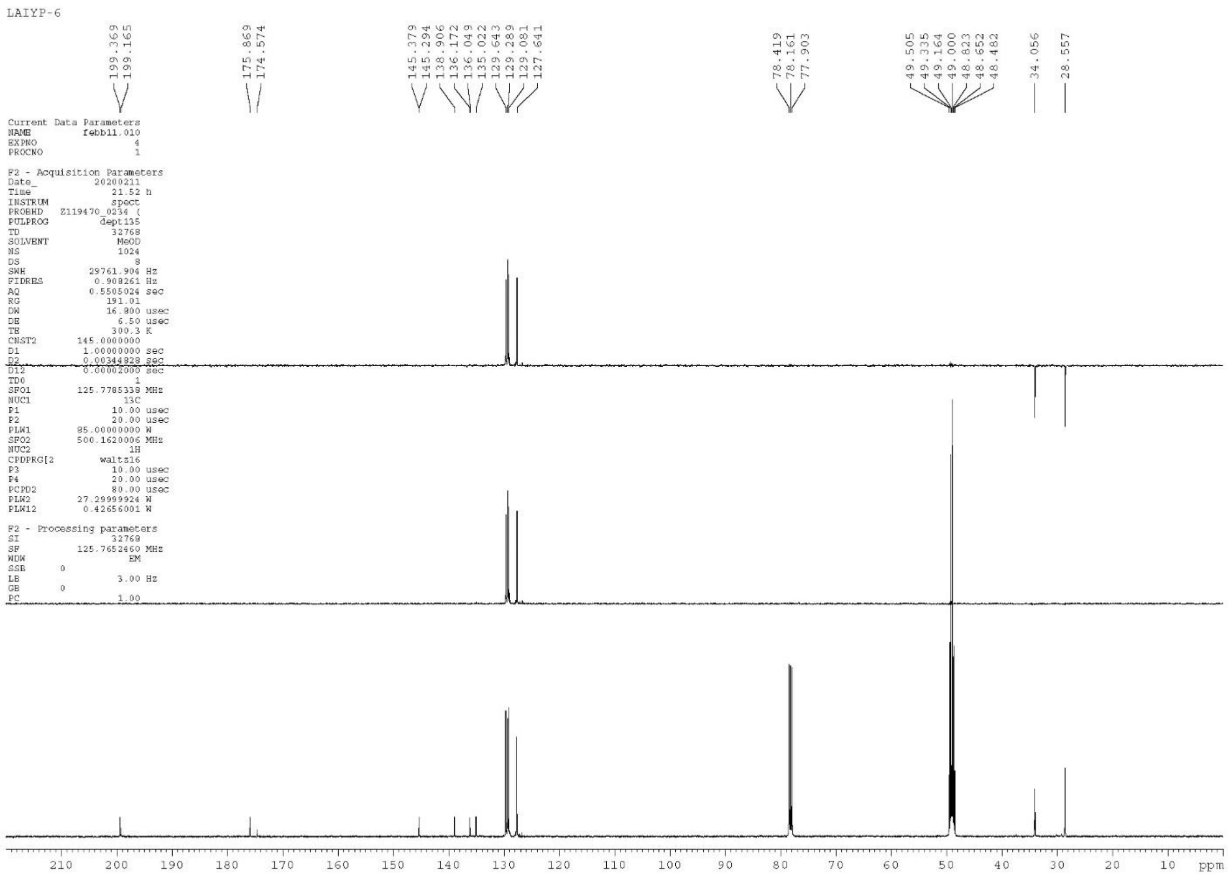

^{13}C-DEPT-135 NMR

6d

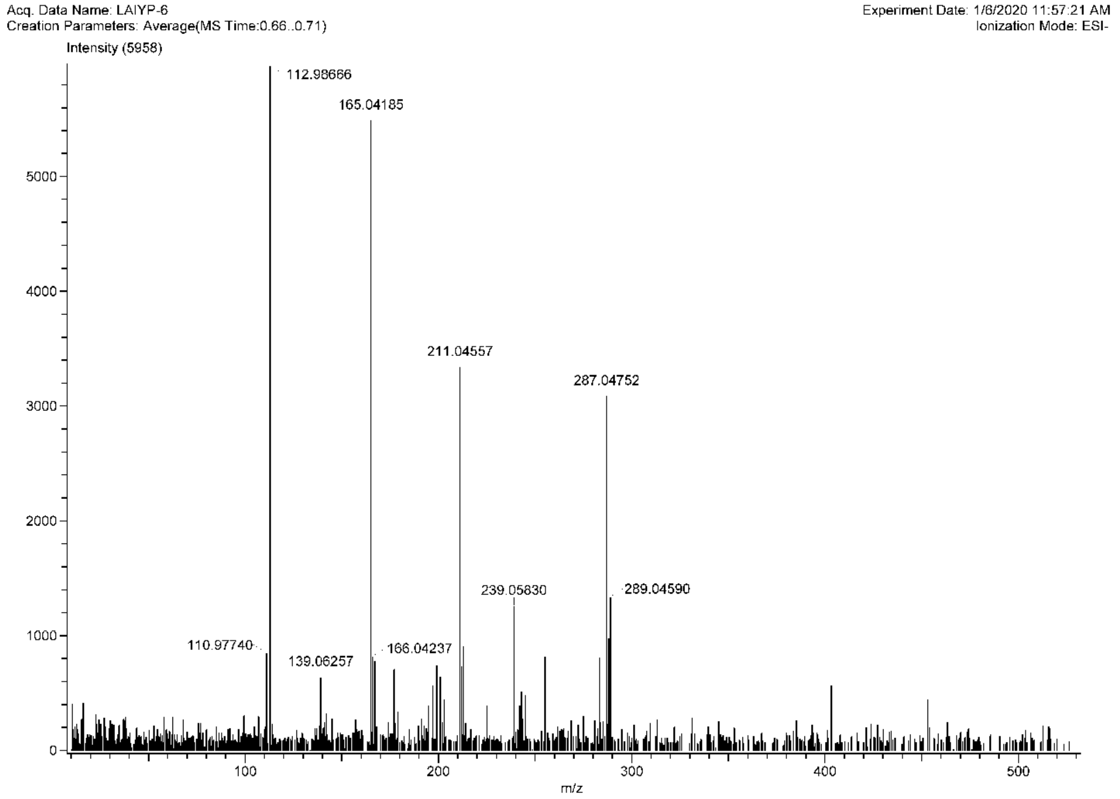

Acq. Data Name: LAIYP-6
Creation Parameters: Average(MS Time:0.66..0.71)
Experiment Date: 1/6/2020 11:57:21 AM
Ionization Mode: ESI-
Intensity (5958)
112.98666
165.04185
211.04557
287.04752
239.05830
289.04590
110.97740
139.06257
166.04237
m/z

HR ESI-MS

6d

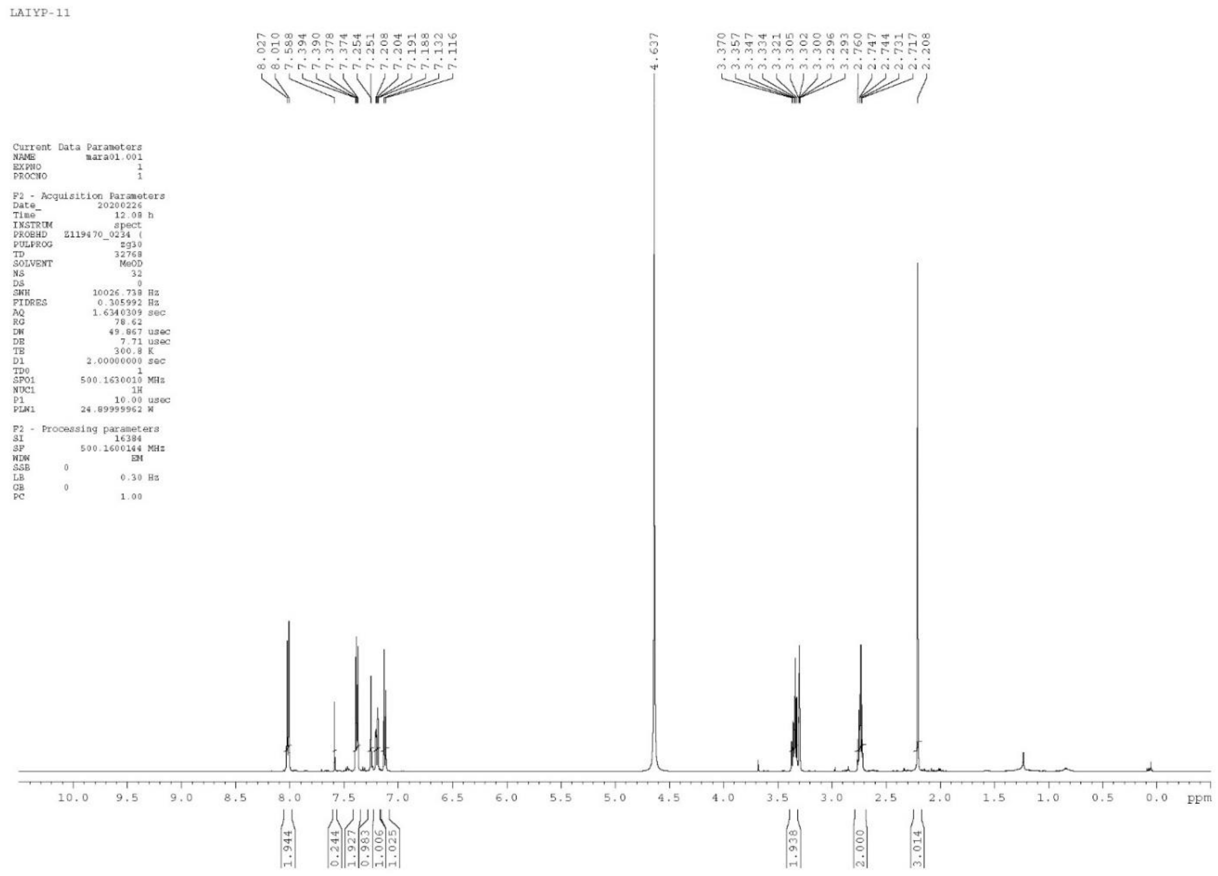

^{1}H NMR

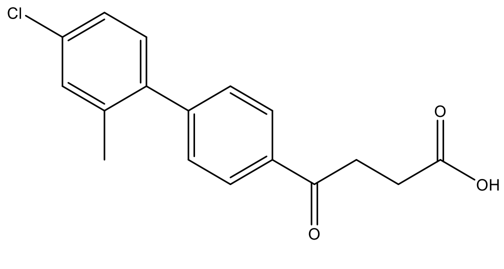

6e

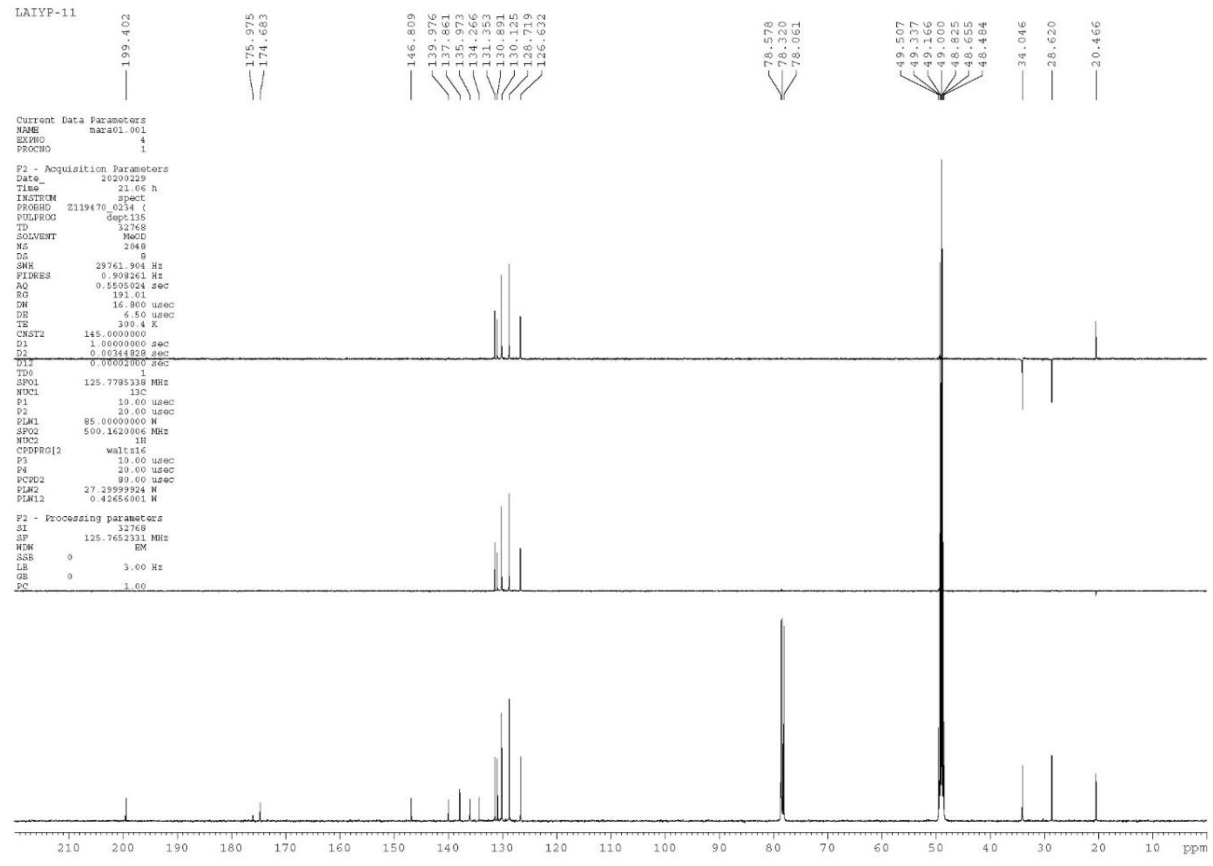

^{13}C-DEPT-135 NMR

6e

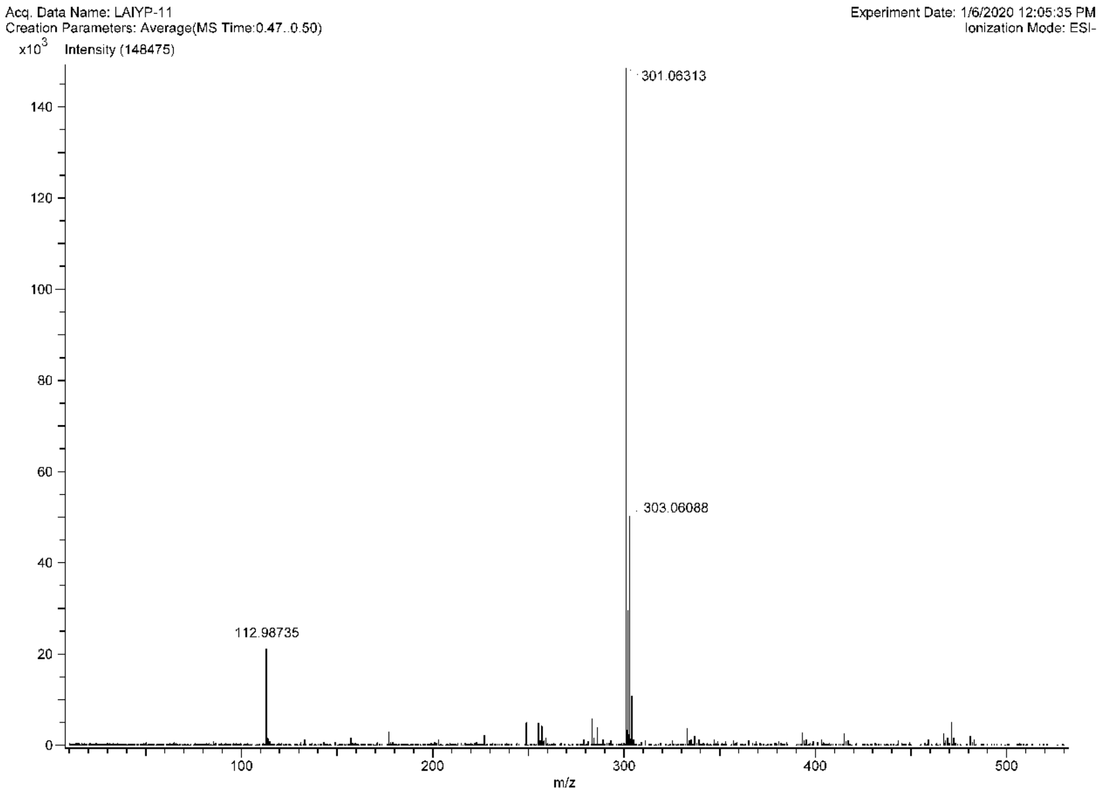

Acq. Data Name: LAIYP-11
Creation Parameters: Average(MS Time:0.47..0.50)
Experiment Date: 1/6/2020 12:05:35 PM
Ionization Mode: ESI-
x10^3 Intensity (148475)
301.06313
303.06088
112.98735
m/z

HR ESI-MS

6e

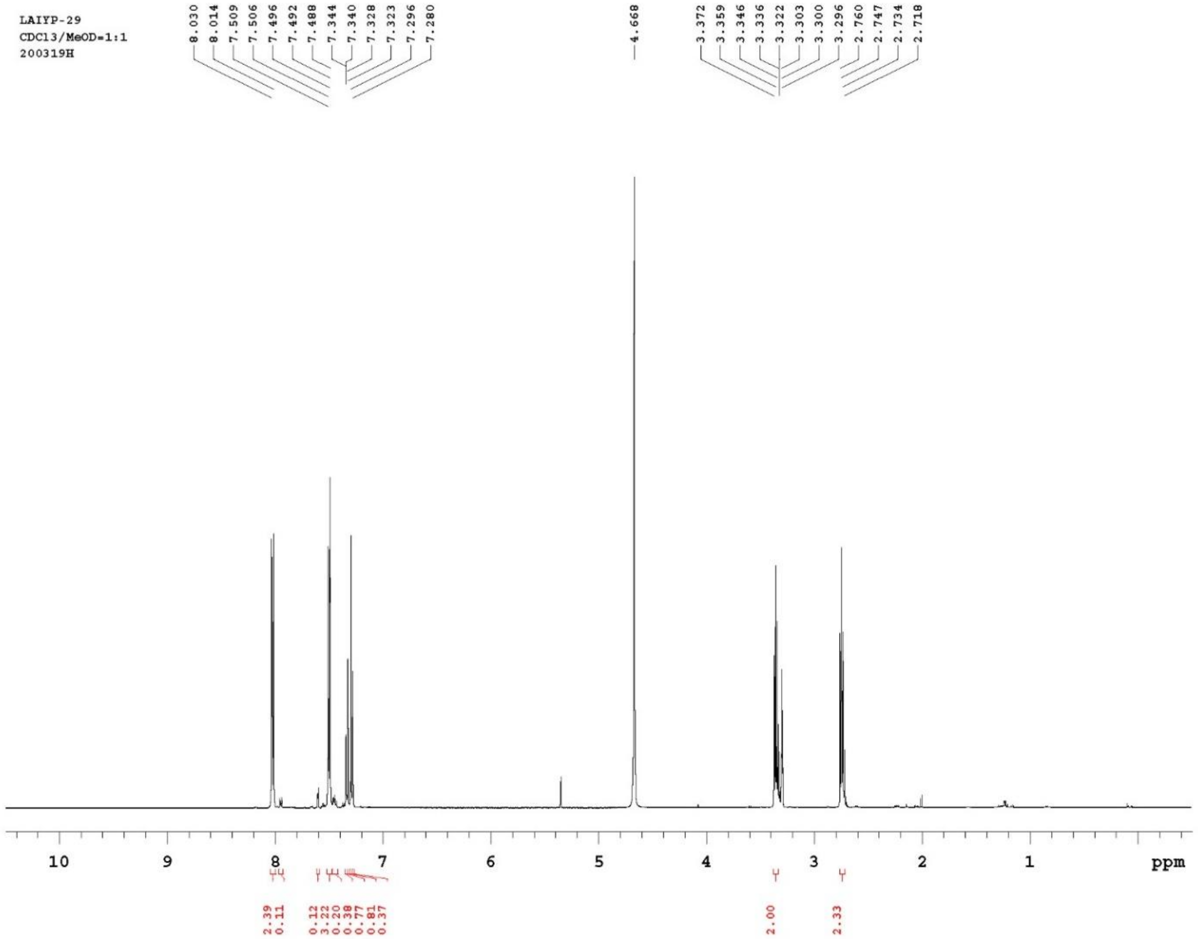
LAIYP-29
CDCl3/MeOD=1:1
200319H
8.030
8.014
7.509
7.506
7.496
7.492
7.488
7.344
7.340
7.328
7.323
7.296
7.280
4.668
3.372
3.359
3.346
3.336
3.322
3.303
3.300
3.296
2.760
2.747
2.734
2.718
10
9
8
7
6
5
4
3
2
1
ppm
2.39
0.11
0.12
3.22
0.20
0.38
0.77
0.81
0.37
2.00
2.33

^{1}H NMR

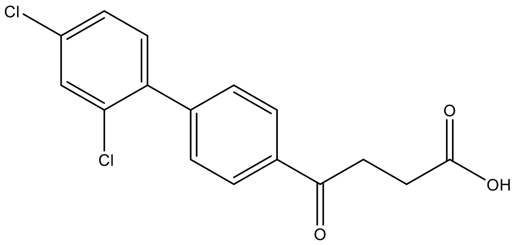
Cl
Cl
O
OH
O

6f

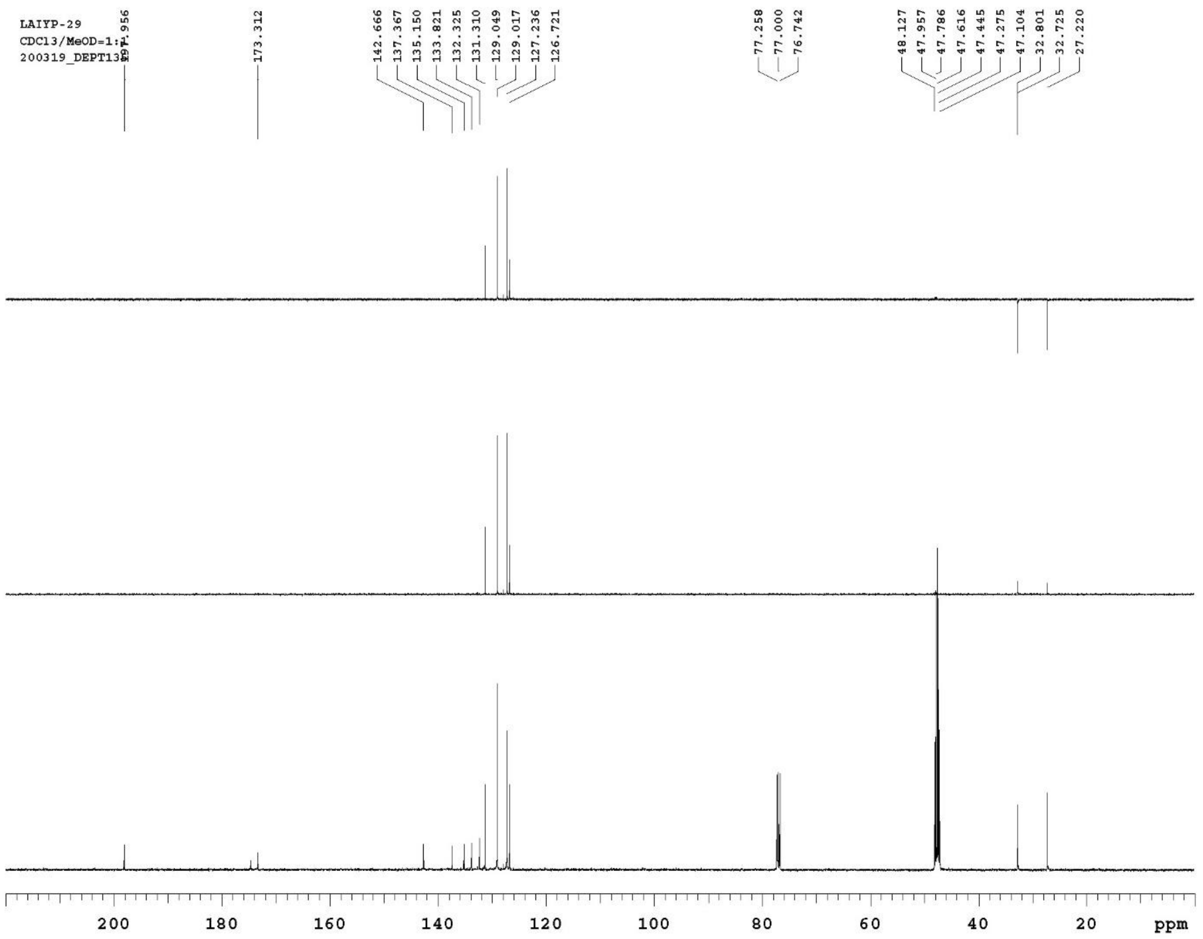

^{13}C-DEPT-135 NMR

6f

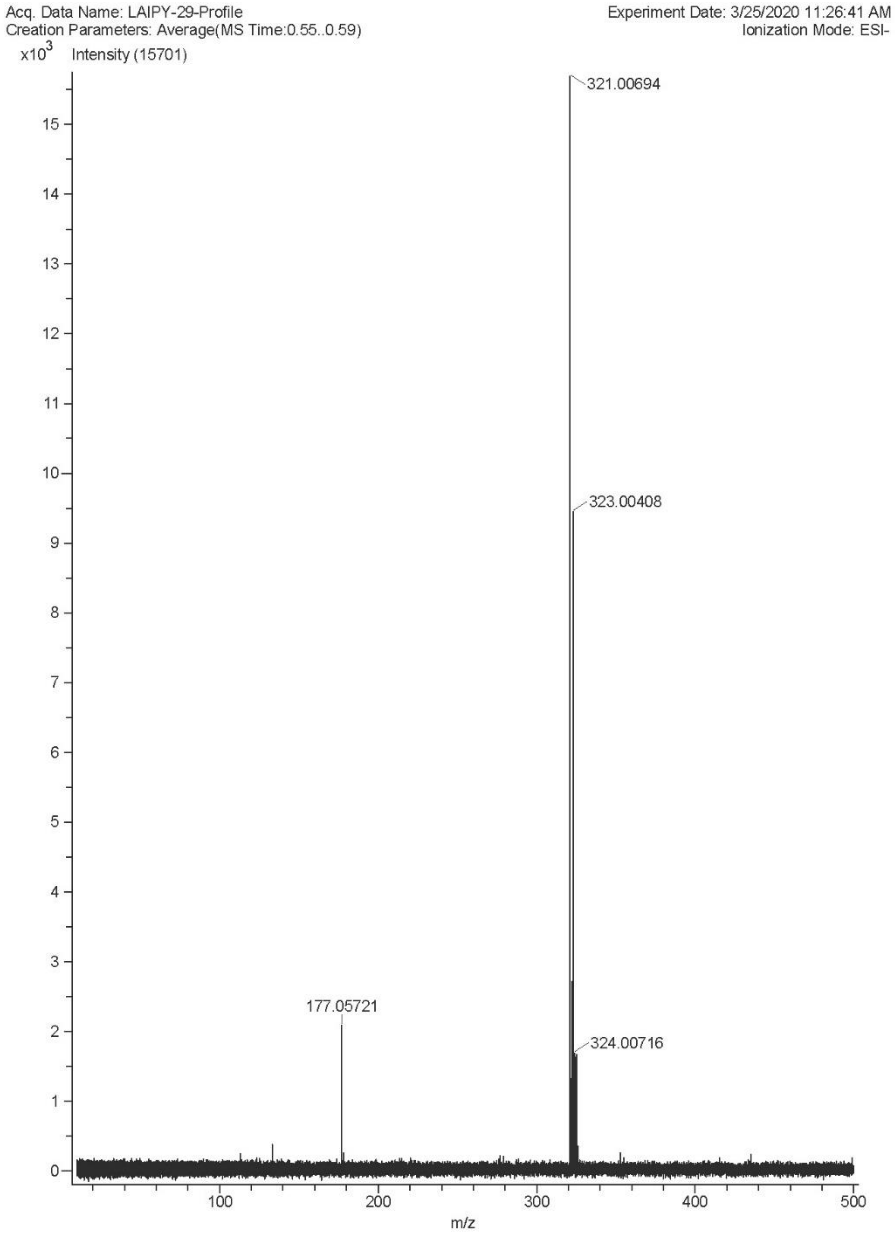

Acq. Data Name: LAIPY-29-Profile
Creation Parameters: Average(MS Time:0.55..0.59)
Experiment Date: 3/25/2020 11:26:41 AM
Ionization Mode: ESI-
x10^3 Intensity (15701)
321.00694
323.00408
324.00716
177.05721
m/z

HR ESI-MS

6f

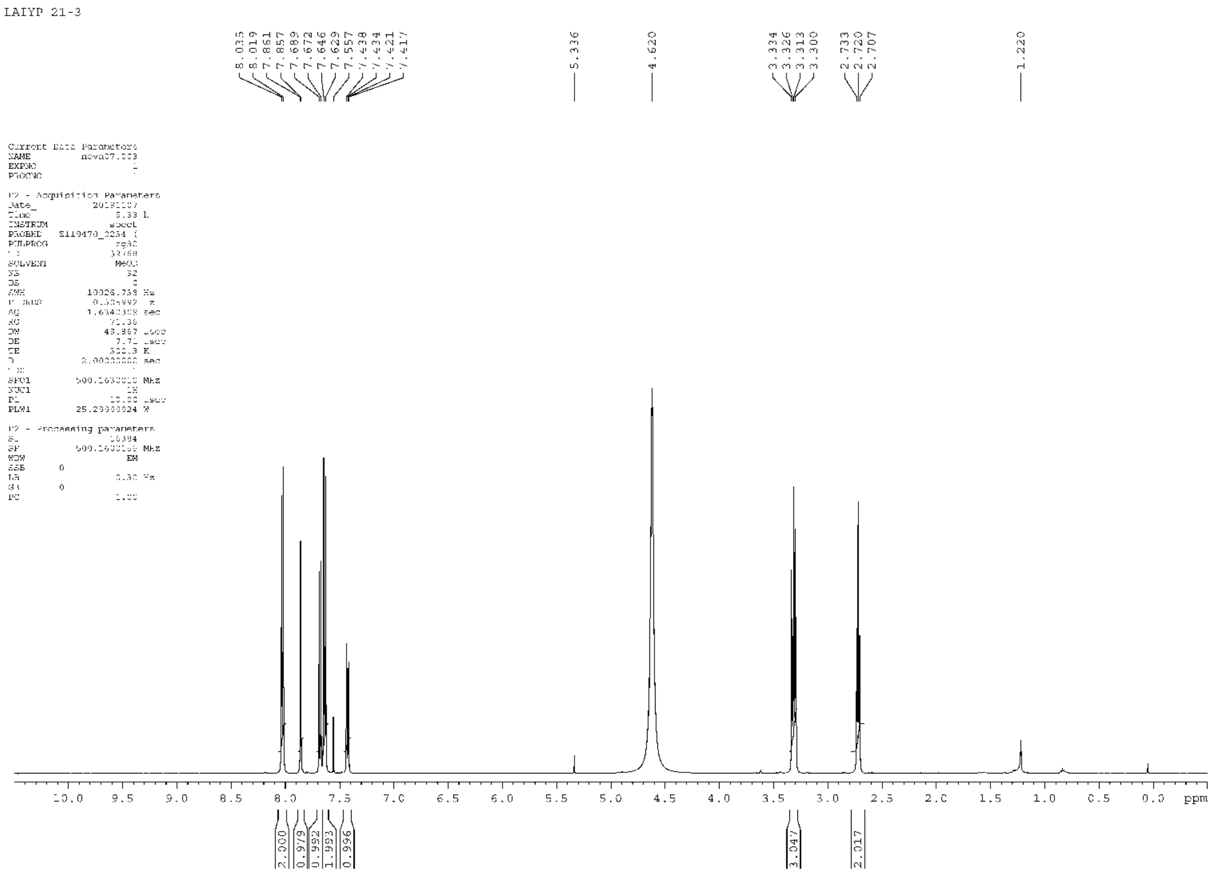
LAIYP 21-3
8.035
8.019
7.861
7.857
7.689
7.672
7.646
7.629
7.557
7.438
7.434
7.421
7.417
5.336
4.620
3.334
3.326
3.313
3.300
2.733
2.720
2.707
1.220
ppm
2.000
0.979
0.992
1.993
0.996
3.047
2.017

^{1}H NMR

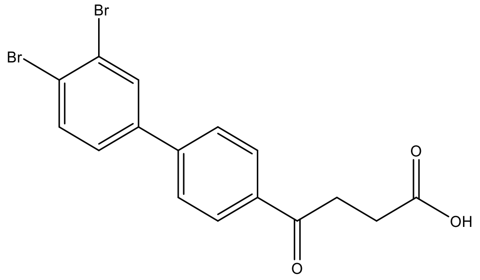
Br
Br
O
OH
O

6g

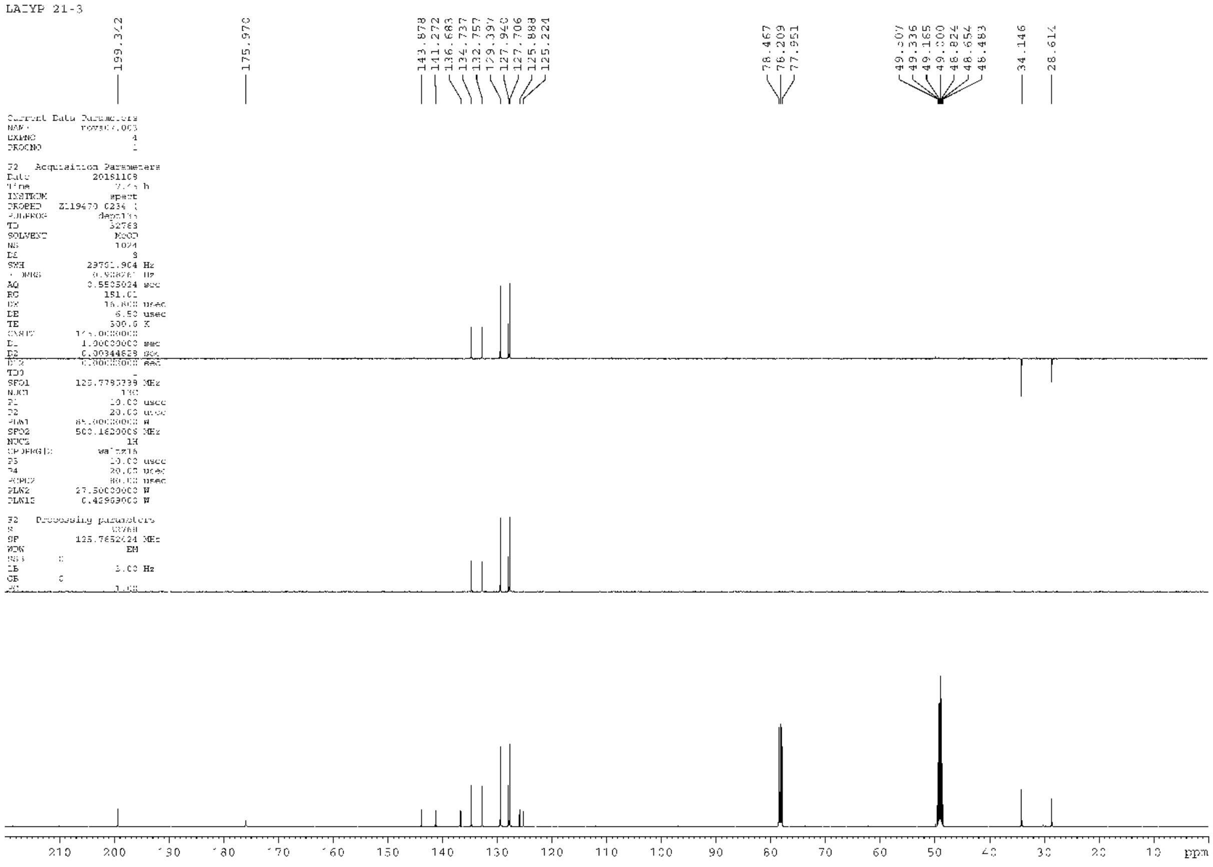

^{13}C-DEPT-135 NMR

6g

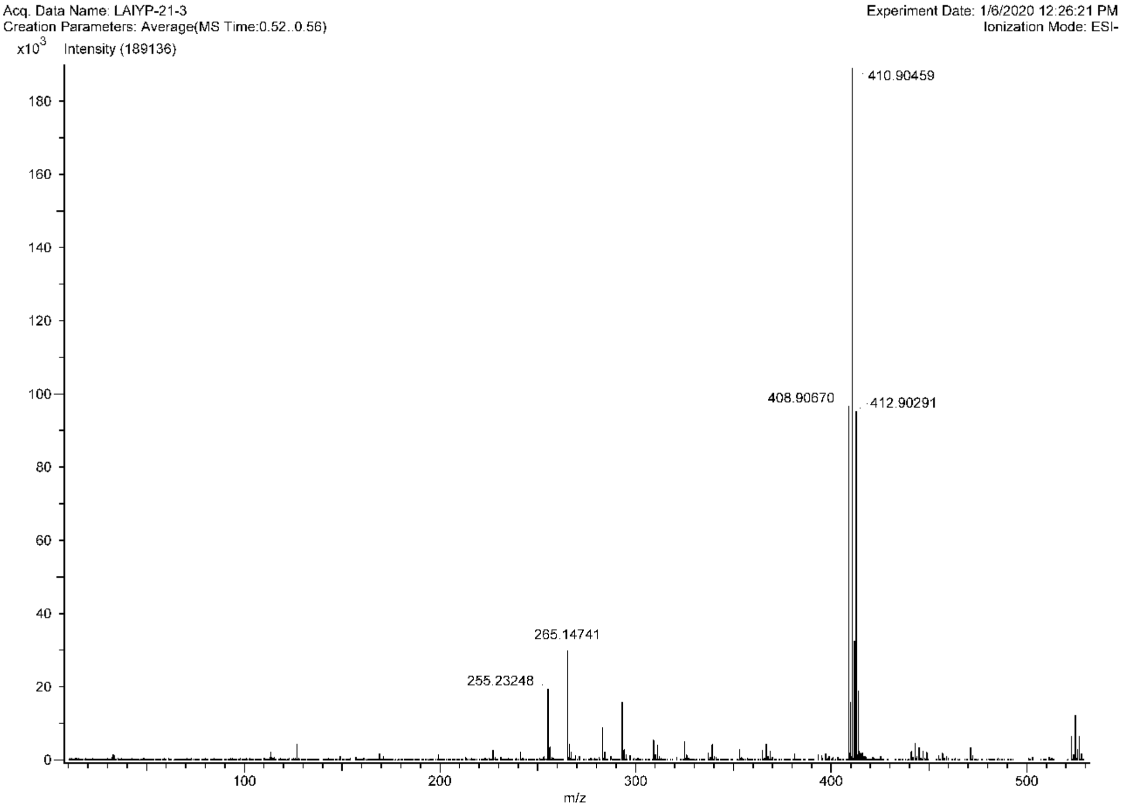

Acq. Data Name: LAIYP-21-3
Creation Parameters: Average(MS Time:0.52..0.56)
Experiment Date: 1/6/2020 12:26:21 PM
Ionization Mode: ESI-
x10^3 Intensity (189136)
410.90459
408.90670
412.90291
265.14741
255.23248
m/z

HR ESI-MS

6g

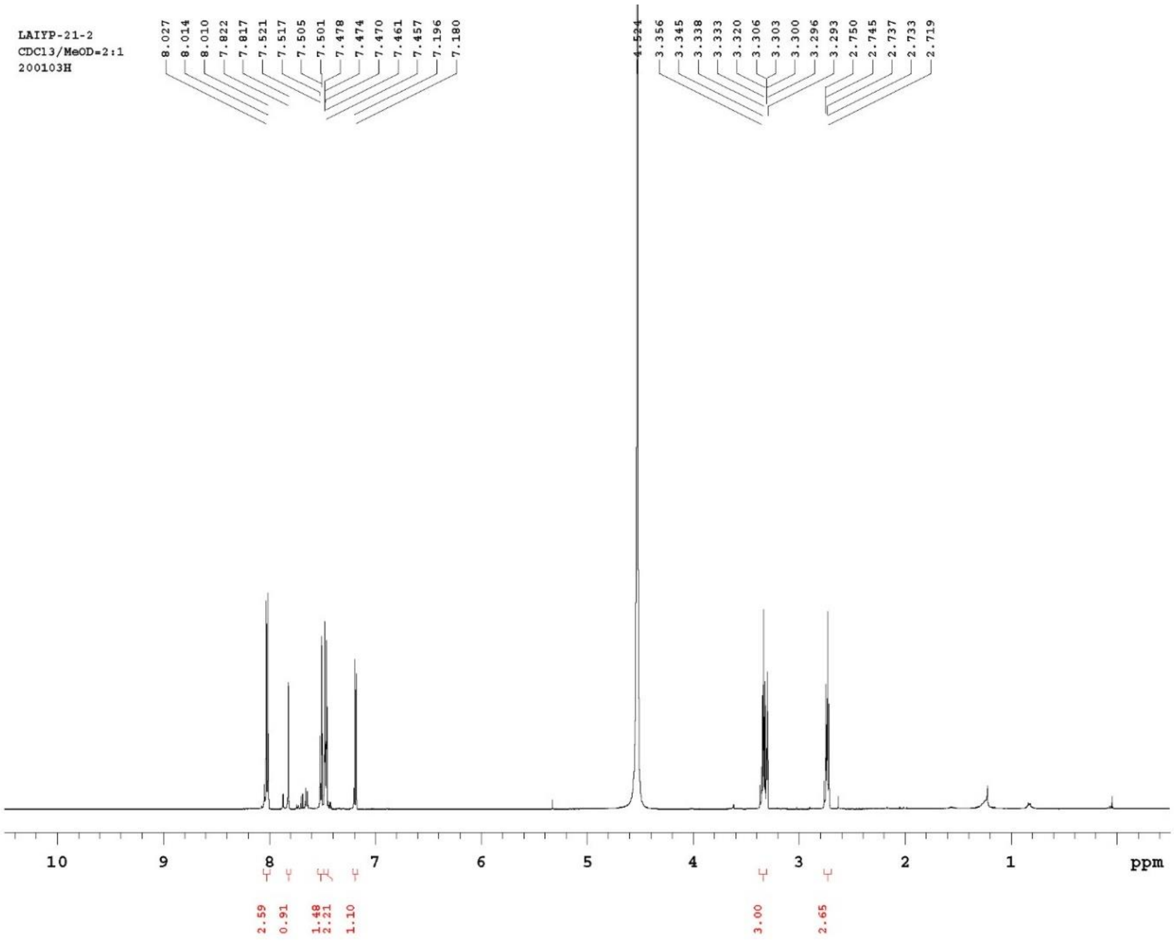
LAIYP-21-2
CDCl3/MeOD=2:1
200103H
8.027
8.014
8.010
7.822
7.817
7.521
7.517
7.505
7.501
7.478
7.474
7.470
7.461
7.457
7.196
7.180
4.524
3.356
3.345
3.338
3.333
3.320
3.306
3.303
3.300
3.296
3.293
2.750
2.745
2.737
2.733
2.719
10
9
8
7
6
5
4
3
2
1
ppm
2.59
0.91
1.48
2.21
1.10
3.00
2.65

^{1}H NMR

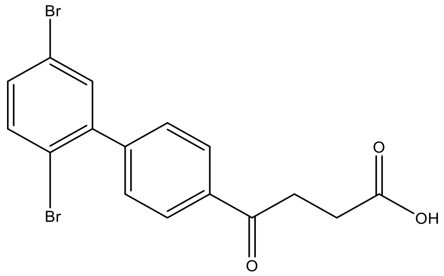
Br
Br
O
OH
O

6h

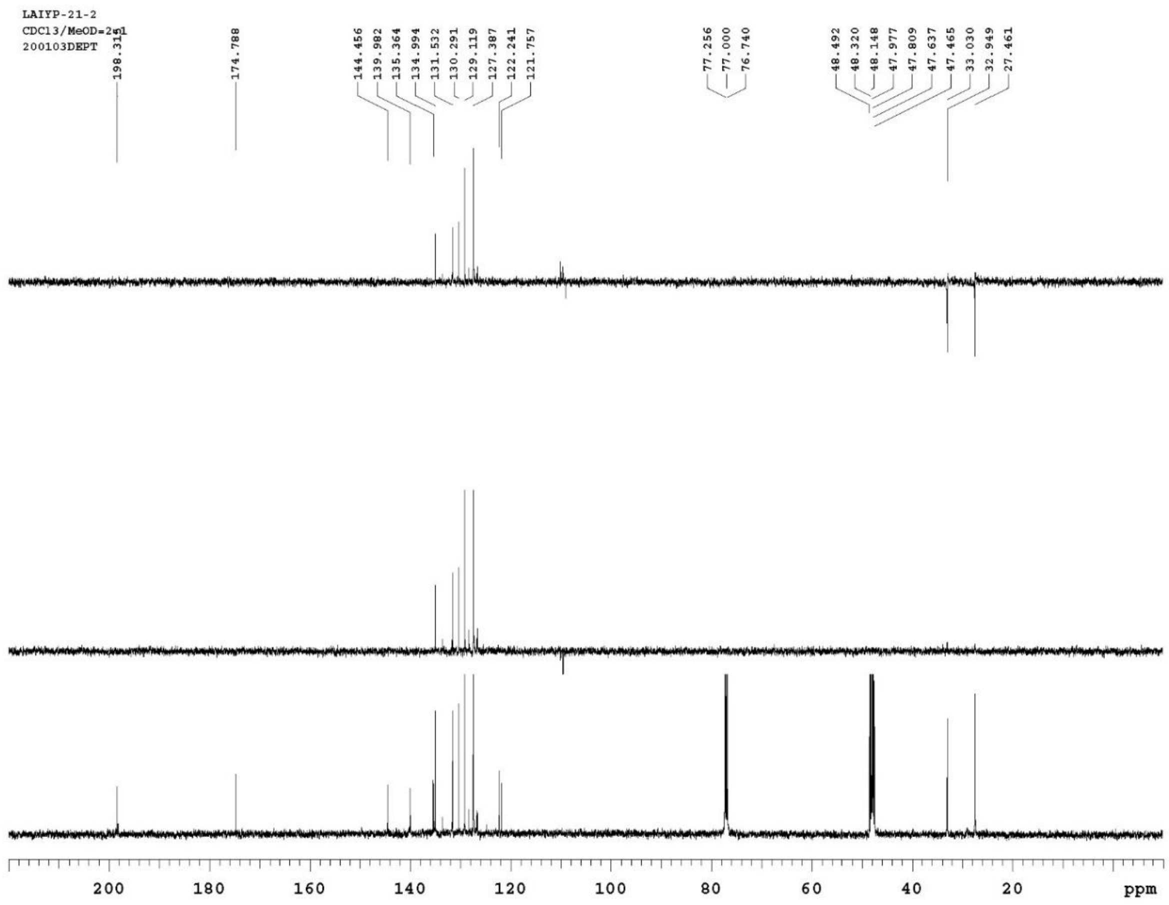

^{13}C-DEPT-135 NMR

6h

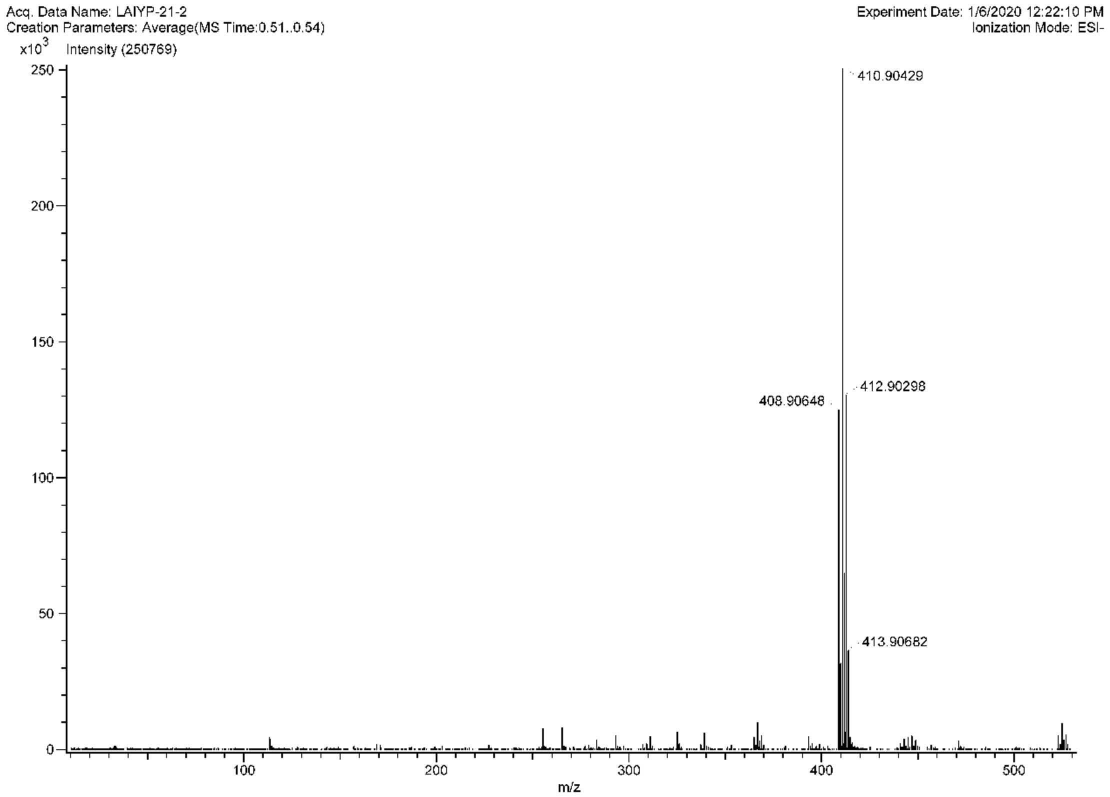
Acq. Data Name: LAIYP-21-2
Creation Parameters: Average(MS Time:0.51..0.54)
x10^3 Intensity (250769)
Experiment Date: 1/6/2020 12:22:10 PM
Ionization Mode: ESI-
410.90429
412.90298
408.90648
413.90682
m/z

HR ESI-MS

6h

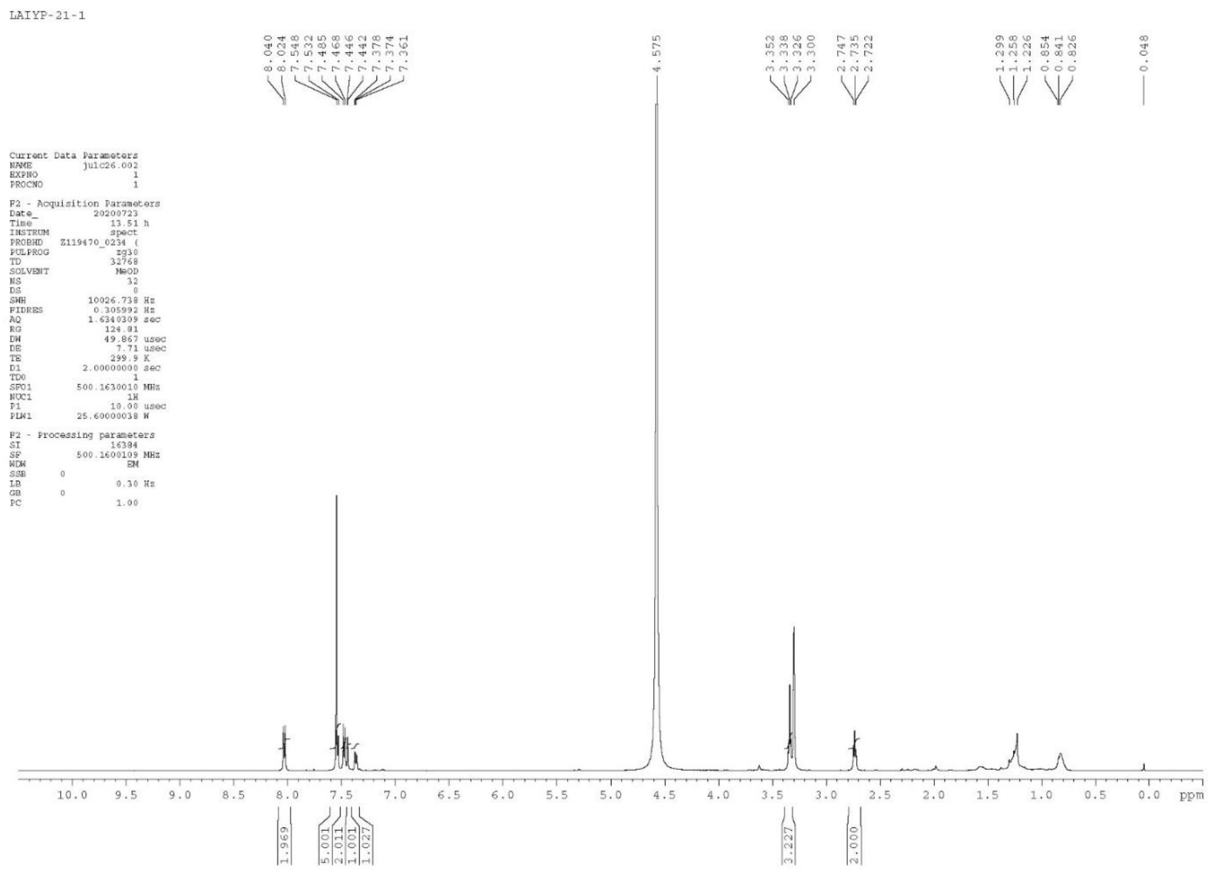

^{1}H NMR

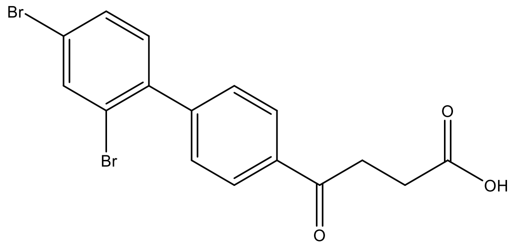

6i

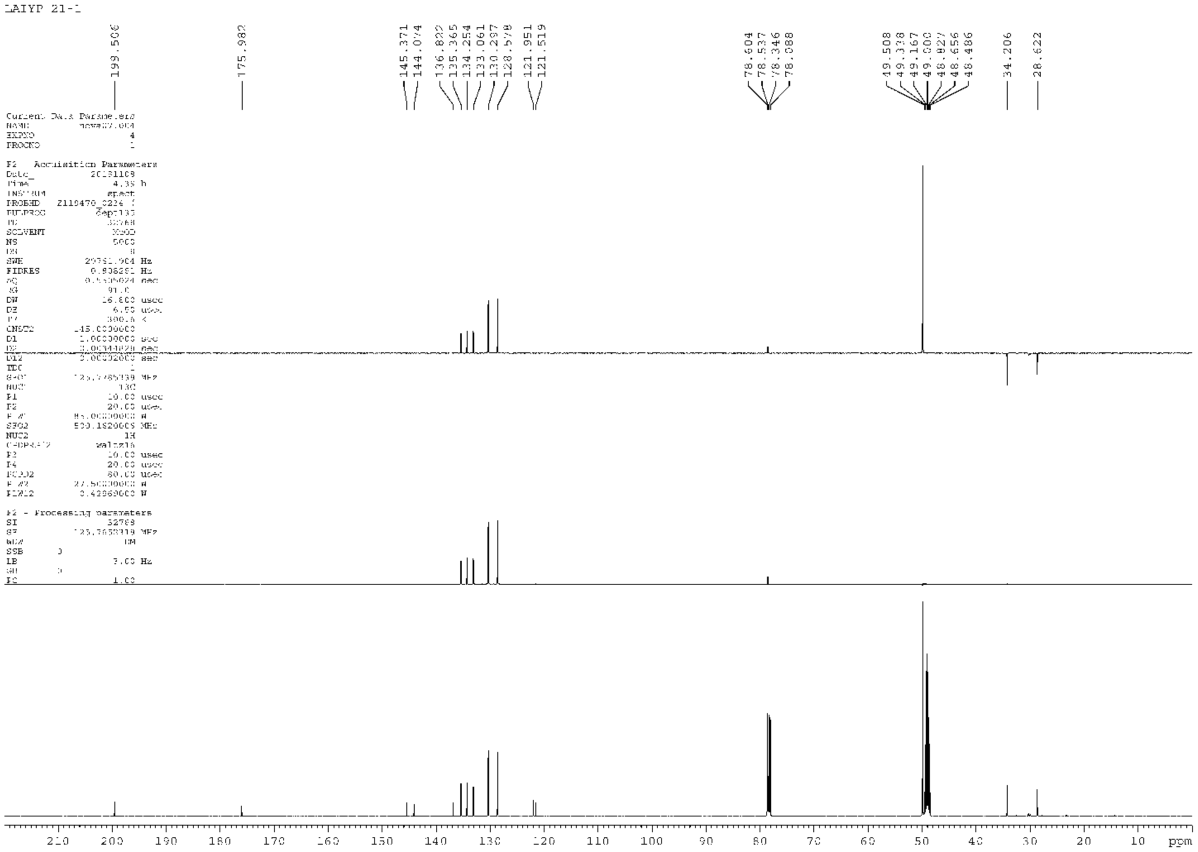

^{13}C-DEPT-135 NMR

6i

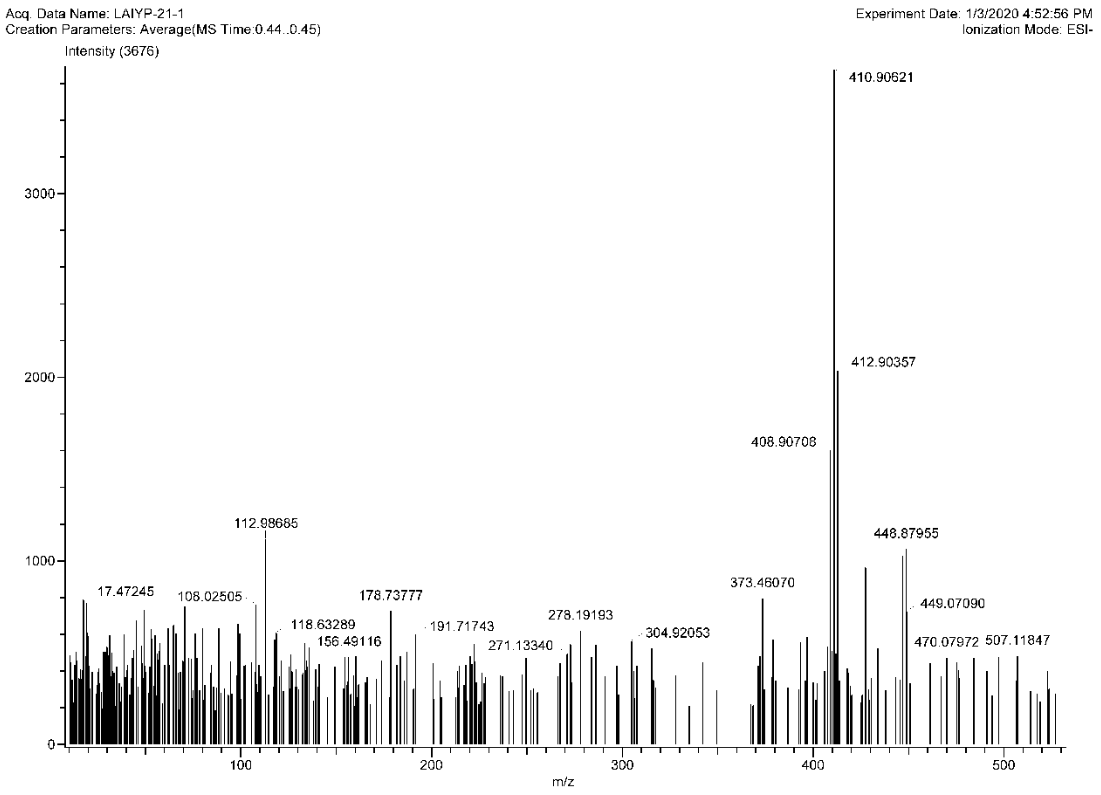
Acq. Data Name: LAIYP-21-1
Creation Parameters: Average(MS Time:0.44..0.45)
Experiment Date: 1/3/2020 4:52:56 PM
Ionization Mode: ESI-
Intensity (3676)
410.90621
412.90357
408.90708
112.98685
448.87955
373.46070
17.47245
108.02505
178.73777
449.07090
118.63289
191.71743
278.19193
156.49116
304.92053
271.13340
470.07972
507.11847
m/z

HR ESI-MS

6i

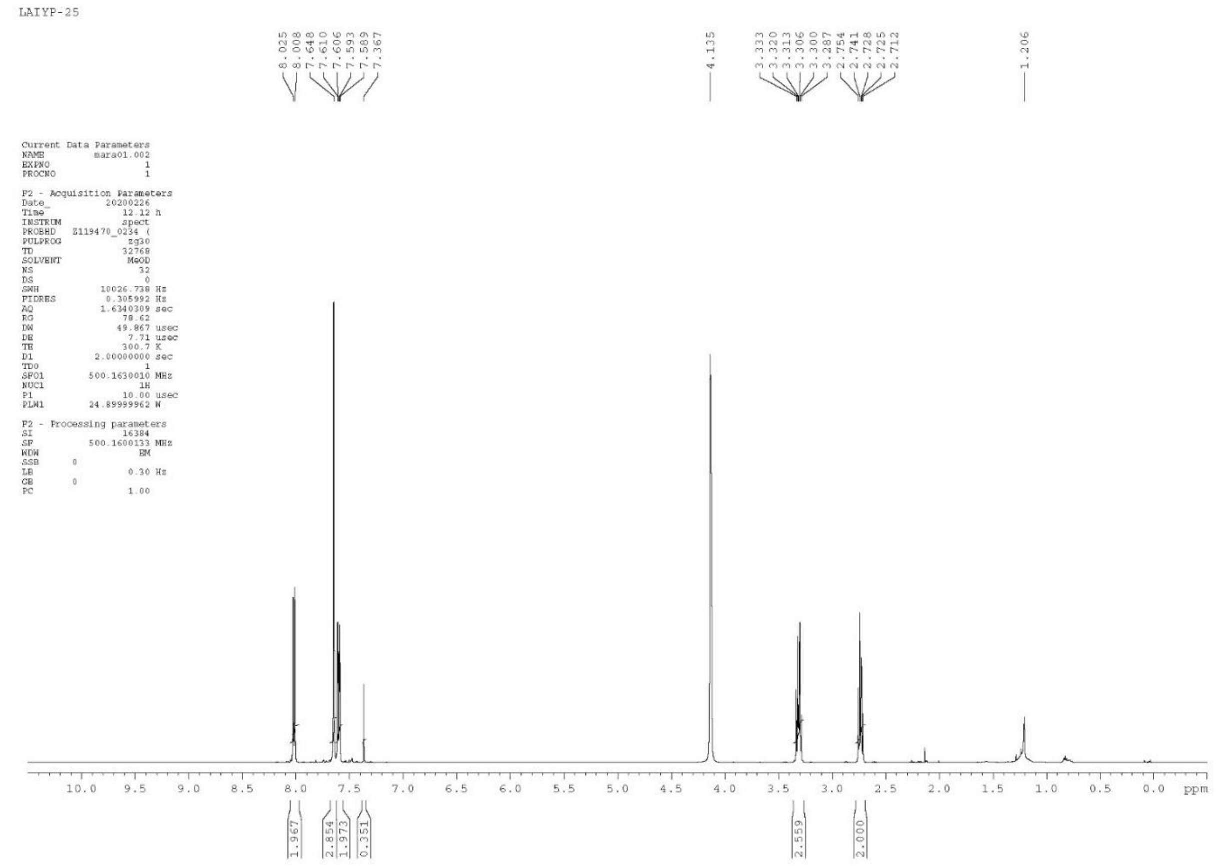
LAIYP-25
8.025
8.008
7.648
7.610
7.606
7.593
7.589
7.367
4.135
3.333
3.320
3.313
3.306
3.300
3.287
2.754
2.741
2.728
2.725
2.712
1.206
Current Data Parameters
NAME mara01.002
EXPNO 1
PROCNO 1
F2 - Acquisition Parameters
Date_ 20200226
Time 12.12 h
INSTRUM spect
PROBHD Z119470_0234 (
PULPROG zg30
TD 32768
SOLVENT MeOD
NS 32
DS 0
SWH 10026.738 Hz
FIDRES 0.305992 Hz
AQ 1.6340309 sec
RG 78.62
DW 49.867 usec
DE 7.71 usec
TE 300.7 K
D1 2.00000000 sec
TD0 1
SFO1 500.1630010 MHz
NUC1 1H
P1 10.00 usec
PLW1 24.89999962 W
F2 - Processing parameters
SI 16384
SF 500.1600133 MHz
WDW EM
SSB 0
LB 0.30 Hz
GB 0
PC 1.00
10.0 9.5 9.0 8.5 8.0 7.5 7.0 6.5 6.0 5.5 5.0 4.5 4.0 3.5 3.0 2.5 2.0 1.5 1.0 0.5 0.0 ppm
1.967
2.854
1.973
0.351
2.559
2.000

^{1}H NMR

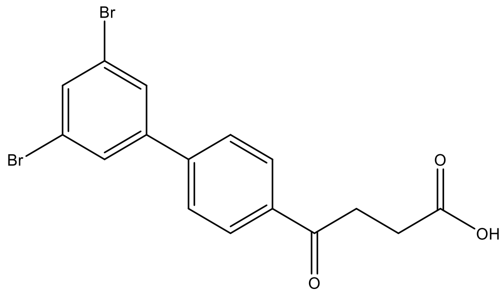
Br
Br
O
OH
O

6j

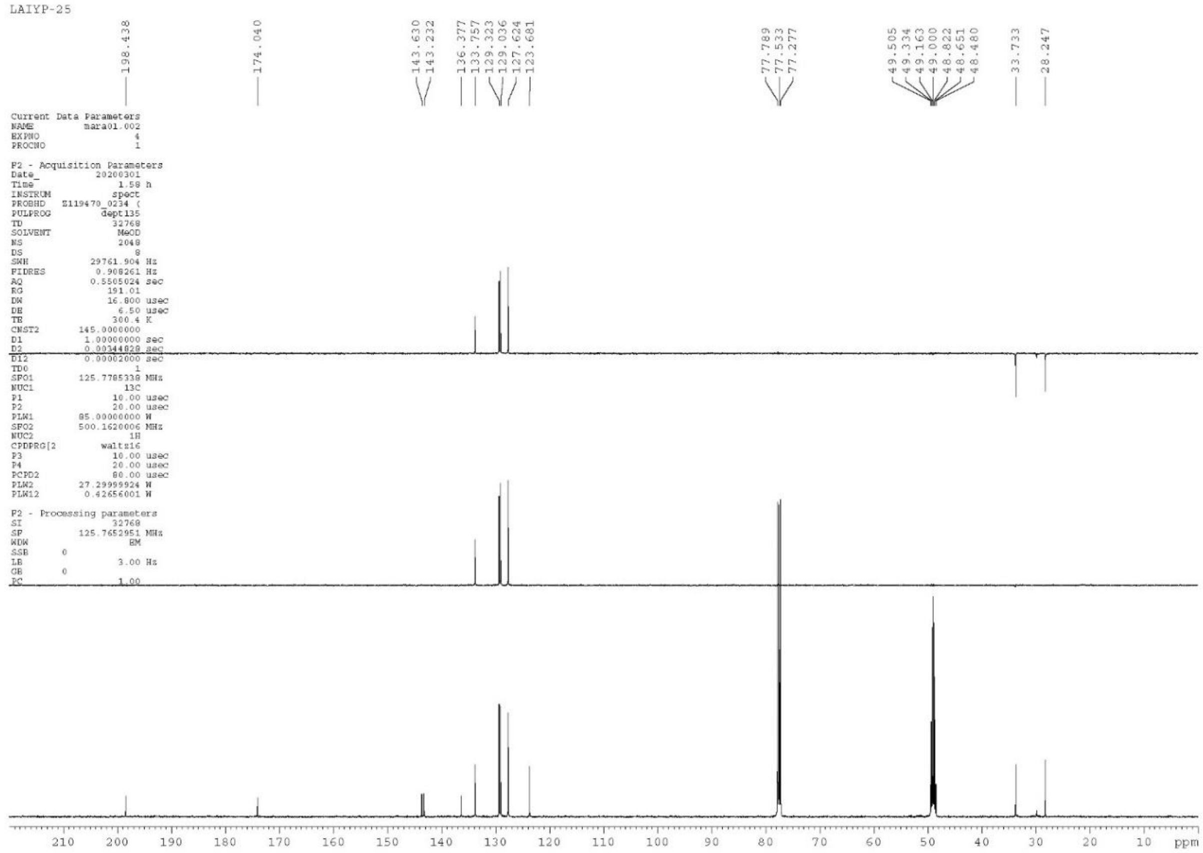

^{13}C-DEPT-135 NMR

6j

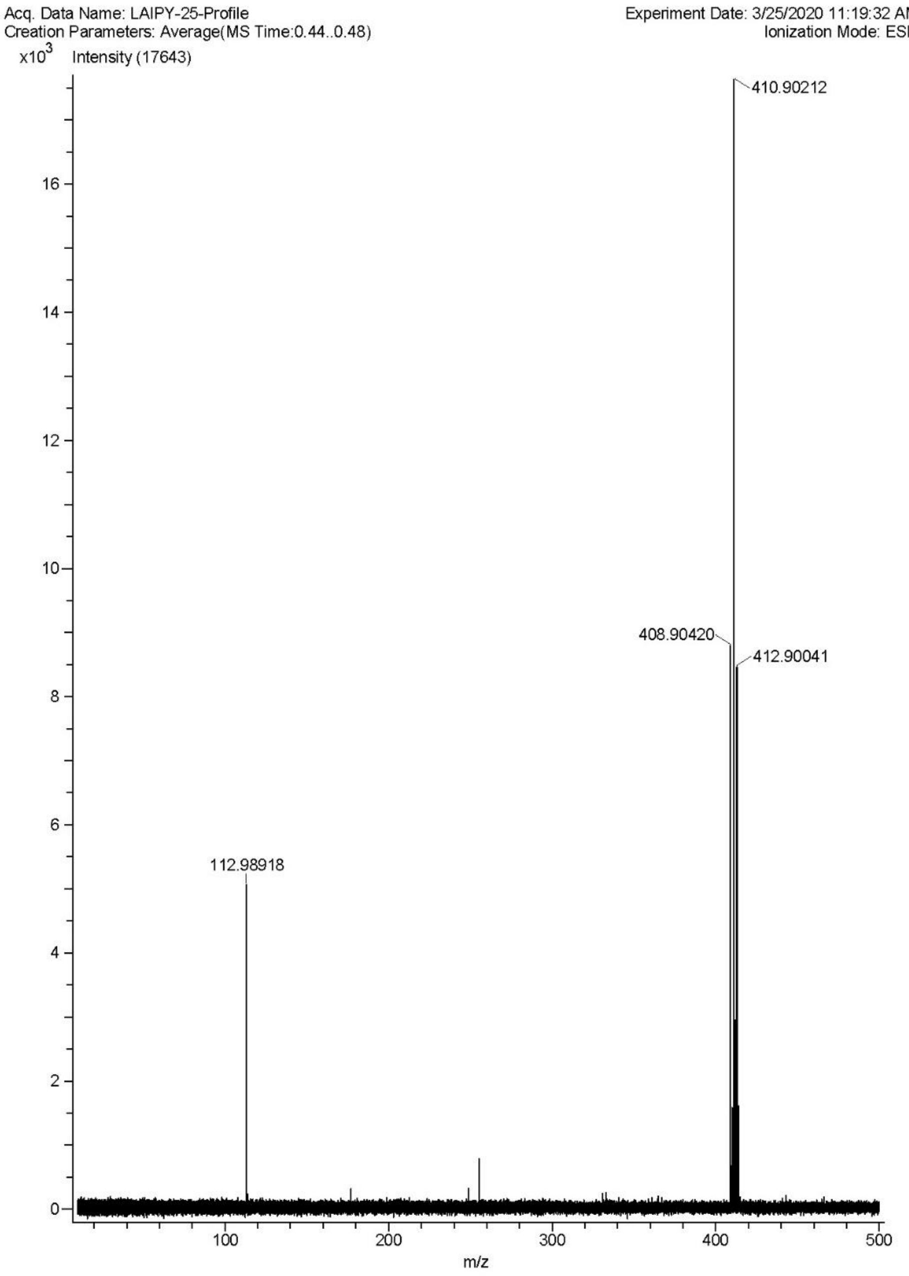

Acq. Data Name: LAIPY-25-Profile
Creation Parameters: Average(MS Time:0.44..0.48)
Experiment Date: 3/25/2020 11:19:32 AM
Ionization Mode: ESI-
x10^3 Intensity (17643)
410.90212
408.90420
412.90041
112.98918
m/z

HR ESI-MS

6j

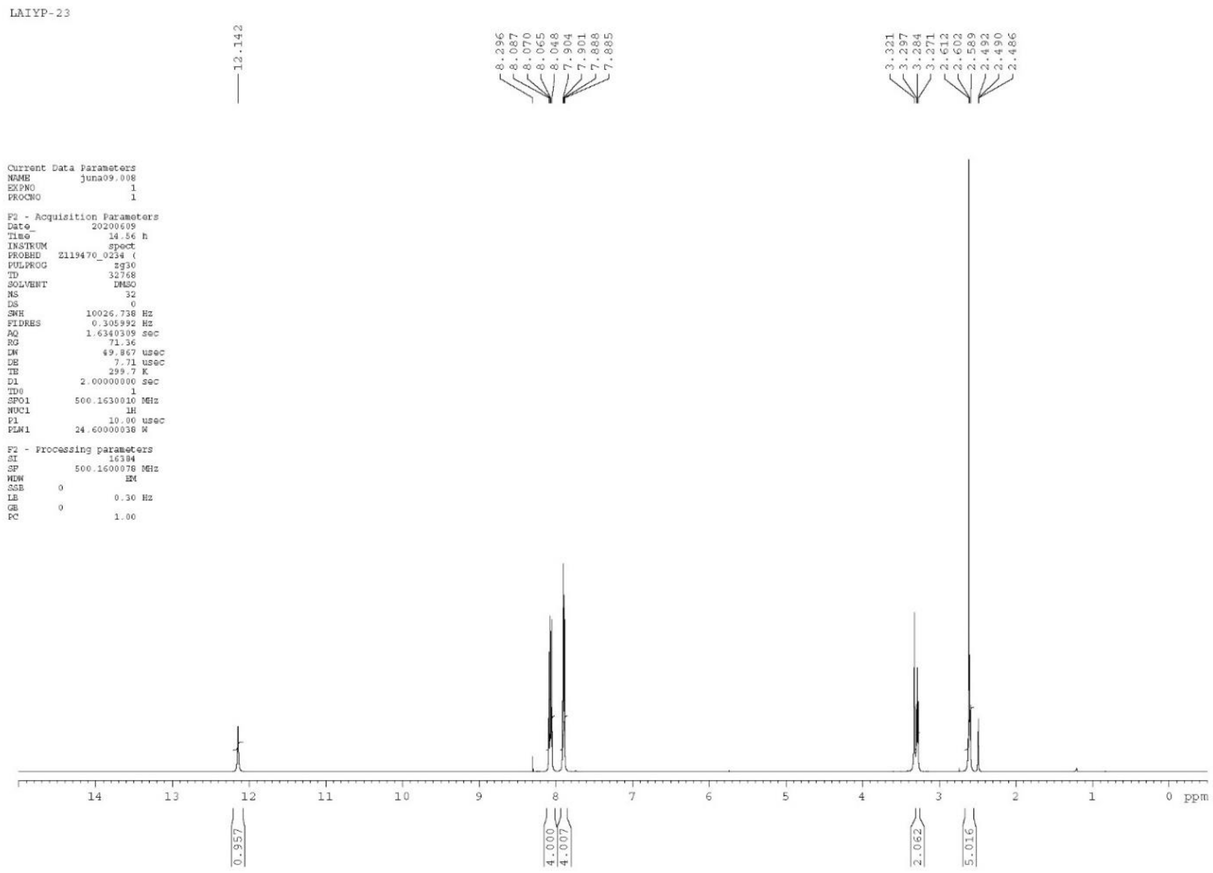

LAIYP-23
12.142
8.296
8.087
8.070
8.065
8.048
7.904
7.901
7.888
7.885
3.321
3.297
3.284
3.271
2.612
2.602
2.589
2.492
2.490
2.486
Current Data Parameters
NAME juna09.008
EXPNO 1
PROCNO 1
F2 - Acquisition Parameters
Date_ 20200609
Time 14.56 h
INSTRUM spect
PROBHD Z119470_0234 (
PULPROG zg30
TD 32768
SOLVENT DMSO
NS 32
DS 0
SWH 10026.738 Hz
FIDRES 0.305992 Hz
AQ 1.6340309 sec
RG 71.36
DW 49.867 usec
DE 7.71 usec
TE 299.7 K
D1 2.00000000 sec
TD0 1
SFO1 500.1630010 MHz
NUC1 1H
P1 10.00 usec
PLW1 24.60000038 W
F2 - Processing parameters
SI 16384
SF 500.1600078 MHz
WDW EM
SSB 0
LB 0.30 Hz
GB 0
PC 1.00
14 13 12 11 10 9 8 7 6 5 4 3 2 1 0 ppm
0.957
4.000
4.007
2.062
5.016

^{1}H NMR

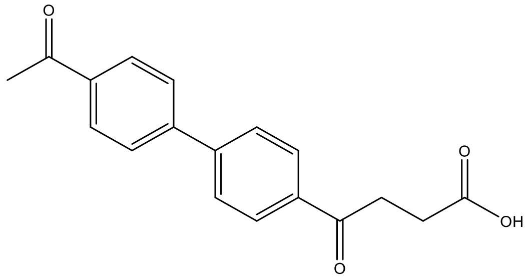

O
O
OH
O

6k

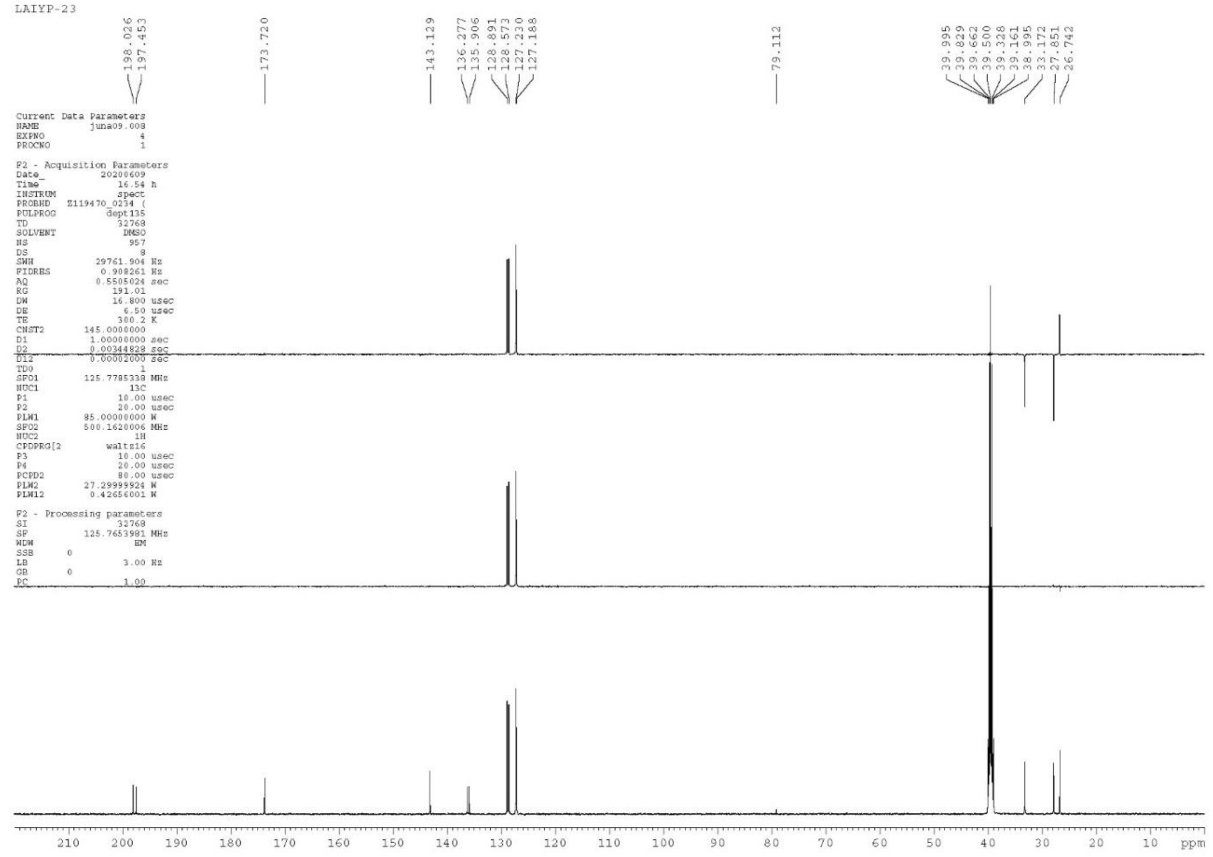

^{13}C-DEPT-135 NMR

6k

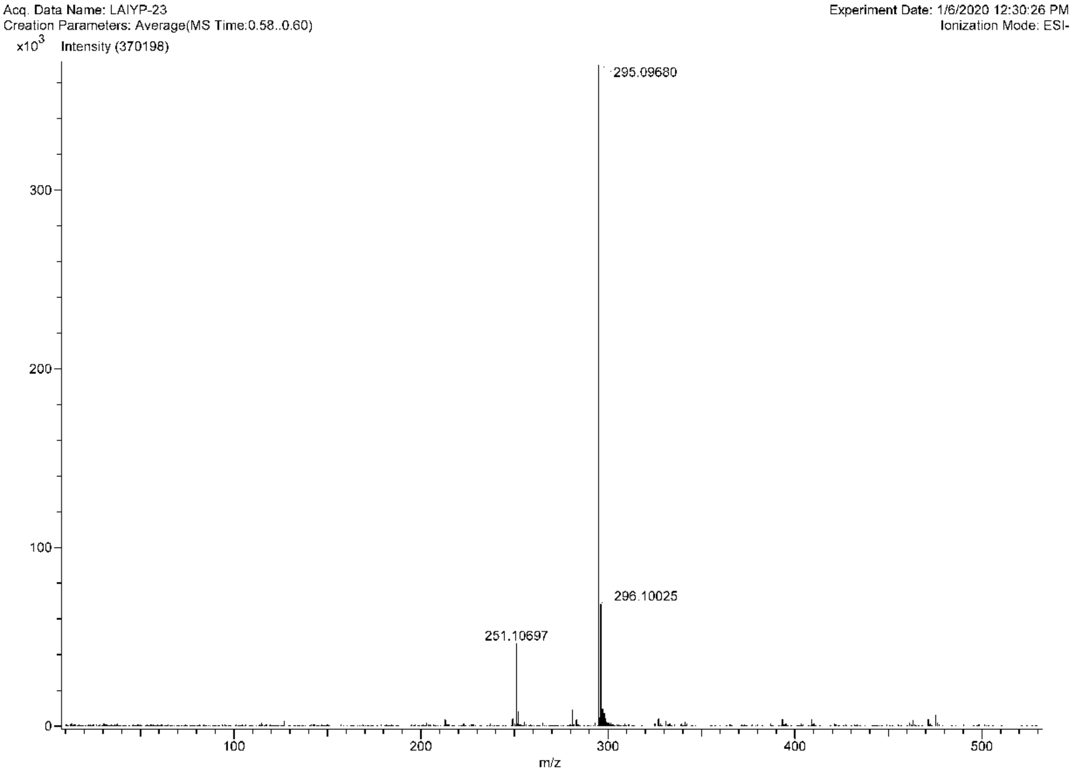
Acq. Data Name: LAIYP-23
Creation Parameters: Average(MS Time:0.58..0.60)
Experiment Date: 1/6/2020 12:30:26 PM
Ionization Mode: ESI-
x10^3 Intensity (370198)
295.09680
296.10025
251.10697
m/z

HR ESI-MS

6k

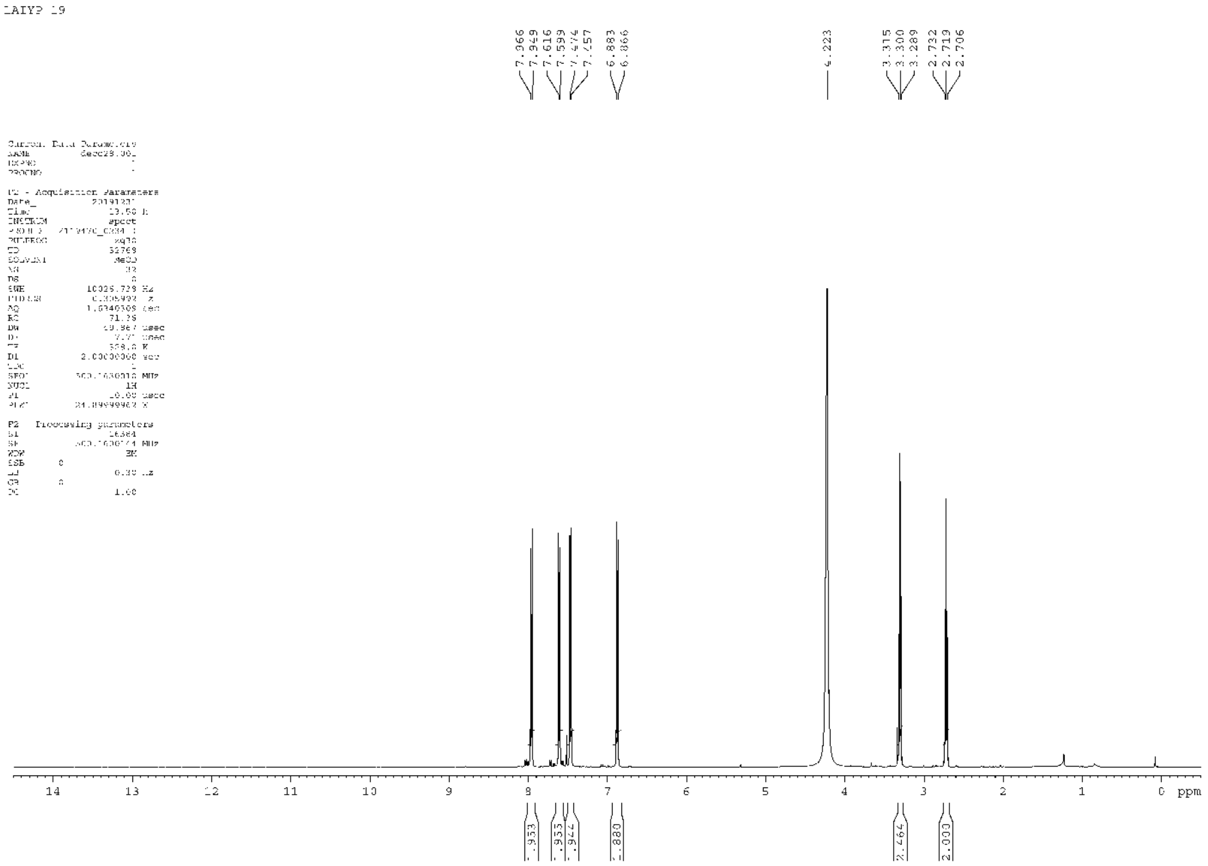
LAIYP 19
7.966
7.949
7.616
7.599
7.474
7.457
6.883
6.866
4.223
3.315
3.300
3.289
2.732
2.719
2.706
14
13
12
11
10
9
8
7
6
5
4
3
2
1
0
ppm
2.464
2.000

^{1}H NMR

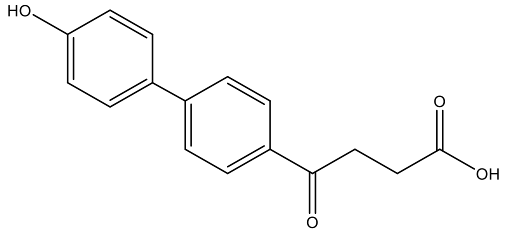
HO
O
OH
O

6l

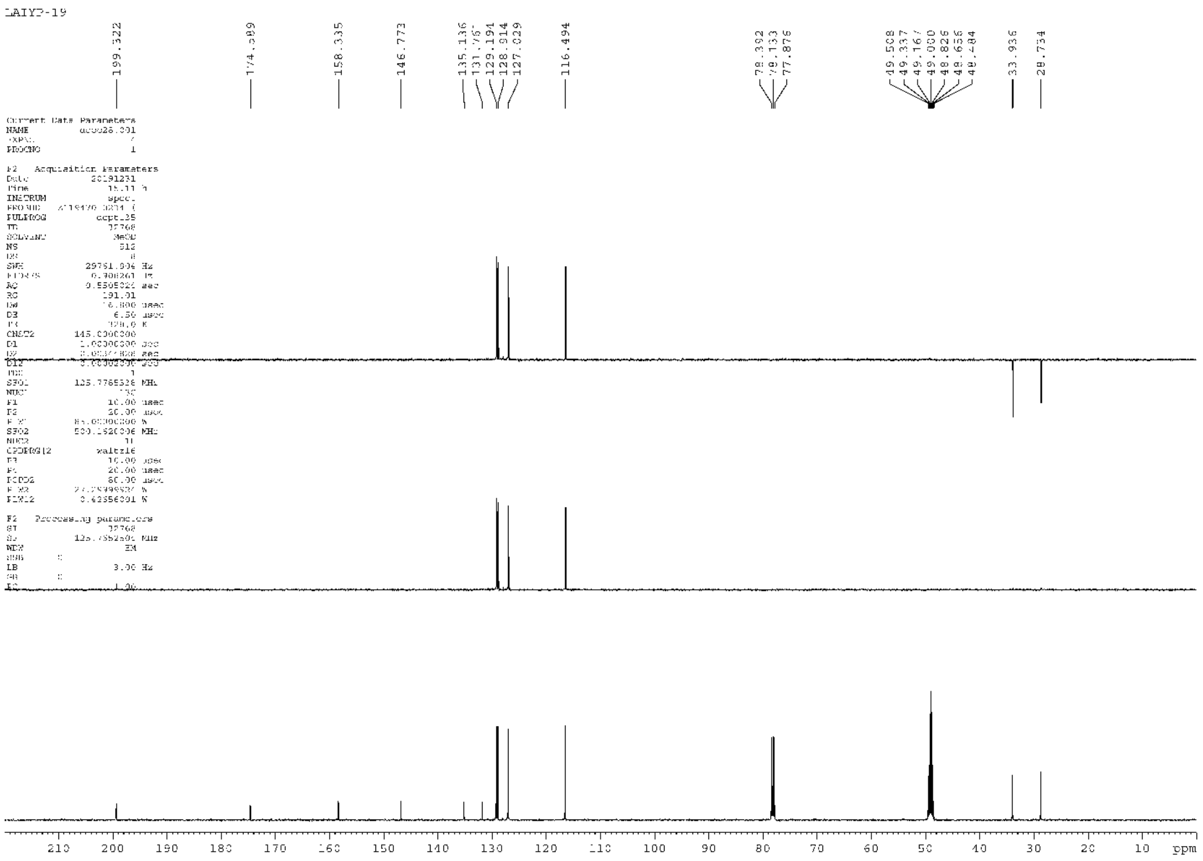

^{13}C-DEPT-135 NMR

6l

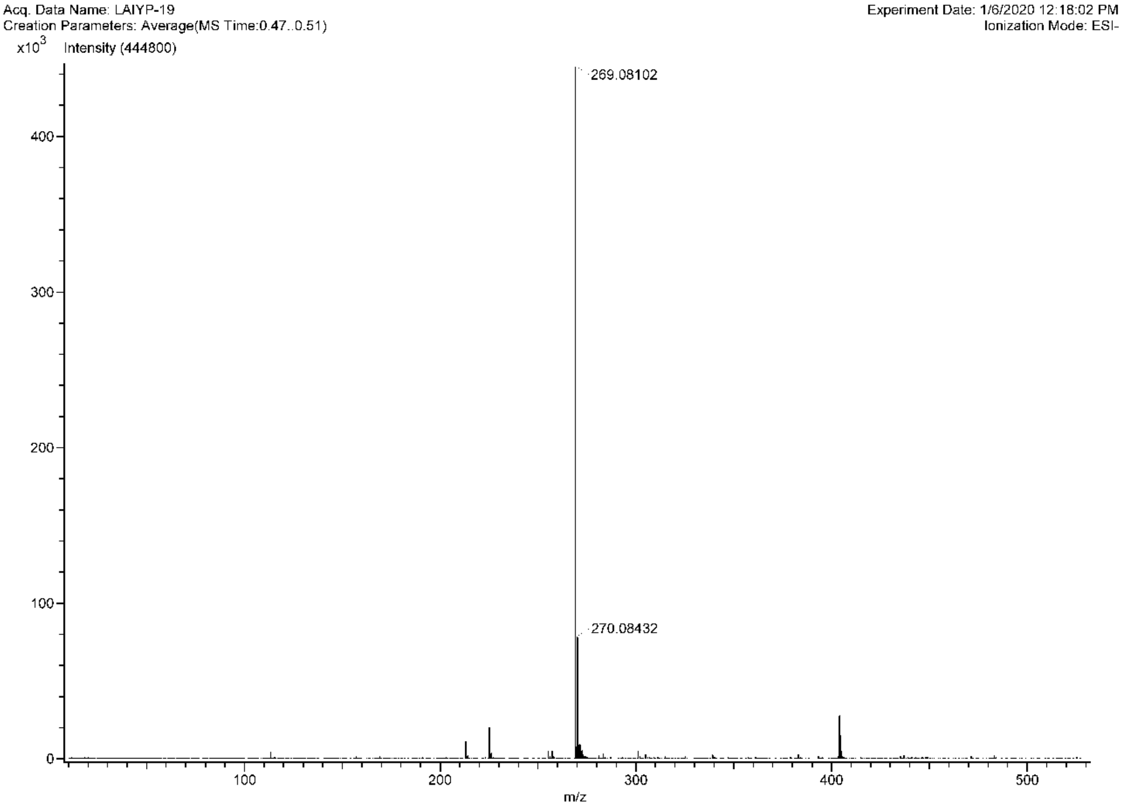
Acq. Data Name: LAIYP-19
Creation Parameters: Average(MS Time:0.47..0.51)
Experiment Date: 1/6/2020 12:18:02 PM
Ionization Mode: ESI-
x10^3 Intensity (444800)
269.08102
270.08432
m/z

HR ESI-MS

6l

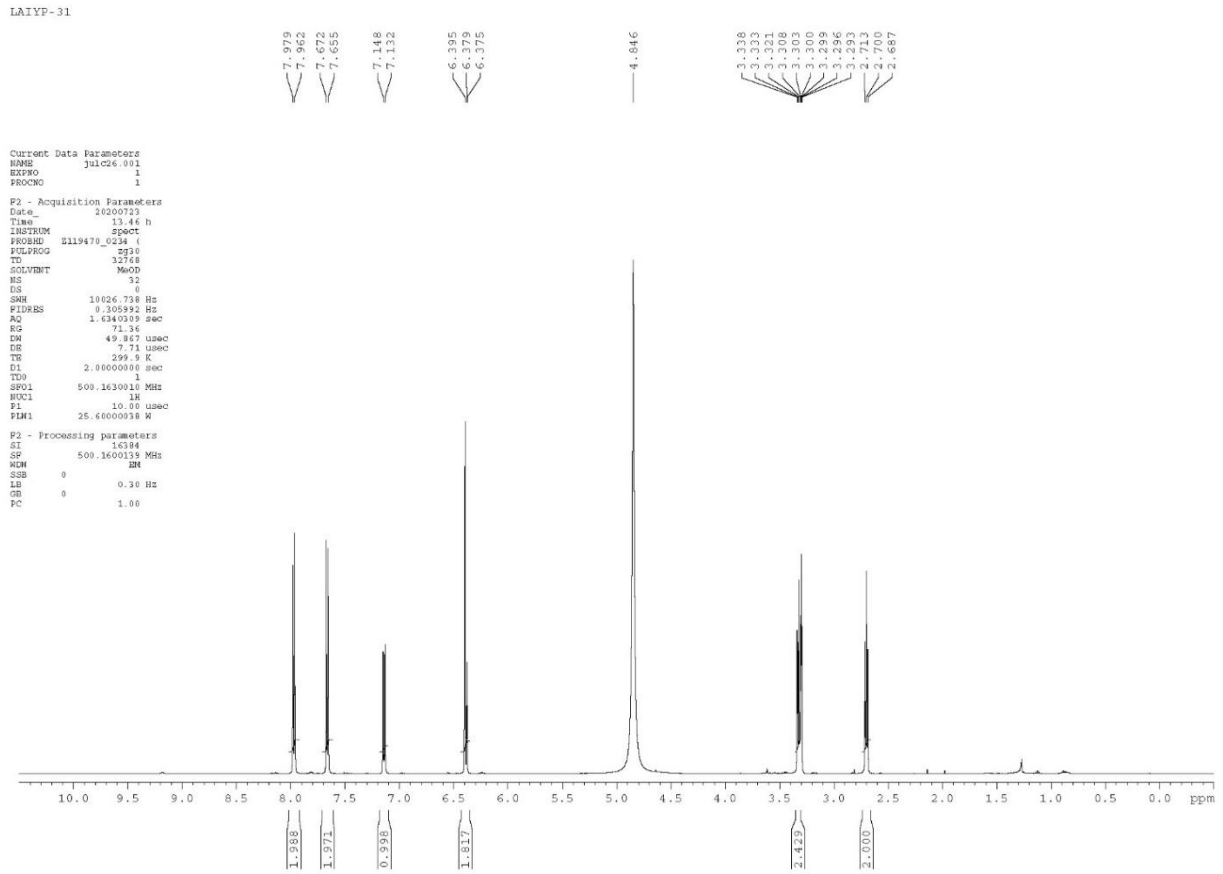
LAIYP-31
7.979
7.962
7.672
7.655
7.148
7.132
6.395
6.379
6.375
4.846
3.338
3.333
3.321
3.308
3.303
3.300
3.299
3.296
3.293
2.713
2.700
2.687
Current Data Parameters
NAME julc26.001
EXPNO 1
PROCNO 1
F2 - Acquisition Parameters
Date_ 20200723
Time 13.46 h
INSTRUM spect
PROBHD Z119470_0234 (
PULPROG zg30
TD 32768
SOLVENT MeOD
NS 32
DS 0
SWH 10026.738 Hz
FIDRES 0.305992 Hz
AQ 1.6340309 sec
RG 71.36
DW 49.867 usec
DE 7.71 usec
TE 299.9 K
D1 2.00000000 sec
TD0 1
SFO1 500.1630010 MHz
NUC1 1H
P1 10.00 usec
PLW1 25.60000038 W
F2 - Processing parameters
SI 16384
SF 500.1600139 MHz
WDW EM
SSB 0
LB 0.30 Hz
GB 0
PC 1.00
10.0 9.5 9.0 8.5 8.0 7.5 7.0 6.5 6.0 5.5 5.0 4.5 4.0 3.5 3.0 2.5 2.0 1.5 1.0 0.5 0.0 ppm
1.988
1.971
0.998
1.817
2.429
2.000

^{1}H NMR

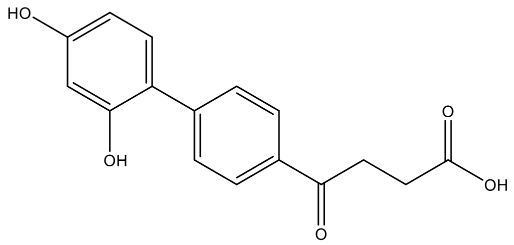
HO
OH
O
OH
O

6m

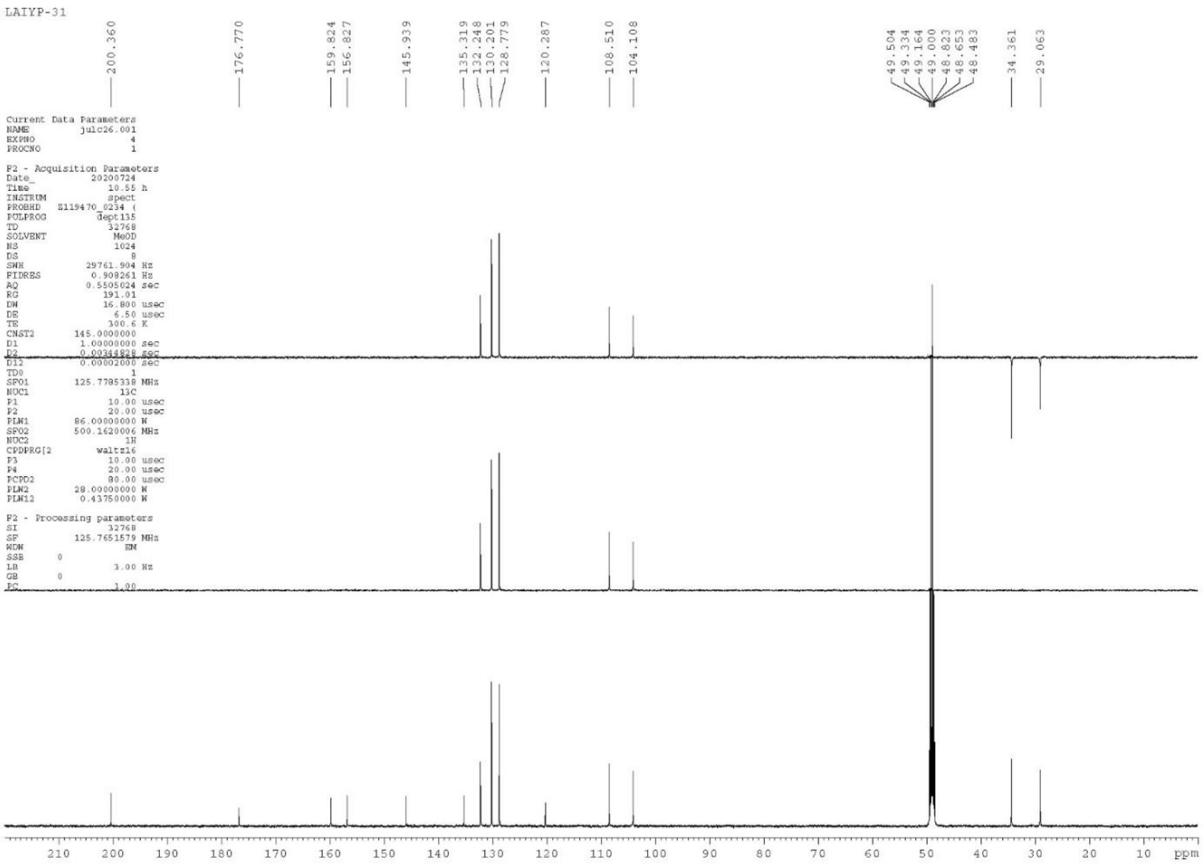

^{13}C-DEPT-135 NMR

6m

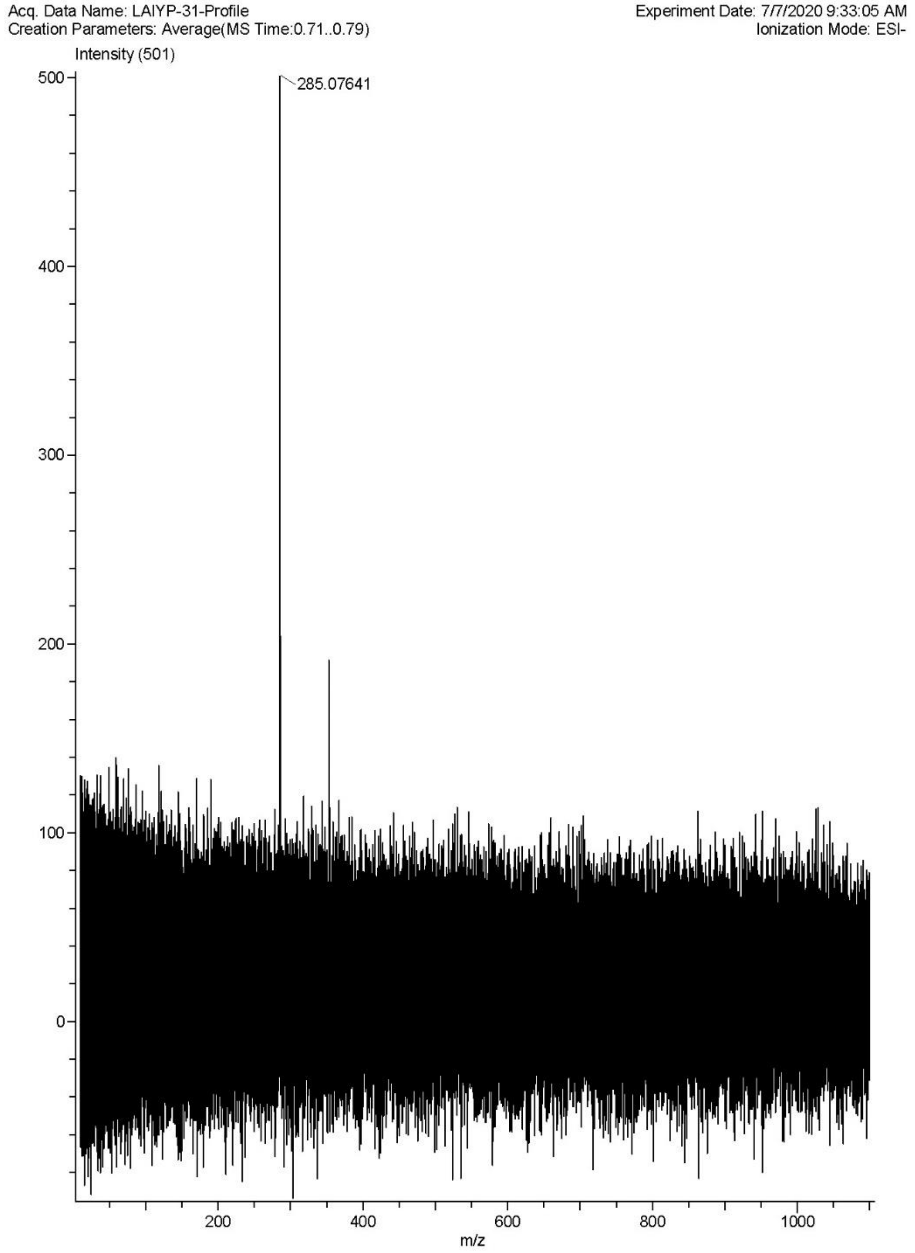
Acq. Data Name: LAIYP-31-Profile
Creation Parameters: Average(MS Time:0.71..0.79)
Experiment Date: 7/7/2020 9:33:05 AM
Ionization Mode: ESI-
Intensity (501)
285.07641
500
400
300
200
100
0
200
400
600
800
1000
m/z

HR ESI-MS

6m

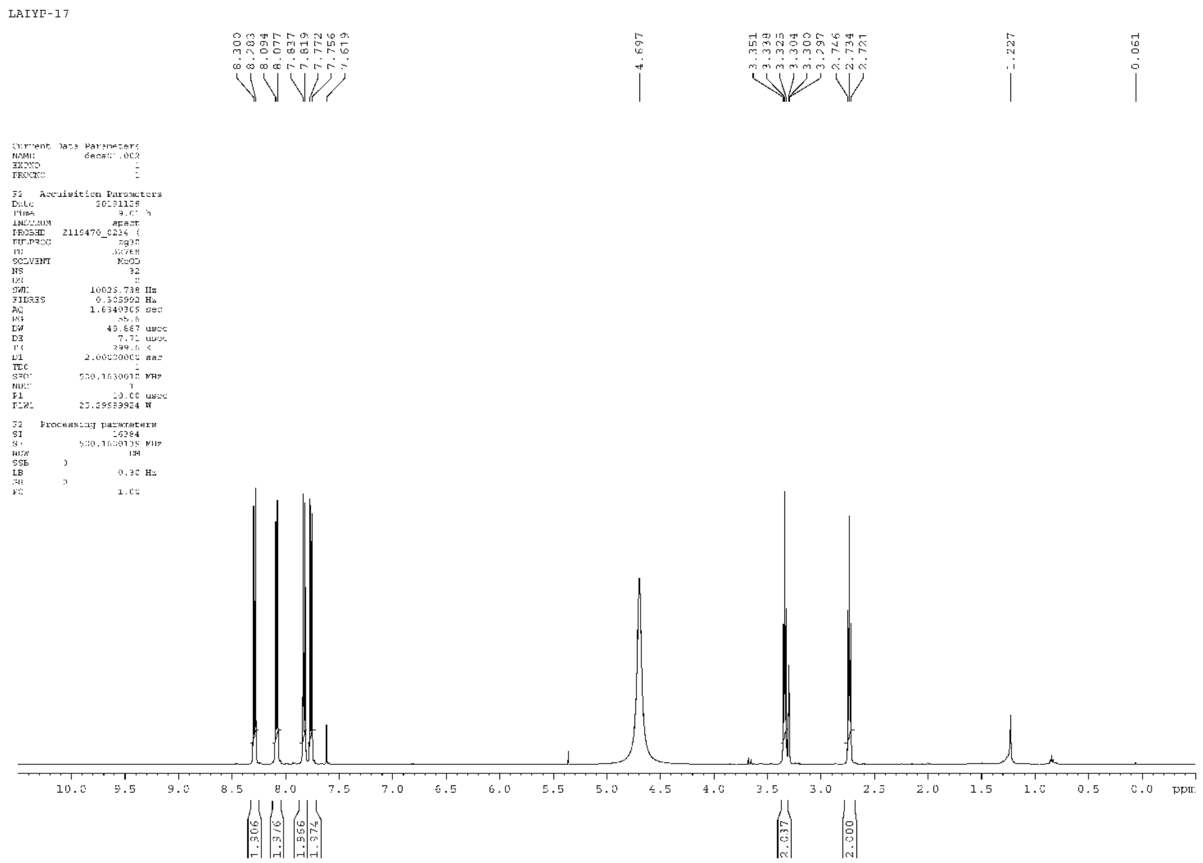
LAIYP-17
8.300
8.283
8.094
8.077
7.837
7.819
7.772
7.756
7.619
4.697
3.351
3.338
3.325
3.304
3.300
3.297
2.746
2.734
2.721
1.227
0.061
10.0 9.5 9.0 8.5 8.0 7.5 7.0 6.5 6.0 5.5 5.0 4.5 4.0 3.5 3.0 2.5 2.0 1.5 1.0 0.5 0.0 ppm
1.906
1.976
1.966
1.974
2.037
2.000

¹H NMR

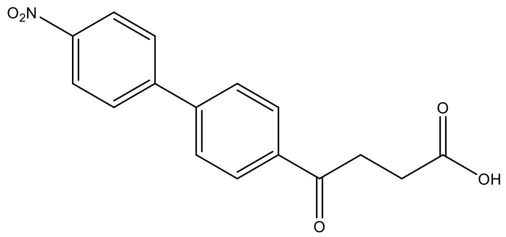
O2N
O
OH
O

6n

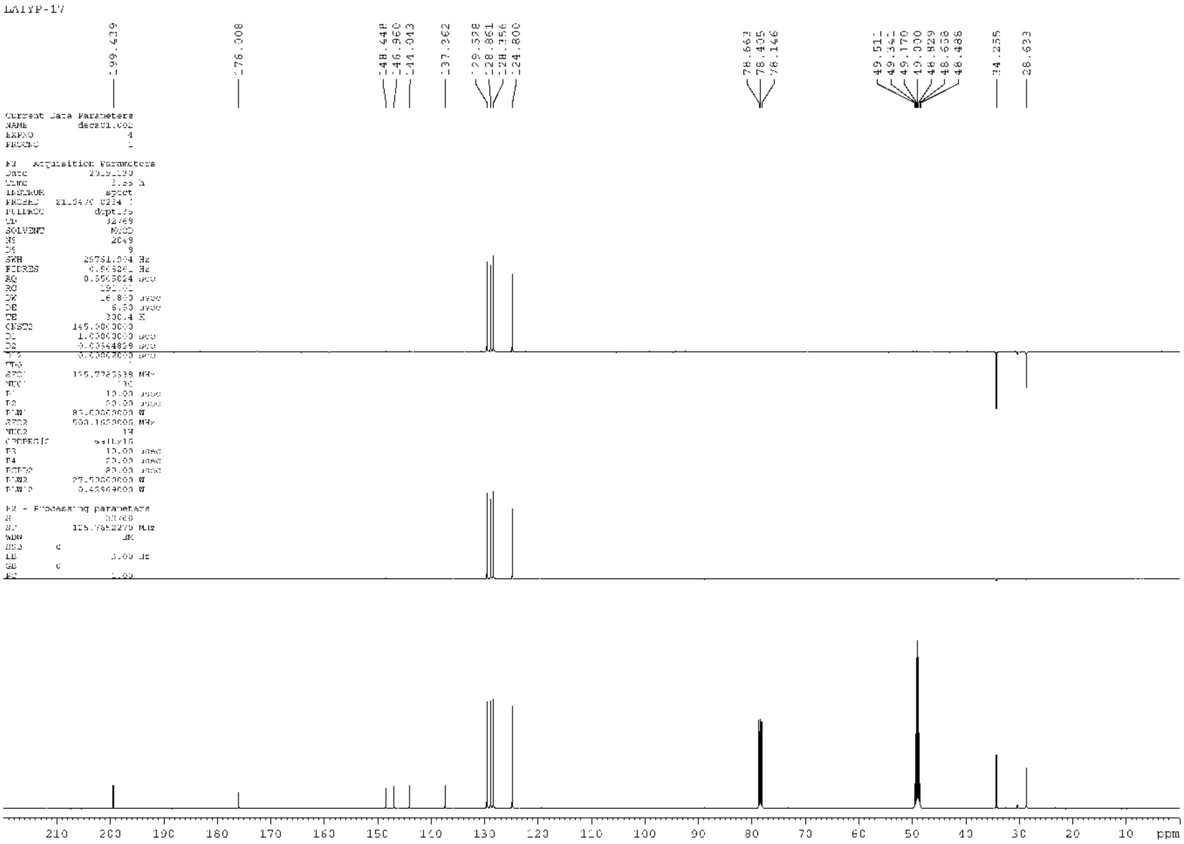

^{13}C-DEPT-135 NMR

6n

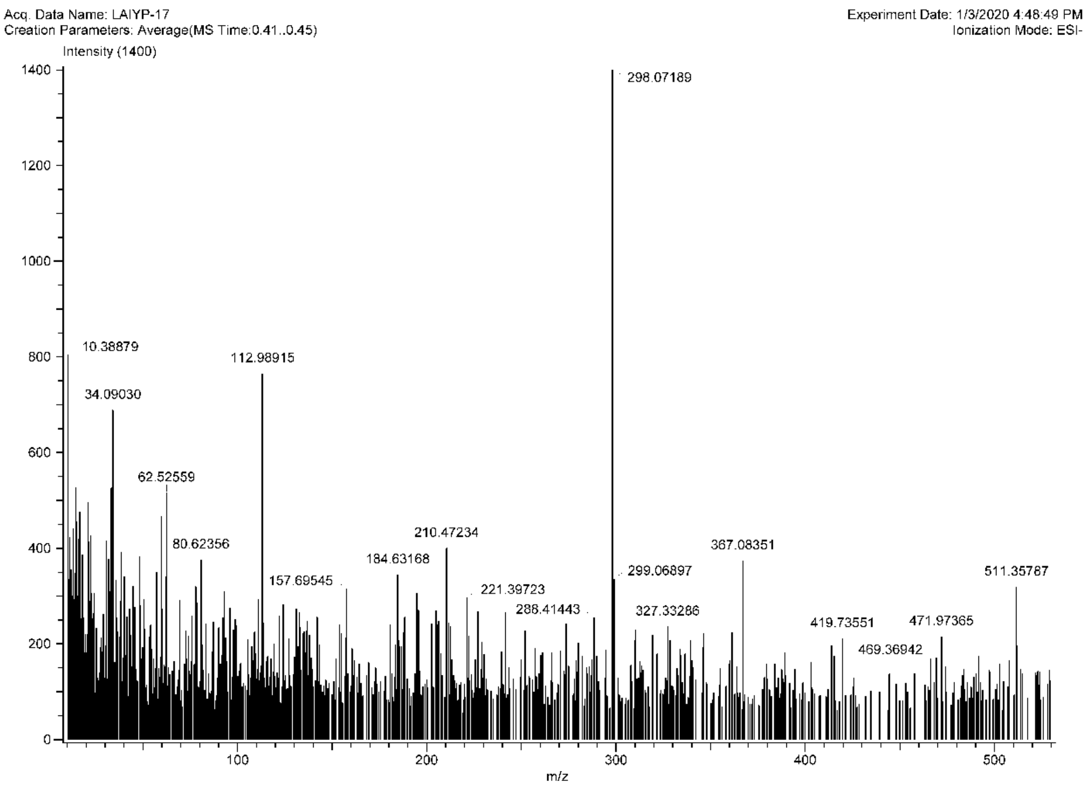
Acq. Data Name: LAIYP-17
Creation Parameters: Average(MS Time:0.41..0.45)
Experiment Date: 1/3/2020 4:48:49 PM
Ionization Mode: ESI-
Intensity (1400)
298.07189
10.38879
112.98915
34.09030
62.52559
80.62356
210.47234
367.08351
184.63168
157.69545
299.06897
511.35787
221.39723
288.41443
327.33286
419.73551
471.97365
469.36942
m/z

HR ESI-MS

6n

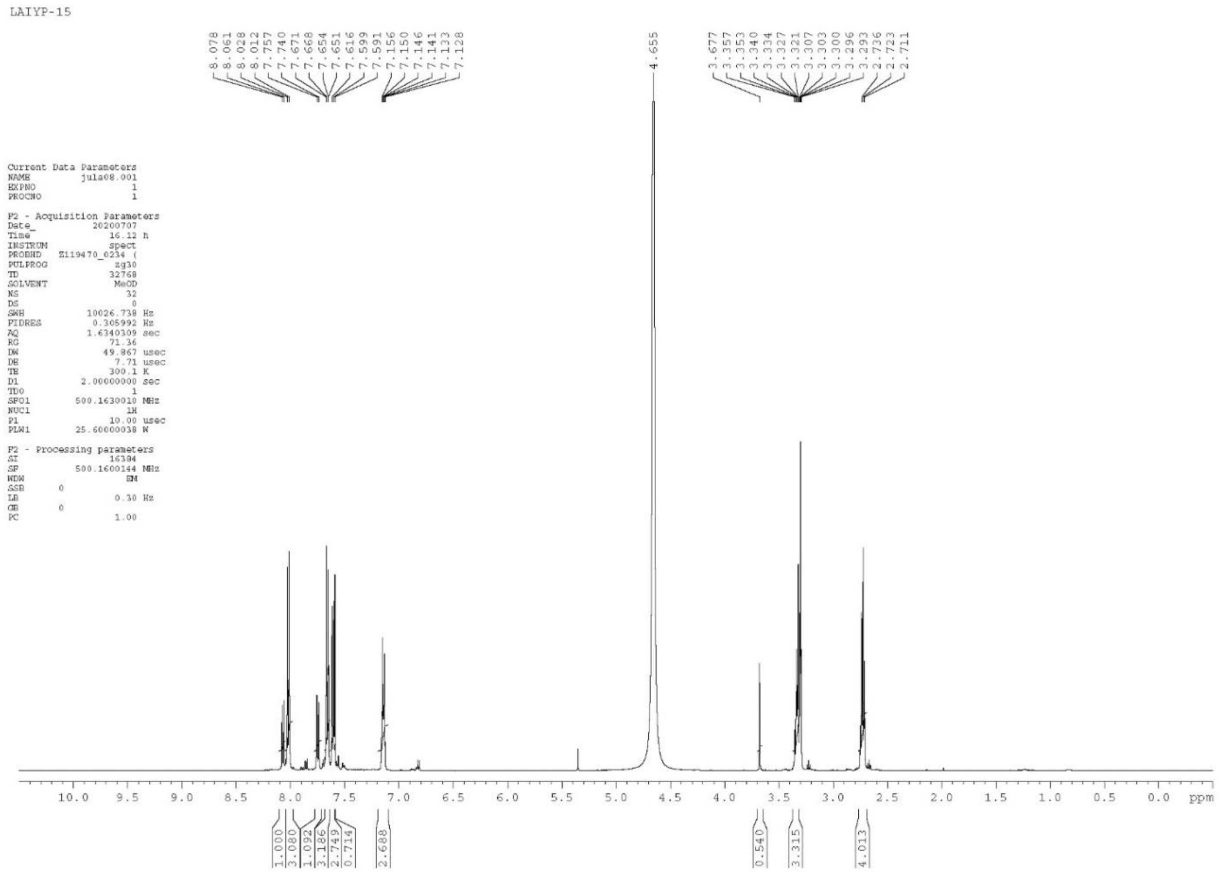

^{1}H NMR

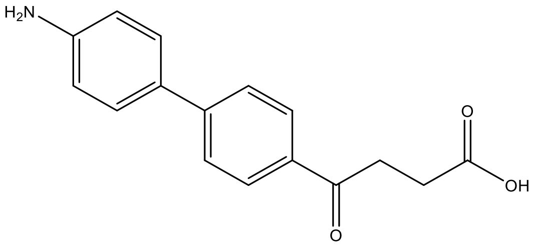

6o

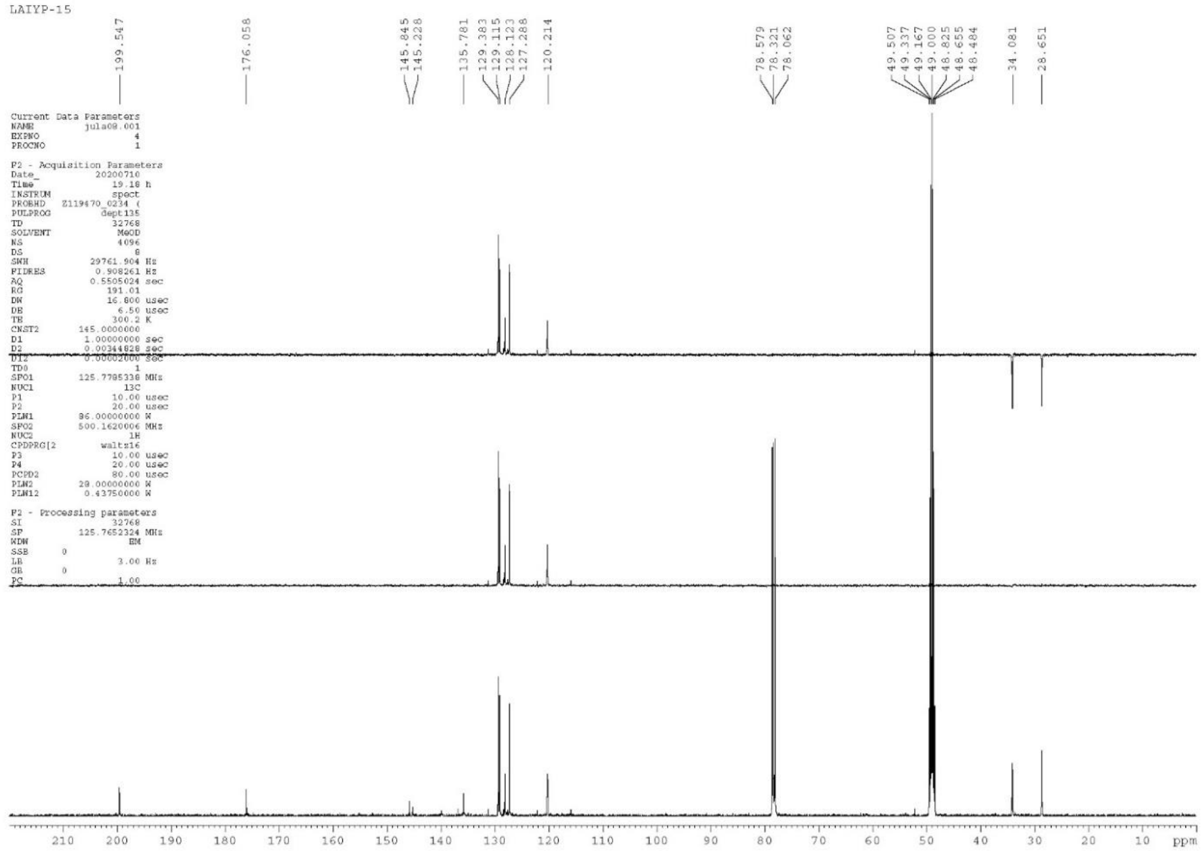

^{13}C-DEPT-135 NMR

6o

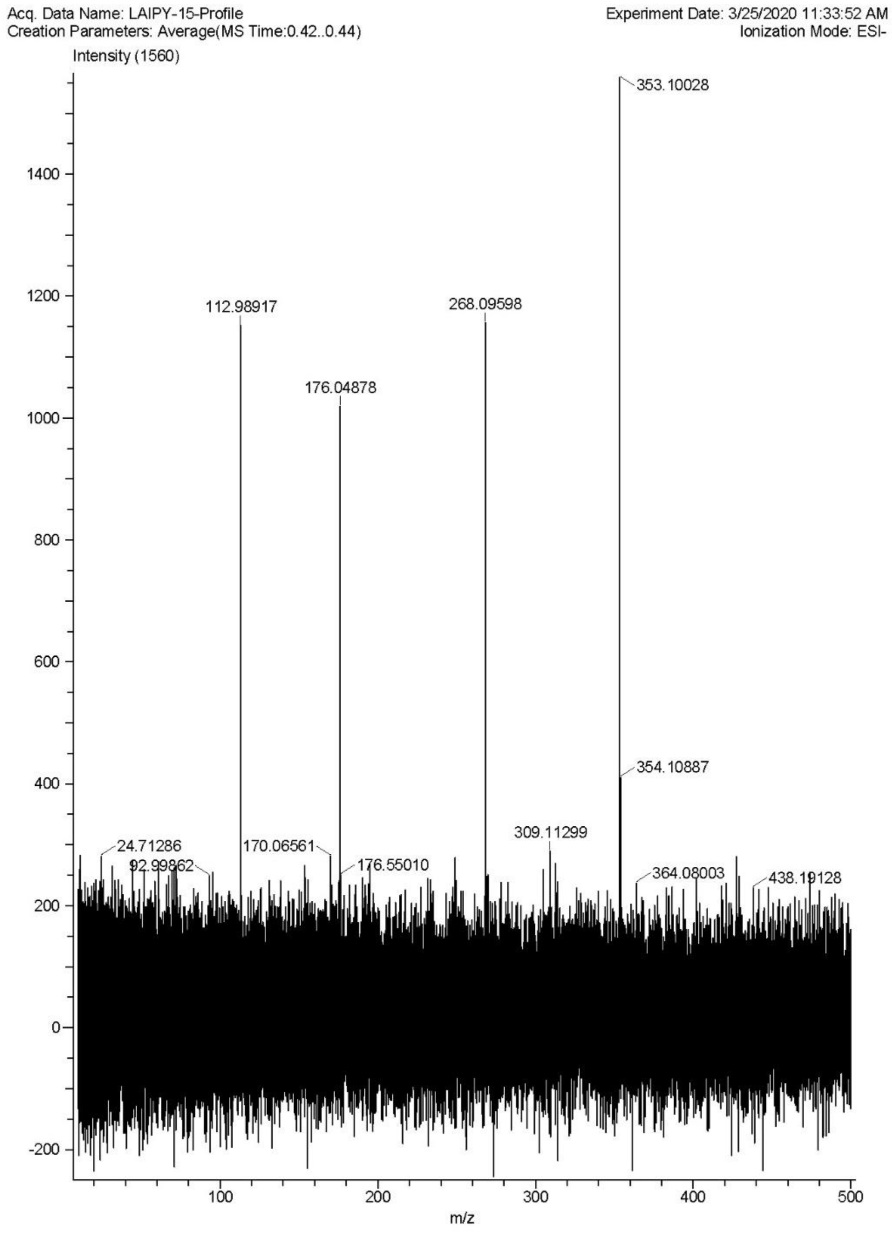

Acq. Data Name: LAIPY-15-Profile
Creation Parameters: Average(MS Time:0.42..0.44)
Experiment Date: 3/25/2020 11:33:52 AM
Ionization Mode: ESI-
Intensity (1560)
353.10028
112.98917
268.09598
176.04878
354.10887
24.71286
92.99862
170.06561
176.55010
309.11299
364.08003
438.19128
m/z

HR ESI-MS

6o

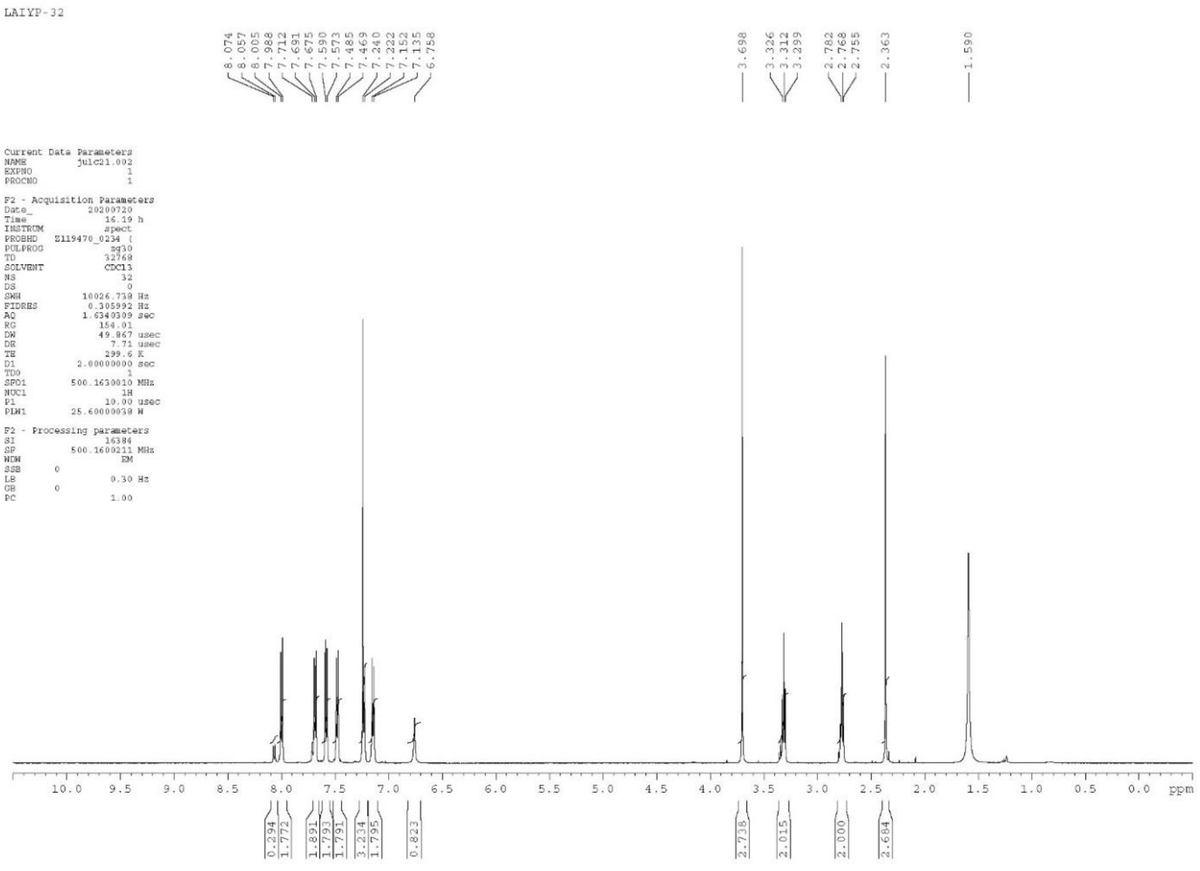
LAIYP-32
8.074
8.057
8.005
7.988
7.712
7.691
7.675
7.590
7.573
7.485
7.469
7.240
7.222
7.152
7.135
6.758
3.698
3.326
3.312
3.299
2.782
2.768
2.755
2.363
1.590
10.0 9.5 9.0 8.5 8.0 7.5 7.0 6.5 6.0 5.5 5.0 4.5 4.0 3.5 3.0 2.5 2.0 1.5 1.0 0.5 0.0 ppm
0.294
1.772
1.891
1.793
1.791
3.234
1.795
0.823
2.738
2.015
2.000
2.684

^{1}H NMR

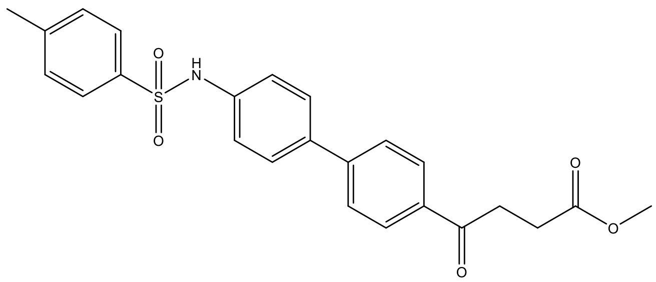
O
H
N
S
O
O
O
O
O

7

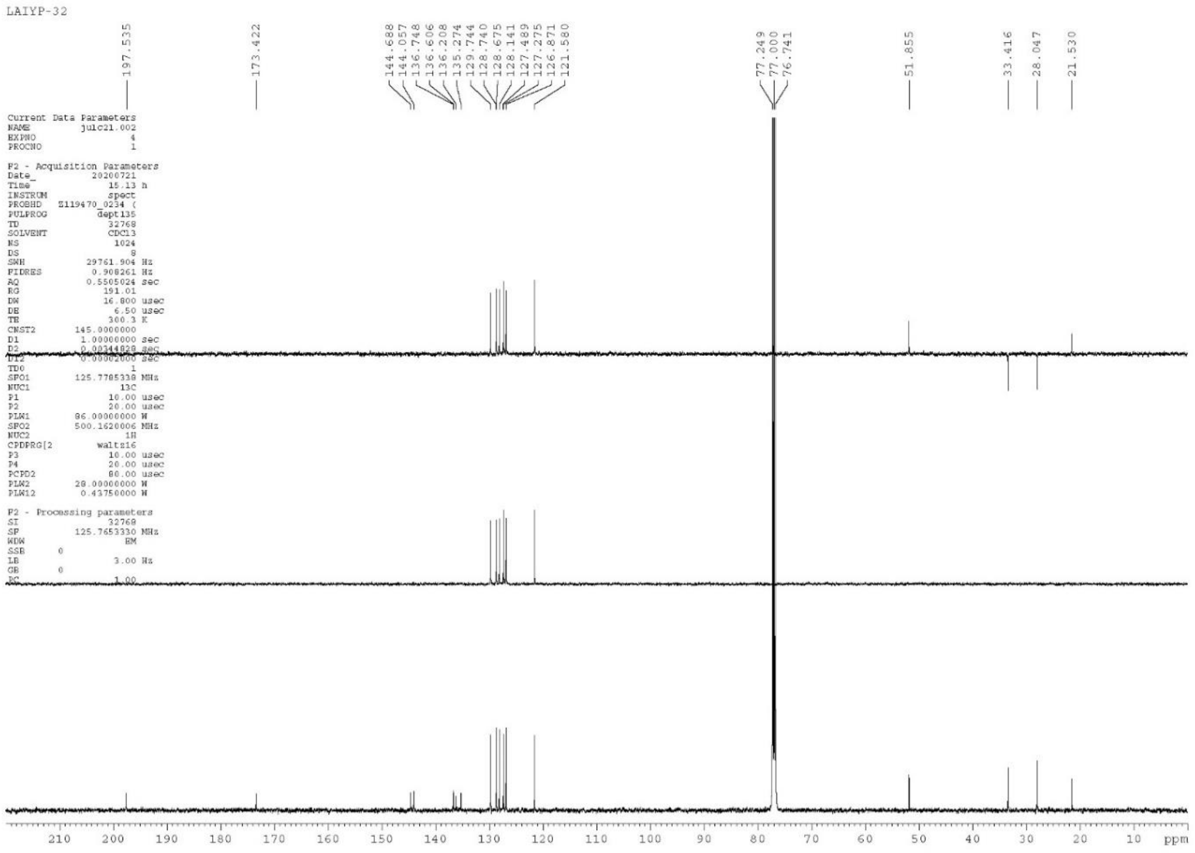

^{13}C-DEPT-135 NMR

7

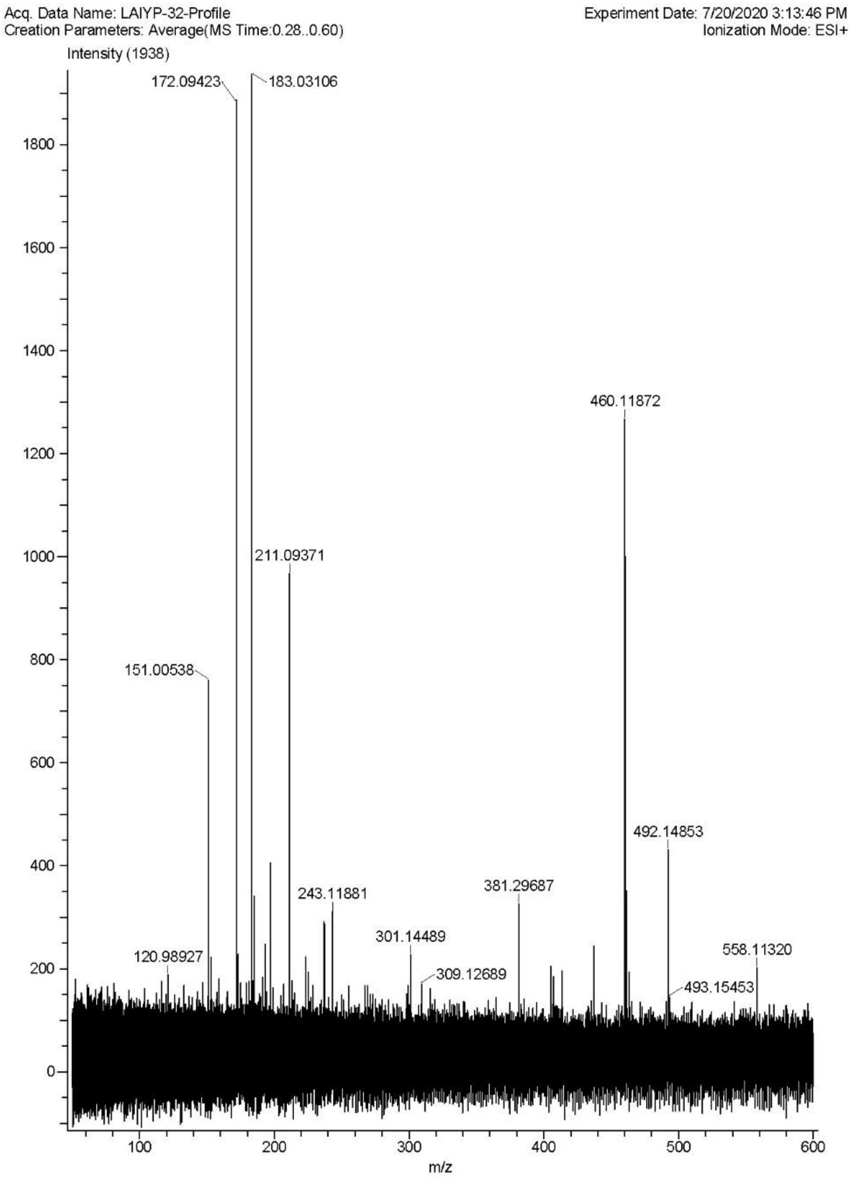

Acq. Data Name: LAIYP-32-Profile
Creation Parameters: Average(MS Time:0.28..0.60)
Experiment Date: 7/20/2020 3:13:46 PM
Ionization Mode: ESI+
Intensity (1938)
172.09423
183.03106
460.11872
211.09371
151.00538
492.14853
243.11881
381.29687
301.14489
120.98927
558.11320
309.12689
493.15453
m/z

HR-ESI-MS

7

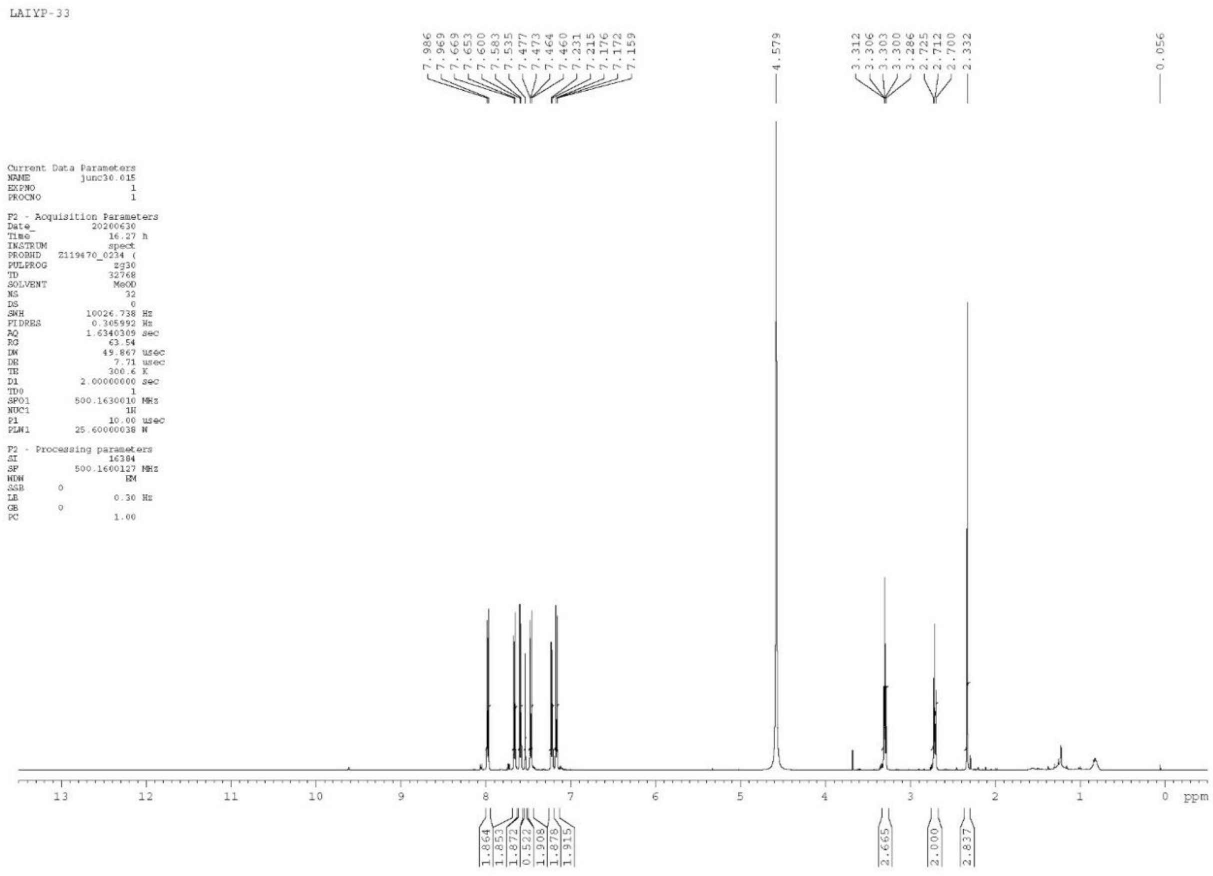
LAIYP-33
7.986
7.969
7.669
7.653
7.600
7.583
7.535
7.477
7.473
7.464
7.460
7.231
7.215
7.176
7.172
7.159
4.579
3.312
3.306
3.303
3.300
3.286
2.725
2.712
2.700
2.332
0.056
Current Data Parameters
NAME junc30.015
EXPNO 1
PROCNO 1
F2 - Acquisition Parameters
Date_ 20200630
Time 16.27 h
INSTRUM spect
PROBHD Z119470_0234 (
PULPROG zg30
TD 32768
SOLVENT MeOD
NS 32
DS 0
SWH 10026.738 Hz
FIDRES 0.305992 Hz
AQ 1.6340309 sec
RG 63.54
DW 49.867 usec
DE 7.71 usec
TE 300.6 K
D1 2.00000000 sec
TD0 1
SFO1 500.1630010 MHz
NUC1 1H
P1 10.00 usec
PLW1 25.60000038 W
F2 - Processing parameters
SI 16384
SF 500.1600127 MHz
WDW EM
SSB 0
LB 0.30 Hz
GB 0
PC 1.00
13 12 11 10 9 8 7 6 5 4 3 2 1 0 ppm
1.864
1.853
1.872
0.522
1.908
1.878
1.915
2.665
3.000
2.837

^{1}H NMR

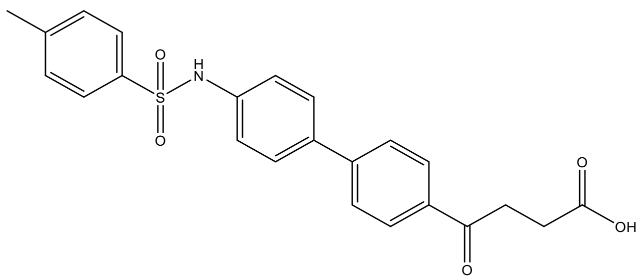
O
S
O
H
N
O
OH
O

8

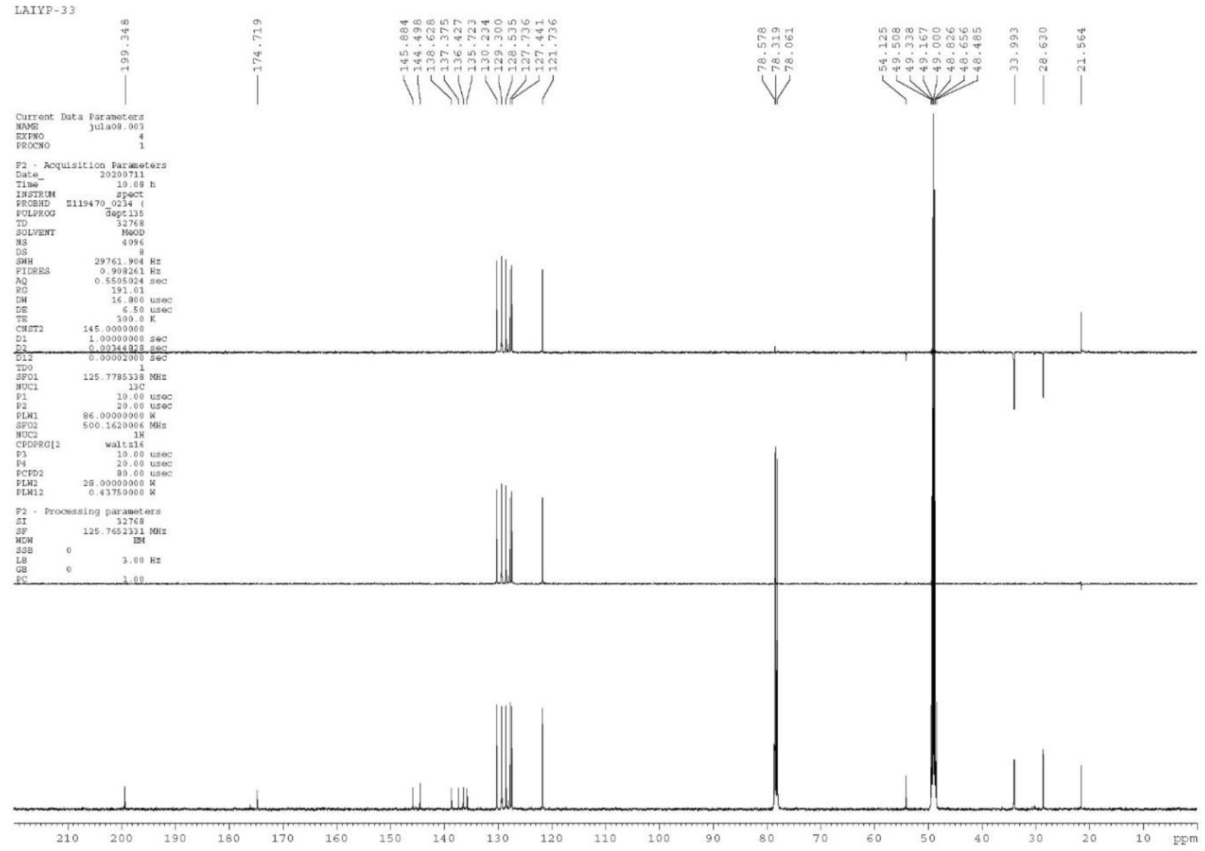

^{13}C-DEPT-135 NMR

8

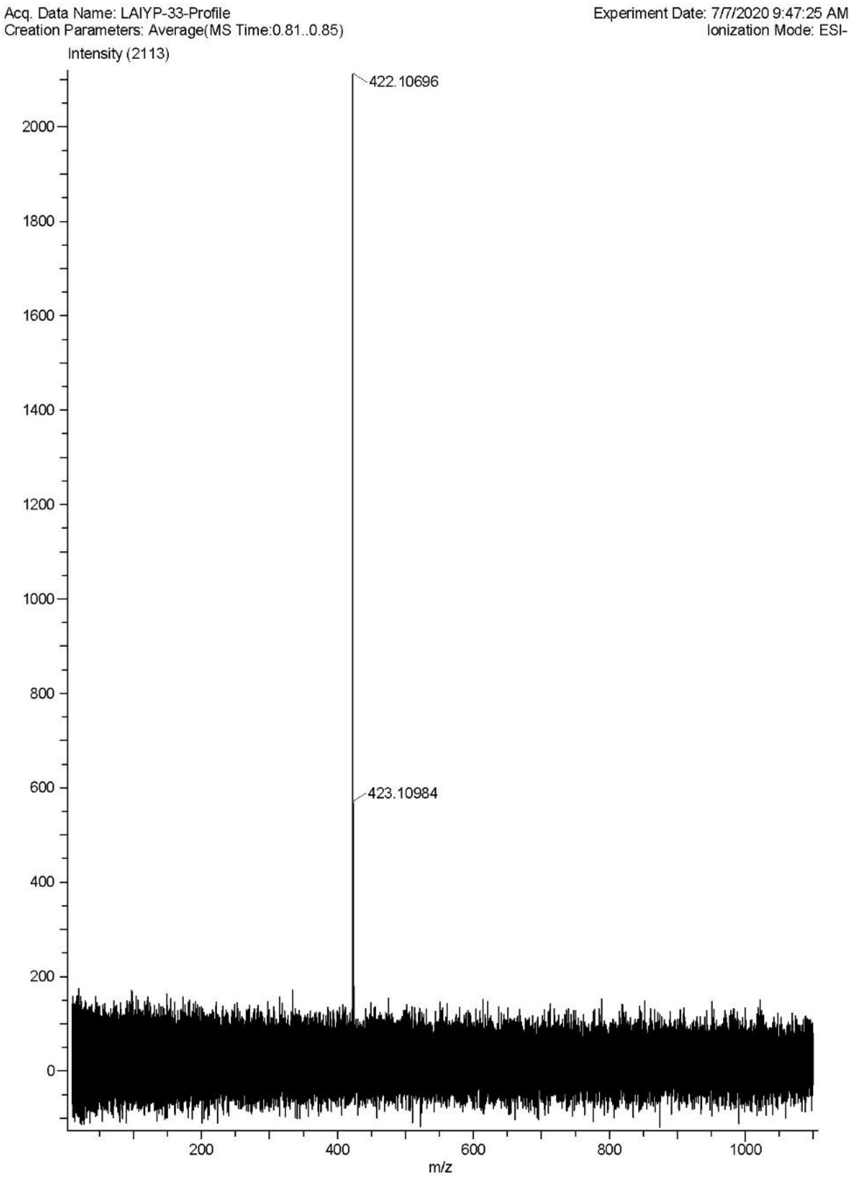
Acq. Data Name: LAIYP-33-Profile
Creation Parameters: Average(MS Time:0.81..0.85)
Experiment Date: 7/7/2020 9:47:25 AM
Ionization Mode: ESI-
Intensity (2113)
422.10696
423.10984
m/z

HR-ESI-MS

8

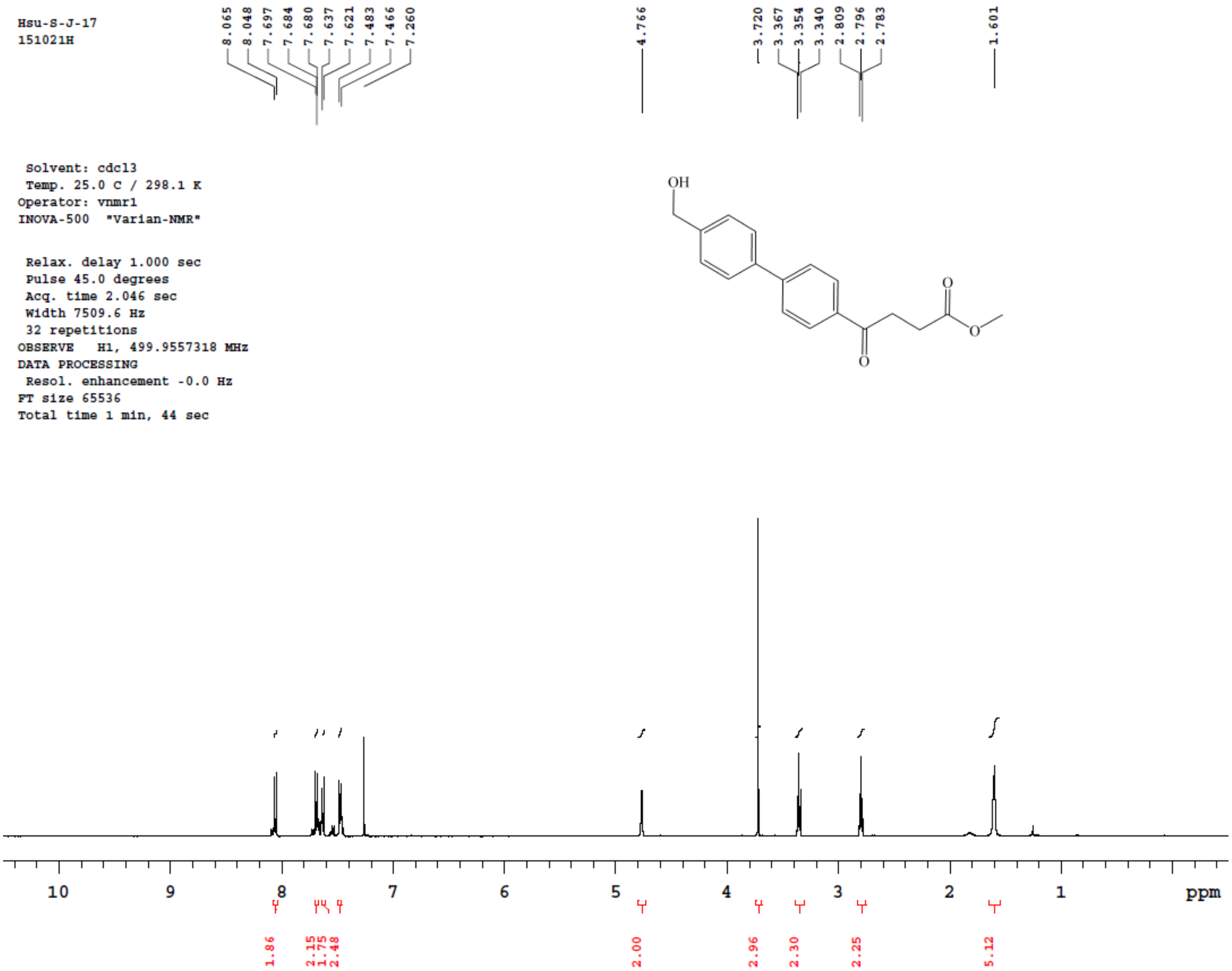
Hsu-S-J-17
151021H
8.065
8.048
7.697
7.684
7.680
7.637
7.621
7.483
7.466
7.260
4.766
3.720
3.367
3.354
3.340
2.809
2.796
2.783
1.601
Solvent: cdcl3
Temp. 25.0 C / 298.1 K
Operator: vnmr1
INOVA-500 "Varian-NMR"
Relax. delay 1.000 sec
Pulse 45.0 degrees
Acq. time 2.046 sec
Width 7509.6 Hz
32 repetitions
OBSERVE H1, 499.9557318 MHz
DATA PROCESSING
Resol. enhancement -0.0 Hz
FT size 65536
Total time 1 min, 44 sec
10
9
8
7
6
5
4
3
2
1
ppm
1.86
2.15
1.75
2.48
2.00
2.96
2.30
2.25
5.12
OH
O
O
O

^{1}H-NMR

12

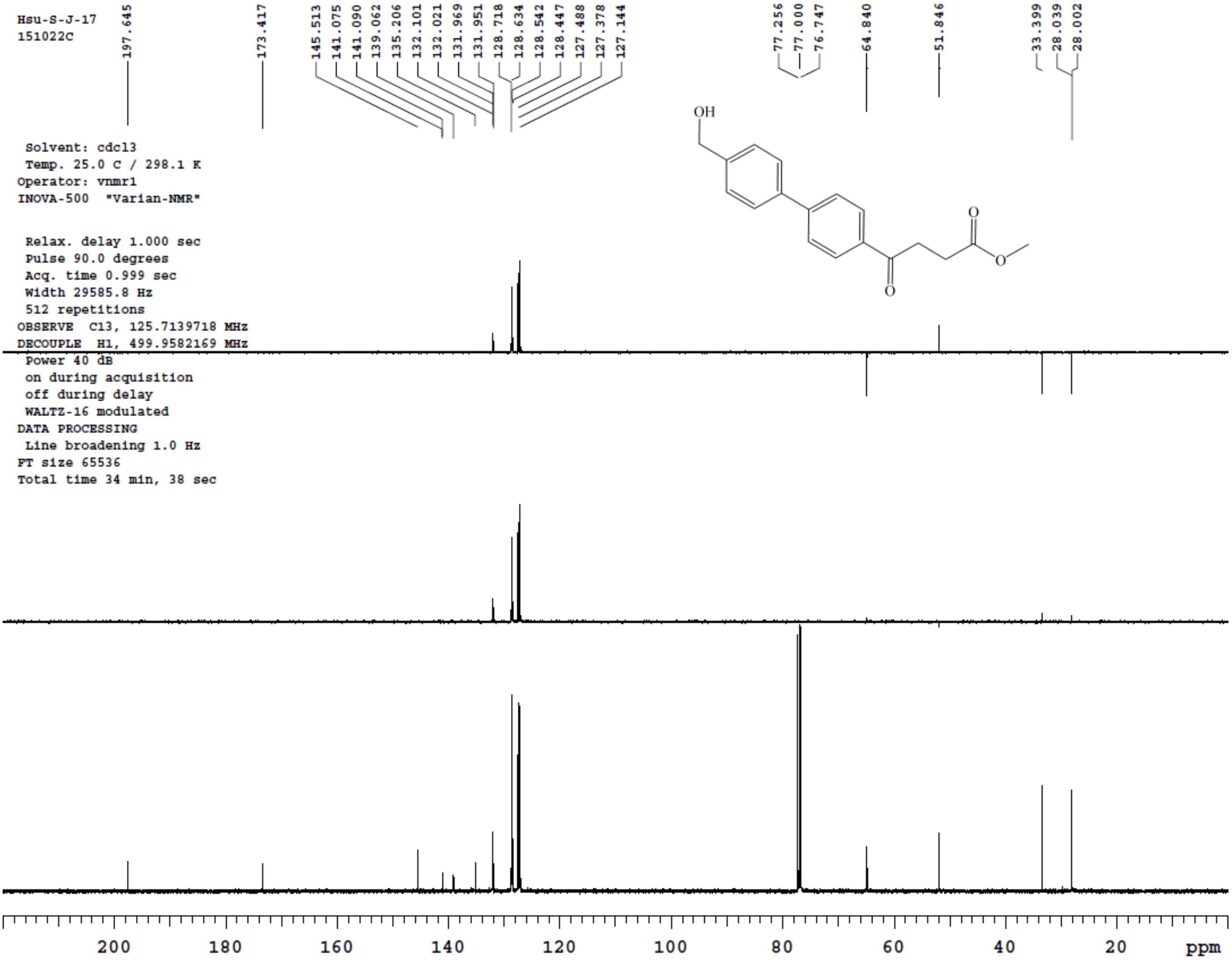

^{13}C-DEPT-135 NMR

12

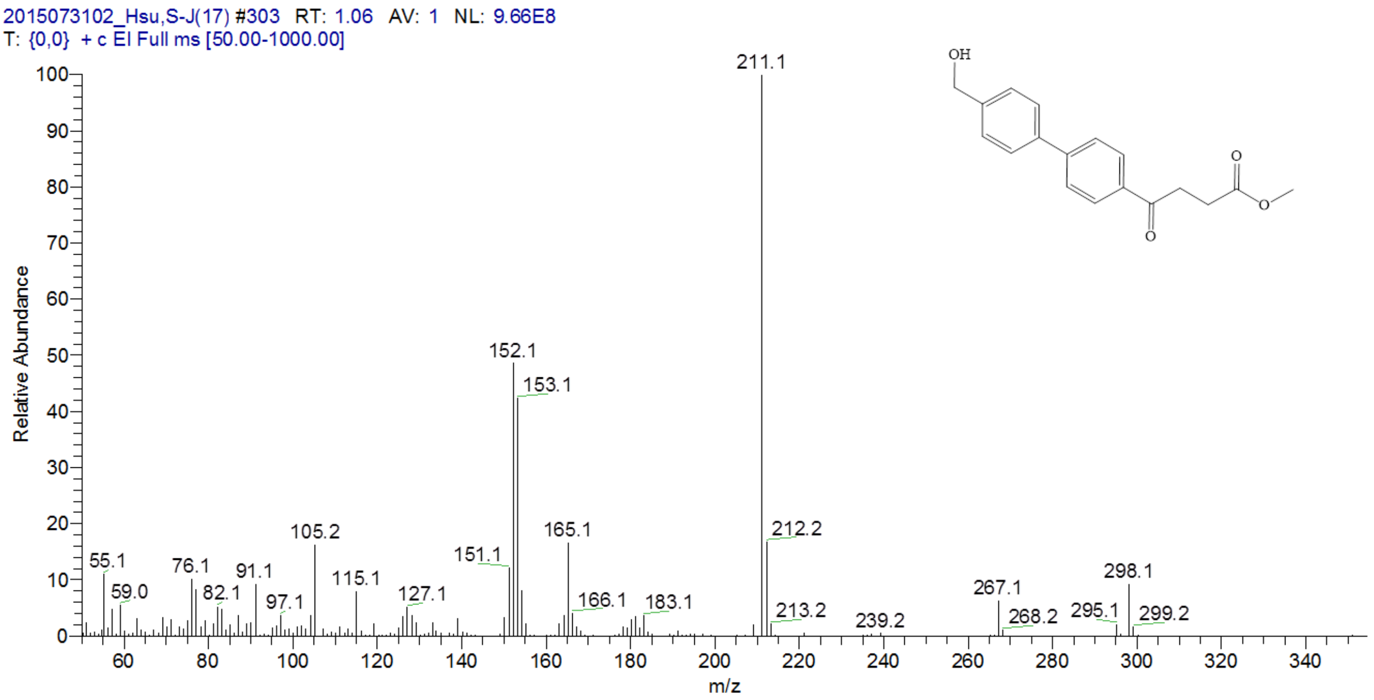

2015073102_Hsu,S-J(17) #303 RT: 1.06 AV: 1 NL: 9.66E8
T: {0,0} + c EI Full ms [50.00-1000.00]
Relative Abundance
m/z
211.1
152.1
153.1
165.1
105.2
212.2
151.1
55.1
76.1
91.1
115.1
82.1
59.0
127.1
97.1
166.1
183.1
213.2
239.2
267.1
268.2
295.1
298.1
299.2
OH
O
O
O

LR-ESI-MS

12

國立中興大學研發處貴重儀器使用中心
元素分析儀服務報告書

說明:

1.本實驗數據為檢測結果，不得用於商業廣告、認證及法律証據使用 。(This result is for academic use only, not to be used for any judicial or commercial advertising purpose.)

2.儀器負責人: 鄭政峯 教授　檢測技術員: 陳宜絹。
(Instrument Director : Prof. Jen-Fon Jen　Operator : I-Chuan Chen)

樣品資訊 :

Web NO	SEA00010020150100139		
Department :	中興化研所		DATE
Supervisor :	柯寶燦	收件日 :	2015.10.19
User name :	林佩旻	分析日 :	2015.10.19

分析結果 :

Sample code	Weight(mg)	N %	C %	H %	O %	S %	Repeat	Charge
1	2.739<br>2.755		72.25<br>72.41	6.06<br>6.14			1	$ 1,500
推測值			72.47	6.08				

備註:

使用儀器: Elementar vario EL III(CHN-OS Rapid, German), Accuracy: 0.1%, Precision: 0.2%

	標準品	N %	C %	H %	O %	S %
★	Acetanilid	10.36	71.09	6.71		
	Benzoic acid				26.20	
	Sulfanilic acid	8.09	41.60	4.07		18.50
	Daily standard	10.40	71.01	6.79		

特殊建議: 無

★本服務報告書共 1 頁, 本次實驗共計 1 件, 總計金額新台幣:1,500 元

Elemental analysis

12

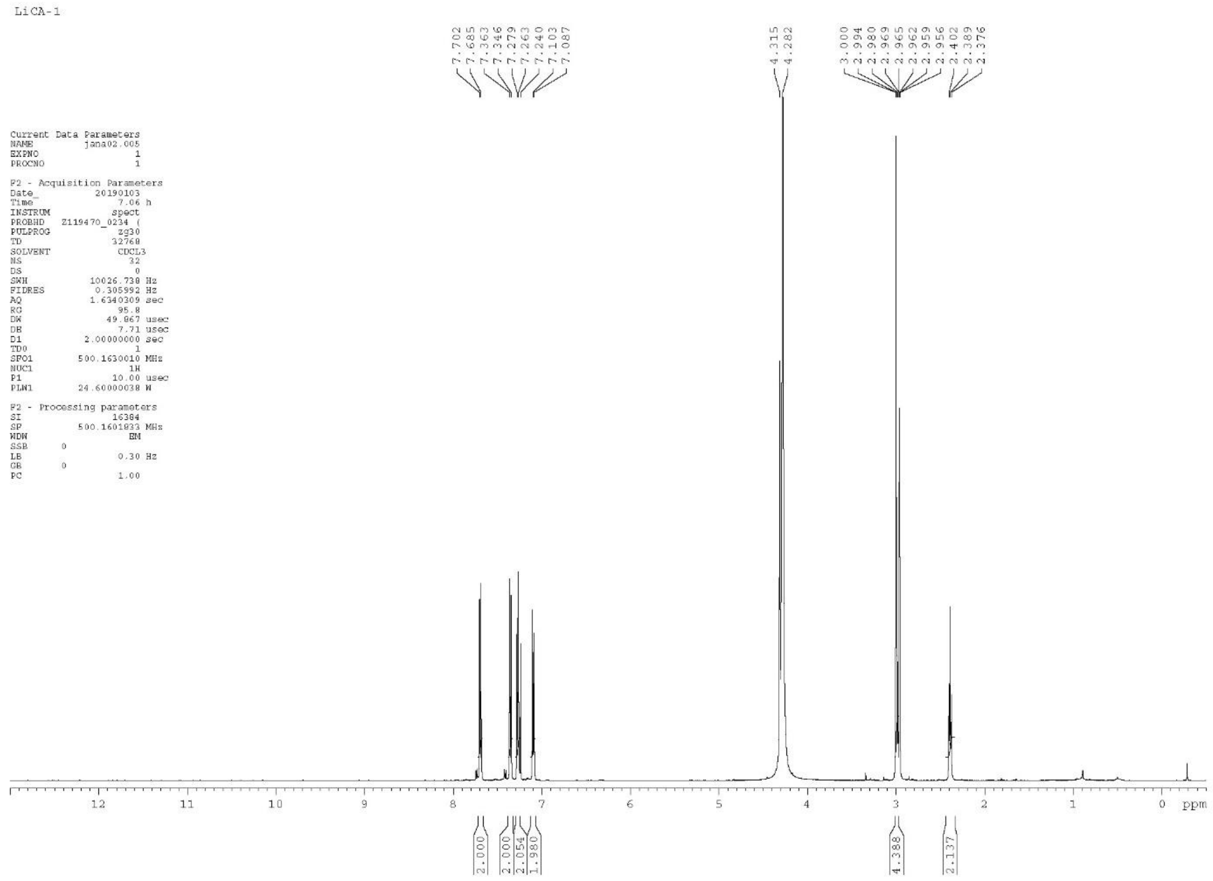
LiCA-1
Current Data Parameters
NAME jana02.005
EXPNO 1
PROCNO 1
F2 - Acquisition Parameters
Date_ 20190103
Time 7.06 h
INSTRUM spect
PULPROG zg30
TD 32768
SOLVENT CDCL3
NS 32
DS 0
SWH 10026.738 Hz
FIDRES 0.305992 Hz
AQ 1.6340309 sec
RG 95.8
DW 49.867 usec
DE 7.71 usec
D1 2.00000000 sec
TD0 1
SFO1 500.1630010 MHz
NUC1 1H
P1 10.00 usec
PLW1 24.60000038 W
F2 - Processing parameters
SI 16384
SP 500.1601833 MHz
WDW EM
SSB 0
LB 0.30 Hz
GB 0
PC 1.00
ppm

^{1}H NMR

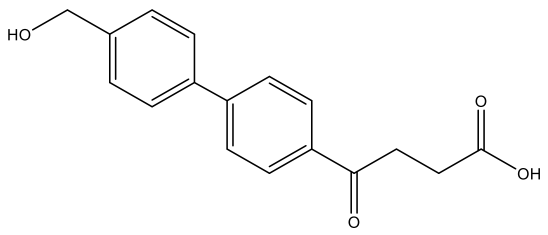
HO
O
OH
O

11

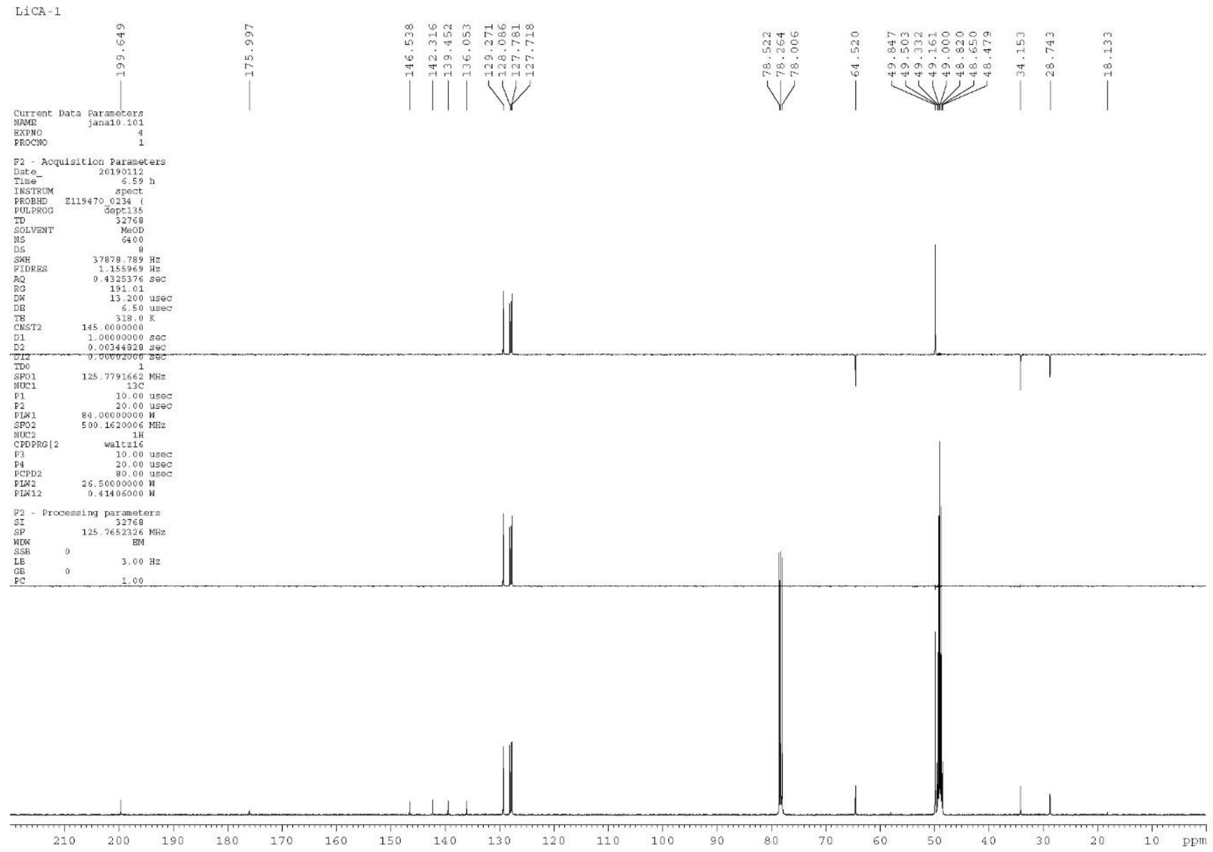

^{13}C-DEPT-135 NMR

11

Display Report

Analysis Info

Analysis Name	D:\Data\NCTU SERVICE\Data\2019\20190104\LiCA-1_GD4_01_21766.d	Acquisition Date	1/4/2019 12:23:35 PM
Method	Small molecule.m	Operator	NCTU
Sample Name	LiCA-1	Instrument	impact HD 1819696.00164
Comment			

Acquisition Parameter

Source Type	ESI	Ion Polarity	Positive	Set Nebulizer	1.0 Bar
Focus	Active	Set Capillary	4500 V	Set Dry Heater	200 °C
Scan Begin	50 m/z	Set End Plate Offset	-500 V	Set Dry Gas	6.0 l/min
Scan End	1500 m/z	Set Charging Voltage	2000 V	Set Divert Valve	Waste
		Set Corona	0 nA	Set APCI Heater	0 °C

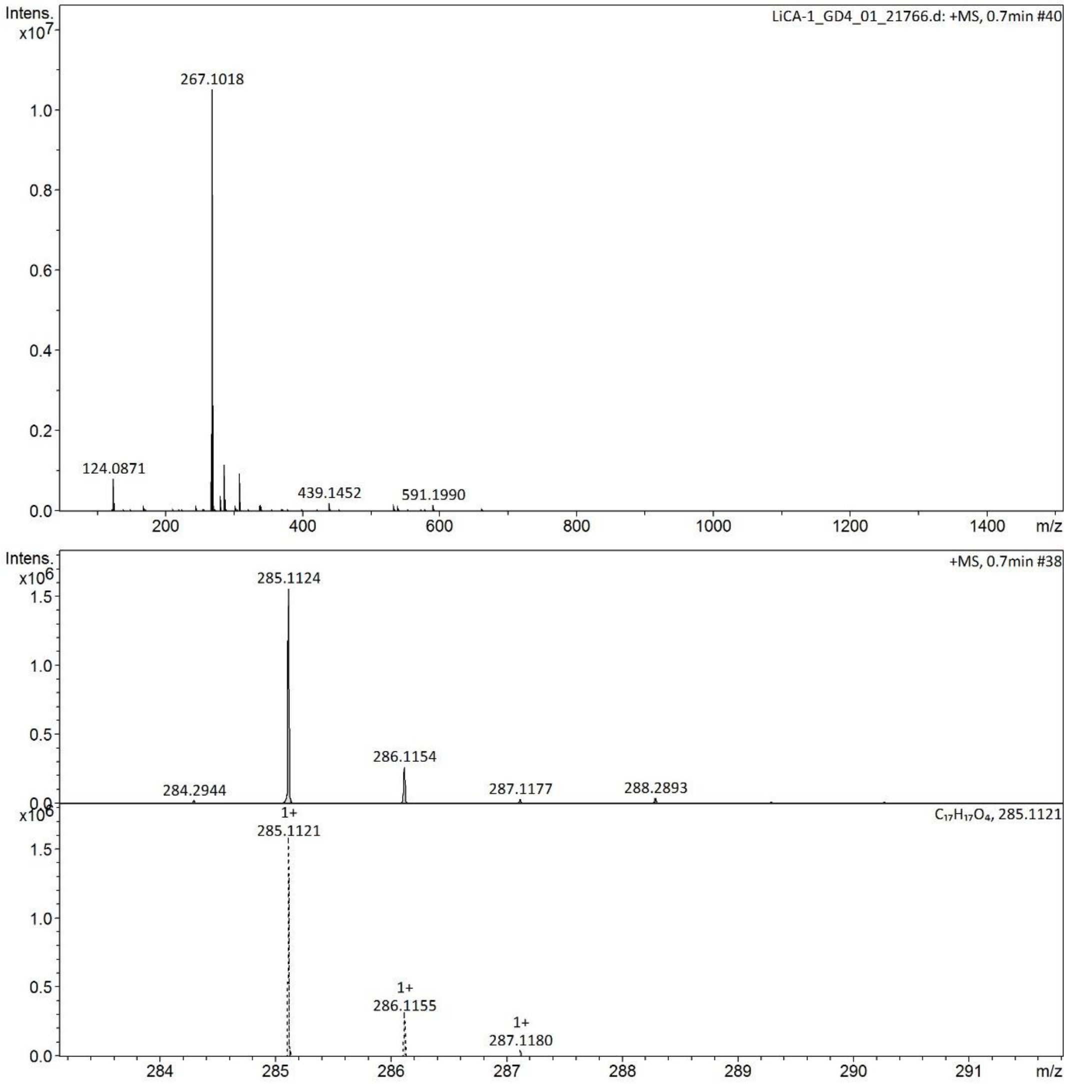

HR-ESI-MS

11

2. COX-1 or COX-2 inhibitor library assay protocol

1) Preparation stage

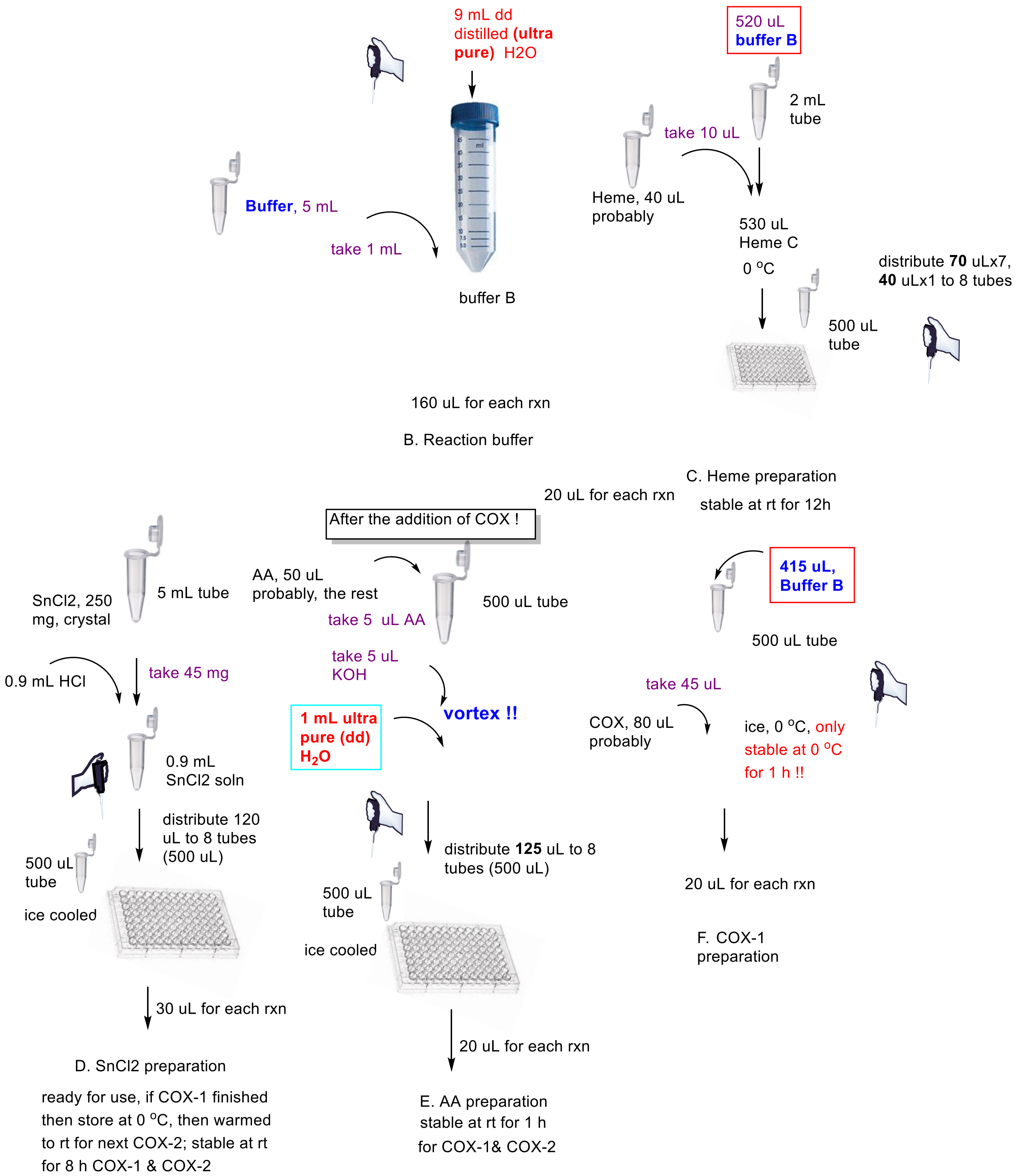

2) Performing stage

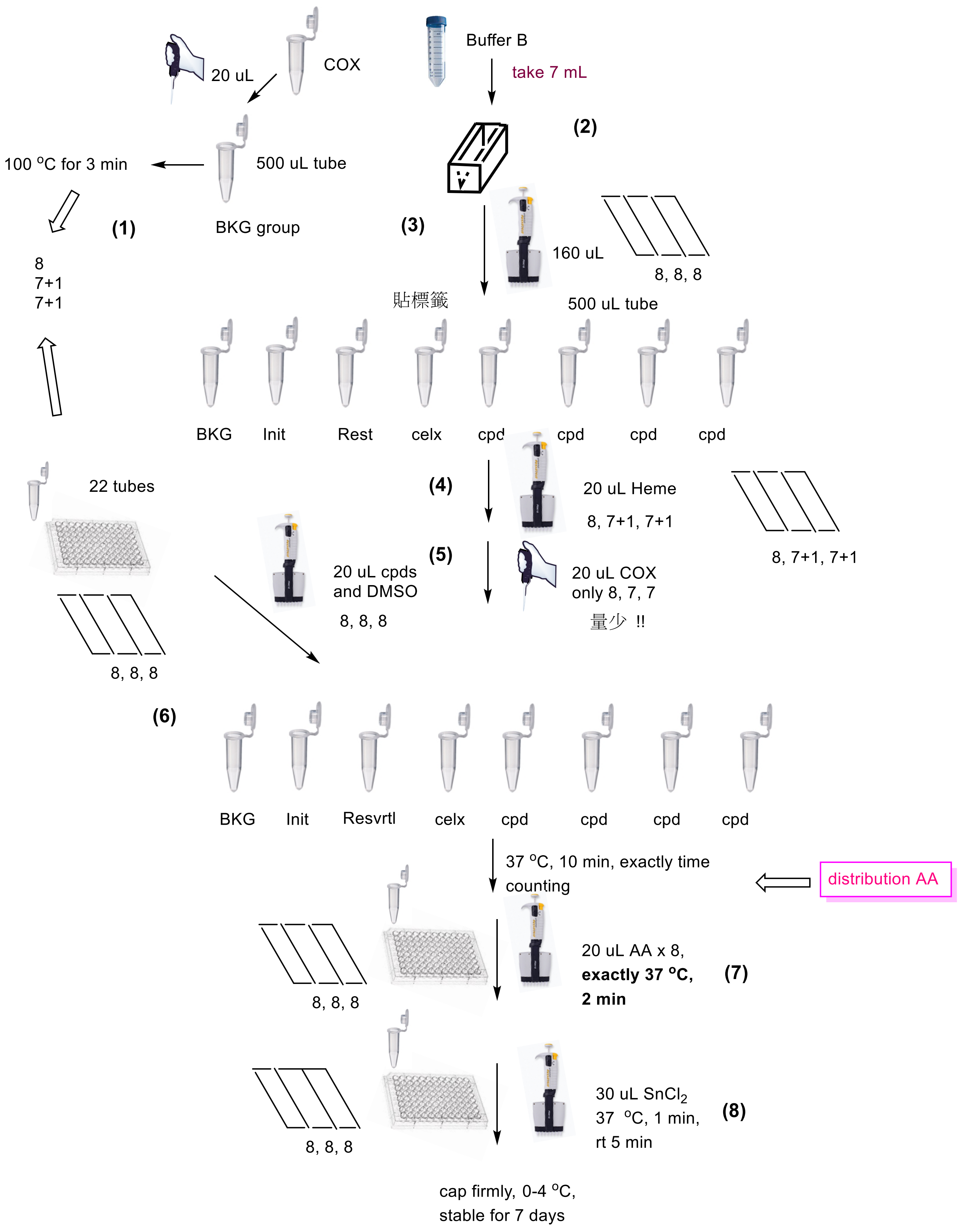

3) Elisa assay stage

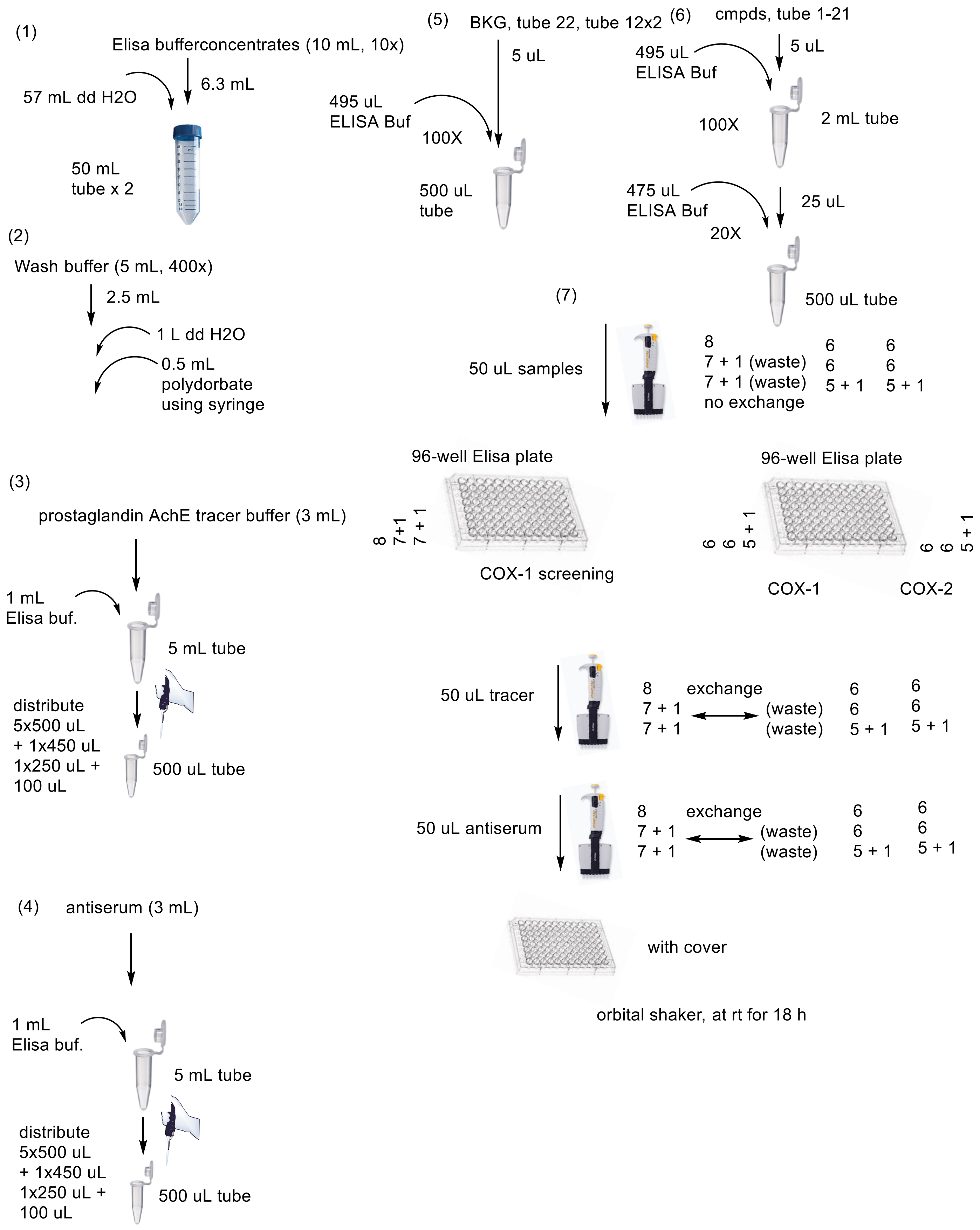

4) Development stage

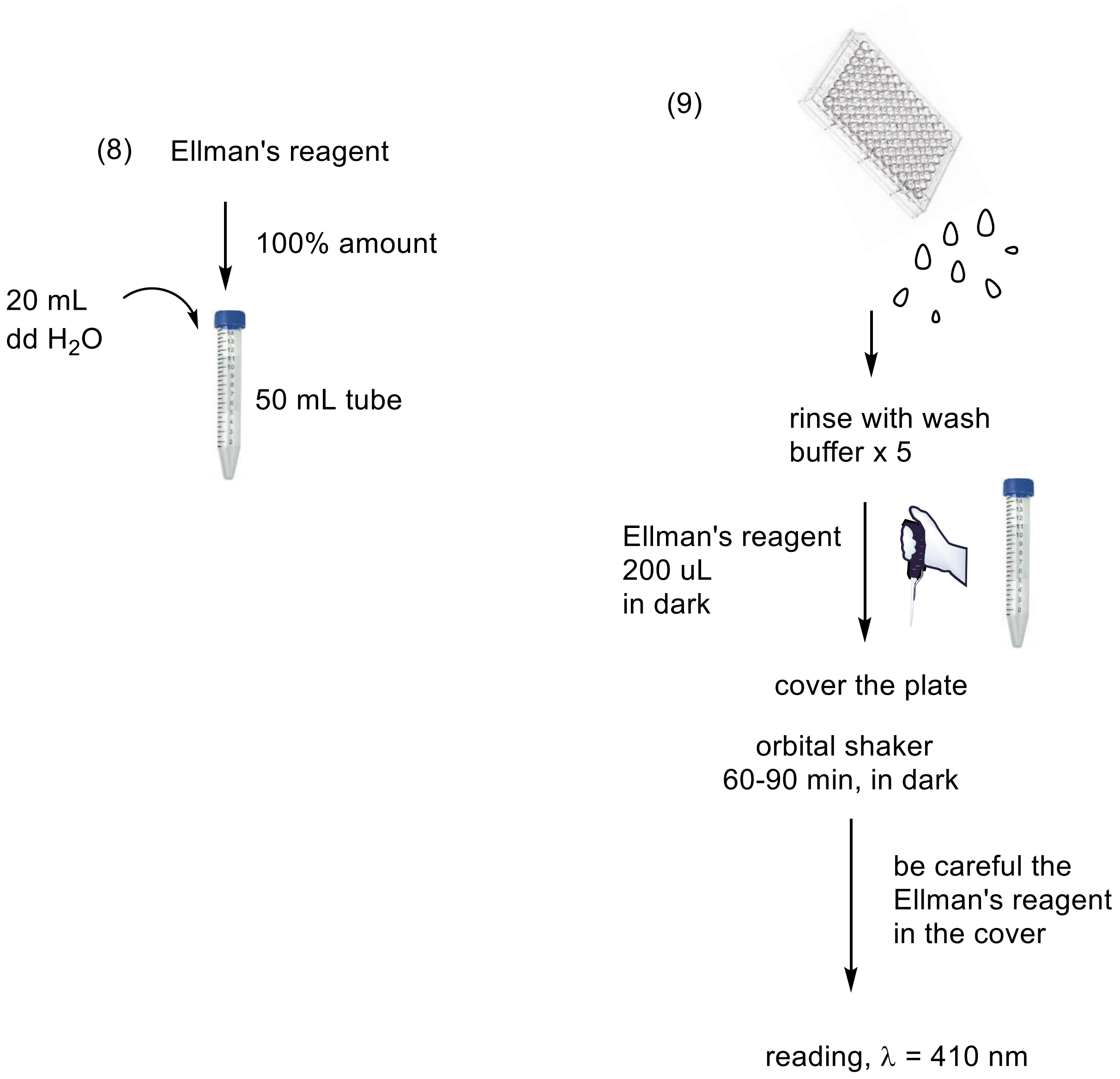

Supplement: Supplementary file 1 [file molecules-27-02850-s001.zip › molecules-1662876-supplementary.pdf]
